# Taxonomy, natural history, and conservation of the mammals of Honduras

**DOI:** 10.3897/zookeys.1288.129555

**Published:** 2026-08-07

**Authors:** Manfredo A. Turcios-Casco, Franklin Castañeda, Don E. Wilson, Bruce Miller, Daniel Gonzalez-Socoloske, Eric A. Ramos, Travis W. King, Richard K. LaVal, Nicté Ordóñez-Garza, Danny Ordoñez, Gustavo Graciolli, Daniel Thornton, Marcio Martínez, Nereyda M. Estrada-Andino, Hefer D. Ávila-Palma

**Affiliations:** 1 Asociación para la Sostenibilidad e Investigación Científica en Honduras (ASICH), Barrio La Granja, entre calles 28 y 29, Comayagüela M.D.C., Francisco Morazán, Honduras Department of Ecology and Evolutionary Biology, University of Michigan Ann Arbor United States of America; 2 Departamento de Ciências Biológicas, Universidade Estadual de Santa Cruz, Ilhéus, BA, Brazil Centro Universitario de Zacapa, Universidad de San Carlos de Guatemala Zacapa Guatemala; 3 Wildlife Conservation Society (WCS), Programa Honduras, Tegucigalpa, Honduras Division of Mammals, National Museum of Natural History, Smithsonian Institution Washington United States of America; 4 Panthera, Col. Los Robles, Bloque L, 3415, Tegucigalpa, Honduras Departamento de Ciências Biológicas, Universidade Estadual de Santa Cruz Ilhéus Brazil; 5 Division of Mammals, National Museum of Natural History, Smithsonian Institution, Washington, D.C., USA Mote Marine Laboratory and Aquarium Sarasota United States of America; 6 Neotropical Bat Acoustic Assessment Projects, Canadian Lakes, Michigan, USA Universidade Federal de Mato Grosso do Sul (UFMS) Campo Grande Brazil; 7 Department of Biology, Andrews University, Berrien Springs, Michigan, USA Escuela de Biología, Universidad Nacional Autónoma de Honduras (UNAH) Tegucigalpa Honduras; 8 Mote Marine Laboratory and Aquarium, Sarasota, Florida, USA Department of Biology, Andrews University Berrien Springs United States of America; 9 Washington State University, Pullman, Washington, USA Programa de Pós-graduação em Ecologia, Conservação e Biodiversidade, Universidade Federal de Uberlândia Minas Gerais Brazil; 10 Monteverde Bat Jungle, Monteverde, Costa Rica Washington State University Pullman United States of America; 11 Department of Ecology and Evolutionary Biology, University of Michigan, Ann Arbor, MI, USA Wildlife Conservation Society (WCS), Programa Honduras Tegucigalpa Honduras; 12 Centro Universitario de Zacapa, Universidad de San Carlos de Guatemala, Guatemala City, Guatemala Asociación para la Sostenibilidad e Investigación Científica en Honduras (ASICH) Francisco Morazán Honduras; 13 Programa de Pós-graduação em Ecologia, Conservação e Biodiversidade, Universidade Federal de Uberlândia, Minas Gerais, Brasil Panthera Tegucigalpa Honduras; 14 Universidade Federal de Mato Grosso do Sul (UFMS), Campo Grande, Mato Grosso do Sul, Brazil Neotropical Bat Acoustic Assessment Projects Michigan United States of America; 15 Departamento de Vida Silvestre, Instituto Nacional de Conservación y Desarrollo Forestal, Áreas Protegidas y Vida Silvestre (ICF), Brisas de Olancho, Honduras Monteverde Bat Jungle Monteverde Costa Rica; 16 Escuela de Biología, Universidad Nacional Autónoma de Honduras (UNAH), Boulevard Suyapa, Tegucigalpa M.D.C., Honduras Departamento de Vida Silvestre, Instituto Nacional de Conservación y Desarrollo Forestal, Áreas Protegidas y Vida Silvestre (ICF) Brisas de Olancho Honduras

**Keywords:** Biological monitoring, environmental safeguarding, Honduran biodiversity, Mammalia, systematic revision

## Abstract

Our account of the mammals of Honduras presents distribution based on verifiable data, with notes on natural history, ecology, and conservation. Notable additions include information from a recent checklist of bat ectoparasites for Honduras and basic acoustic descriptions of vocal signatures for some bat species. A total of 227 extant native mammalian species within 146 genera and 42 families, including three introduced rodent species, are verified to occur in Honduras. We anticipate that an additional 28 species could eventually be verified in Honduras, bringing the total to 255. A discussion of the importance of additional studies and the conservation of Honduran mammals is included. In closing, we discuss why some questionable records have persisted over time, with local and regional extinctions, and new threats. We hope this review will serve as a new baseline for the next generation to continue to study mammals systematically in the country.

## Introduction

Documenting which species occur and in what numbers is important for field studies and conservation assessments. We reviewed all available sources to establish the current number of verified mammal species in Honduras. While compiling information on Honduran mammals was perceived as straightforward, it proved more complex than initially imagined. Many historical records were based on documents that were no longer accessible or existed only in limited printed or handwritten forms. Additionally, records that had served as vouchers were often based on outdated taxonomy, leading to confusion about which species they represented. Many such historical specimen vouchers are scattered across the globe, including in private collections, with restricted or no access for review or verification of identifications. A large segment of the literature focused on charismatic species, such as jaguars, tapirs, and peccaries, with limited documentation of small- and medium-sized mammals or of their conservation. Notably, marine mammals have received limited attention; many are known only from stranding events. Despite these challenges, we have compiled a thorough review of verifiable records, resulting in the most up-to-date compendium of mammals confirmed to occur in Honduras. Although all authors contributed broadly, each specializes in particular taxa or groups of taxa and has carefully reviewed the groups with which they are most familiar. See author contributions for details.

The most recent list of mammals was included in a 2018 compendium of biodiversity (McCranie et al. 2018), which listed 157 species. Since then, there have been significant updates, taxonomic changes, and clarifications that have impacted the understanding of species limits and resulting distributions. For example, *Spilogale putorius* (Linnaeus, 1758), which was included on that prior list, is now known not to occur in the country, and several rodent species were omitted entirely. With advancements in taxonomic and field survey methods (e.g., molecular analysis, camera traps, harp traps, acoustic monitoring) and increased survey efforts, many additional species have now been confirmed to occur here. For example, *Myrmecophaga tridactyla* Linnaeus, 1758 was confirmed in 2001 (McCain 2001). Several species of bats have also recently been verified, e.g., *Hylonycteris underwoodi* Thomas, 1903 in 2019 (Turcios-Casco and Medina-Fitoria 2019); *Diaemus youngii* (Jentink, 1893) in 2020 (Turcios-Casco et al. 2020a), *Centronycteris centralis* Thomas, 1912 (Espinal et al. 2021) in 2021, and *Cormura brevirostris* (Wagner, 1843) in 2025 (Turcios-Casco et al. 2025a), and the ranges of others have been greatly expanded. Recent taxonomic studies have redefined occurrence, redefining species limits and nomenclature. As an example, *Metachirus myosuros* (Temminck, 1824) was previously recognized as *Metachirus nudicaudatus* (É. Geoffroy Saint-Hilaire, 1803) by Mérida and Cruz (2015). For further insights on recent advances in Honduran mammalogy, see Turcios-Casco (2026) and a discussion of mammal specimens and collections from the country by Turcios-Casco and López (2025).

Our goal is not to create a comprehensive bibliography of all literature related to Honduran mammals, but to consolidate essential information about the species that have been verified as occurring. This includes updated species counts and ecological and distributional information for each species. We aimed to gather all available data on Honduran mammals and provide notes on taxonomy, morphology, and ecology where applicable. Consequently, the species accounts only include relevant citations and are not intended to provide all published sources, nor do they represent the only works related to a given species.

As acoustic identification of bats is increasingly important for understanding species distributions, we include values within the 90% range of the characteristic frequency of the dominant harmonic (Fc) of the vocal signature. Information on mammalian ectoparasites is scarce; therefore, occurrences on rodents and bats are included. Species accounts include conservation threats and report levels of concern at both global and national levels, which may differ. This compilation includes new and previously unpublished records from the 1960s, historical monitoring by one of us (RKL), and the first acoustic records of bats (BWM).

### Materials and methods

Verified records are based on published sources, books, journals, a wide selection of “gray literature” including government and non-government reports, theses, technical reports, protected area management plans, and documents from government agencies. We also reviewed virtual collection records included in the Global Biodiversity Information Facility (GBIF) (www.GBIF.com), VertNet (www.vertnet.org), and observations reported in iNaturalist (www.inaturalist.org). Previously unpublished records from the 1990s to the present include NGO-led surveys, the authors’ fieldwork, and occasional sightings, such as carcasses.

Each account includes the species name, relevant taxonomic notes, location, global and national conservation status. When location data were sufficiently detailed, we verified the coordinates and confirmed the distribution; for those lacking sufficient location detail, we derived approximate coordinates. When multiple species records were reported within a 10-km radius, we consolidated them into a single derived point to improve distribution estimates.

Records lacking verification of species identification or specific geographical location information (e.g., unverified iNaturalist observational records) were excluded from our compilation. As this compilation concerns extant species, the fossil record was not included.

### Study area

**Figure 1.**
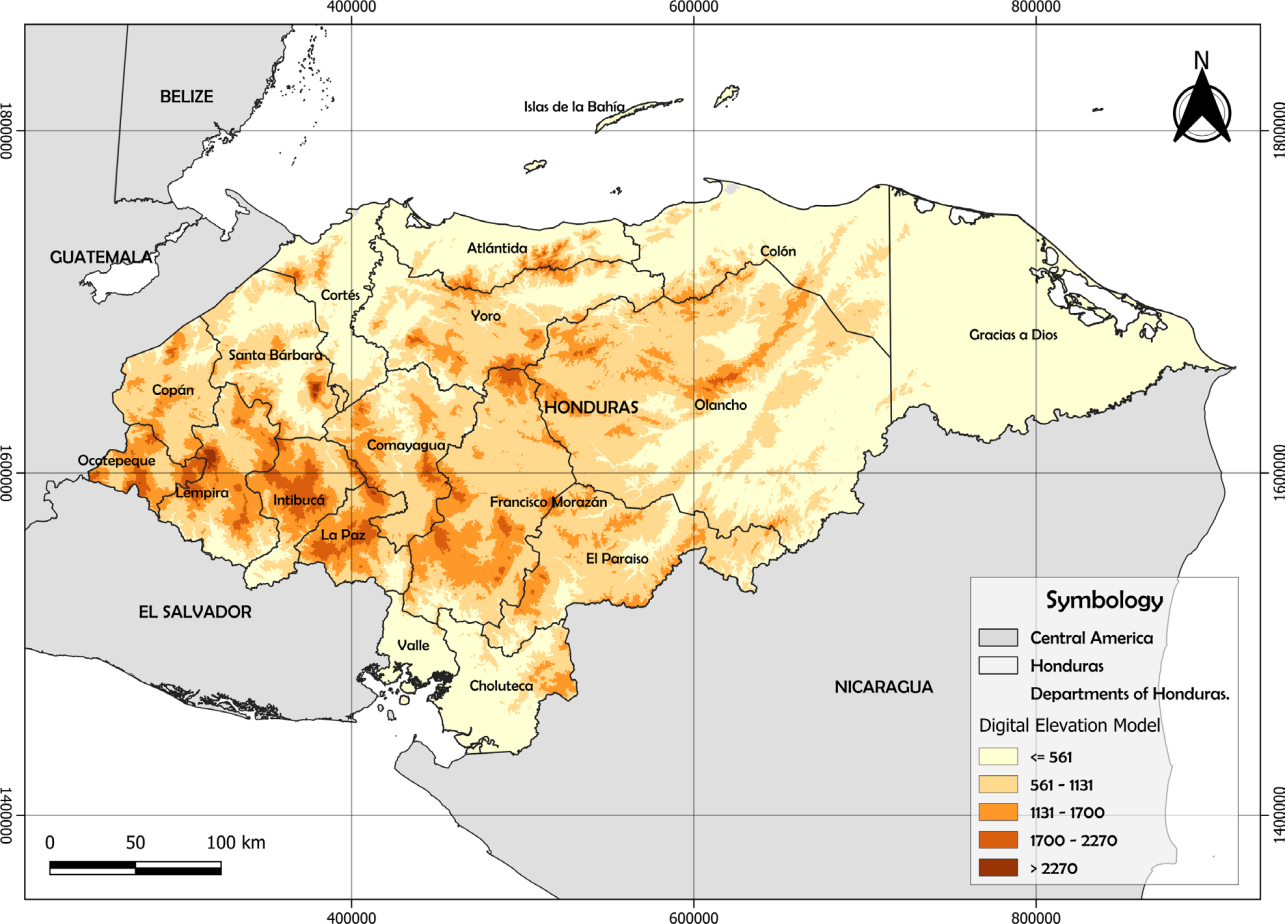
Elevation map of Honduras derived from a Digital Elevation Model (DEM). Elevation is represented in five altitudinal classes (in meters above sea level), with darker colors indicating higher elevations. Major topographic features are concentrated in western, central, and southern Honduras, while lowland areas dominate the northern Caribbean coastal plain and the eastern region of Gracias a Dios (La Moskitia). Departmental boundaries are indicated, and surrounding countries (Guatemala, El Salvador, Nicaragua, and Belize) are shown for geographic reference.

This review covers the terrestrial and coastal area of Honduras, which spans 112,492 km^2^ (Fig. 1), although unofficial estimates suggest it could be 112,777 km^2^ (including cays, islands, and islets not part of the continental area) bordering the Caribbean Sea to the north, Nicaragua to the east and southeast, the Gulf of Fonseca and El Salvador to the south, and Guatemala to the west (McCranie and Castañeda 2007). The diverse terrain has been shaped by past geologic movements and volcanic activity (Mejía-Ordoñez and House 2002). The topography ranges from sea level to mountain peaks, with Cerro Las Minas in Celaque the highest at ~ 2870 m above sea level.

### Camera trapping

Beginning in the early 2000s, camera traps were deployed across the country. Units were set near animal tracks, feces, suspected animal corridors, and areas with livestock to determine which species might pose wildlife/human conflicts. Placement was on tree trunks, branches, riverbanks, and creeks. Camera stations were typically set 30–50 cm above ground to detect medium to large-sized mammals. Most camera stations were spaced 1–5 km apart, with a mix of single and two-camera pairs per station. From 2007 to 2026, ~ 90,000 camera-trap nights were accumulated from across the country. The areas with the highest survey effort were the Río Plátano Biosphere Reserve (~ 15,000 camera nights), Pico Bonito, Jeannette Kawas, and Cusuco National Parks (~ 13,000, 16,000, and 11,000 camera nights, respectively). Annual surveys have been conducted in these areas since 2012. Camera settings varied by unit type, and most were thermo-motion-triggered. While most were still photographs, some cameras were programmed to record 10-second videos every 10–20 minutes.

### Mist nets

Bat distributions include previously unpublished mist-net capture records from RKL, based on fieldwork conducted in the 1960s and subsequent surveys by others. Nets used were generally 12 × 2.5 m and 9 × 2.5 m with a 30–35 mm mesh. Netting locations included open fields, closed-canopy forests, urban and rural areas, villages, river basins, seasonal ponds, pastures, coffee and cacao plantations, other crops, and cave entrances. Recent netting followed the placement criteria of Kunz and Kurta (1988).

### Acoustic monitoring

Many of the new bat distribution records presented here are based on confirmed vocal signatures from acoustic surveys. Acoustic monitoring has become a primary, cost-effective, non-invasive sampling method for free-flying bats (e.g., O’Farrell and Gannon 1999; Miller 2001). Acoustics detect species that frequently avoid traditional capture methods and habitats not readily sampled with mist nets or harp traps by recording calls of individuals flying high above the ground, over open areas, and in water bodies (O’Farrell and Miller 1997; Ochoa et al. 2000; Miller and Miller 2025). Species of the family Phyllostomidae generally rely on sight and not echolocation while flying, and like birds, often do not detect nets and are more readily captured. Members of the Phyllostomidae are known as “whispering bats” (Griffin 1986), as their echolocation calls are generally quieter than those of other families. These species are sometimes recorded as free-flying bats. However, nearly all reference calls of phyllostomids have been recorded from hand-released bats at very close range. Such calls are multiharmonic, decreasing frequency, broadband FM pulses. Species calls exhibit considerable overlap in shape and call parameters and are not yet practical for identifying bats in the field.

However, data from acoustic monitoring of free-flying bats can reliably identify most species in five of the eight families occurring in the country: Emballonuridae Gervais, 1855, Molossidae Gervais, 1856, Mormoopidae Saussure, 1860, Noctilionidae Gray, 1821, and Vespertilionidae Gray, 1821. Many high-flying species, such as molossids, are difficult to capture and have not yet been acoustically verified. Matching faces to voices is critical for verifying species’ vocal signatures, as these serve as acoustic voucher records used to confirm acoustic identifications. Many other species from the families noted above detect nets via echolocation, generally avoiding capture, and remain acoustically unverified.

Most species in the families Natalidae Gray, 1866 and Thyropteridae Miller, 1907 have not yet been acoustically verified. The few species recorded produce high-frequency broadband calls that are not yet reliable for identifying free-flying species. Species identifications were confirmed by comparing recordings of search-phase vocal signature sequences to published accounts (e.g., Miller et al. 2024), and primarily by comparison of verified sequences in a master call (>2 million sequences) reference library (Miller et al. 2024). The majority of files were manually vetted by one of us (BWM).

To our knowledge, one of us (BWM) conducted the first acoustic bat surveys in Honduras at Capiro y Calentura N.P. Trujillo, Colón in October 1999 (Miller and Miller 2000), followed in November 2005 by four nights of passive monitoring over an open parking area from the second floor of the Hotel Palma Real - Sambo Creek, Atlántida, in conjunction with the first acoustic workshop taught in Honduras. This was followed by multiple nights of opportunistic passive recording on the campus of the agricultural college, Zamorano.

More recent acoustic surveys (e.g., Espinal and Mora 2012) in Honduras were reviewed, and mistaken identifications were corrected or discounted during verification of species distributions. For example, the *Cynomops mexicanus* (Jones and Genoways, 1967) (Molossidae) record reported by Espinal and Mora (2016) was based on an early 1996 misidentified acoustic recording from Belize, which has now been verified as *Promops centralis* Thomas, 1915. The record of *Aeorestes cinereus* (Palisot de Beauvios, 1796) included in that publication as *Lasiurus cinereus* (Palisot de Beauvois, 1796), was also found to be invalid. Considering the abovementioned, the records of Espinal and Mora (2012) were not considered for the other species.

Just as physical specimens of captured and released, or voucher mammalian specimens, are measured to provide a standard set of comparative and diagnostic values, acoustic voucher records also include diagnostic details. The characteristic frequency of the dominant harmonic (Fc) of search-phase calls is generally one of the most robust parameters for species identification. As this is not intended as a guide to species acoustic identification, we include values only for species accounts in which 90% of the Fc of the dominant harmonic has been acoustically verified. For a complete review of vocal signatures, we refer readers to the acoustic species accounts and interactive identification keys for bats known to occur in Honduras (Miller et al. 2024).

Bat distributions include recent unpublished records from capture and acoustic surveys conducted throughout the country by members of The Big Bat Theory, a project within the NGO Asociación para la Sostenibilidad e Investigación Científica en Honduras (ASICH).

### Occasional and other observations

In addition to collecting project-specific data during fieldwork, opportunistic observations of mammals were also noted. Such records were supported by photographs, video clips, fecal samples from felines, and roadkill carcasses for species identifications and GPS coordinates for locations.

### Live trapping

We mainly followed Matson et al. (2016) in using Sherman and Tomahawk collapsible live traps and one-liter plastic containers as pitfall traps. Live traps were checked in the morning and evening using standard baits (e.g., oats, peanut butter, raisins, vanilla, tuna, or sardines).

### Museum abbreviations

**CNMH** Carnegie Museum of Natural History

**FMNH** Field Museum of Natural History

**MCZ** Harvard University, Museum of Comparative Zoology

**MSB** Museum of Southwestern Biology

**MVZ** Museum of Vertebrate Zoology University of California Berkeley

**NHMUK (ZD)** Natural History Museum, London

**NHMO-DMA** University of Oslo, Mammal Collection, Oslo

**NML-VZ** World Museum, National Museums Liverpool-Vertebrate Zoology

**USNM** Smithsonian Institution, National Museum of Natural History

**UNAH** Universidad Nacional Autónoma de Honduras.

### Ethical statements

While we have no way of knowing how historical specimens were captured or how animals were handled, all recently captured animals were handled in accordance with the guidelines of Sikes et al. (2016).

### Distribution

Species accounts include the occurrence in one or more of the 18 departments: Atlántida, Choluteca, Colón, Comayagua, Copán, Cortés, El Paraíso, Francisco Morazán, Gracias a Dios, Intibucá, Islas de la Bahía, La Paz, Lempira, Ocotepeque, Olancho, Santa Bárbara, Valle, and Yoro. When a species record represents a new, previously unreported, occurrence, the department name is bolded. Previous records were classified as historical (before 2000) and recent (published since 2000), considering only published and verified occurrences. In general, more than 300,000 records were screened and verified. Elevation ranges are provided as meters above sea level (m a.s.l.). Distributions include verified identification of historical records from the GBIF.org (https://www.gbif.org/, accessed during 2023), published records, and unpublished records from the authors. Map coordinates are based on UTM zone 16N grids, using the WGS84 base datum. The source of information is https://geoportal.icf.gob.hn and https://sinit.hn.

### Ectoparasites

The only group of mammals for which we found a large number of published records of ectoparasites in Honduras was for bats (Dick 2013). We also include more recent bat host-parasite associations subsequent to those reported in Dick (2013). In a few cases, ectoparasites have been recorded on rodents and are included accordingly. See Table 1 for a checklist of mammalian ectoparasites for Honduras.

**Table 1.** Checklist of ectoparasitic arthropods of bats recorded in Honduras and their distribution in 11 departments: Atlántida, Comayagua, Copán, Cortés, Francisco Morazán, Gracias a Dios, Islas de la Bahía, Lempira, Olancho, Santa Bárbara, and Valle. A total of 56 species were documented, including 50 species from 18 genera of bat flies of the family Streblidae, three species of *Basilia* (bat flies of the family Nycteriidae), and two species of *Periglischrus* (wing mites of the family Spinturnicidae). Species presence in each department is indicated by an “X”. The records show an uneven geographic distribution, with some species occurring in several departments (e.g., *Trichobius parasiticus* and *Strebla wiedemanni*), while others are restricted to single localities based on current data. This checklist summarizes the known distribution of streblid flies associated with bats in Honduras and reflects regional differences in sampling effort and species occurrence.

Species	Atlántida	Comayagua	Copán	Cortés	Francisco Morazán	Gracias a Dios	Islas de la Bahía	Lempira	Olancho	Santa Bárbara	Valle
*Anastrebla modestini*					X						
*Anatrichobius scorzai*					X				X		
*Aspidoptera delatorrei*		X							X		
*Aspidoptera falcata*	X	X			X			X	X		X
*Aspidoptera phyllostomatis*	X				X						
*Basilia dubiaquercus*									X		
*Basilia ortizi*						X					
*Basilia rondanii*						X					
*Exastinion clovisi*		X			X						
*Mastoptera guimaraesi*	X										
*Mastoptera minuta*	X										
*Mastoptera* sp. (*minuta* complex)	X										
*Megistopoda aranea*	X						X	X	X		
*Megistopoda proxima*	X	X			X			X	X		X
*Metelasmus pseudopterus*	X									X	
*Megistopoda* sp. (undescribed species)					X						
*Neotrichobius bisetosus*						X					
*Neotrichobius stenopterus*	X										
*Noctiliostrebla traubi*									X		
*Nycterophilia coxata*		X	X						X		
*Paradyschiria fusca*									X		
*Paradyschiria parvuloides*									X		
*Paraeuctenodes longipes*		X							X		
*Paratrichobius dunni*	X										
*Paratrichobius longicrus*	X	X						X			X
*Paratrichobius* sp. (*salvini* complex)	X										
*Periglischrus iheringi*	X				X						
*Periglischrus ojastii*		X			X						
*Speiseria ambigua*	X										
*Speiseria peytonae*								X			
*Strebla chrotopteri*								X			
*Strebla curvata*	X	X							X		
*Strebla diphyllae*	X										
*Strebla galindoi*	X										
*Strebla guajiro*	X						X	X	X		
*Strebla hertigi*					X						X
*Strebla kohlsi*	X										
*Strebla matsoni*										X	
*Strebla wiedemanni*	X			X	X			X	X		X
*Trichobioides perspicillatus*	X		X	X	X						X
*Trichobius angulatus*							X				
*Trichobius caecus*	X		X					X	X		
*Trichobius costalimai*	X		X	X	X						X
*Trichobius diphyllae*	X										
*Trichobius dugesii*	X	X					X	X	X		X
*Trichobius galei*					X		X				X
*Trichobius hirsutulus*				X							
*Trichobius intermedius*		X						X			X
*Trichobius joblingi*	X	X		X				X	X		
*Trichobius longipes*	X			X							
*Trichobius parasiticus*	X	X	X	X	X			X	X		X
*Trichobius* sp. (complex dugesii)	X										
*Trichobius sparsus*	X										
*Trichobius uniformis*	X	X						X	X		X
*Trichobius yunkeri*		X									

### Natural history

All known studies on Honduran mammals were reviewed, and the natural history information was summarized for each species account. This included information on reproduction, predation, refuges, and related topics, with the aim of providing a comprehensive and consolidated summary for each species.

Due to the limited number of studies and field observations of marine mammals, we include natural history information compiled from regional accounts (e.g., the Caribbean or the Tropical Eastern Pacific) as a summary for species occurring in Honduran waters.

### Additional notes

This section includes other relevant information, such as local common names, specific threats, and related details. Conservation concerns include the global status provided by the IUCN Red List (https://www.iucnredlist.org/, accessioned during 2026) and Honduran assessments (WCS 2021), when available. The following two-letter codes: NE–Not Evaluated, DD–Data Deficient, LC–Least Concern, NT–Near Threatened, VU–Vulnerable, EN–Endangered, and CR–Critically Endangered are used. To avoid a lengthy list of individual citations for each species, all are cited as IUCN (2026) and WCS (2021).

### Taxonomy

Species distributions depend not only on understanding where species occur, but also on using universally accepted names for taxa to avoid confusion. We relied on the following for accepted names: Integrated Taxonomic Information System (ITIS) and the American Society of Mammalogists (ASM) Mammal Diversity Database (https://www.mammaldiversity.org/, accessed during 2026) for non-volant terrestrial mammals, Simmons and Cirranello compiled database for bats (https://www.batnames.org/, accessed during 2026), and the Society of Marine Mammalogy (SMM) List of Marine Mammal Species and Subspecies for the aquatic mammals (https://marinemammalscience.org/, accessed during 2026). The few exceptions to these are noted, with clarifications and justifications provided in the additional notes of the respective species account. Subspecies are generally noted only when multiple subspecies have been verified to occur here.

### Species accounts

Each taxon was reviewed and compiled by one or more of the specialist authors; see author contributions for details. The arrangement is based on the taxonomy, ranging from order to specific (scientific) name, following the Integrated Taxonomic Information System (ITIS; https://www.itis.gov/, accessed during 2026) and the American Society of Mammalogists (ASM) Mammal Diversity Database (https://www.mammaldiversity.org/, accessed during 2026) from the higher classifications to the species level, with a few exceptions. Depending on available information, natural history includes ecology, diet, reproduction, parasites, a basic description of bat vocalizations, and external or cranial measurements. Citations are provided for selected sources; otherwise, the data is that of the authors. For marine mammals, there is virtually no Honduran natural history; therefore, the specialists for this group (EAR and DGS) compiled information using local/regional data, which is cited accordingly.

Distributions are reported by the departments that have verified species, with elevation ranges when available. New distribution records are noted by bolding the department name. Distributions include historical records from the GBIF.org (https://www.gbif.org/, accessed during 2023), and unpublished records of the authors.

## Results and discussion

**Figure 2.**
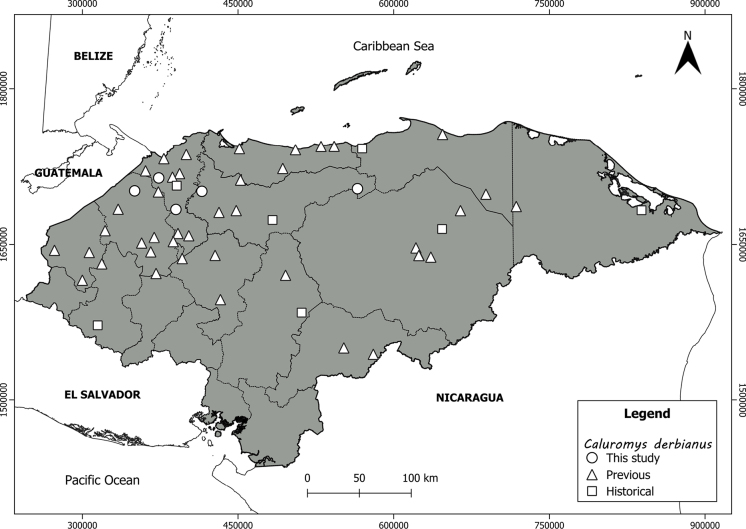
Distribution of *Caluromys derbianus* in Honduras.

**Figure 3.**
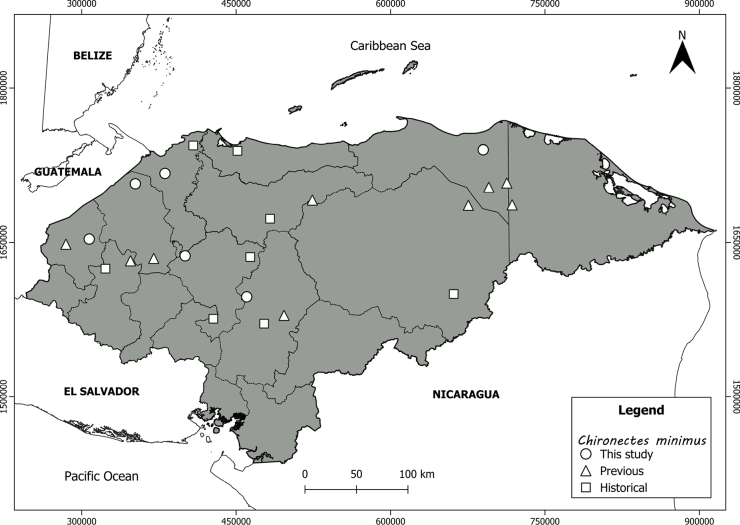
Distribution of *Chironectes minimus* in Honduras.

We confirmed a total of 42 families, 146 genera, and 227 extant mammalian species occurring in Honduras (Fig. 2), including three introduced rodent species: *Rattus norvegicus* (Berkenhout, 1769), *Rattus rattus* (Linnaeus, 1758), and *Mus musculus* (Linnaeus, 1758). Diversity of orders ranked by species is as follows: Chiroptera (115), followed by Rodentia (38 native + 3 introduced), Carnivora (20), Artiodactyla (19), Eulipotyphla (9), Didelphimorphia (7), Pilosa (5), Primates (3), Lagomorpha (2), Cingulata (2), Sirenia (1), and Perissodactyla (1) (Fig. 3). A summary of species of conservation concern based on the Honduran Red List (WCS 2021) indicates that 15.9% of species are threatened (3.5% Critically Endangered [CR], 2.6% Endangered [EN], and 9.7% Vulnerable [VU]). An additional 4.0% are Near Threatened (NT), and 24.2% are Data Deficient (DD). Globally, 7.5% of Honduran mammal species are considered threatened (0.4% CR, 1.3% EN, and 5.7% VU), 5.7% are NT, and 4.8% are DD.

### Species accounts

#### Didelphimorphia Gill, 1872

We recognize seven species, following Bautista et al. (2022). We do not include *Marmosa alstoni* J. A. Allen, 1900, as the only known specimen from Tate (1933) was a juvenile (Voss et al. 2021) and not unambiguously identifiable as *M. alstoni*. Additionally, Bautista et al. (2022) considered it extinct, suggesting it may be anticipated but has not been verified. However, Marineros-Sánchez et al. (2016) reported a putative individual of *M. alstoni*, and assuming both *M. alstoni* and *Marmosa nicaraguae* Thomas, 1905 may be present in Honduras, a thorough review of that specimen is warranted before confirming this species in the country. Additional undescribed species of the *M. alstoni* group seem likely to exist based on the evidence at hand, and they emphasized the need for renewed collecting (Voss et al. 2021).

#### Didelphidae Gray, 1821

##### *Caluromys* J. A. Allen, 1900

###### 1. Caluromys derbianus

Taxon classificationAnimaliaDidelphimorphiaDidelphidae

(Waterhouse, 1841)

01662CFD-6D1E-5DDB-95FA-A265F6543C64

####### Natural history.

Females have been captured with pouched young in March, May, and June. Marineros et al. (2016) documented predation by the Spectacled owl (*Pulsatrix perspicillata* (Latham, 1890)). We observed *C. derbianus* near mist nets while capturing bats. Marineros et al. (2016) reported that they occurred in rural areas, may use hollow trees from 3–5 m tall, and noted that a dead individual was found in Río Santiago, Atlántida department. Vocalizing females have been found in trees (Malvaceae Juss.) > 6 m tall. An individual with what appeared to be a severe dermal septic necrosis was observed in the north side of Pico Pijol, Yoro, in 2018.

####### Distribution.

Atlántida, Colón, Comayagua, Copán, Cortés, El Paraíso, Francisco Morazán, Gracias a Dios, Lempira, Olancho, Santa Bárbara, and Yoro (Fig. 2); 0–1500 m a.s.l.

####### Additional notes.

Occasional records include those from hunting (e.g., skins and anecdotal reports from local people who noted killing them for pest control) and from captive animals kept as pets (Marineros et al. 2016). They may be known as ratón guazalo, guazalillo dorado, or suk-suk among the Tawahka in eastern Honduras (see Marineros et al. 2016); locals also call them guazalo dorado. Global conservation status is LC and DD in Honduras.

##### *Chironectes* Illiger, 1811

###### 2. Chironectes minimus

Taxon classificationAnimaliaDidelphimorphiaDidelphidae

(Zimmermann, 1780)

0F07603F-65B7-5888-B794-4B951F475AB2

####### Natural history.

A pregnant female was captured in March with five pouched young. This species is nocturnal and semi-aquatic, typically active around ponds, rivers, and streams. It has been found in caves in western Honduras, suggesting some flexibility in roosting behavior. Food items based on feces include aquatic invertebrates, small fish, and amphibians. An individual with skin lesions resembling ulcers was observed swimming in a small stream near the village of Río Amarillo in Copán, western Honduras, in 2006.

####### Distribution.

Atlántida, Colón, Comayagua, Copán, Cortés, Francisco Morazán, Gracias a Dios, Lempira, Olancho, Santa Bárbara, and Yoro (Fig. 3); 100–1450 m a.s.l.

####### Additional notes.

They are known as li kiskika by the Miskitus in eastern Honduras (Jones 1965) and as perro de agua or guazalo de agua in Spanish. In some regions, local people have reported that they are no longer found in certain water bodies due to pollution, particularly from mining and agricultural runoff, which degrade aquatic habitats. Global conservation status is LC and DD in Honduras.

##### *Didelphis* Linnaeus, 1758

###### 3. Didelphis marsupialis

Taxon classificationAnimaliaDidelphimorphiaDidelphidae

Linnaeus, 1758

7BF55291-C117-52EF-A12C-9CFB96460EDB

####### Natural history.

Females with offspring have been found from January through March, and from September to December. Although primarily nocturnal, it has been observed to be active during the day, suggesting behavioral flexibility. They have been found in hollow logs. Individuals have been documented opportunistically preying on the bat *Carollia perspicillata* (Linnaeus, 1758) that were entangled in mist nets. It is an omnivore, feeding on fruits (mango, guajavas, papayas), insects, and small vertebrates (coral snakes). We have observed individuals climbing trees at > 25 meters above ground.

####### Distribution.

**Figure 4.**
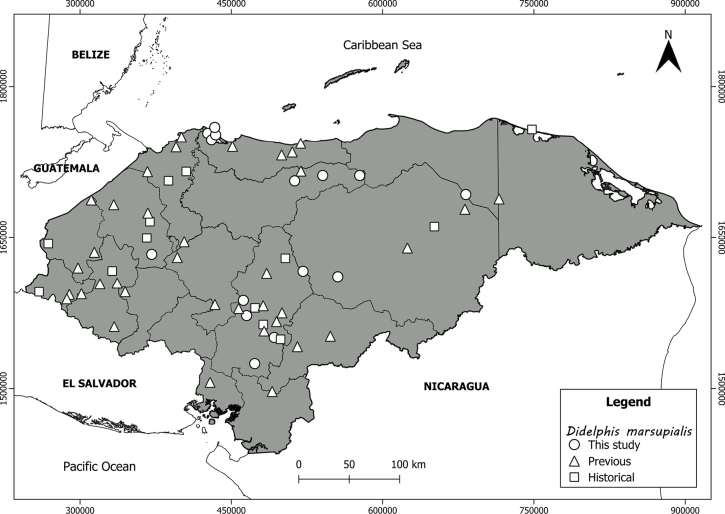
Distribution of *Didelphis marsupialis* in Honduras.

Atlántida, **Colón**, Comayagua, Copán, Cortés, El Paraíso, Francisco Morazán, Gracias a Dios, Intibucá, Lempira, Ocotepeque, Olancho, Santa Bárbara, Valle, and Yoro (Fig. 4); 0–2050 m a.s.l.

####### Additional notes.

They are known as kiski tara by the Miskitus in eastern Honduras (Jones 1965). It is commonly referred to in Spanish as zorro, zarigüeya, or comadreja. Localized threats include being killed as a perceived predator of poultry. This species is also a victim of roadkill on main roads throughout the country. Despite these pressures, the populations are resilient, and as a habitat generalist, it thrives in both natural and human-modified environments. Global conservation status is LC and NE in Honduras.

###### 4. Didelphis virginiana

Taxon classificationAnimaliaDidelphimorphiaDidelphidae

Kerr, 1792

19F4ECCD-EDB2-569D-9577-0030FB30AEF5

####### Natural history.

Females with offspring have been found between February and May, and August. This species is generally solitary and primarily nocturnal, although it may be active during the day in undisturbed areas. It is a semi-arboreal marsupial that inhabits a wide range of environments, from humid lowland forests to pine-oak woodlands and even urban areas. It is an opportunistic omnivore that feeds on a variety of fruits (e.g., *Ficus* L., *Cecropia* Loefl., *Psidium* L., *Musa* L.), insects (especially beetles, orthopterans, and cockroaches), small vertebrates such as rodents, frogs, and birds, and occasionally scavenges carrion and human food waste. Individuals have been observed feeding on mist-netted bats (*Artibeus inopinatus* Davis & Carter, 1964 and *C. perspicillata*), reflecting their opportunistic foraging habits. Shelters include tree hollows, rock crevices, or human structures such as roofs and abandoned buildings. The use of their tail as a fifth limb to securely hold items, such as dry leaves, has been recorded by camera traps at several locations in Honduras. Reports of this species are frequently misidentified *D. marsupialis*, which is considerably more common than this species. It appears well adapted to both urban and rural habitats across the country, as evidenced by the large number of roadkills.

####### Distribution.

**Figure 5.**
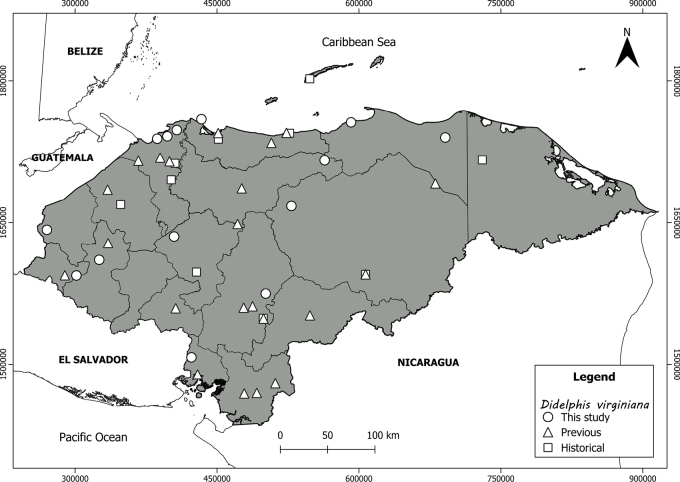
Distribution of *Didelphis virginiana* in Honduras.

It is known to occur in Atlántida, Choluteca, **Colón**, Comayagua, **Copán**, Cortés, El Paraíso, Francisco Morazán, Gracias a Dios, La Paz, Lempira, Ocotepeque, Olancho, Santa Bárbara, Valle, and Yoro (Fig. 5); 0–2680 m a.s.l. Apparently, it was inadvertently introduced to the Islas de la Bahía department via freight.

####### Additional notes.

This species is impacted by roadkill. They are known as kiski tara by the Miskitus in eastern Honduras (Jones 1965). Global conservation status is LC and NE in Honduras.

##### *Marmosa* Gray, 1821

###### 5. Marmosa mexicana

Taxon classificationAnimaliaDidelphimorphiaDidelphidae

Merriam, 1897

6996674D-BFEE-590A-9B98-7CFF74E17D3F

####### Natural history.

They are common in Islas de la Bahía, particularly in Roatán and Guanaja, where they have been observed occasionally entering houses (Marineros and Elvir 2019). Oats and peanut butter are commonly used as bait for trapping. It has been reported to occasionally interact with the endemic cactus *Stenocereus yunckeri* (Standl.). P. V. Heath (Turcios-Casco et al. 2025b).

####### Distribution.

**Figure 6.**
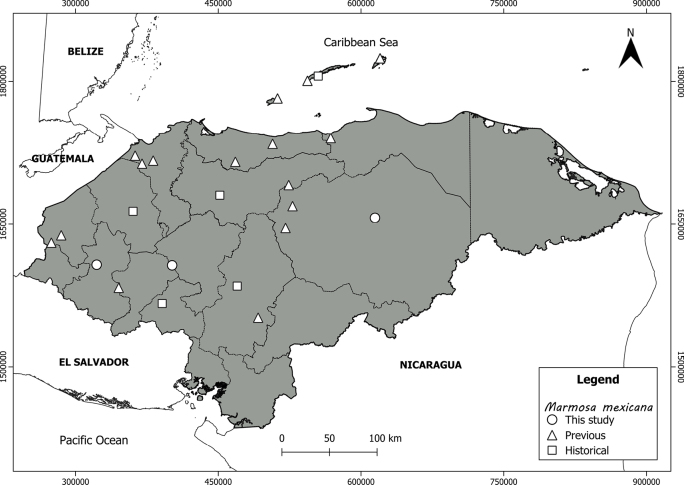
Distribution of *Marmosa mexicana* in Honduras.

Atlántida, Colón, **Comayagua**, Copán, Cortés, Francisco Morazán, Intibucá, Islas de la Bahía, La Paz, **Lempira**, Ocotepeque, Olancho, Santa Bárbara, and Yoro (Fig. 6); 0–2670 m a.s.l. It is likely that *M. mexicana* occurs in Gracias a Dios, based on camera-trap images that were clearly of a *Marmosa* spp. As it was not possible to identify it to species, we have not included these records here.

####### Additional notes.

Historical specimens have been referred to as *Marmosa ruatanica* Goldman, 1911, *Marmosa mexicana mexicana* Merriam, 1897, or *Marmosa robinsoni* Bangs, 1898. Marmosas are known as kiski (sikiski) sirpi by Miskitus in eastern Honduras (see Tate 1933; Voss et al. 2021; Bautista et al. 2022; Astúa et al. 2023). The species is expected to occur in Gracias a Dios; however, given its potential sympatry with other congeners, specimens should be carefully examined to confirm their identification. They are commonly known as marmosas, comadrejas or guazalillos dorados. Six specimens collected between 1932 and 1934, identified as *Marmosa murina* (Linnaeus, 1758), should be referred to *Marmosa alstoni* J. A. Allen, 1900, which is now considered extirpated. Bautista et al. (2022) included *Marmosa ruatanica* as a synonym of *M. mexicana*. A review of all *Marmosa* specimens is needed, and may result in the recognition of additional species in the country. Global conservation status is LC and NT in Honduras.

##### *Metachirus* Burmeister, 1854

###### 6. Metachirus myosuros

Taxon classificationAnimaliaDidelphimorphiaDidelphidae

(Temminck, 1824)

24E908D5-88BB-5EFB-9791-BD1D9531C170

####### Natural history.

The species was confirmed by Mérida and Cruz (2015) and Marineros et al. (2019).

####### Distribution.

**Figure 7.**
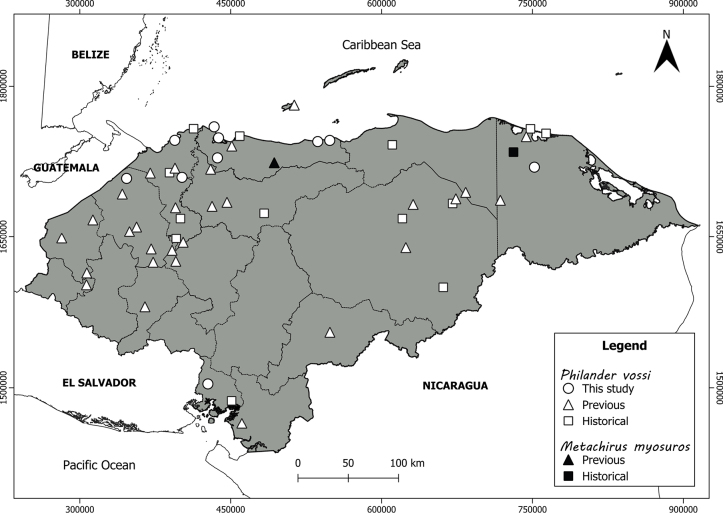
Distribution of *Metachirus myosuros* and *Philander vossi* in Honduras.

Atlántida and Gracias a Dios (Fig. 7); 0–145 m a.s.l. This species was recently reported from Sierra Caral, Guatemala, 6 km from the Honduran border (Trujillo and Escobar-Anleu 2023), suggesting that it likely occurs in the Cordillera El Merendón in northwestern Honduras.

####### Additional notes.

Previous records referred to this species as *Metachirus nudicaudatus* (É. Geoffroy Saint-Hilaire, 1803). The occurrence of this species is supported by Marsh et al. (2022), Voss and Jansa (2021), Bautista et al. (2022), and Astúa et al. (2023). Global conservation status is LC and DD in Honduras; both assessments refer to *M. nudicaudatus*.

##### *Philander* Brisson, 1762

###### 7. Philander vossi

Taxon classificationAnimaliaDidelphimorphiaDidelphidae

A. L. Gardner & Ramírez-Pulido, 2002

9D06F472-3BE9-551E-AE3B-E87A1602C843

####### Natural history.

We observed this species while netting bats. Most of the available natural history information (e.g., Marineros et al. 2015) cannot be attributed to this species, as the occurrence of *Philander melanurus* (Thomas, 1899) is still debated for the country (Bautista et al. 2022, but see also Gardner and Ramírez-Pulido 2020; Voss and Jansa 2021; Voss et al. 2021; Astúa et al. 2023). However, throughout its distribution in Honduras, *Philandersensu lato* is a nocturnal, semi-terrestrial marsupial that likely inhabits humid lowland forests, forest edges, and secondary growth near rivers and streams. Shelter is likely taken in tree hollows, dense undergrowth, or abandoned burrows during the day. A broad diet that includes fruits (*Ficus*, *Cecropia*, *Psidium*), insects (especially beetles and orthopterans), small vertebrates such as frogs and lizards, and occasionally bird eggs. It forages mainly on the ground but may climb low vegetation in search of food. Individuals have been observed near mist nets during bat sampling activities, suggesting opportunistic behavior toward potential prey or food scraps.

####### Distribution.

Atlántida, Choluteca, Colón, Comayagua, Copán, Cortés, El Paraíso, Gracias a Dios, Intibucá, Islas de la Bahía, Olancho, Santa Bárbara, Valle, and Yoro (Fig. 7); 0–1750 m a.s.l.

####### Additional notes.

Historical specimens are referred to by several names, including *Philander laniger* Trouessart, 1898; *Philander opossum* (Linnaeus, 1758); and *Philander pallidus* J. A. Allen, 1901. They are known as kiski (sikiski) tilams by the Miskitus in eastern Honduras (Jones 1965). Other common names include guazalo negro, guazalillo de cuatro ojos, guazalo cuatro ojos, or comadreja; the misuse of the common name Cayopolín was clarified by Marineros et al. (2015). Threats include road kills in northern and western Honduras, but they are not taken by native people. The occurrence in Islas de la Bahía needs study to determine if it is naturally occurring or has been introduced, as was the case for the genus *Didelphis*. LC global refiriéndose a Philander opossum.

#### Cingulata Illiger, 1811

We currently accept the proposal of the genus *Dasypus* Linnaeus, 1758, and the species that occurs in Honduras as *Dasypus mexicanus* Peters, 1864, following Barthe et al. (2025).

#### Chlamyphoridae Bonaparte, 1850

##### *Cabassous* McMutrie, 1831

###### 8. Cabassous centralis

Taxon classificationAnimaliaCingulataChlamyphoridae

(Miller, 1899)

F4C56D0F-BEA5-501A-9A5A-6BDF01ABD726

####### Natural history.

They are mainly nocturnal. Their habitat ranges from core zones of protected areas to disturbed areas. One individual was observed burrowing while trying to escape from humans near the city of Tela.

####### Distribution.

**Figure 8.**
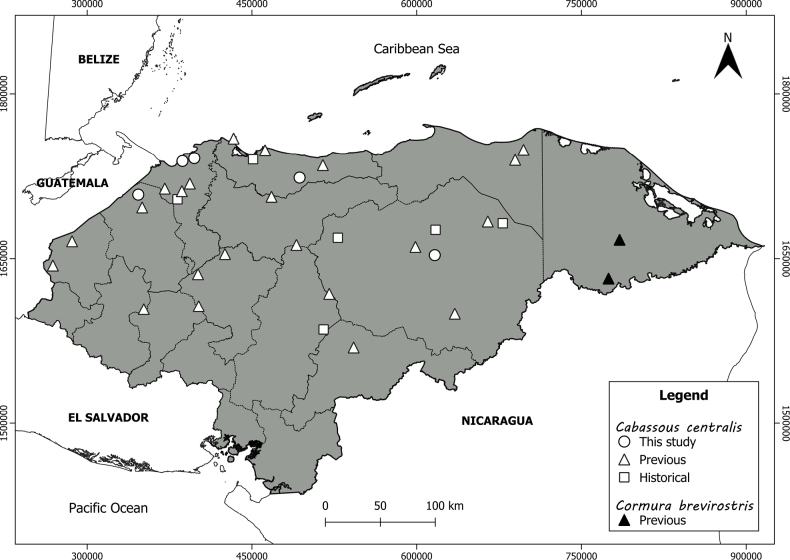
Distribution of *Cabassous centralis* and *Cormura brevirostris* in Honduras.

Atlántida, Colón, Comayagua, Copán, Cortés, El Paraíso, Francisco Morazán, Intibucá, Olancho, Santa Bárbara, and Yoro (Fig. 8); 10–2100 m a.s.l.

####### Additional notes.

*C. centralis* is known as takan by indigenous Miskitus who believe it secretes what they call leche in Spanish, and that it is poisonous. Additionally, the species is part of the Honduran folklore, see Turcios-Casco et al. (2020b). It appears this species may be widely distributed across the country and not be as rare as previously reported (Turcios-Casco et al. 2020b, 2023a). Global conservation status is LC and DD in Honduras.

#### Dasypodidae Gray, 1821

##### *Dasypus* Linnaeus, 1758

###### 9. Dasypus mexicanus

Taxon classificationAnimaliaCingulataDasypodidae

Peters, 1864

6BB9EA3F-1B63-5FF6-9AF9-42BF0C5ABE64

####### Natural history.

Offspring have been found from February to March and from July to October. *D. mexicanus* is a primarily nocturnal and solitary armadillo occurring over a wide range of habitats, including dry forests, pine-oak forests, tropical rainforests, and agricultural landscapes. It forages mostly on the ground, using its strong claws to dig for invertebrates, particularly ants, termites, beetle larvae, and earthworms, but it also consumes fruits, small vertebrates, and carrion on occasion. Natural predators include large carnivores such as coyotes (*Canis latrans* Say, 1823), pumas (*Puma concolor* (Linnaeus, 1771)), and jaguars (*Panthera onca* (Linnaeus, 1758)), while juveniles may occasionally be preyed upon by tayras (*Eira barbara* (Linnaeus, 1758)) and small felids (*Leopardus* Gray, 1842).

####### Distribution.

**Figure 9.**
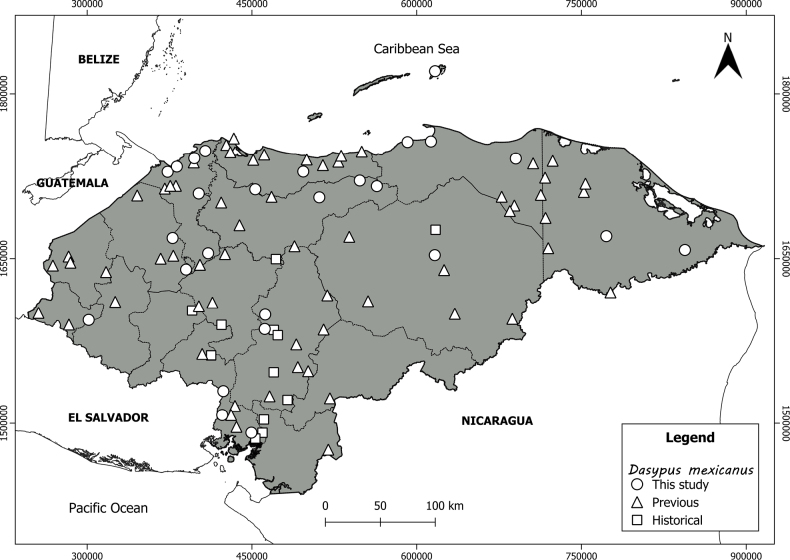
Distribution of *Dasypus mexicanus* in Honduras.

They have been found in all departments (Fig. 9); 0–2150 m a.s.l.

####### Additional notes.

They are known as taiira (tayra) by the Miskitus in eastern Honduras (Jones 1965). Not to be confused with the mustelid Tayra. They are the most widely distributed xenarthrans in Honduras (Turcios-Casco et al. 2020b) and among the most commonly taken animals by subsistence hunters. The carapace is often displayed as a trophy in houses and is considered to bring good luck. Camera-trap data indicate that this species occurs on the island of Guanaja, Islas de la Bahía, and, based on reports from local residents, was introduced for hunting. Locally referred to as Cusucos, they are prized food items in some communities. In some cases, they are used as bait to hunt larger mammals. Global conservation status is LC and NT in Honduras; the latter refers to *D. novemcinctus*.

#### Pilosa Flower, 1883

**Bradypodidae Gray, 1821**

##### *Bradypus* Linnaeus, 1758

###### 10. Bradypus variegatus

Taxon classificationAnimaliaPilosaBradypodidae

Schinz, 1825

46ABBAD0-7DFA-59D1-851C-97DA303D0428

####### Natural history.

Its diet consists mainly of leaves, shoots, and buds from a limited number of tree species, and it plays a role in canopy ecology as both a folivore and a host to diverse commensal organisms, including algae, moths, and mites. The species may occur in the tropical forests of Cortés and Yoro and has been found in subtropical wet forests and tropical moist forests, including mangroves (Marineros and Portillo Reyes 2015). There is a record of *B. variegatus* from Francisco Morazán (https://www.inaturalist.org/observations/98646697); however, this may represent an individual kept as a wildlife pet.

####### Distribution.

**Figure 10.**
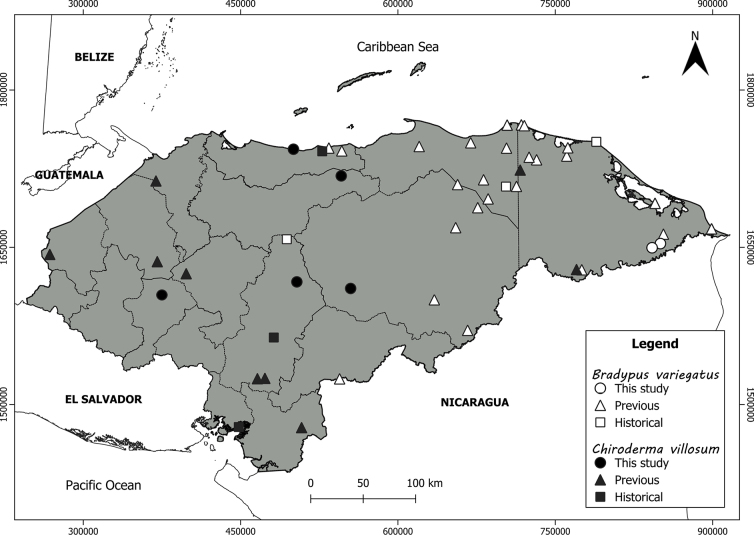
Distribution of *Bradypus variegatus* and *Chiroderma villosum* in Honduras.

Atlántida, Colón, El Paraíso, Francisco Morazán, Gracias a Dios, and Olancho (Fig. 10); 0–700 m a.s.l.

####### Additional notes.

The main threats to *B. variegatus* in Honduras are deforestation, habitat fragmentation, and illegal capture for the pet trade (prices in Honduras vary from USD $100 to $400 and can increase up to threefold when the animals are sold to foreigners). This is the most trafficked mammal in northern Honduras, often sold illegally at tourist centers, particularly in Islas de la Bahía and other Caribbean destinations (Turcios-Casco et al. 2020b). Young individuals are sometimes captured and kept as pets, resulting in high mortality due to improper care and dietary deficiencies. Additionally, sloths are vulnerable to attack by domestic dogs when forced to move on the ground. Global conservation status is LC and EN in Honduras.

#### Megalonychidae Ameghino, 1889

##### *Choloepus* Illiger, 1811

###### 11. Choloepus hoffmanni

Taxon classificationAnimaliaPilosaMegalonychidae

Peters, 1858

998830AA-87D4-5E8E-810A-4CD6A3C0C225

####### Natural history.

The first records were by Gamero (1978) and McCarthy et al. (1999). Turcios-Casco et al. (2020b), based on Acosta (2015), noted that **>** 50% of records of this species were attributable to illegal trafficking. Preliminary camera-trap studies have shown that activity consists of sleeping (61.3%), feeding (19.4%), preening (16.1%), and defecating (3.2%) (Martínez et al. 2020). Although *C. hoffmanni* is cryptic and difficult to detect due to its arboreal habits, local observations confirm its presence in mature forest canopies and occasionally in secondary forest patches or riparian corridors. Its diet consists primarily of leaves, fruits, and shoots from a wide range of tree species (*Inga oerstediana* Willd. and *Cecropia peltata* L.), and it plays an important ecological role in nutrient cycling through its defecation behavior and potential seed dispersal.

####### Distribution.

**Figure 11.**
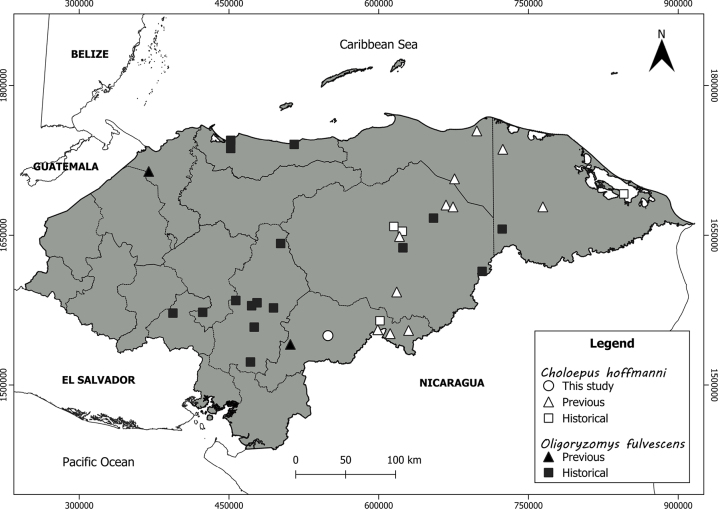
Distribution of *Choloepus hoffmanni* and *Oligoryzomys fulvescens* in Honduras.

Colón, El Paraíso, Gracias a Dios, and Olancho (Fig. 11); 0–1000 m a.s.l. It likely occurs in Yoro as well.

####### Additional notes.

We have documented that, in southern La Moskitia, it is occasionally hunted for its meat. It is also taken and sold at tourist centers and in other Caribbean countries, especially Jamaica and Cayman Islands, posing a key threat to this species. Prices in Honduras vary from USD$100 to $400 and can increase up to threefold when the animals are sold to foreigners. Global conservation status is LC and EN in Honduras.

#### Cyclopedidae Pocock, 1924

##### *Cyclopes* Gray, 1821

###### 12. Cyclopes dorsalis

Taxon classificationAnimaliaPilosaCyclopedidae

(Gray, 1865)

55F86AB9-F4DB-5ECB-BFAF-AFCDAE0F62CF

####### Natural history.

*Cyclopes dorsalis* is known from only 18 localities since 1891 (Turcios-Casco et al. 2020b, but see Portillo Reyes et al. 2021). McCain (2001) documented a mother with her half-grown offspring in the Río Plátano Biosphere Reserve (RPBR) during 1995–1996. It is the smallest arboreal xenarthran in the country. It is a strictly nocturnal, solitary species, highly specialized to feed on ants and termites, which it extracts using its long, sticky tongue. In Honduras, the species inhabits tropical moist and subtropical wet forests, usually in well-preserved primary or mature secondary forests. Records from the RPBR (McCain 2001) include observations of a mother with a half-grown juvenile, suggesting a prolonged maternal care typical of the genus. Camera-trap evidence indicates that the tayra may function as a natural predator in northern populations.

####### Distribution.

**Figure 12.**
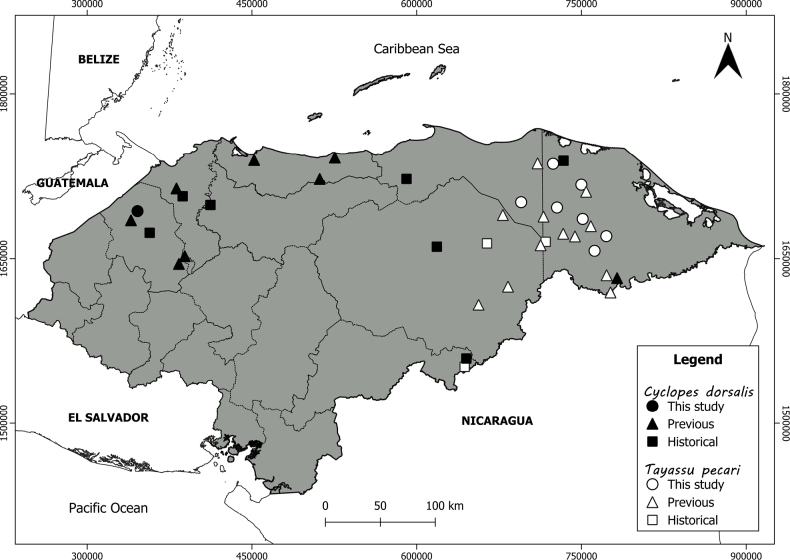
Distribution of *Cyclopes dorsalis* and *Tayassu pecari* in Honduras.

Atlántida, Colón, Cortés, El Paraíso, Gracias a Dios, Olancho, Santa Bárbara, and Yoro (Fig. 12); 50–1,430 m a.s.l. Based on the known distribution, they may also occur in northern Comayagua.

####### Additional notes.

We recognize the name of *C. dorsalis* based on Miranda et al. (2017). Historical records refer to *Cyclopes didactylus* (Linnaeus, 1758). Common names include serafín o perezocito and they are known as likur by the Miskitus in eastern Honduras (Jones 1965). In Honduras, *C. dorsalis* faces multiple threats, primarily habitat loss and fragmentation resulting from deforestation, agricultural expansion, and logging—particularly in the lowland and premontane forests of the Caribbean slope. It is strictly arboreal and nocturnal habits make it highly sensitive to canopy disruption and forest isolation. Although not directly targeted by hunters, individuals are occasionally captured or killed upon encounter, likely due to unfamiliarity with the species. Surveys specifically targeting this species are limited, and most records have been gathered opportunistically. Strengthening long-term camera-trap and arboreal-survey programs, especially in eastern and northern Honduras, is essential to better understand population status, ecological requirements, and conservation needs. Global conservation status is LC and VU in Honduras.

#### Myrmecophagidae Gray, 1825

##### *Myrmecophaga* Linnaeus, 1758

###### 13. Myrmecophaga tridactyla

Taxon classificationAnimaliaPilosaMyrmecophagidae

Linnaeus, 1758

7DF57CC8-A0AD-51A2-B51A-F5BF5D6809BA

####### Natural history.

Goodwin (1942a) included this species as occurring here, but it was not verified, and McCain (2001) presented photographic evidence, confirming the species for the country. The first camera-trap record of *M. tridactyla* was obtained in 2008 near Brus Laguna in La Moskitia (Gonthier and Castañeda 2013). Mérida Colindres and Cruz (2014) confirmed it for Honduras based on skull morphology. In January 2022, the first offspring of *M. tridactyla* were reported in northern Central America, in Wuarska, Colón Department (Martínez et al. 2022). Recently, another offspring was recorded for southern Moskitia in March 2025. A carcass of this species was found in the Rus Rus river gallery forest in southcentral Moskitia during 2001. The location was characterized by a narrow strip of broad-leaf forest surrounded by extensive pine savannas. Indigenous hunters in La Moskitia do not intentionally persecute this species, but it may be shot on sight as they are considered a threat to their dogs. Extensive camera-trap surveys conducted from 2012 to 2026 in Pico Bonito, Cusuco, and Jeannette Kawas National Parks have not yielded any records of this species. Field observations indicate that individuals frequently use seasonal ponds and temporary water bodies for bathing, particularly during periods of elevated temperatures. These aquatic sites may play an important role in thermoregulation and parasite control, although their ecological significance has yet to be studied in detail. WCS Honduras is developing a project to identify and study the northernmost population of this species in the country, representing the first initiative of its kind in Central America.

####### Distribution.

**Figure 13.**
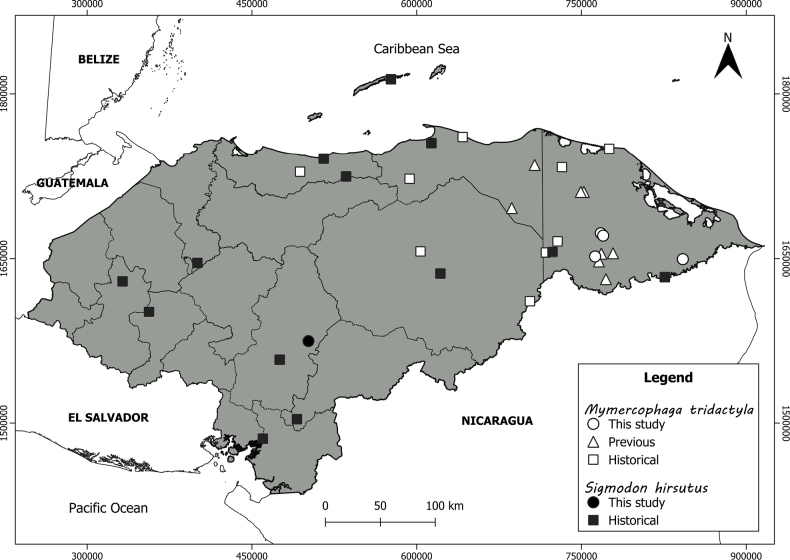
Distribution of *Myrmecophaga tridactyla* and *Sigmodon hirsutus* in Honduras.

Colón, Gracias a Dios, and Olancho (Fig. 13); 0–600 m a.s.l. Historical records from Atlántida may indicate that the species is extirpated from that area.

####### Additional notes.

They are known as winku tara by the Miskitus in eastern Honduras (Jones 1965). The giant anteater is considered the most threatened xenarthran in Honduras (Turcios-Casco et al. 2020b). Given that Belize was formerly known as British Honduras from 1783 to 1964 and was renamed Belize only in 1973, early references to “Honduras” often caused confusion in early collection records (Miller and Miller 2025). Some trade skins may have been labelled “Honduras” or “Belize,” reflecting the port of origin of Belize City rather than the collection location. The northernmost report from southern Belize is from near Punta Gorda (Alston 1879–1882), representing the third-hand account of the giant anteater by Mr. Sarg, who related to Godman (1915) an anecdotal observation that one had been killed near Punta Gorda on the coast of the Bay of Honduras. This has been the basis for the assumption that this species once occurred in Belize. During a 3.5-year (2001–2005) program, the Honduran Program of Biological Monitoring verified four new records of the giant anteater in the Moskitia (Reyes et al. 2010). Based on the results of this program, it has been suggested that the giant anteater may have been eradicated from the Honduran Caribbean coastal area (Reyes et al. 2010). There is evidence that this species has been captured in eastern Honduras for sale in Asia (McCain 2001). It is hypothesized that there are only two populations in Honduras. The northernmost population is in northern RPBR, and another population is in southern Honduras, including parts of Olancho and Gracias a Dios. It is assumed these are disjunct, as there is no longer a landscape connection between them, as connectivity between the forests within the Patuca region has been lost (Martínez et al. 2022). Due to habitat loss, the Wuarska population may disappear. Global conservation status is VU and CR in Honduras.

##### *Tamandua* Gray, 1825

###### 14. Tamandua mexicana

Taxon classificationAnimaliaPilosaMyrmecophagidae

(Saussure, 1860)

FAF40EC8-97D9-53C6-AA72-2DAC6B0E46FB

####### Natural history.

We have documented individuals carrying offspring in March, suggesting that reproduction may occur during the fall rainy season. The tamandua is primarily arboreal but frequently descends to the ground to forage. It inhabits a wide range of ecosystems, including lowland rainforests, pine-oak forests, secondary vegetation, and even agricultural landscapes, showing high ecological adaptability. It is mainly nocturnal and solitary, feeding predominantly on ants and termites, which it extracts from nests using its elongated snout and long, sticky tongue. When threatened, individuals exhibit defensive postures, standing upright and using their forelimbs to strike. Some local people report that tamanduas may attack their dogs if threatened.

####### Distribution.

**Figure 14.**
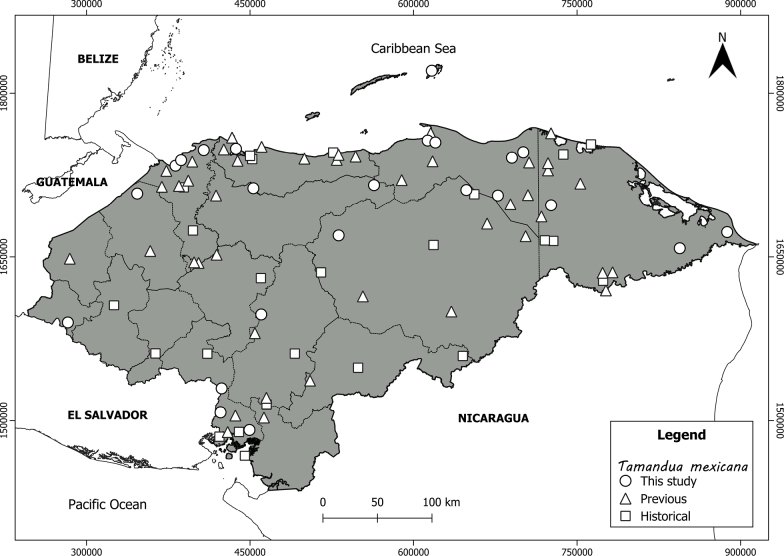
Distribution of *Tamandua mexicana* in Honduras.

Expected to occur throughout the country (Fig. 14); 0–2050 m a.s.l.

####### Additional notes.

They are known as winku or winku sirpi by the Miskitus in eastern Honduras. This is among the most widely distributed xenarthrans in Honduras. Historical records refer to this species as *Tamandua tetradactyla* (Linnaeus, 1758). Recently, camera-trap data confirmed the occurrence on the island of Guanaja, Islas de la Bahía. It was apparently introduced by indigenous peoples and has since become established. Turcios-Casco et al. (2020b) corrected a record from 1937 to Catacamas, Olancho department. Global conservation status is LC and in Honduras NE.

#### Sirenia Illiger, 1811

**Trichechidae Gill, 1872**

##### *Trichechus* Linnaeus, 1758

###### 15. Trichechus manatus

Taxon classificationAnimaliaSireniaTrichechidae

Linnaeus, 1758

08DF170F-F073-57C4-9A58-CF3E70E7E0C6

####### Natural history.

This species inhabits coastal and riverine habitats. American manatees may use marine habitats for prolonged periods but must always remain near a freshwater source to drink (Olivera-Gómez and Mellink 2005). No manatees have been radio tagged in Honduras, but nearby populations in Mexico and Belize indicate that manatees prefer freshwater habitats when available. Manatees are capable of long-distance movements, as evidenced by tagged manatees migrating several hundred kilometers between Mexico and Belize (Castelblanco-Martínez et al. 2012, 2013); however, they often remain for long periods within 25 km of where they were captured (Deutsch et al. 2022). Migration appears to be seasonal, with manatees more likely to travel to and use marine habitats during the rainy season (Deutsch et al. 2022).

####### Distribution.

**Figure 15.**
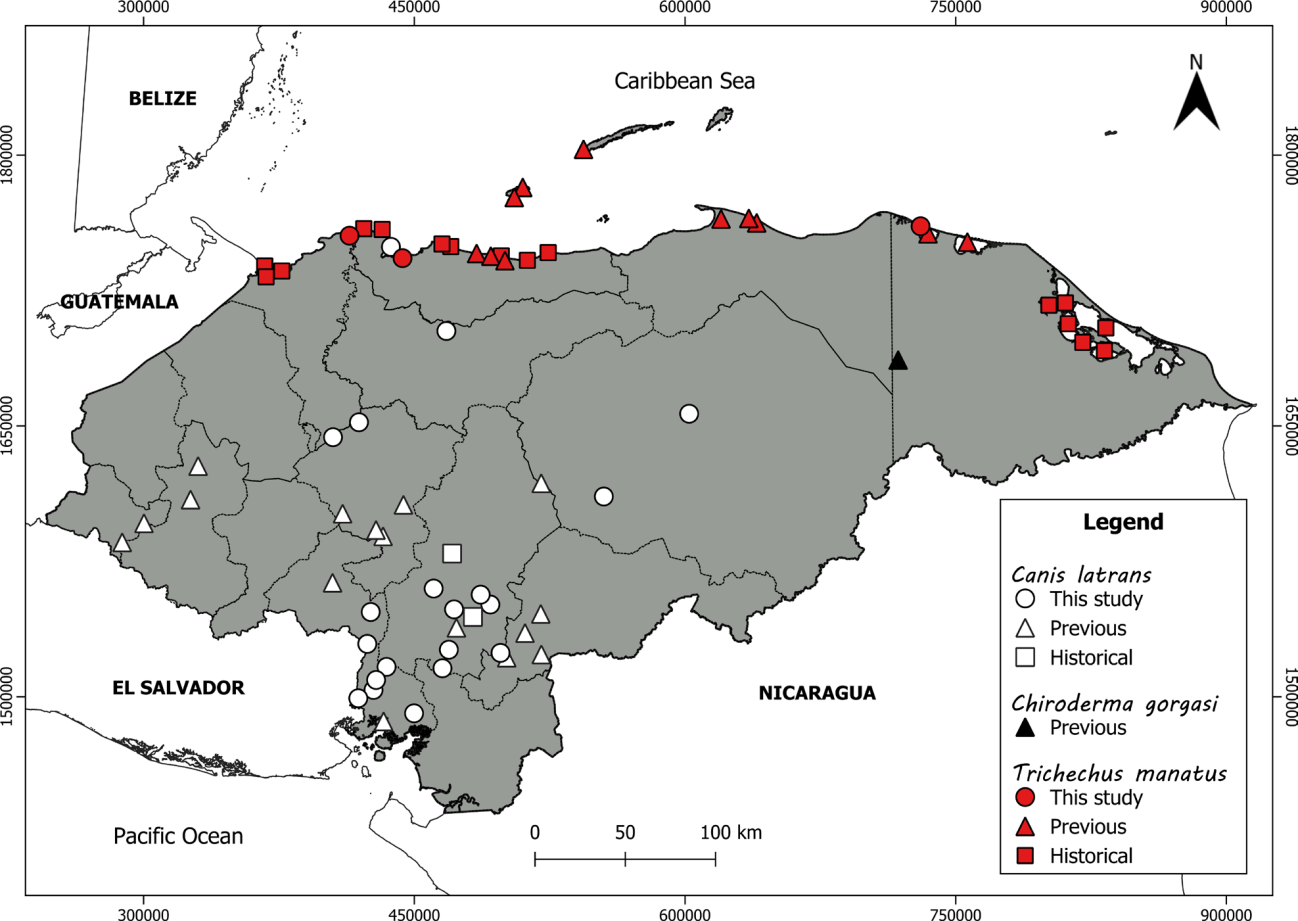
Distribution of *Canis latrans*, *Chiroderma gorgasi*, and *Trichechus manatus* in Honduras.

Manatees are present in all departments on the Caribbean coast (Cortés, Atlántida, Colón, Gracias a Dios, and Islas de la Bahía departments; Fig. 15). However, aerial surveys indicate three primary areas used by manatees in Honduras, the rivers between Tela and La Ceiba (Lean, San Juan, Salado, and Cuero Rivers), the rivers east of Trujillo (Chapagua and Aguán rivers), and the extensive region of La Moskitia (Klein 1979; Rathbun et al. 1983; Marineros and Martínez 1998; Gonzalez-Socoloske et al. 2011). Most manatee sightings occur in rivers and estuaries; however, some marine coastal sightings have been reported in the Bahía de Omoa (Quintana-Rizzo 2005). Greater Caribbean manatees were historically reported to occur around the Islas de la Bahía (Strong 1935); and thought to have been extirpated by the 1970s (Rathbun et al. 1983; Gonzalez-Socoloske et al. 2011). Recent credible reports from citizen scientists on the island of Roatán indicate that this may represent an expansion of the mainland population or simply a few wandering individuals.

####### Additional notes.

They are known as palpa by the Miskitus in eastern Honduras. According to the natives on the Miskitu coast, manatees were abundant in the past and were frequently hunted as an important source of protein. In the village of Tansin, in the Caratasca Lagoon System, there is a church temple that the locals claim was constructed and financed mainly by the sale of manatee meat some 70 years ago. Entanglement in fishing nets accounts for ~ 50% of mortality (Gonzalez-Socoloske et al. 2011). Other threats include habitat loss and poaching. Manatees are still hunted and consumed in some parts of eastern Honduras. The Honduran population is recognized as the Greater Caribbean subspecies, *T. m. manatus*, of the American manatee (Mignucci-Giannoni et al. 2024). Wells (1857) mentioned that a popular dish for Englishmen on the Atlantic slope of Honduras was manatee in curry sauce. Global conservation status is VU and CR in Honduras.

#### Primates Linnaeus, 1758

Even though Turcios-Casco et al. (2021a) recognized three species of Primates as occurring in Honduras, the Mammal Diversity Database (2026) suggests that *Cebus capucinus* (Linnaeus, 1758) includes *Cebus imitator* Thomas, 1903. We agree that the species occurring in Central America is *C. imitator*, as reported by Boubli et al. (2012). Mora and Ruedas (2023) accepted that *C. imitator* is also the *Cebus* species occurring in Costa Rica. Regardless of which name is applied to the Honduran species, the count of taxa present in Honduras will not change.

#### Atelidae Gray, 1825

As a conservation note, all species in the region are considered at risk due to hunting in the northeast, including the protected areas Misoco Biological Reserve, the Montecillos Biological Reserve, and the RPBR. They are hunted for food and for alleged medicinal purposes. Typically, they are hung upside down to drain the fat, which is believed to treat bone diseases, diabetes, and pulmonary conditions. Local lore suggests that only caciques consume the brains.

##### *Alouatta* de Lacépède, 1799

###### 16. Alouatta palliata

Taxon classificationAnimaliaPrimatesAtelidae

(Gray, 1849)

A4F9044D-AFF9-5D26-B074-0B63ACDEB6D2

####### Natural history.

*A. palliata* is the only species of the genus that in the country; see Turcios-Casco et al. (2021a) for a discussion of misidentified specimens of *A. pigra* Lawrence, 1933. Goodwin (1942a) reported that individuals from Copán exhibit lighter neck and shoulder coloration. Gonzalez-Socoloske and Snarr (2010) observed an adult male swimming across a 150 m stretch of the Salado River (Cuero y Salado Wildlife Refuge, Atlantida) demonstrating that while uncommon, rivers are not effective barriers for this species as was previously thought. Individuals feed on leaves of Fabaceae Lindl., Rubiaceae Juss., and Moraceae Gaudich. We have observed them self-anointing using leaves from the family Momiaceae Juss.

####### Distribution.

**Figure 16.**
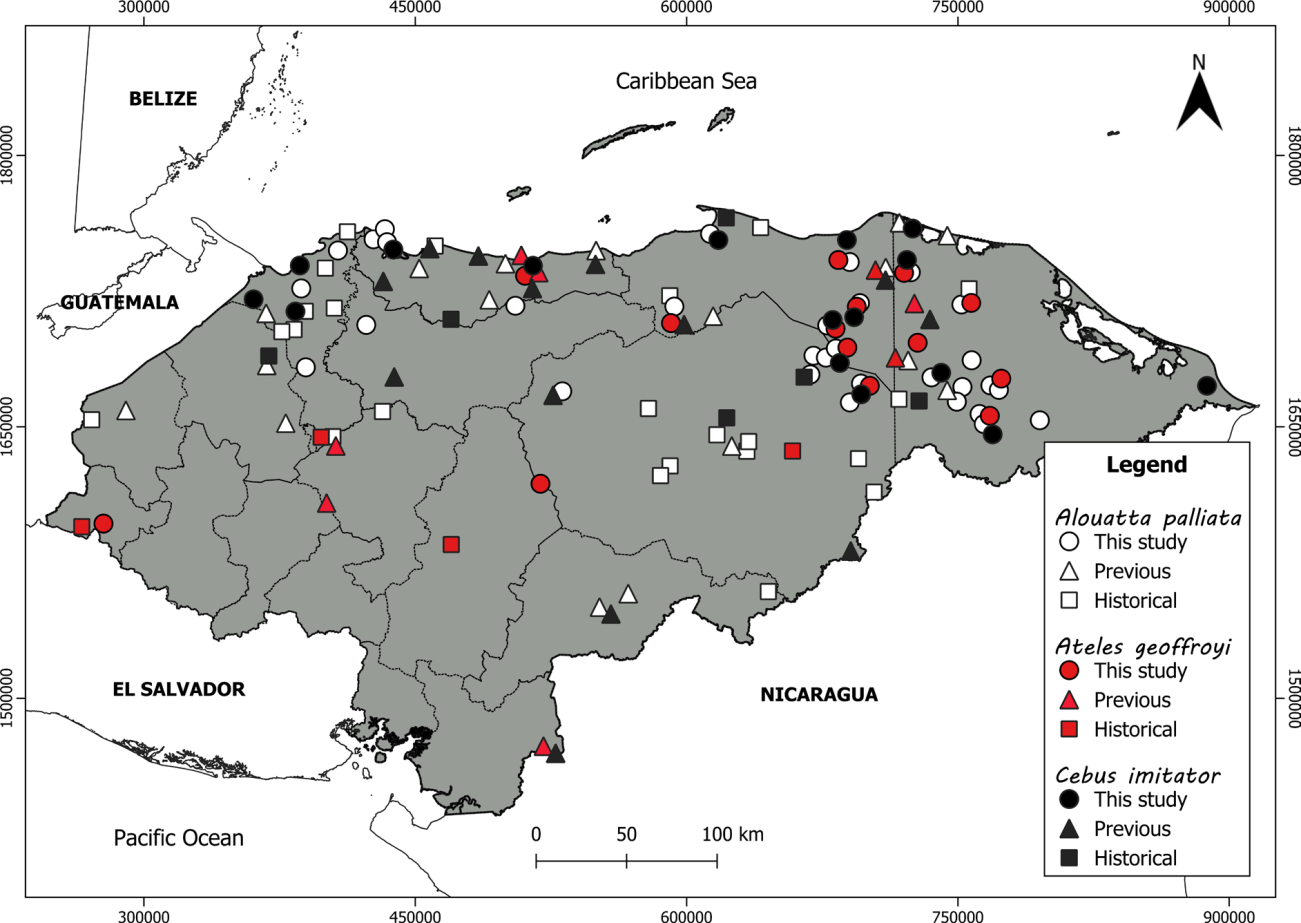
Distribution of *Alouatta palliata*, *Ateles geoffroyi*, and *Cebus imitator* in Honduras.

Atlántida, Colón, Comayagua, Copán, Cortés, El Paraíso, Gracias a Dios, Olancho, Santa Bárbara, and **Yoro** (Fig. 16); 0–2200 m a.s.l.

####### Additional notes.

They are known as kung-kung by the Miskitus in eastern Honduras (Jones 1965); they are also known as olingo, mono colorado or mono barba-roja, but the most common name is mono aullador. Although uncommon, there is evidence that some are kept as pets. Global and national conservation status is VU.

##### *Ateles* É. Geoffroy Saint-Hilaire, 1806

###### 17. Ateles geoffroyi

Taxon classificationAnimaliaPrimatesAtelidae

Kuhl, 1820

47BCD212-34AA-583E-9275-82655437FDB4

####### Natural history.

The subspecies occurring in the country is *Ateles geoffroyi vellerosus* (Gray, 1842). In July, a female with her offspring was observed in Cerro Azul Meámbar National Park (PANACAM), Comayagua department (Midence 2020), and juveniles were observed in the Corralitos Wildlife Refuge in Francisco Morazán, Honduras, in May. A case of albinism was reported in a juvenile female in San Pedro de Pisijire in Dulce Nombre de Culmí in eastern Honduras (Espinal et al. 2016). Groups typically consist of 4–20 individuals. Indigenous hunters in La Moskitia reported using spider monkeys as bait for jaguars during the commercial jaguar pelt trade in the 1950s and 1960s (for more details, see the jaguar natural history section). Additionally, this species is highly prized as a food source among the Miskitu and Tawahka people. Self-anointing behavior has been documented in captive *Ateles geoffroyi* in Honduras, where individuals rubbed citrus fruits (*Citrus* sp.) on their fur, particularly in the sternal and axillary regions; this behavior has been interpreted as potentially related to olfactory communication and chemical signaling within the species in an ex-situ conservation center (Paz-Sánchez 2023). They like to feed on leaves and fruits of Sapotaceae. We have observed them rubbing leaves from the family Momiaceae Juss. on some occasions.

####### Distribution.

Atlántida, Choluteca, Colón, Comayagua, Cortés, Francisco Morazán, Gracias a Dios, Olancho, and Ocotepeque. (Fig. 16); 0–2100 m a.s.l.

####### Additional notes.

They are known as muskoka by the Miskitus in eastern Honduras. Groups of up to seven individuals have been reported. There are isolated populations in central Honduras: at the Biological Reserve Misoco in the Francisco Morazán department, at the Biological Reserve Montecillos (Meza 2018), and at PANACAM (Midence López 2020) in the Comayagua department, with no connections among them. A group of four individuals was observed in the dry forest of the Guanacaure protected area in Choluteca, Honduras, in 2006. Although prohibited (La Gaceta 2017), it is the most commonly kept monkey in captivity in the country, with individuals living up to 15 years. Global and national conservation status is EN.

#### Cebidae Bonaparte, 1831

##### *Cebus* Erxleben, 1777

###### 18. Cebus imitator

Taxon classificationAnimaliaPrimatesCebidae

Thomas, 1903

EEF51D6E-5B62-5DBF-95FD-76E9A2435B54

####### Natural history.

Lactating females were found in April. Pinel Ramos (2020) estimated 1.04 groups per km^2^ and 4.96 individuals per km^2^ in a remnant of dry forest in southern Honduras. See Buckley (1983) for comments on the behavior of capuchin monkeys in Trujillo in northern Honduras. We have observed groups with up to 12 individuals. Capuchins have been recorded by ground-level camera traps in the Tulian, Merendón Reserve Zone, Sierra Río Tinto National Park, and Jeannette Kawas National Park. We have observed them self-anointing using leaves from the family Rutaceae.

####### Distribution.

Atlántida, Choluteca, Colón, Cortés, El Paraíso, Gracias a Dios, Olancho, Santa Bárbara, and Yoro (Fig. 16); 0–900 m a.s.l.

####### Additional notes.

With the elevation of the subspecies to full species (Boubli et al. 2012), we consider the historical records of *Cebus capucinus limitaenus* Hollister, 1914, to be *C. imitator*. This is further supported by recognition of the species in Costa Rica (Mora and Ruedas 2023). They are known as waklin among the Miskitu in eastern Honduras. The population in southern Honduras, in the Choluteca department, is likely connected to Nicaragua along the Segovia River (Pinel-Ramos 2020). We assume this may be a disjunct population, as there is no connection between this population and populations in other regions of the country. The species is threatened by deforestation and illegal trafficking (Pinel-Ramos 2020). They have been introduced to the island of Roatán in Islas de la Bahía as a tourist attraction and as pets. The major threats to *Cebus imitator* in Honduras are deforestation, habitat fragmentation, and illegal wildlife trafficking. Global and national conservation status is VU.

#### Lagomorpha Brandt, 1855

We consider historical specimens identified as either *Sylvilagus brasiliensis* (Linnaeus, 1758) or *S. floridanus hondurensis* Goldman, 1932 to be *Sylvilagus hondurensis* Goldman, 1932 and specimens identified as *S. brasiliensis gabbi* to be *Sylvilagus gabbi* (J. A. Allen, 1877). A specimen identified as *S. tapeti* (MCZ 29261- GBIF.org 2023) from Honduras needs verification.

#### Leporidae Fischer, 1817

##### *Sylvilagus* Gray, 1867

Recent taxonomic changes proposed by Ruedas et al. (2023) suggest that both *S. gabbi* and *S. hondurensis* may occur here; although a thorough review is needed to differentiate them, we assume that both species occur in the country. Additionally, we believe the specimen identified as *S. tapeti* (MCZ 29261; GBIF.org 2023) was misidentified and not from Honduras. Wells (1857) noted that a popular dish for Englishmen on Honduras’s Atlantic slope was rabbits cooked in an Indian style. In some regions of Honduras, currently rabbit meat is highly prized and is one of the most commonly consumed types of wild game.

###### 19. Sylvilagus gabbi

Taxon classificationAnimaliaLagomorphaLeporidae

(J. A. Allen, 1877)

6AE300F5-5F16-5C7D-8B79-551747F11203

####### Natural history.

It can be found within rural areas.

####### Distribution.

**Figure 17.**
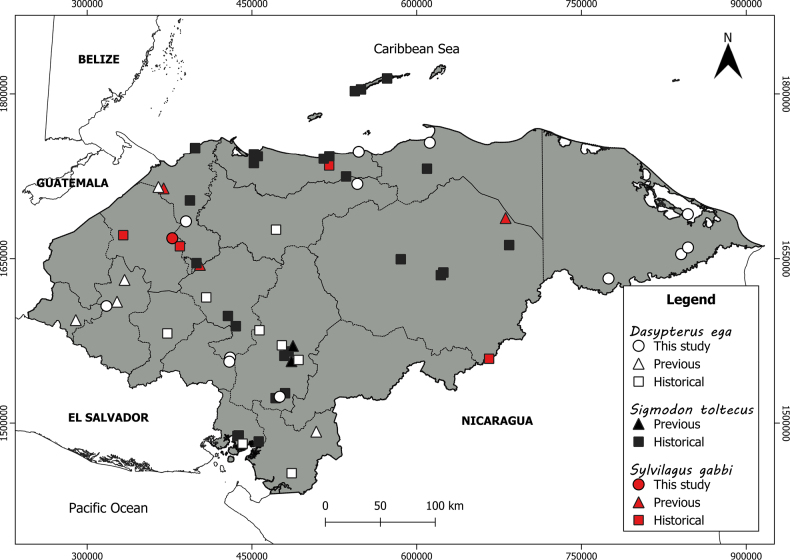
Distribution of *Dasypterus ega*, *Sigmodon toltecus*, and *Sylvilagus gabbi* in Honduras.

Atlántida, Cortés, Olancho, and Santa Bárbara (Fig. 17); 60–750 m a.s.l.

####### Additional notes.

Historical specimens previously identified as *S. brasiliensis gabbi* are recognized as *S. gabbi*. There appears to be no hunting preference between the two species of rabbits. Local communities occasionally hunt both species for subsistence purposes, but at a relatively low intensity. However, local names differ, with *S. gabbi* called conejo de campo and *Sylvilagus hondurensis* called liebre. Global conservation status is LC and NE in Honduras.

###### 20. Sylvilagus hondurensis

Taxon classificationAnimaliaLagomorphaLeporidae

Goldman, 1932

069C17D4-00D7-5F91-943D-F8D1613CACC9

####### Natural history.

Goodwin (1942a) noted differences in pelage throughout the year: duller in January and in summer (June); however, overall, this is a dark-colored species. The Honduran Rabbit has been identified as one of the wildlife species most frequently associated with cassava crop damage in eastern Honduras. Recent observations indicate that non-invasive deterrent methods, particularly artificial light systems, can effectively reduce crop incursions and may provide a practical alternative to lethal control measures.

####### Distribution.

**Figure 18.**
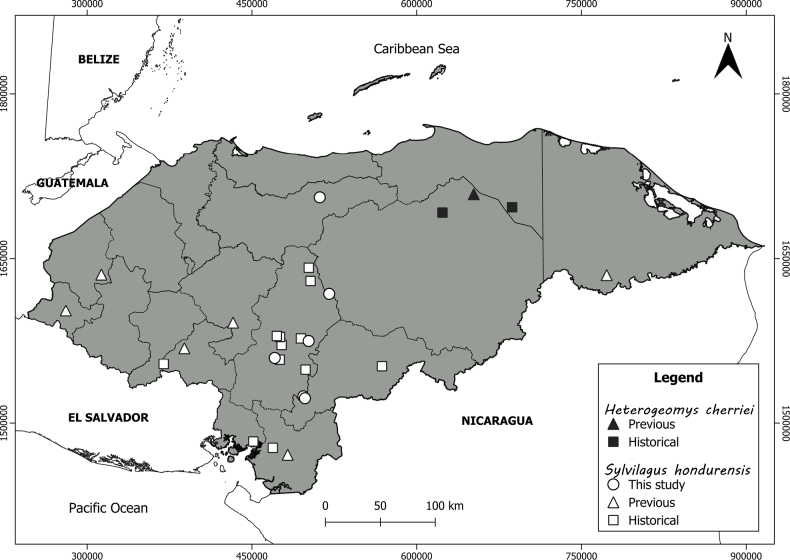
Distribution of *Heterogeomys cherriei* and *Sylvilagus hondurensis* in Honduras.

Choluteca, Comayagua, Copán, El Paraíso, Francisco Morazán, Gracias a Dios, La Paz, Ocotepeque, Valle, and Yoro (Fig. 18); 60–2000 m a.s.l.

####### Additional notes.

They are known as tibang by the Miskitus in eastern Honduras (Jones 1965). Historical specimens previously identified as either *S. brasiliensis* or *S. floridanus hondurensis* are considered here to be *S. hondurensis*. Goodwin (1942a) noted that specimens from Santa Bárbara are lighter in color, have a very small bulla, and recommended that further material be collected to determine whether this is a distinct taxon found in the low arid land of northern Honduras. To our knowledge, no further collections have substantiated this hypothesis but see Ruedas et al. (2023) for a discussion of the taxonomy. This species is frequently confused with *S. gabbi* in camera-trap images (e.g., as shown in Sánchez et al. 2023). Historically, *Sylvilagus* have been one of the preferred species for subsistence hunting. Global conservation status is LC and NE in Honduras.

#### Rodentia Bowdich, 1821

For Geomyidae, we follow Spradling et al. (2016). For the family Muridae, although we did not examine specimens of *Rattus norvegicus* (Berkenhout, 1769), there is verifiable evidence of the species’ presence in Honduras (Puckett et al. 2016). Notably, Espinoza Montero (2015) published a photo of *R. norvegicus* from central Honduras. For the family Sciuridae, we recognize the use of *Echinosciurus* Trouessart, 1880, for the genus of squirrels, *Echinosciurus deppei* Peters, 1863, and *Echinosciurus variegatoides* Ogilby, 1839, following de Abreu et al. (2020, 2022). For the family Cricetidae, we recognize that the species occurring in Honduras is *Baiomys brunneus* (J. A. Allen & Chapman, 1897), following Hernández-Canchola and León-Paniagua (2021). We follow López et al. (2024) and suggest that the *Peromyscus* Gloger, 1841, species occurring in Honduras are *Peromyscus beatae* Thomas, 1903; *Peromyscus nicaraguae* J. A. Allen, 1908; *Peromyscus cordillerae* Dickey, 1928; *Peromyscus salvadorensis* Dickey, 1928; and *Peromyscus stirtoni* Dickey, 1928. Although Reid (2009) noted that *Peromyscus gymnotis* O. Thomas, 1894 occurs in Central America, we found no evidence of it in Honduras; the gap between Nicaragua and the remaining countries suggests that this is likely a polytypic cryptic species complex.

*Reithrodontomys gracilis* J. A. Allen & Chapman, 1897, was not confirmed to occur here, as suggested by the Mammal Diversity Database (2026), and we include it only as an expected species, even though it has been assessed in the Honduran Red List (WCS 2021) without supporting evidence. A specimen of *Rheomys* Thomas, 1906, from Cusuco National Park in northwestern Honduras was recorded by Gilroy et al. (2017). Martin et al. (2021) noted that more specimens have been collected and that they differ morphologically from other *Rheomys* species, suggesting they may represent a new taxon. We also accept the proposal of *Casiomys* by Voss (2024).

#### Cuniculidae Miller & Gidley, 1918

##### *Cuniculus* Brisson, 1762

###### 21. Cuniculus paca

Taxon classificationAnimaliaRodentiaCuniculidae

(Linnaeus, 1766)

4466B6B2-0BEA-55B6-98F1-12EF07493F2C

####### Natural history.

They mainly consume fruits and seeds of palms (e.g., *Attalea* Kunth). Females with offspring have been noted in October and November. Activity is variable, occurring mainly at night. They use burrows along river banks or dense vegetation for shelter. When females are raising their young, they tend to emit loud distraction sounds and may leave the burrow to distract predators or hunters and lead them away, while the young remain in the burrow.

####### Distribution.

**Figure 19.**
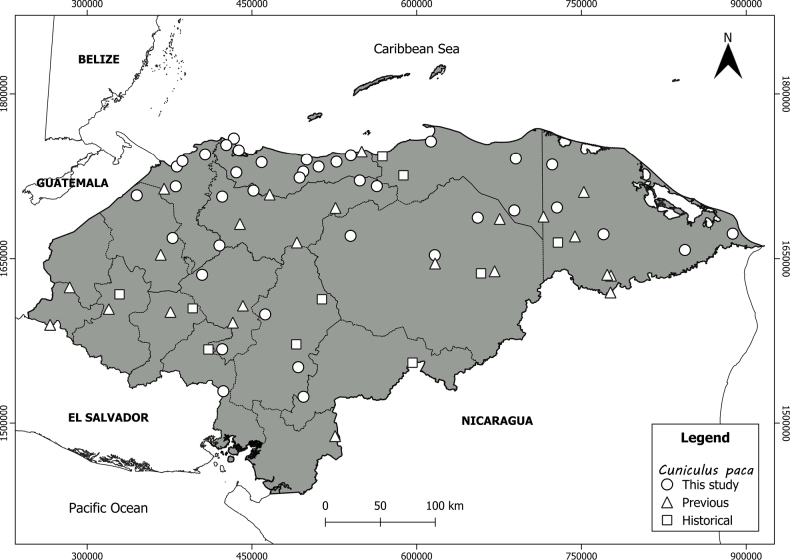
Distribution of *Cuniculus paca* in Honduras.

Atlántida, Choluteca, Colón, Comayagua, Cortés, El Paraíso, Francisco Morazán, Gracias a Dios, Intibucá, La Paz, Lempira, Ocotepeque, Olancho, Santa Bárbara, Valle, and Yoro (Fig. 19); 0–2140 m a.s.l. Bermingham et al. (1998) reported pacas on Cayos Cochinos and Guanaja Island in the Islas de la Bahía department, and by fishermen from Utila Island in the 1970s. It is important to determine if the species has been introduced across all the islands.

####### Additional notes.

Historical specimens from Honduras might be referred to *Agouti paca nelsoni* (Goldman, 1913). They are known as ibihna among the Miskitus in eastern Honduras (Jones 1965), but are commonly called tepezcuintles. Historically, culturally, and anecdotally, pacas have been hunted in all the regions of Honduras by ladino and indigenous people. The most highly valued food made from them is chicharrones (fried meat with the skin on). Rural residents near protected areas or areas with forest remnants still hunt pacas because they are considered a desirable bushmeat. The pacas are among the most highly valued game species in Honduras and are subject to intense hunting pressure throughout much of its range. Hunting strategies vary geographically. In the Caribbean region, hunters commonly search for animals occupying caves, hollow logs, or other refuges, whereas in central and western Honduras, elevated hunting platforms (tapescos) are frequently used; which are platforms high in the trees where the hunter climbs and waits for the animal to come by. There have been some attempts to keep this species in game ranches around the country, but this has not been successful. Hunters will urinate in plastic bottles while waiting, believing otherwise the scent will deter pacas from using the area. Similar practices have also been reported from La Moskitia. Hunters occasionally report exceptionally large individuals exceeding 10 kg in body mass. Environmental education programs can help reduce hunting pressure and promote the ecological importance of pacas as seed dispersers. Despite its broad distribution, local declines have been reported in some regions. In portions of southern Honduras, residents often consider the species extremely rare, and some communities question whether it still occurs locally. These perceptions likely reflect long-term population reductions driven by overhunting and habitat modification. Given its ecological role as a large seed predator and disperser, continued monitoring of *C. paca* populations is warranted throughout the country. This species is particularly vulnerable to forest fires, which commonly occur in pine forests as well as seasonally dry tropical forests. Following severe fire events, whether natural or human-induced, it is not uncommon to encounter burned carcasses, suggesting that wildfires can represent a significant source of mortality in affected populations. Global conservation status is LC and VU in Honduras.

#### Dasyproctidae Bonaparte, 1838

##### *Dasyprocta* Illiger, 1811

###### 22. Dasyprocta punctata

Taxon classificationAnimaliaRodentiaDasyproctidae

Gray, 1842

B0816573-9FFD-57EC-B68E-F6A1A4B7B710

####### Natural history.

We have found offspring throughout the year and groups up to three in camera traps. *D. punctata* is a diurnal terrestrial rodent that inhabits tropical rainforests, dry forests, gallery forests, and secondary growth. A case of albinism was reported in northeastern Honduras by Elvir Valle et al. (2021), and similar individuals have been observed in ex situ conservation centers in western Honduras. The species has also been introduced to other islands in the Islas de la Bahía department. Agoutis are primarily frugivorous, feeding on fruits, seeds, and nuts, and are important seed dispersers that influence forest regeneration. They are highly territorial and use scent marking to delineate their ranges. The Central American Agouti is among the most common large rodents in Honduras and is widely distributed across a variety of habitats. In several regions, local abundance appears closely associated with areas containing mango trees, where fallen fruits provide an important seasonal food resource. Although the species is not typically a primary target for hunters, opportunistic hunting does occur in some rural areas. As an important seed predator and disperser, agoutis likely play a significant role in forest regeneration processes throughout much of the country.

####### Distribution.

**Figure 20.**
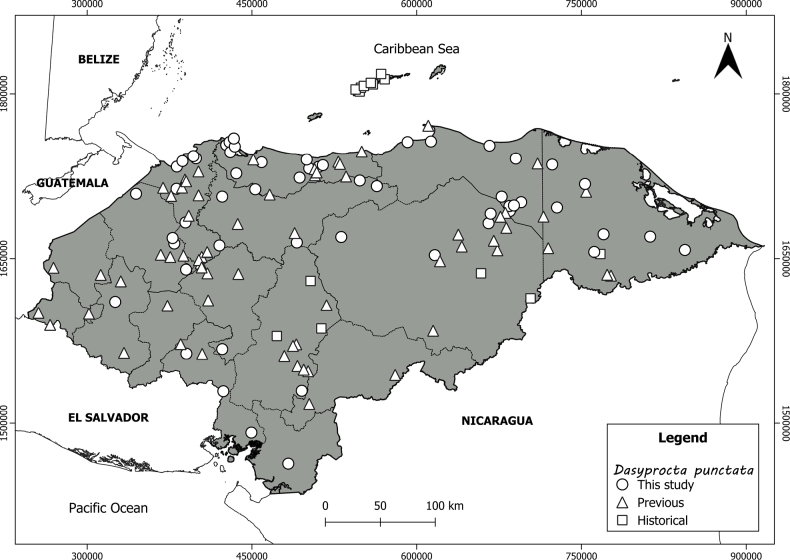
Distribution of *Dasyprocta punctata* in Honduras.

All departments (Fig. 20); 0–2200 m a.s.l.

####### Additional notes.

The recognition of *Dasyprocta ruatanica* Thomas, 1901 as a distinct species was unclear. Teta and Reyes-Amaya (2021) suggested that insular species might be smaller individuals of continental species, and Ruiz-Garcia et al. (2022) and Turcios-Casco et al. (2026) concluded that *D. ruatanica* is a synonym of *Dasyprocta punctata punctata* Gray, 1842, based on morphological evidence. Therefore, herein we treat *D. ruatanica* as a synonym of *D. punctata*.

Agoutis are known as kiski or kiahki by the Miskitus in eastern Honduras and commonly called guatuzas in Spanish. Prized for their meat, agoutis, like pacas, are among the most hunted mammals in Honduras. Natural predators include jaguars, cougars or pumas, and coyotes. Introduced populations on islands may influence local ecosystems, but the species has demonstrated resilience in both mainland and island habitats. The main threats to *D. punctata* are hunting pressure and habitat loss. In some regions of eastern Honduras, they are highly valued for their meat. Hunting for subsistence or commercial purposes is widespread, particularly in rural and forested areas. Deforestation for agriculture, cattle ranching, and urban expansion reduces available habitat and resources. Nonetheless, agoutis are commonly seen in coffee, cacao, and oil palm plantations. Environmental education programs can help reduce hunting pressure and promote the ecological importance of agoutis as seed dispersers. The subpopulation of Islas de la Bahía should be reassessed, as other subspecies occur on the islands and could extirpate this putatively endemic subspecies. Similar to pacas, agoutis are particularly vulnerable to forest fires, which frequently occur in pine-dominated forests as well as seasonally dry tropical ecosystems. After intense fire events, whether natural or anthropogenic in origin, burned carcasses are sometimes observed, indicating that wildfires may represent an important source of mortality in affected populations. Global conservation status is LC and NT in Honduras.

#### Erethizontidae Bonaparte, 1845

##### *Coendou* Lacépède, 1799

###### 23. Coendou mexicanus

Taxon classificationAnimaliaRodentiaErethizontidae

(Kerr, 1792)

1627F7A6-DB81-52A0-8542-755B05259F52

####### Natural history.

We have found offspring throughout the year. Individuals are primarily nocturnal, resting during the day, and are frequently recorded by camera traps foraging on the ground with peak activity in the early morning hours. *C. mexicanus* is an arboreal and nocturnal rodent, though it also exhibits terrestrial foraging behavior. It inhabits a wide range of ecosystems, including tropical rainforests, pine-oak forests, cloud forests, and secondary growth areas. The species primarily feeds on fruits, seeds, and young leaves, and occasionally on bark. It is a solitary species that uses tree cavities or dense vegetation as diurnal shelters. Communication occurs through vocalizations and scent marking. When threatened, individuals erect their spines as a defensive display. Local people believe that porcupines can shoot or throw their quills.

####### Distribution.

**Figure 21.**
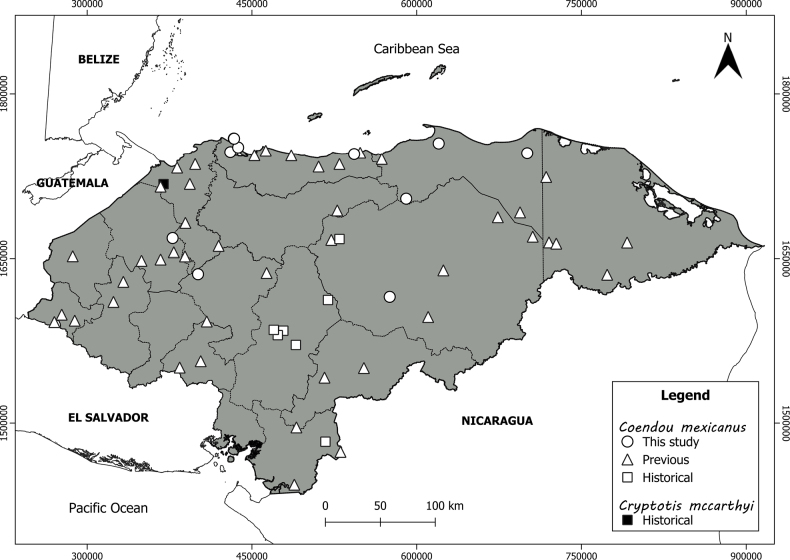
Distribution of *Coendou mexicanus* and *Cryptotis mccarthyi* in Honduras.

Atlántida, Choluteca, Colón, Comayagua, Copán, Cortés, El Paraíso, Francisco Morazán, Gracias a Dios, La Paz, Lempira, Ocotepeque, Olancho, Santa Bárbara, and Yoro (Fig. 21); 0–1900 m a.s.l.

####### Additional notes.

Historical records from Honduras have been identified as *Coendou mexicanum mexicanum* Miller, 1912, and *Sphiggurus mexicanus* (Kerr, 1792). They are known as kiski (sikiski) kiaikirra, or aksuk by the Miskitus in eastern Honduras (Jones 1965). Marineros-Sánchez et al. (2018) noted that in La Moskitia, porcupines are hunted for their fat, which is used for medicinal purposes, as well as for their flavorful meat. Poachers often target these animals due to the misconception that porcupines can throw their quills and injure dogs. Human encounters with porcupines frequently result in injuries; reports from rural communities in Francisco Morazán indicate that individuals have had to remove multiple quills after handling them. A camera trap survey conducted in Jeannette Kawas National Park captured images of an ocelot stalking a porcupine, with subsequent photos showing numerous quills embedded in the ocelot's face. Although porcupines are rare in southern Honduras, they have been found in areas impacted by timber extraction, particularly in *Pinus oocarpa* forests. However, they are a poorly documented arboreal rodent that often goes undetected by ground-level camera trap surveys. Global conservation status is LC and NE in Honduras.

#### Echimyidae Gray, 1825

##### *Hoplomys* J. A. Allen, 1908

During the final proof revision, a proposal to synonymize *Hoplomys gymnurus* (Thomas, 1897) within the genus *Proechimys* J. A. Allen, 1899 was published (Herrera-Martínez et al. 2026). Therefore, we have retained the genus *Hoplomys* in this manuscript, as adopting this taxonomic change would require a thorough review.

###### 24. Hoplomys gymnurus

Taxon classificationAnimaliaRodentiaEchimyidae

(Thomas, 1897)

63FFF7EE-95D2-547C-AD62-8F53D5FA9EB4

####### Natural history.

Lactating and pregnant females (three embryos) were captured in April. They have been observed eating fruits of Sapotaceae Juss (sapote or zapote de montaña), and burrows have been found in the roots of Moraceae and Lauraceae Juss.

####### Distribution.

**Figure 22.**
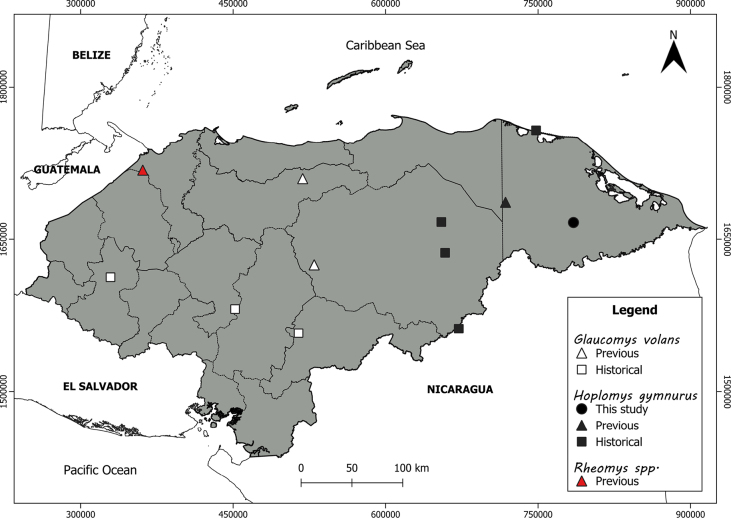
Distribution of *Glaucomys volans*, *Hoplomys gymnurus*, and *Rheomys* spp. in Honduras.

Only known from Olancho and Gracias a Dios (Fig. 22); 0–520 m a.s.l.

####### Additional notes.

In some regions of eastern Honduras, they hunted and highly valued for their meat. Global conservation status is EN and VU in Honduras.

##### *Proechimys* J. A. Allen, 1899

###### 25. Proechimys semispinosus

Taxon classificationAnimaliaRodentiaEchimyidae

(Tomes, 1860)

B1E13AE4-3C76-5F2E-BD7B-7A3D557D2CF1

####### Natural history.

Dead individuals have been found on riversides.

####### Distribution.

**Figure 23.**
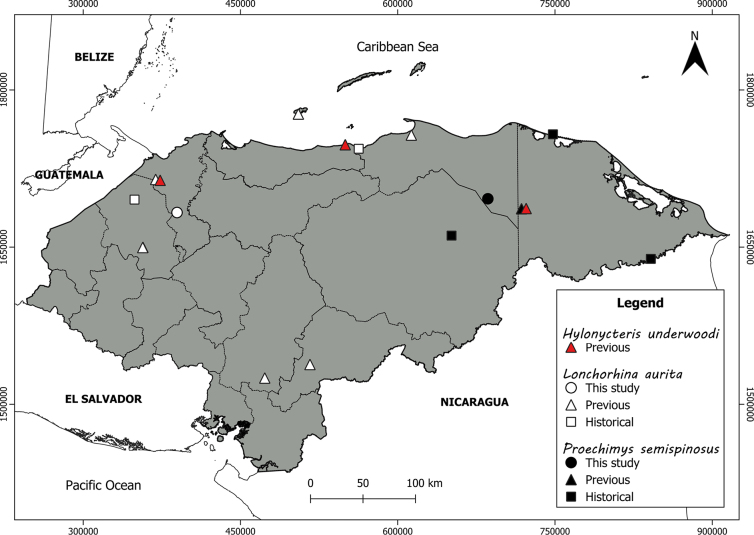
Distribution of *Hylonycteris underwoodi*, *Lonchorhina aurita*, and *Proechimys semispinosus* in Honduras.

Colón, Gracias a Dios, and Olancho (Fig. 23); 0–410 m a.s.l.

####### Additional notes.

Gardner (1983) referred to this species as *Proechimys centralis* J. A. Allen, 1899. In some regions of eastern Honduras, they are highly valued for their meat. Global conservation status is LC and VU in Honduras.

#### Sciuridae Fischer de Waldheim, 1817

##### *Echinosciurus* Trouessart, 1880

Wells (1857) noted that squirrels were hunted in Olancho for meat and did not specify which species. However, this continues throughout the country. We recognize the genus *Echinosciurus*, rather than *Sciurus* Linnaeus, 1758 for the two species occurring here, following de Abreu et al. (2020, 2022).

###### 26. Echinosciurus deppei

Taxon classificationAnimaliaRodentiaSciuridae

Peters, 1863

F93C0C24-15C5-5B79-8DD8-456390B3BC55

####### Natural history.

This squirrel is sympatric with *Echinosciurus variegatoides* W. Ogilby, 1839 is a diurnal and arboreal rodent that inhabits tropical and subtropical forests, including humid lowland forests, pine-oak forests, and secondary growth. It is sympatric with *E. variegatoides*. We recognize two subspecies: *E. deppei deppei* Peters, 1863, and *E. deppei matagalpae* J.A. Allen, 1908. However, Goodwin (1942a) reported variation in color among Honduran populations, suggesting that subspecific distinctions may not be meaningful. *E. deppei* feeds primarily on seeds, fruits, nuts, and occasionally fungi, and is an important seed disperser in forest ecosystems. It nests in tree cavities or constructed dreys and exhibits territorial behavior. It will be important to re-evaluate squirrel populations in southeastern Honduras, as species in the genus *Microsciurus* J. A. Allen, 1985 may yet be found and would be sympatric with this species.

####### Distribution.

**Figure 24.**
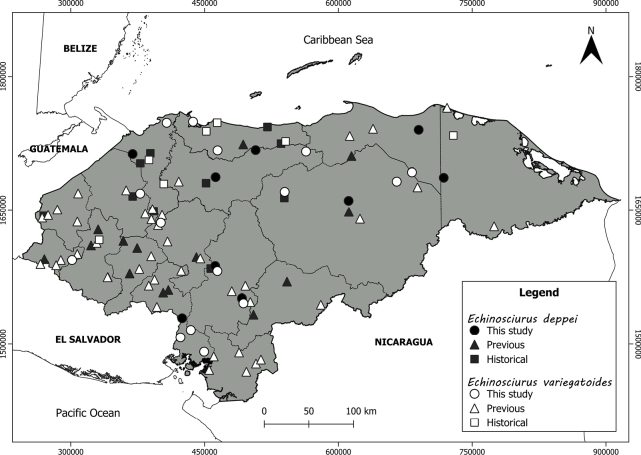
Distribution of *Echinosciurus deppei* and *E. variegatoides* in Honduras.

Atlántida, Colón, Comayagua, Copán, Cortés, El Paraíso, Francisco Morazán, **Gracias a Dios**, Intibucá, La Paz, Lempira, Ocotepeque, Olancho, Santa Bárbara, **Valle**, and Yoro (Fig. 24); 0–2200 m a.s.l. Expected to occur throughout the country.

####### Additional notes.

Individuals have been sold as wild pets for between USD $15–$30 in central Honduras. They are commonly known as moritas. Global conservation is LC and NE in Honduras, as *Sciurus deppei* Peters, 1863.

###### 27. Echinosciurus variegatoides

Taxon classificationAnimaliaRodentiaSciuridae

Ogilby, 1839

E32EA915-E1E5-5A4E-AB25-B9B5CC3E558A

####### Natural history.

*E. variegatoides* is a diurnal and arboreal rodent, sympatric with *E. deppei*. Individuals mainly inhabit pine-oak forests, cloud forests, and secondary growth. Medina-Fitoria and Turcios-Casco (2019) reported that during a survey in Gracias a Dios, a captured *Boa imperator* Daudin, 1803, regurgitated a reproductive male, indicating that large snakes were also predators. In pine-oak forests, they commonly build nests using oak leaves within tree cavities. The species feeds on seeds, fruits, and nuts, and plays an important role in seed dispersal.

####### Distribution.

Found throughout the country, except on the Islas de la Bahía (Fig. 24). Elevations from 0–2100 m a.s.l.

####### Additional notes.

Historically, specimens referred to the previous genus *Sciurus boothiae belti* Gray, 1843. In Santa Ana, central Honduras, dried skins have been sold for around USD $1.20 each during traditional festivities. Like other squirrels, they are also captured and sold as pets in central Honduras. Prices range from $10 to $ 15, depending on the color phase. Colors do not necessarily distinguish the subspecies, and this complex requires revision of its range throughout northern Central America to clarify the limits of each. They are known as butsung or tastas by the Miskitus in eastern Honduras. They are killed during tree felling as they nest in hollow pine trees (*Pinus oocarpa* Schiede ex. Schltdl.) and are frequently roadkill in urban areas. Global conservation status is LC and NE in Honduras, referring to *S. variegatoides*.

##### *Glaucomys* Thomas, 1908

###### 28. Glaucomys volans

Taxon classificationAnimaliaRodentiaSciuridae

(Linnaeus, 1758)

B998BB62-CF90-5DB9-8CA3-8D40E97F6868

####### Natural history.

The recognized subspecies is *Glaucomys volans underwoodi* Goodwin, 1936. It is sympatric with the other two squirrel species in the country. This species was recently rediscovered during logging in Olancho and has subsequently been recorded from Francisco Morazán and Atlántida. This constitutes the southernmost confirmed population of its range. They usually reuse woodpecker *Melanerpes formicivorus* (Swainson, 1827) and other squirrel (i.e., *E. variegatoides*) nests (Turcios-Casco et al. 2023b). Camera trapping has been used to study the species’ natural history (Alvarado-Ortíz et al. 2024).

####### Distribution.

El Paraíso, Francisco Morazán, Lempira, Olancho, and Yoro (Fig. 22); 680–860 m a.s.l.

####### Additional notes.

Medina-Fitoria et al. (2018) noted two unverified records for Nicaragua in the remaining undisturbed pine ecosystem. The species is threatened by poor forestry practices, and, like other squirrels, it is often kept as a pet. It appears to be strongly dependent on woodpecker nests in eastern Honduras. As the pine ecosystems of Honduras are scattered and fragmented without connecting corridors, the populations are disjunct. The Central American and Sierra Madre populations also are discontinuously distributed relative to the North American population, suggesting that they may constitute distinct species-level taxa (e.g., Arbogast et al. 2017). Global conservation status is LC and DD in Honduras.

#### Geomyidae Bonaparte, 1845

Pocket gophers are commonly known as taltuzas. Gómez-Corea et al. (2023) discussed known records of Pocket gophers; however, we retained the historical records and added only the single record of *Orthogeomys grandis* (Thomas, 1893) from La Paz, as the other records were *incertae sedis*.

In crop production systems, particularly maize, beans, and root crops, burrowing activity can damage root systems, destabilize young plants, and reduce overall productivity. These impacts often lead to strong human–wildlife conflict and motivate intensive control efforts by farmers. Management strategies commonly include mechanical destruction of burrow systems, flooding, trapping, and other traditional eradication methods described in local agricultural practices. In some cases, these control efforts are applied repeatedly and indiscriminately across fields. Such intensive and non-selective management may have unintended ecological consequences, particularly for small, poorly studied burrowing mammals and other soil-associated fauna that share similar habitats. Moreover, the lack of species-level ecological information in Honduras limits the ability to assess population trends or differentiate between locally common and potentially vulnerable lineages.

##### *Heterogeomys* Merriam, 1895

###### 29. Heterogeomys cherriei

Taxon classificationAnimaliaRodentiaGeomyidae

(J. A. Allen, 1893)

4480979D-97EB-599D-B582-7615B67A5368

####### Natural history.

There is no information on this species’ natural history or ecology within the country.

####### Distribution.

Colón and Olancho (Fig. 18); 20–1200 m a.s.l.

####### Additional notes.

This species is supported by a report from Medina-Fitoria (2008) in Colón. Molecular analyses by Spradling et al. (2016) suggested that *Orthogeomys matagalpae* (J. A. Allen, 1910), known to occur in northern and central Honduras, and is a synonym of *H. cherriei*. For Honduras, the current subspecies is *H. c. metagalpae* (Pardiñas et al. 2017). It is probably the least-known pocket gopher (taltuza) in Honduras. Global conservation status is NE and DD in Honduras as *Heterogeomys matagalpae*.

###### 30. Heterogeomys hispidus

Taxon classificationAnimaliaRodentiaGeomyidae

(Le Conte, 1852)

9B22701B-E839-5D7B-BA17-279705971BF9

####### Natural history.

Considered an agricultural pest in sugar cane plantations throughout Honduras.

####### Distribution.

**Figure 25.**
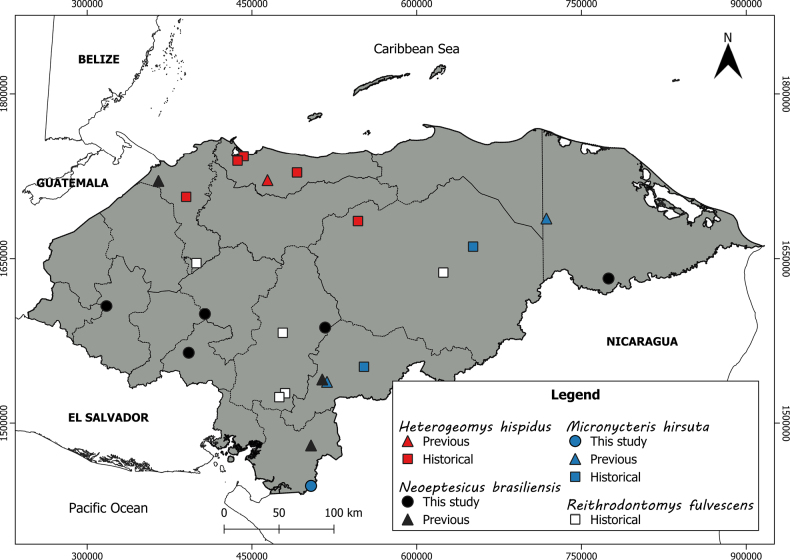
Distribution of *Heterogeomys hispidus*, *Micronycteris hirsuta*, *Neoeptesicus brasiliensis*, and *Reithrodontomys fulvescens* in Honduras.

Atlántida, Cortés, and Olancho (Fig. 25); 15–1060 m a.s.l.

####### Additional notes.

Some historical specimens have been referred to as *Orthogeomys hispidus* (Le Conte, 1852) or *Heterogeomys hispidus hondurensis* Davis, 1966 Recent molecular analyses supported *O. hispidus* as a synonym of *H. hispidus* (Spradling et al. 2016). Pardiñas et al. (2017) recognized the subspecies *H. h. hondurensis* as the one occurring in the country. Like other gophers, they are considered pests and often killed. Global conservation status is NE and DD in Honduras.

##### *Orthogeomys* Merriam, 1895

###### 31. Orthogeomys grandis

Taxon classificationAnimaliaRodentiaGeomyidae

(Thomas, 1893)

61EE9E12-CCC2-5366-9BC8-8C3E357BC614

####### Natural history.

The only subspecies occurring in the country is *Ortheogeomys grandis pluto* Lawrence, 1933, described from specimens collected at Cerro Cantoral in Francisco Morazán (Lawrence 1933). It is considered an agricultural pest in coffee plantations in western and central Honduras and is usually trapped and killed.

####### Distribution.

**Figure 26.**
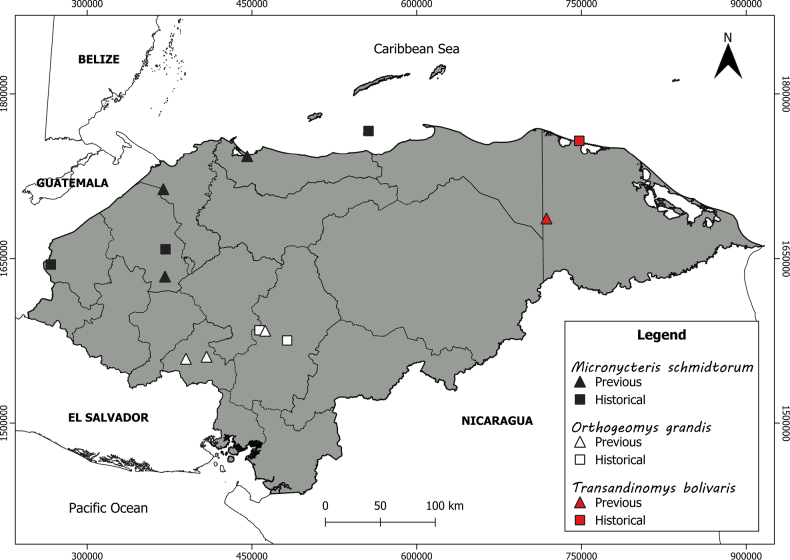
Distribution of *Orthogeomys grandis*, *Micronycteris schmidtorum*, and *Transandinomys bolivaris* in Honduras.

Francisco Morazán and La Paz (Fig. 26); 1400–2060 m a.s.l.

####### Additional notes.

Crop damage caused by taltuzas often leads to conflict with farmers, who typically trap or capture them as a form of pest control. Global conservation statuses LC and NT in Honduras.

#### Heteromyidae Gray, 1868

##### *Heteromys* Desmarest, 1817

Rogers and González (2010) note that further sampling is necessary to confirm the distinct clades within the genus.

###### 32. Heteromys desmarestianus

Taxon classificationAnimaliaRodentiaHeteromyidae

Gray, 1868

A3B18AAC-B902-54B4-95E0-197C386393FA

####### Natural history.

The only subspecies found in Honduras is *Heteromys desmarestianus fuscatus* J. A. Allen, 1908. They have been captured near streams using tuna as bait. The first occurrence of a pseudoscorpion associated with *H. desmarestianus* was recently recorded from Intibucá, along with distribution information and burrows in anthills shared with snakes (e.g., *Tantilla* Baird & Girard, 1853) (López et al. 2026), and the case of false autotomy was presented for the first time for the species (López and Turcios-Casco 2025). Additionally, López et al. (2026) reported individuals feeding in seeds of *Eugenia* L. (Myrtaceae), *Bursera simaruba* (L.) Sarg. (Burseraceae Kunth), and *Dalbergia glomerata* Hemsl. (Fabaceae), and feces in Celaque National Park; and orthopterans in Montecillos Biological Reserve. López et al. (2026) reported lactating females have been captured in January, February, March, and November. They have also been reported to carry phoretic pseudoscorpions (López pers. comm.).

####### Distribution.

**Figure 27.**
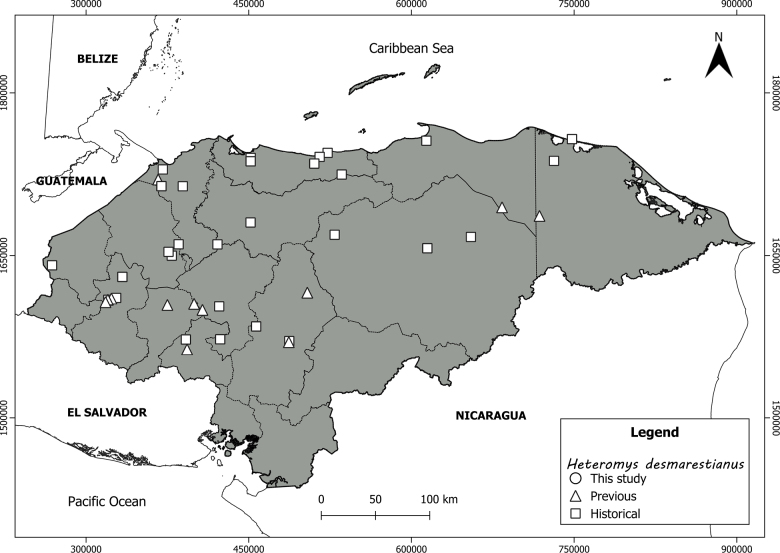
Distribution of *Heteromys desmarestianus* in Honduras.

Atlántida, Colón, Comayagua, Copán, Cortés, Francisco Morazán, Gracias a Dios, Intibucá, La Paz, Lempira, Olancho, Santa Bárbara, and Yoro (Fig. 27); 0–2560 m a.s.l.

####### Additional notes.

Medina-Fitoria and Turcios-Casco (2019) reported that a specimen of *Transandinomys bolivaris* (J. A. Allen, 1901) was erroneously labeled as *H. desmarestianus*. Global conservation status is LC and DD in Honduras.

###### 33. Heteromys salvini

Taxon classificationAnimaliaRodentiaHeteromyidae

Thomas, 1893

D99C1696-3E74-546C-966F-9F02F7FEA048

####### Natural history.

Merriam (1902) listed San Pedro Sula, in Cortés department, as the type locality of *Liomys heterothrix* Merriam, 1902) (formerly a synonym of *H. salvini*). *H. desmarestianus* and *H. salvini* are sympatric in the central region of Honduras (López et al. 2026). Recent notes on their natural history include lactating females in January, a description of burrows as cylindrical cavities with narrow entrances, and the occurrence of Solanaceae and Myrtaceae seeds (López et al. 2026).

####### Distribution.

**Figure 28.**
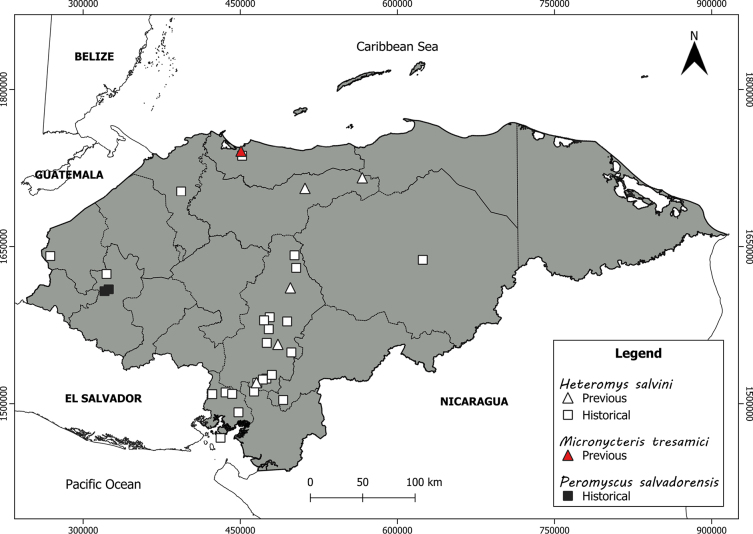
Distribution of *Heteromys salvini*, *Micronycteris tresamici*, and *Peromyscus salvadorensis* in Honduras.

Atlántida, Choluteca, Copán, Cortés, El Paraíso, Francisco Morazán, Lempira, Olancho, Valle, and Yoro (Fig. 28); 100–1305 m a.s.l.

####### Additional notes.

*H. salvini* is common in the valleys of the Río Negro and Río Motagua in northwestern Honduras. Historical records have referred to this species as either *Liomys salvini* (Thomas, 1893), or *L. heterothrix*; see Genoways (1973), Carter and Genoways (1978), and Rogers and González (2010). Global conservation status is LC and DD in Honduras.

#### Cricetidae Fischer, 1817

##### *Baiomys* True, 1894

###### 34. Baiomys brunneus

Taxon classificationAnimaliaRodentiaCricetidae

(J. A. Allen & Chapman, 1897)

5F58E2E5-7D43-51C6-8B01-C073AA6074A0

####### Natural history.

It is common to find it in suburban areas.

####### Distribution.

**Figure 29.**
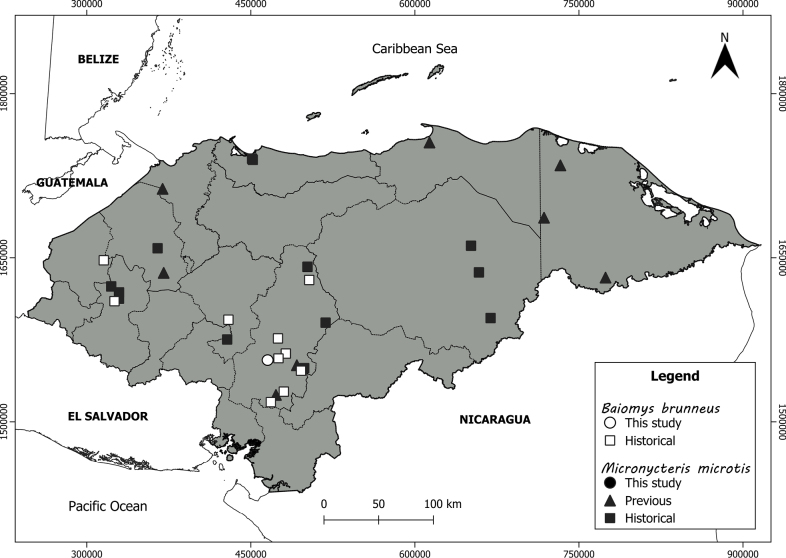
Distribution of *Baiomys brunneus* and *Micronycteris microtis* in Honduras.

Comayagua, Copán, Francisco Morazán, and Lempira (Fig. 29); 655–1435 m a.s.l.

####### Additional notes.

Individuals of this species have been identified as *Baiomys grisescens Goldman, 1932* and are sometimes confused with *M. musculus*. The subspecies *B. mexicanus* grisescens Goldman, 1932, was formerly referred to as *B. musculus* (Merriam, 1892) and described from Comayagüela, Francisco Morazán. It has been elevated to a distinct species (Amman and Bradley 2004; Hernández-Canchola and León-Paniagua 2021). *B. brunneus* ranges from Guerrero, Mexico, south through Central America (Álvarez-Castañeda 2024). Global conservation status is LC and DD in Honduras.

##### *Casiomys* Voss, 2024

Phylogenetic analyses by Weksler (2006) suggested ten genera of *Oryzomys* Baird, 1857 *sensu lato*. However, they did not provide a generic name for the Alfaroi group” including species distributed in North and Central America. Almendra et al. (2018) support monophyly of the Alfaroi group, providing evidence of the divergence from *Handleyomys* Voss, Gómez-Laverde & Pacheco, 2002 sensu stricto. Recently, Voss (2024) assigned the genus *Casiomys* to the Alfaroi group of the *Oryzomyssensu lato*. The species included in the Alfaroi group are: *Casiomys alfaroi* (J. A. Allen, 1891), *Casiomys chapmani* Thomas, 1898, *Casiomys guerrerensis* Goldman, 1915, *Casiomys melanotis* Thomas, 1893, *Casiomys rhabdops* Merriam, 1901, *Casiomys rostratus* Merriam, 1901, and *Casiomys saturatior* Merriam, 1901.

###### 35. Casiomys alfaroi

Taxon classificationAnimaliaRodentiaCricetidae

(J. A. Allen, 1891)

E09BD35C-4E6E-59A5-96C1-6BEAC77713D9

####### Natural history.

There is no information regarding this species’ ecology or natural history in Honduras.

####### Distribution.

**Figure 30.**
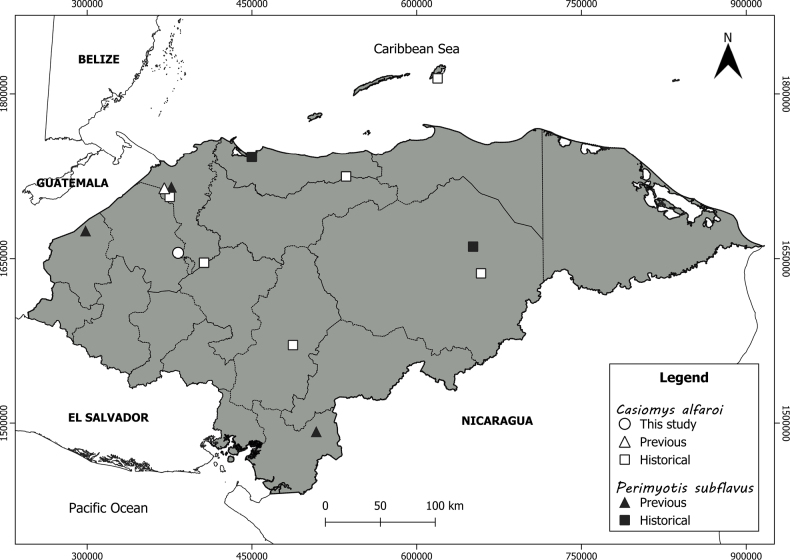
Distribution of *Casiomys alfaroi* and *Perimyotis subflavus* in Honduras.

Atlántida, Comayagua, Cortés, Francisco Morazán, Islas de la Bahía, Olancho, and **Santa Bárbara** (Fig. 30); 0–1960 m a.s.l.

####### Additional notes.

Historical records were not included because it cannot be determined if they represent *Handleyomys alfaroi* (Weksler, Percequillo & Voss, 2006) or *Handleyomys saturatior* (Weksler, Percequillo & Voss, 2006). Many historical records of this species referred to it as *Oryzomys alfaroi* (J. A. Allen, 1891). Molecular evidence supporting this species’ presence in the country comes from sequences of mitochondrial and nuclear genes (Almendra et al. 2018). Global conservation status is LC and NE in Honduras, referring to *H. alfaroi*.

###### 36. Casiomys rostratus

Taxon classificationAnimaliaRodentiaCricetidae

(Merriam, 1901)

2EF807C9-33D5-5A44-8983-6D29295F9C3B

####### Natural history.

There is no information regarding this species’ ecology or natural history in Honduras.

####### Distribution.

**Figure 31.**
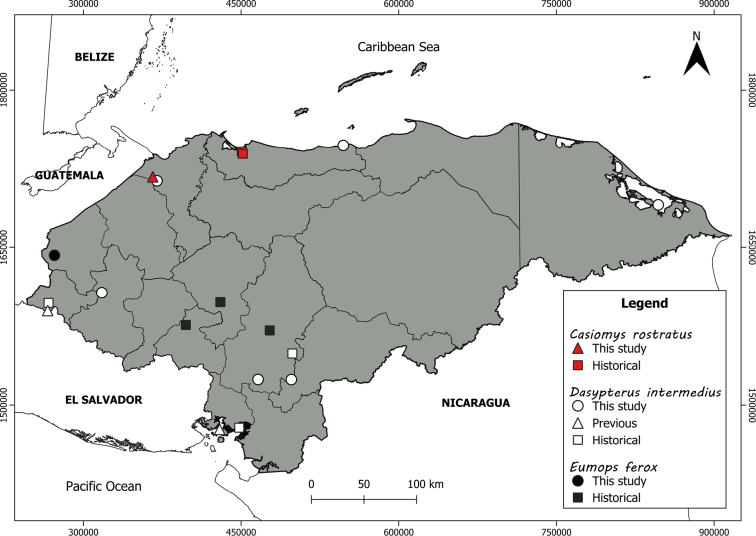
Distribution of *Casiomys rostratus*, *Eumops ferox*, and *Dasypterus intermedius* in Honduras.

Known only from Atlántida and Cortés (Fig. 31); 0–1500 m a.s.l.

####### Additional notes.

The species has also referred to it as *Oryzomys rostratus* Merriam, 1901. Almendra et al. (2018) support monophyly of the Alfaroi group and assign phylogroups based on the distribution of the specimens examined. A phylogroup *Handleyomys rostratus* (Weksler, Percequillo & Voss, 2006) including Clade III is restricted to Chortís volcanic Front and the Honduras borderlands or Chortís highlands (Almendra et al. 2018). This phylogroup corresponds to the subspecies *Oryzomys rostratus salvadorensis* Felton, 1958, confirmed based on molecular data (four mitochondrial and eight nuclear loci), originally included within *O. rostratus* (Almendra et al. 2018). This subspecies exhibits significant ecological niche differentiation compared to the other *H. rostratus* lineages, supporting its potential recognition as a separate species (Almendra et al. 2018). Global conservation status is LC and DD in Honduras.

###### 37. Casiomys saturatior

Taxon classificationAnimaliaRodentiaCricetidae

(Merriam, 1901)

58A2293C-0F3E-5D6A-A87D-E8FDFBBF6CF5

####### Natural history.

There is no information regarding this species’ ecology or natural history in Honduras.

####### Distribution.

**Figure 32.**
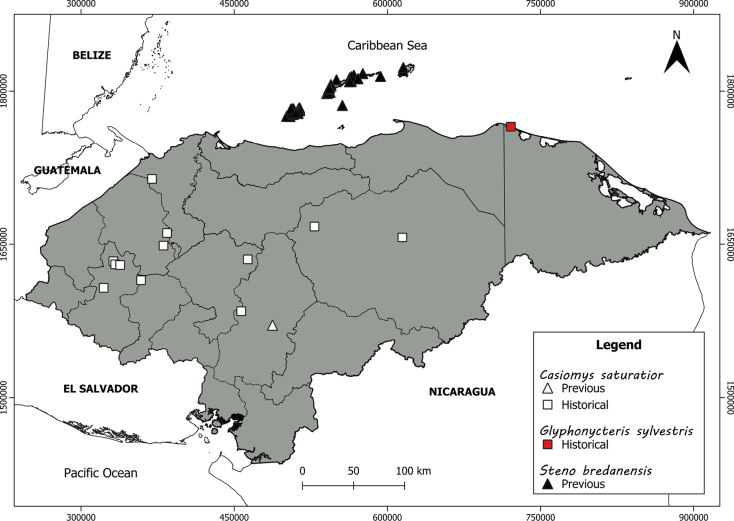
Distribution of *Casiomys saturatior*, *Glyphonycteris sylvestris*, and *Steno bredanensis* in Honduras.

Comayagua, Cortés, Francisco Morazán, Intibucá, Lempira, Olancho, and Santa Bárbara (Fig. 32); 1380–2125 m a.s.l.

####### Additional notes.

Sequences of mitochondrial and nuclear genes provide evidence of this species’ presence in the country (Almendra et al. 2018). Global conservation status is LC and DD in Honduras, both making reference to *Handleyomys saturatior* (Weksler, Percequillo & Voss, 2006).

##### *Melanomys* Thomas, 1902

###### 38. Melanomys chrysomelas

Taxon classificationAnimaliaRodentiaCricetidae

(J. A. Allen, 1897)

AE318DAA-A983-5002-9F33-2B79F4866A84

####### Natural history.

They have been captured near streams using tuna and oats as bait Medina-Fitoria and Turcios-Casco (2019).

####### Distribution.

**Figure 33.**
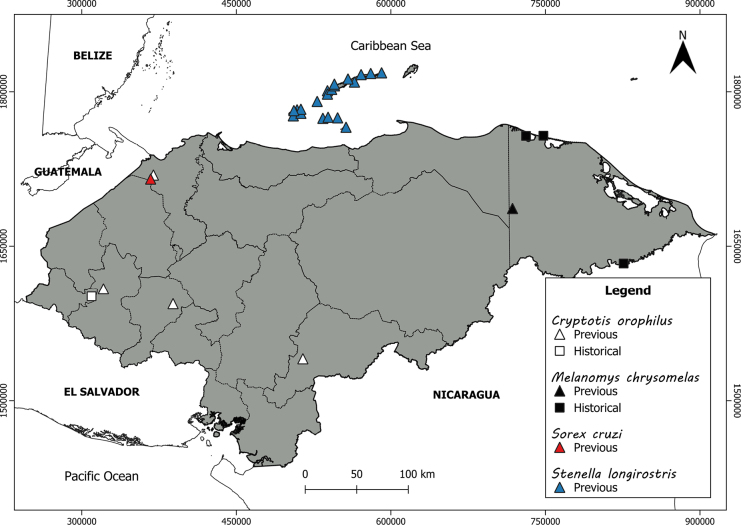
Distribution of *Cryptotis orophilus*, *Melanomys chrysomelas*, *Sorex cruzi*, and *Stenella longirostris* in Honduras.

Only known from Gracias a Dios (Fig. 33); 0–240 m a.s.l.

####### Additional notes.

Historical records refer to this species as *Melanomys caliginosus* (Tomes, 1860). Global conservation status is NE and DD in Honduras, making reference to *M. caliginosus*.

##### *Neotoma* Say & Ord, 1825

###### 39. Neotoma chrysomelas

Taxon classificationAnimaliaRodentiaCricetidae

J. A. Allen, 1908

B701D45D-C7B8-5AE4-884E-AB1EA3A27E5A

####### Natural history.

There is no information on this species’ natural history or ecology within the country.

####### Distribution.

**Figure 34.**
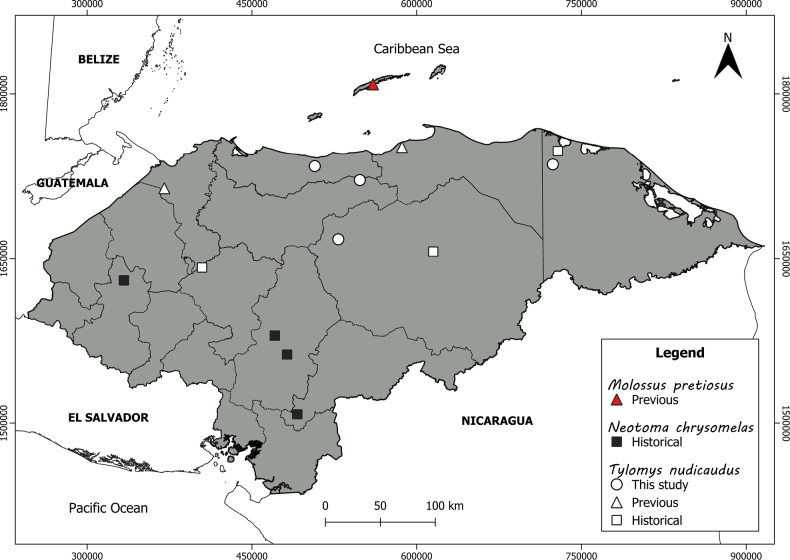
Distribution of *Molossus pretiosus*, *Neotoma chrysomelas*, and *Tylomys nudicaudus* in Honduras.

El Paraíso, Francisco Morazán, and Lempira (Fig. 34); 620–1435 m a.s.l.

####### Additional notes.

It is a regional endemic in Honduras and Nicaragua. No additional specimens have been collected since 1984. As this is an uncommon species and co-endemic with other species in the genus, a regional reassessment is warranted. Global conservation status is LC and DD in Honduras.

###### 40. Neotoma ferruginea

Taxon classificationAnimaliaRodentiaCricetidae

Tomes, 1862

66DD240E-5F29-5BF5-AF7D-1BDE79505D3B

####### Natural history.

There is no information on this species’ natural history or ecology within the country.

####### Distribution.

**Figure 35.**
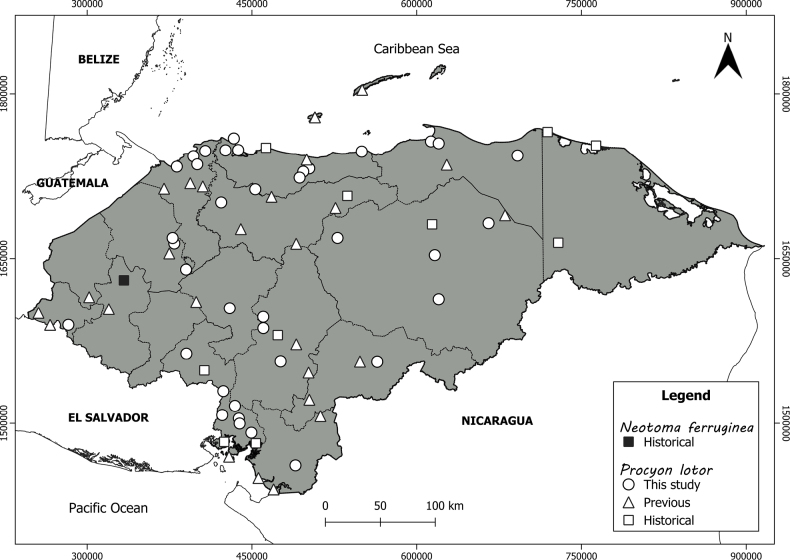
Distribution of *Neotoma ferruginea* and *Procyon lotor* in Honduras.

Lempira (Fig. 35); 2080–2130 m a.s.l., based on a single specimen collected in 1938 (Goodwin 1942a). However, Matson et al. (2016) reported that an individual was captured by UNAH staff from Celaque National Park (Campamento Don Tomás).

####### Additional notes.

Individuals of *Neotoma* in Honduras have previously been identified as subspecies *N. mexicana solitaria* Hall, 1981, or as *N. chrysomelas*. *Neotoma mexicana* Baird, 1855 is currently recognized as distributed from Arizona, Utah, and Colorado (US) through Oaxaca, Mexico, excluding Honduras (Álvarez-Castañeda 2024). Based on molecular evidence from Guatemalan populations, Ordóñez-Garza et al. (2014) indicated that *Neotoma mexicana ferruginea* Hall, 1981 is a distinct species, *Neotoma ferruginea* Tomes, 1862. This species is distributed in the highlands from Oaxaca and Chiapas south through Central America (Álvarez-Castañeda 2024). The species *N. chrysomelas* was described from a specimen collected in Nicaragua and was reported to differ from *N. ferruginea* by its larger size, relatively broader skull, and longer nasals, although the two species share similar coloration (Allen 1908). Global conservation status is NE and DD in Honduras, making reference to *N. mexicana*.

##### *Nyctomys* (Saussure, 1860)

###### 41. Nyctomys sumichrasti

Taxon classificationAnimaliaRodentiaCricetidae

(Saussure, 1860)

C89D00CA-6E71-52E6-807C-ACA0C7763C6B

####### Natural history.

Based on cytochrome b gene sequences (Corley et al. 2011), the subspecies postulated to occur here is *Nyctomys sumichrasti florencei* Goldman, 1937. They usually forage on fig, mango, and *Cecropia* trees up to 5 meters tall. Specimens from Francisco Morazán had a karyotype of 2N = 50, FN = 52 (see Bradley and Ensink 1987). López and Turcios-Casco (2025) first reported false autotomy in the species in central Honduras.

####### Distribution.

**Figure 36.**
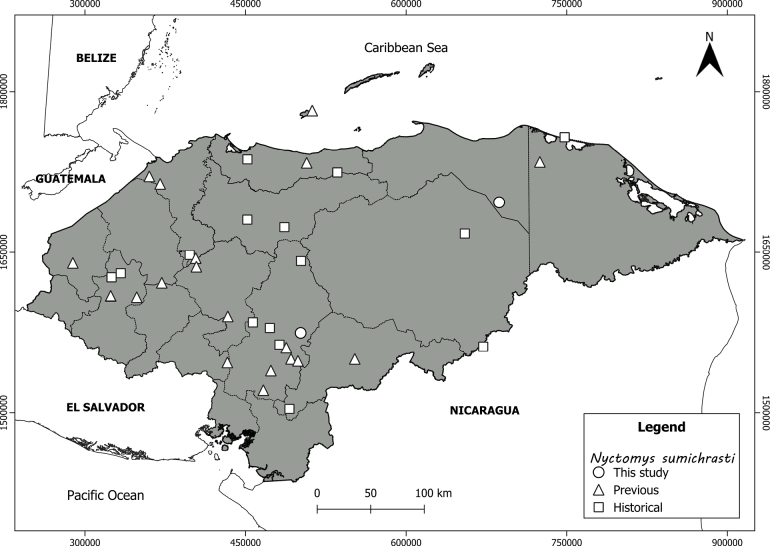
Distribution of *Nyctomys sumichrasti* in Honduras.

Atlántida, **Colón**, Comayagua, Copán, Cortés, El Paraíso, Francisco Morazán, Gracias a Dios, Islas de la Bahía, La Paz, Lempira, Olancho, Santa Bárbara, and Yoro (Fig. 36); 0–1980 m a.s.l. Global conservation status is LC and NE in Honduras.

##### *Oligoryzomys* Bangs, 1900

###### 42. Oligoryzomys fulvescens

Taxon classificationAnimaliaRodentiaCricetidae

(Saussure, 1860)

C55DE518-60C6-595D-BDA4-CF88EAF258CE

####### Natural history.

There is no information regarding this species’ ecology or natural history in Honduras.

####### Distribution.

Atlántida, Comayagua, Cortés, El Paraíso, La Paz, Francisco Morazán, Gracias a Dios, and Olancho (Fig. 11); 0–1450 m a.s.l.

####### Additional notes.

Historical specimens are referred to as *Oligoryzomys flavescens* (Waterhouse, 1837). Global conservation status is LC and NE in Honduras.

##### *Oryzomys* Baird, 1857

###### 43. Oryzomys couesi

Taxon classificationAnimaliaRodentiaCricetidae

(Alston, 1877)

A84A639F-ACB1-5F8D-AB84-41827E9E8A3E

####### Natural history.

It has been captured near artificial lagoons within coffee plantations. Specimens from Olancho had a karyotype of 2N = 56, FN = 60 (see Bradley and Ensink 1987). Apanaskevich and Bermúdez (2013) reported the occurrence of *Dermacentor panamensis* Apanaskevich & Bermúdez, 2013 and (Acari: Ixodidae) on *O. couesi* (as *Oryzomys* sp.) in Olancho.

####### Distribution.

**Figure 37.**
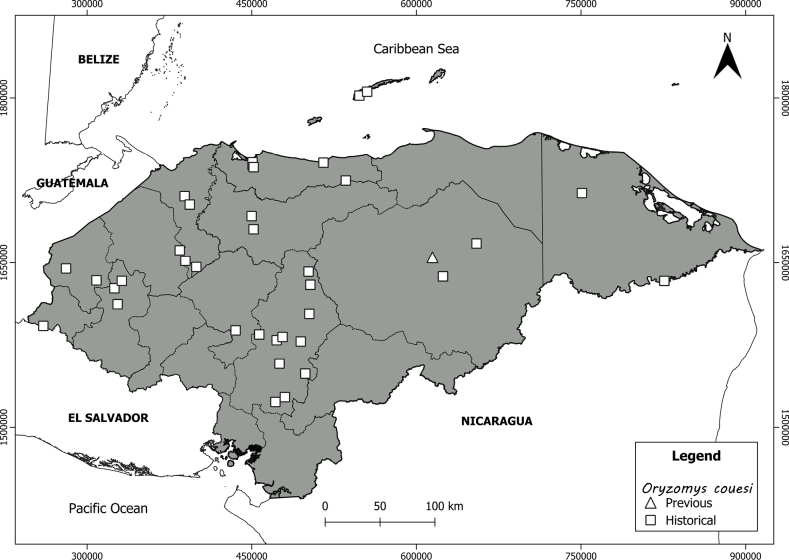
Distribution of *Oryzomys couesi* in Honduras.

Atlántida, Comayagua, Copán, Cortés, Francisco Morazán, Gracias a Dios, Islas de la Bahía, Lempira, Ocotepeque, Olancho, Santa Bárbara, and Yoro (Fig. 37); 0–2030 m a.s.l.

####### Additional notes.

Historically, some records have been referred to as *Oryzomys palustris* (Harlan, 1837). However, the current distribution for *O. palustris* is NW of the US (Pardiñas et al. 2017). Thomas (1898) indicated that several other species of *Oryzomys* may occur in Honduras; however, only *O. couesi* currently is verified from Honduras (Pardiñas et al. 2017). Other records identified as either *Oryzomys melanotis* Thomas, 1893, or *O. rhabdops* require verification and are currently assigned to a different genus (Almendra et al. 2018; Voss 2024). Global conservation is LC and NE in Honduras.

##### *Ototylomys* Merriam, 1901

###### 44. Ototylomys phyllotis

Taxon classificationAnimaliaRodentiaCricetidae

Merriam, 1901

BF7CD1D2-39D3-5D5F-90A6-6B77F5A44CCB

####### Natural history.

Gómez-Corea et al. (2019) reported a larva of the rodent bot fly *Cuterebra* Clark, 1815 (Diptera) parasitizing an individual from Colón. López and Turcios-Casco (2025) reported, for the first time, false autotomy in the species in central Honduras. We have captured individuals in small pineapple plantations and seasonal water bodies.

####### Distribution.

**Figure 38.**
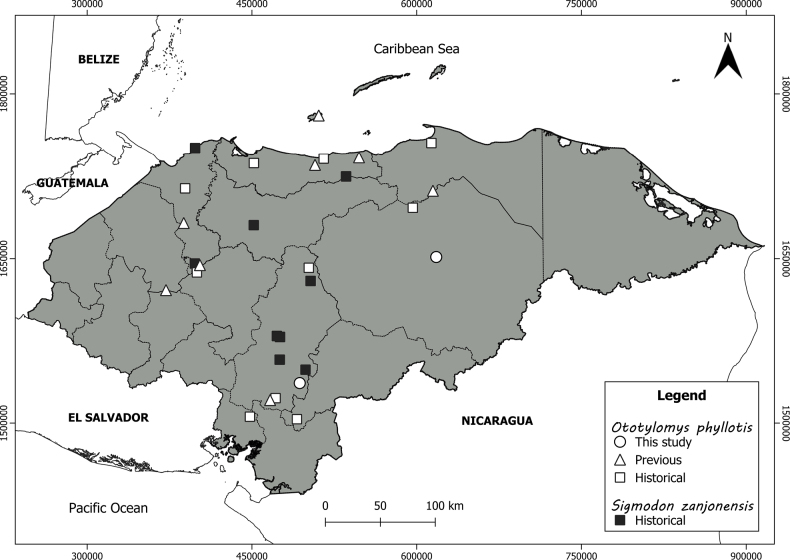
Distribution of *Ototylomys phyllotis* and *Sigmodon zanjonensis* in Honduras.

Atlántida, Colón, Comayagua, Cortés, El Paraíso, Francisco Morazán, Islas de la Bahía, Olancho, Santa Bárbara, and Valle (Fig. 38); 260–1600 m a.s.l.

####### Additional notes.

In some rural or semi-urban areas, they are killed because they may be mistaken for *R. norvegicus* and considered pests. Global conservation status is LC and NE in Honduras.

##### *Peromyscus* Gloger, 1841

Verifying which species of *Peromyscus* occur in the country remains problematic. We do not include the records from Cobo-Simón et al. (2018) as these referred to the *Peromyscus mexicanus* (Saussure, 1860) complex, as defined by Pérez-Consuegra and Vásquez Domínguez (2015, 2016), and were not identified to the species level.

Brown (2018) reported a Honduran milk snake, *Lampropeltis abnorma* (Bocourt, 1886), feeding on a *P. mexicanus* in northwestern Honduras. Acosta (2011) reported the presence of the flea *Strepsylla dalmati* Traub & Barrera, 1955 on *P. mexicanus* from Naranjo, Parque Nacional Celaque (Lempira). Given that several species occur in Lempira, it is uncertain which species was the host. The flea species *S. dalmati* was reported by Acosta (2011) from a *Peromyscus aztecus* (Saussure, 1860), which was reported by Acosta (2011) from the National Park La Tigra (Francisco Morazán). We agree with Kilpatrick et al. (2021) and López et al. (2024) that specimens identified as *P. aztecus* from Francisco Morazán (Acosta 2011) and Olancho (Matson et al. 2016) require careful review to verify species identity. The hosts referred by Apanaskevich and Bermúdez (2013) of the tick *Dermacentor panamensis* Apanaskevich & Bermúdez, 2013 (Ixodidae), referred by Apanaskevich and Bermúdez (2013) as *P. aztecus* and *P. mexicanus* from Agalta (Olancho) remain uncertain. Additionally, von Lehmann and Schaefer (1979) presented information on the sperm morphology and comparative cytochemistry of individuals of *Peromyscus hondurensis* Goodwin, 1941, and *Peromyscus guatemalensis tropicalis* Goodwin, 1932 from Honduras was provided by von Lehmann and Schaefer (1979). Therefore, the specimens should be carefully reviewed and verified. Bradley and Ensink (1987) describe the karyotype (2N = 48, FN = 56) from a specimen identified as *Peromyscus mexicanus saxatilis* Merriam, 1898 from Francisco Morazán. Based on López et al. (2024), we suggest that the species of *Peromyscus* that occur here are *Peromyscus beatae* Thomas, 1903, *Peromyscus nicaraguae* J. A. Allen, 1908, *Peromyscus cordillerae* Dickey, 1928, *Peromyscus salvadorensis* Dickey, 1928, and *Peromyscus stirtoni* Dickey, 1928. We accept the distribution proposed by López et al. (2024) until further evidence or corroboration from historical specimens becomes available. López and Turcios-Casco (2025) confirmed false autotomy for the genus.

###### 45. Peromyscus beatae

Taxon classificationAnimaliaRodentiaCricetidae

Thomas, 1903

F779844A-9D52-56AF-8983-F6B9F35ED9DB

####### Natural history.

Specimens from Francisco Morazán have a karyotype of 2N = 48, and FN = 52 and 54 (Bradley and Ensink 1987).

####### Distribution.

**Figure 39.**
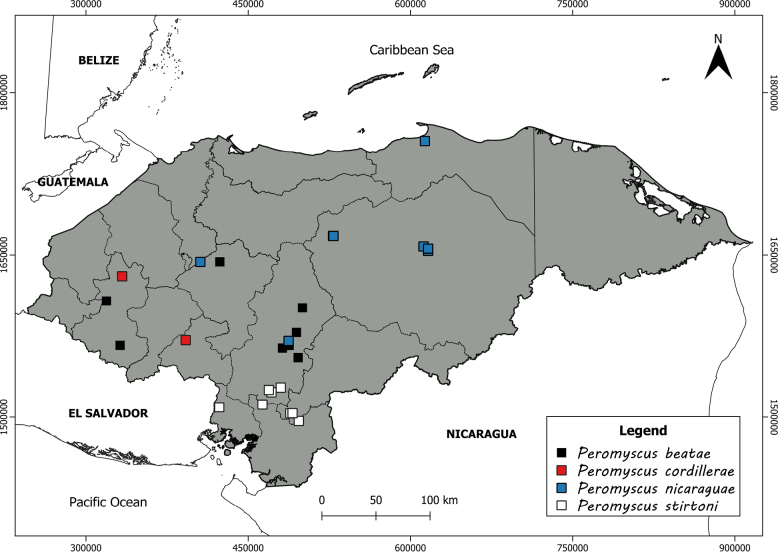
Distribution of *Peromyscus beatae*, *P. cordillerae*, *P. nicaraguae*, and *P. stirtoni* in Honduras. Note that all the records are considered historical.

**Francisco Morazán**, **Comayagua**, and **Lempira** (Fig. 39); 300–2000 m a.s.l.

####### Additional notes.

Recent studies indicate that *P. beatae is* genetically distinct from *Peromyscus boylii* (Baird, 1855) and most likely represents a cryptic species and *Peromyscus beatae sacarensis* Thomas, 1903. Considering that *P. beatae* is recognized as different from *P. boylii*, we consider the records of Goodwin (1942a) identified as *P. boylii* to be *P. beatae*. Global conservation status is LC and DD in Honduras.

###### 46. Peromyscus cordillerae

Taxon classificationAnimaliaRodentiaCricetidae

Dickey, 1928

A98DAF35-547C-50D9-96FE-E15BFD08C80A

####### Natural history.

There is no information regarding this species’ ecology or natural history in Honduras.

####### Distribution.

La Paz and Lempira (Fig. 39); 1000–2000 m a.s.l.

####### Additional notes.

Dickey (1928) described the subspecies *Peromyscus boylii cordillerae* Dickey, 1928, comparing and distinguishing it from members of the *P. boylii* species group, and indicating that it is similar to *Peromyscus oaxacensis* Merriam, 1898 (member of the *P. aztecus* species group). Subsequently, Goodwin (1941) described *P. hondurensis* from individuals collected in Muya, 5 mi N Chinacla, Department La Paz, and individuals from “Gracia, Cerro Pucca”, Department of Lempira. Musser (1969) indicated that *P. oaxacensis* extended southward into Honduras, and *P. oaxacensis cordillerae* was considered a synonym by Pardiñas et al. (2017). However, in their more recent taxonomic review, Kilpatrick et al. (2021) noted that *P. oaxacensis*, northwest of the Isthmus of Tehuantepec, should be considered *Peromyscus aztecus oaxacensis* Kilpatrick, N. Pradhan, & R. W. Norris, 2021, the equivalent to *P. oaxacensis* by Pardiñas et al. (2017), now limited to the type locality in Oaxaca, Mexico. While the species *P. a. oaxacensis* distributed southeast of the Isthmus of Tehuantepec should be recognized as two subspecies of *P. cordillerae*, *P. c.cordillerae and P. c. hondurensis* (Kilpatrick et al. 2021). Since *Peromyscus cordillerae* Dickey, 1928 has priority, Pardiñas et al. (2017) classify this species as *P. oaxacensis cordillerae* Dickey, 1928. Kilpatrick et al. (2021) suggested that the populations located south of the Isthmus of Tehuantepec represent a distinct species. Additional specimens are required to confirm the species’ distribution. Global and national conservation status is NE.

###### 47. Peromyscus nicaraguae

Taxon classificationAnimaliaRodentiaCricetidae

J. A. Allen, 1908

CA390493-8B61-52C5-AA46-8B1F4B1CF1F5

####### Natural history.

There is no information regarding this species’ ecology or natural history in Honduras.

####### Distribution.

Colón, Comayagua, Francisco Morazán, and Olancho (Fig. 39); 500–2100 m a.s.l.

####### Additional notes.

Hall (1981) stated that *P. m. saxatilis* is a synonym of *P. nicaraguae*. We therefore assign the *P. m. saxatilis* records of Goodwin (1942a) to *P. nicaraguae*. More specimens are needed to confirm the species’ distribution. Global and national conservation status is NE.

###### 48. Peromyscus salvadorensis

Taxon classificationAnimaliaRodentiaCricetidae

Dickey, 1928

70A1E6D0-B8A1-5C5E-81D1-0D7119B146ED

####### Natural history.

There is no information regarding this species’ ecology or natural history in Honduras.

####### Distribution.

Lempira (Fig. 28); 1430–2850 m a.s.l. However, there are two clades within *P. salvadorensissensu lato* that are distinct from *P. salvadorensissensu stricto* (see Pérez-Consuegra and Vásquez-Domínguez 2016; Hernández-Canchola et al. 2022).

####### Additional notes.

The species *P. salvadorensis* was originally described by Dickey (1928). Hall (1981) considered *P. salvadorensis* to be a synonym of *P. guatemalensis tropicalis* Goodwin, 1932. Therefore, we include Goodwin (1942a) records of *P. g. tropicalis* as *P. salvadorensis*. Recently, combined genetic sequencing and morphometric analyses indicate that *P. salvadorensis* is distributed at mid- to high elevations (1000–2900 m) in eastern Guatemala, El Salvador, and southern Honduras. It can be differentiated from *P. mexicanus* by skull characteristics (Pérez-Consuegra et al. 2016). Records of *P. salvadorensis*, based on distribution modeling, indicated that populations of these species should be restricted in Honduras to the rainy lowlands to the west of the country’s central depression (Pérez-Consuegra et al. 2023). More specimens are needed to confirm the species’ distribution. Global and national conservation status is NE.

###### 49. Peromyscus stirtoni

Taxon classificationAnimaliaRodentiaCricetidae

Dickey, 1928

AC3E46A8-6513-5AD9-91CF-22E3896F4D7E

####### Natural history.

This species is distributed across the country’s southern region and is likely present in the southwestern region as well.

####### Distribution.

Choluteca, El Paraíso, Francisco Morazán, and Valle (Fig. 39); 500–1000 m a.s.l.

####### Additional notes.

Recent expeditions (2025) in southern Honduras did not find this species. Global conservation status is LC and DD in Honduras.

##### *Reithrodontomys* Giglioli, 1874

Hooper (1952) reviewed the taxonomy and distribution of *Reithrodontomys* in Central America. Young and Jones (1984) suggested that *Reithrodontomys gracilis* J. A. Allen & Chapman, 1897 was expected to occur here; however, we have yet to find supporting evidence confirming its presence.

###### 50. Reithrodontomys fulvescens

Taxon classificationAnimaliaRodentiaCricetidae

J. A. Allen, 1894

B34E851C-4366-5E6D-9983-FA409BDFA746

####### Natural history.

There is no information regarding this species’ ecology or natural history in Honduras.

####### Distribution.

Cortés, Francisco Morazán, and Olancho (Fig. 25); 260–960 m a.s.l.

####### Additional notes.

Global conservation status is LC and DD in Honduras.

###### 51. Reithrodontomys mexicanus

Taxon classificationAnimaliaRodentiaCricetidae

(Saussure, 1860)

159E8BE0-D065-5AE9-98DE-F250570A3A81

####### Natural history.

The flea *Strepsylla dalmati* Traub & Barrera, 1955 has been reported from individuals from Naranjo, National Park Celaque (Lempira) (Acosta 2011).

####### Distribution.

**Figure 40.**
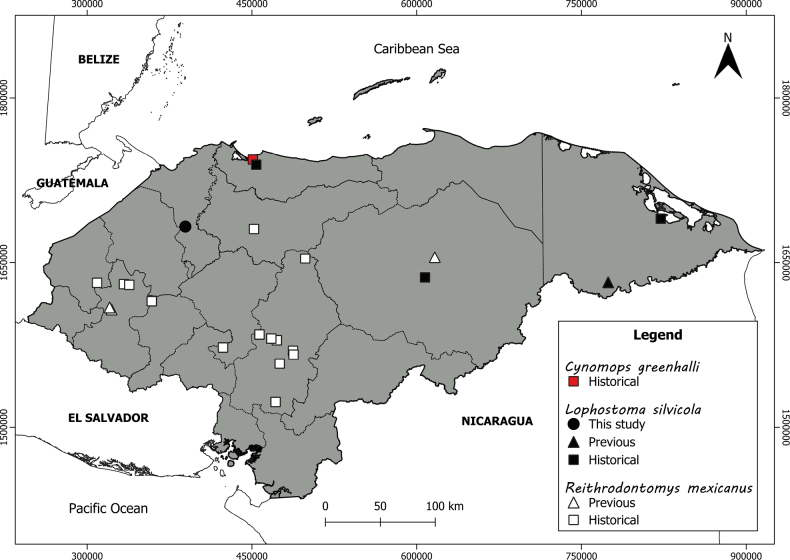
Distribution of *Cynomops greenhalli*, *Lophostoma silvicola*, and *Reithrodontomys mexicanus* in Honduras.

Comayagua, Copán, Francisco Morazán, Intibucá, Lempira, Olancho, and Yoro (Fig. 40); 840–2560.

####### Additional notes.

We recognize two subspecies occurring in Honduras: *Reithrodontomys mexicanus ocotepequensis*Goodwin 1937, distributed in western Honduras, at least in Copán, Intibucá, and Lempira, and occurring sympatrically with *Reithrodontomys mexicanus lucifrons* A. H. Howell, 1932 in Lempira. The subspecies *R. m. lucifrons* is expected to occur in other regions of Honduras but has not yet been verified. Global conservation status is LC and DD in Honduras.

###### 52. Reithrodontomys australis

Taxon classificationAnimaliaRodentiaCricetidae

J. A. Allen, 1895

019A7E84-275D-5311-847F-84DA56B77995

####### Natural history.

See von Lehmann and Schaefer (1979) for comments on sperm morphology and comparative cytochemistry of individuals of this species from Honduras. Specimens from Francisco Morazán have a karyotype of 2N = 40, FN = 76 (Bradley and Ensink 1987). We have recorded this species in habitats located near heavily contaminated municipal waste disposal sites.

####### Distribution.

**Figure 41.**
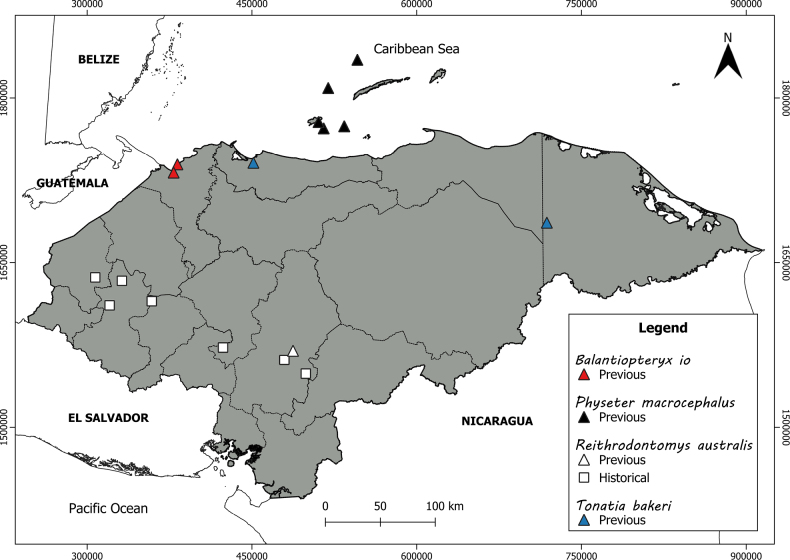
Distribution of *Balantiopteryx io*, *Physeter macrocephalus*, *Reithrodontomys australis*, and *Tonatia bakeri* in Honduras.

Comayagua, Copán, Francisco Morazán, Intibucá, and Lempira (Fig. 41); 820–2240 m a.s.l.

####### Additional notes.

*R. australis* is considered a valid species distinct from other forms of *Reithrodontomys sumichrasti* (Saussure, 1861) occurring west of the Isthmus of Tehuantepec (IT) (Arellano et al. 2023). *R. sumichrasti* clades delimitation was evaluated, and values of genetic divergence support the recognition of populations at the east and south of the IT as valid species (Arellano et al. 2023). *Reithrodontomys australis modestus* A. H. Howell, 1914 is recognized as a subspecies of the former *R. sumichrasti* and is currently acknowledged as present in the country. Molecular studies conducted by Martínez-Borrego et al. (2022) indicate that *R. sumichrasti* is restricted to the northern and western regions of the Isthmus of Tehuantepec. In contrast, *R. australis* is distributed to the south and east of the Isthmus, including Chiapas and the Central American highlands. Regarding the specific distribution of the subspecies *R. a. modestus* in Honduras, Martínez-Borrego et al. (2022) followed the work of Hall (1981).

Historically, *R. australis* has been referred to as *Reithrodontomys dorsalis underwoodi* Goodwin, 1937. The subspecies *R. d. underwoodi* was reported for Monte Verde, Department of Ocotepeque, Honduras; altitude 4,500’; collected on July 24, 1936, Goodwin (1937). However, this taxon should be considered a synonym of *Reithrodontomys australis dorsalis* A. H. Howell, 1914, based on the size and original description of the type specimen, which Goodwin (1937) characterized as smaller and differing in tail and body coloration from *R. dorsalis*. Currently, *R. australis dorsalis* is distributed in the Guatemalan highlands (Martínez-Borrego et al. 2022). Further analyses are needed to determine whether *R. australis* has one or two subspecies in Honduras. Global conservation status (reported as *R. sumichrasti*) is LC and DD in Honduras.

##### *Rheomys* Thomas, 1906

###### 53. Rheomys

Taxon classificationAnimaliaRodentiaCricetidae

sp.

F07D36DE-89CF-59D3-BAF5-4EB053A905D8

####### Natural history.

Gilroy et al. (2017) reported that a specimen collected from Cusuco National Park represented the first confirmed record for the country. Subsequently, Martin et al. (2021) noted additional specimens had been captured along high-elevation streams. Based on preliminary analyses of external phenotype and skull morphology, these individuals differed from both *Rheomys thomasi* Dickey, 1928 and *Rheomys raptor* Goldman, 1912, suggesting that they may represent a new taxon. Martin et al. (2021) did not compare the osteological material with other species distributed in Panama, *R. underwoodi*, but only with *R. raptor*, which they suggested was distributed in El Salvador; however, current records point to Panama as the only known distribution for this species (see Mammal Diversity Database (2026): https://www.mammaldiversity.org/taxon/1002501).

####### Distribution.

Only known from Cusuco National Park in Cortés (Fig. 22).

####### Additional notes.

As the species has yet to be determined, we only assign these to the genus level pending final identification. However, *R. thomasi* is considered NT in the Honduran Red List.

##### *Scotinomys* Thomas, 1913

###### 54. Scotinomys teguina

Taxon classificationAnimaliaRodentiaCricetidae

(Alston, 1877)

B367E6F6-00EF-59E8-BAA1-32D6D4FFA879

####### Natural history.

Sanborn (1935) described the subspecies *Scotinomys teguina rufoniger* Sanborn, 1935 based on individuals collected in San Pedro, Cortés department. Ectoparasites include the tick *Dermacentor panamensis* Apanaskevich & Bermúdez, 2013 (Ixodidae) from Agalta (Olancho).

####### Distribution.

**Figure 42.**
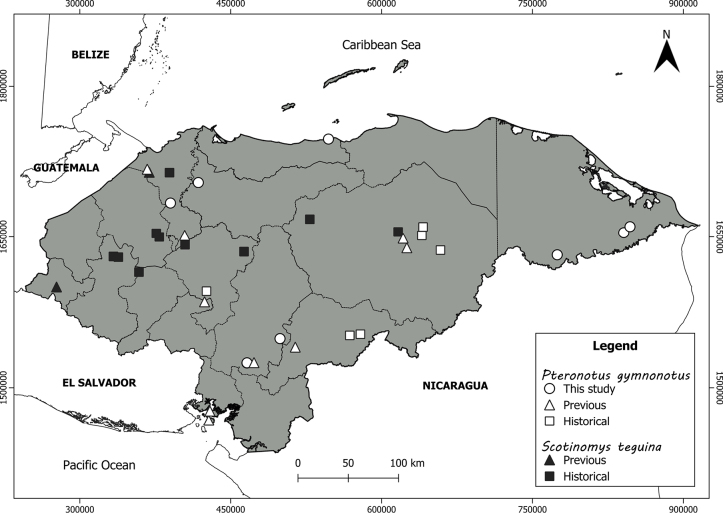
Distribution of *Pteronotus gymnonotus* and *Scotinomys teguina* in Honduras.

Comayagua, Cortés, Intibucá, Lempira, Ocotepeque, Olancho, and Santa Bárbara (Fig. 42); 110–2560 m a.s.l.

####### Additional notes.

Sanborn (1935) described individuals collected in San Pedro, Cortés department, in areas (e.g. core zones of cloud and pine forests) of its distribution that are currently affected by extensive cattle ranching, and deforestation. Global conservation status is LC and NE in Honduras.

##### *Sigmodon* Say & Ord, 1825

*Sigmodon* has only one subgenus (*Sigmodon*) in North America with two species groups: *S. fulviventer* (*Sigmodon fulviventer* J. A. Allen, 1889, *Sigmodon leucotis* Bailey, 1902, and *Sigmodon ochrognathus* Bailey, 1902); and *S. hispidus* (*Sigmodon alleni* Bailey, 1902, *Sigmodon arizonae* Mearns, 1890, *Sigmodon hirsutus* (Burmeister, 1854), *Sigmodon hispidus* Say & Ord, 1825, *Sigmodon mascotensis* J. A. Allen, 1897, and *Sigmodon toltecus* (Saussure, 1860); Álvarez-Castañeda 2024). We did not include the records from Goodwin (1942a), pending clarification of the populations that may occur here. Specimens from Islas de la Bahía should be identified and reviewed, as the species may have been accidentally introduced. Gómez-Corea et al. (2019) included specimens of *S. hispidus*; however, their records were excluded due to taxonomic uncertainty.

###### 55. Sigmodon hirsutus

Taxon classificationAnimaliaRodentiaCricetidae

(Burmeister, 1854)

BC1F814D-C9A3-54F1-AB39-32CDF60574C4

####### Natural history.

They are usually captured in sugar cane fields.

####### Distribution.

Atlántida, Colón, Cortés, El Paraíso, Francisco Morazán, Gracias a Dios, Intibucá, Islas de la Bahía, Lempira, Olancho, and Valle (Fig. 13); 0–1070 m a.s.l.

####### Additional notes.

*Sigmodon hirsutus* was previously regarded as a subspecies of *S. hispidus* and was reinstated as a distinct species by Peppers and Bradley (2000). It includes several subspecies (Henson and Bradley 2009); no morphological characteristics distinguish it from *S. hispidus* or *S. toltecus* (Peppers and Bradley 2000; Carroll and Bradley 2005). Sequences of the Cytochrome-b gene from National Park Capiro and Calentura support the distribution of this species in the country (Bradley et al. 2008). Middle American populations require revision and warrant careful examination to delimit robust, diagnosable intraspecific taxonomic units (Pardiñas et al. 2017). Global conservation status is LC and NE in Honduras.

###### 56. Sigmodon toltecus

Taxon classificationAnimaliaRodentiaCricetidae

(Saussure, 1860)

95003B08-29E8-5046-BCC0-9C2AE22B0A2D

####### Natural history.

There is no information regarding this species’ ecology or natural history in Honduras. See von Lehmann and Schaefer (1979) for comments on sperm morphology and comparative cytochemistry of individuals referred to *Sigmodon hispidus* Say & Ord, 1825 from Honduras.

####### Distribution.

Atlántida, Colón, Comayagua, Cortés, Francisco Morazán, Islas de la Bahía, Olancho, and Valle (Fig. 17); 0–1330 m a.s.l.

####### Additional notes.

*Sigmodon toltecus* was considered a subspecies of *S. hispidus* until molecular analyses documented strong genetic divergence and cladistic separation (Pardiñas et al. 2017). Molecular evidence from the Cytochrome b gene supports the presence of *S. toltecus* in the country (Bradley et al. 2008). Global and national conservation status is NE.

###### 57. Sigmodon zanjonensis

Taxon classificationAnimaliaRodentiaCricetidae

Goodwin, 1932

098AB0C2-0502-5901-988C-08C3393B6E16

####### Natural history.

They are usually captured in corn fields.

####### Distribution.

Atlántida, Cortés, Francisco Morazán, and Yoro. (Fig. 38); 0–1090 m a.s.l.

####### Additional notes.

This montane species needs clarification of its morphological, molecular, ecological, and distribution limits (Pardiñas et al. 2017). Global and national conservation status is NE.

##### *Sigmodontomys* J. A. Allen, 1897

###### 58. Sigmodontomys alfari

Taxon classificationAnimaliaRodentiaCricetidae

J. A. Allen, 1897

9B5D12DE-65CA-5422-9519-C50BA708EF6A

####### Natural history.

There is no information regarding this species’ ecology and natural history from Honduras.

####### Distribution.

**Figure 43.**
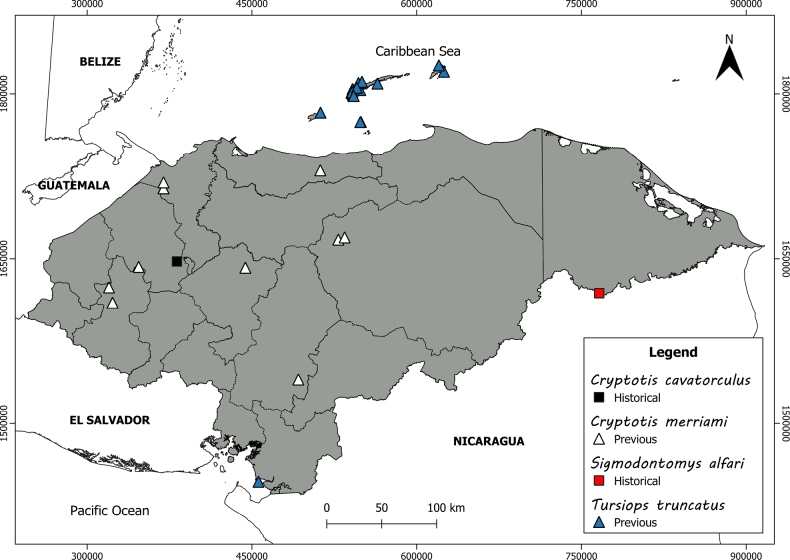
Distribution of *Cryptotis cavatorculus*, *C. merriami*, *Sigmodontomys alfari*, and *Tursiops truncatus* in Honduras.

Only known from Gracias a Dios (Fig. 43); 0–250 m a.s.l.

####### Additional notes.

Global conservation status is LC and DD in Honduras.

##### *Transandinomys* Weksler, Percequillo & Voss, 2006

###### 59. Transandinomys bolivaris

Taxon classificationAnimaliaRodentiaCricetidae

(J. A. Allen, 1901)

55882EEE-0168-5FA6-8ECC-27D4BBE98949

####### Natural history.

We have found young individuals in March and April. Individuals have been captured near streams using tuna as bait (Medina-Fitoria and Turcios-Casco 2019).

####### Distribution.

Known only from Gracias a Dios (Fig. 26); 0–250 m a.s.l.

####### Additional notes.

Global conservation status is LC and DD in Honduras.

##### *Tylomys* Peters, 1866

###### 60. Tylomys nudicaudus

Taxon classificationAnimaliaRodentiaCricetidae

(Peters, 1866)

FF13A201-3F2F-5F9A-B8D6-E87D6A4339D9

####### Natural history.

Individuals have been found in tree holes in Olancho and Gracias a Dios. A tick, *Dermacentor panamensis* Apanaskevich & Bermúdez, 2013 (Ixodidae) was reported from an individual from Agalta (Olancho).

####### Distribution.

**Atlántida**, Colón, Comayagua, Cortés, Gracias a Dios, Olancho, and **Yoro** (Fig. 34); 0–1500 m a.s.l.

####### Additional notes.

Global conservation status is LC and DD in Honduras.

#### Muridae Illiger, 1811

##### *Mus* Linnaeus, 1758

###### 61. Mus musculus

Taxon classificationAnimaliaPhyllodocidaMuridae

Linnaeus, 1758

620CDFB8-9C2C-54B4-8CBE-E0D886FC33AC

####### Natural history.

It is considered a pest nationwide. See von Lehmann and Schaefer (1979) for comments in the sperm morphology and comparative cytochemistry of individuals of this species from Honduras were described by von Lehmann and Schaefer (1979). It is sympatric with other native species within its distribution.

####### Distribution.

It is expected to be distributed throughout the country, but it has only been confirmed in Atlántida, Comayagua, Copán, Cortés, Francisco Morazán, and Valle; 0–1100 m a.s.l.

####### Additional notes.

Global conservation status is LC and NE in Honduras.

##### *Rattus* Fischer, 1803

###### 62. Rattus norvegicus

Taxon classificationAnimaliaRodentiaMuridae

(Berkenhout, 1769)

0240FFBF-2F1D-5034-AF1F-ED54548C607F

####### Natural history.

It is considered a public health pest nationwide. No known specimens are in collections, and only one photograph is presented by Espinoza Montero (2015). However, Puckett et al. (2016) have provided additional evidence of its occurrence.

####### Distribution.

It is expected to be distributed throughout the country, but has been confirmed only in Francisco Morazán, at 0–1630 m a.s.l.

####### Additional notes.

It is considered a plague. Global conservation status is LC and NE in Honduras.

###### 63. Rattus rattus

Taxon classificationAnimaliaRodentiaMuridae

(Linnaeus, 1758)

130B15F0-7B6A-5310-8F33-83DFF8C3A8A1

####### Natural history.

It is considered widespread throughout the country. Sperm morphology and comparative cytochemistry of individuals of this species from Honduras were described by von Lehmann and Schaefer (1979). It may be sympatric with *R. norvegicus* within its distribution in Honduras.

####### Distribution.

It is expected to be distributed throughout the country but confirmed only in Atlántida, Colón, Cortés, El Paraíso, Francisco Morazán, Lempira, Olancho, Valle, and Yoro; 0–1100 m a.s.l. It was introduced in the Islas de la Bahía department (Bermingham et al. 1998).

####### Additional notes.

They are known as matis or matis tara by the Miskitus in eastern Honduras, as *R. norvegicus* (Jones 1965). Global conservation status is LC and NE in Honduras.

#### Eulipotyphla Waddell, Okada & Hasegawa, 1999

For *Sorex* Linnaeus, 1758 & Cr*yptotis* Pomel, 1848, occurrence in Honduras, we agree with the species distributed in the country based on Woodman (2000, 2019), Woodman and Timm (1992), Woodman and Timm (1999), Woodman (2011), and Woodman (2015), as well as the new species published subsequently (e.g., Andino-Madrid et al. 2020). Although Woodman (2019) suggested that *Cryptotis montecristo* Woodman, 2019 is restricted to higher elevations (2150–2418 m a.s.l.), including Montecristo Trifinio National Park, there are no verified records from the country. *Cryptotis mam* Woodman, 2010, and *Cryptotis tropicalis* (Merriam, 1895) likely occur in the mountainous region shared with Guatemala. There is a series of 28 specimens in the GBIF.org (2023) referred to as *Cryptotis goldmani* (Merriam, 1895) from Celaque and Lempira, but we do not include them as the identification could not be verified.

#### Soricidae G. Fischer, 1814

##### *Cryptotis* Pomel, 1848

Individuals identified by Goodwin (1942a) under other names (e.g., *Blarina micrura* (J. A. Allen, 1893)) were not included because the species was not clearly identified and requires careful review (see Materials and methods). Woodman and Croft (2026) described a new endemic species from Copán department, *Cryptotis copanensis* Woodman & Croft, 2026, and although recent evidence suggests that may still persist in Honduras, its current existence has not yet been definitively confirmed. Therefore, we adopt a conservative approach and do not include the species in the checklist of extant Honduran mammals. Instead, we recognize its occurrence based on osteological remains and discuss the available evidence supporting its possible persistence. Given its potential conservation significance, *C. copanensis* should be regarded as a priority for future field surveys aimed at confirming its continued presence, distribution, and conservation status in Honduras

###### 64. Cryptotis cavatorculus

Taxon classificationAnimaliaAnuraSoricidae

Woodman, 2015

59CC4FB1-6B84-5B31-8AB0-B909B020D31D

####### Natural history.

There have been no further records since the original capture in 1995 at Montaña de Santa Bárbara (Woodman 2015). It is likely restricted to higher elevations throughout the mountains. No reproductive data is available.

####### Distribution.

Endemic to Montaña de Santa Bárbara National Park, Santa Bárbara (Fig. 43); 1900 m a.s.l.

####### Additional notes.

Within the Santa Bárbara National Park, it is under threat due to extensive cattle ranching and deforestation. Global and national conservation status is DD.

###### 65. Cryptotis celaque

Taxon classificationAnimaliaAnuraSoricidae

Woodman, 2015

16035E54-63C9-570A-94D5-F098CBFD9F39

####### Natural history.

Females captured in January, February, and October were not reproductively active (Woodman 2015).

####### Distribution.

**Figure 44.**
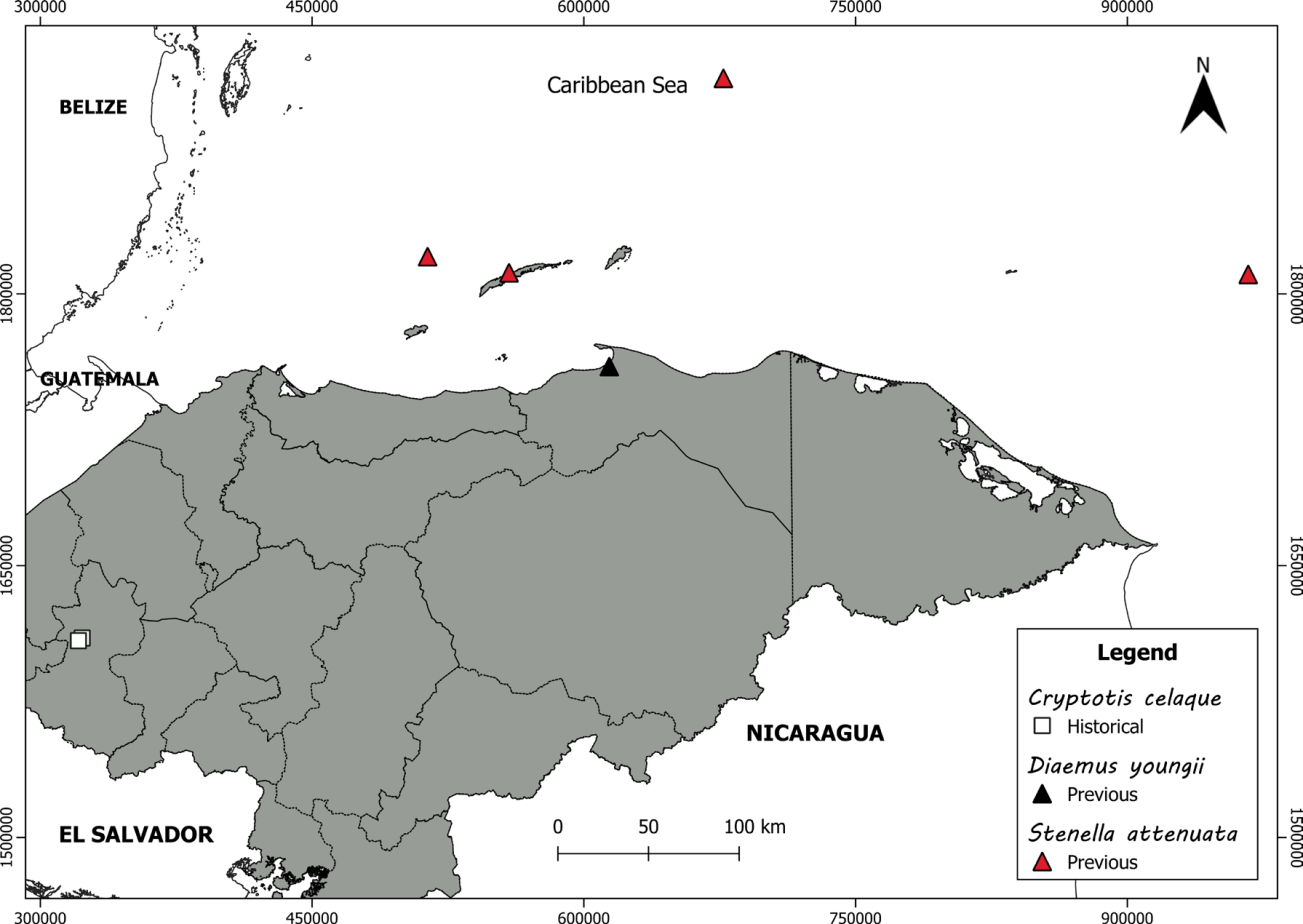
Distribution of *Cryptotis celaque*, *Diaemus youngii*, and *Stenella attenuata* in Honduras.

Endemic to the Celaque National Park, Lempira, which is the type locality (Fig. 44); 1430–2560 m a.s.l.

####### Additional notes.

As it is sympatric with *Cryptotis merriami* Choate, 1970, and *Cryptotis orophilus* (J. A. Allen, 1895), we did not include iNaturalist records. Global conservation status is NE and CR in Honduras.

###### 66. Cryptotis hondurensis

Taxon classificationAnimaliaAnuraSoricidae

Woodman & Timm, 1992

CBEE72A1-66EF-5D1D-9A24-D7D16AC12301

####### Natural history.

This endemic appears to be restricted to pine (*Pinus pseudostrobus* Lindl.) and mixed pine-oak forests in Francisco Morazán. It is suspected to occur in Guatemala, El Salvador, and Nicaragua (Woodman and Timm 1992), but no specimens have been reported. It is only known from the Uyuca Biological Reserve and La Tigra National Park in Honduras.

####### Distribution.

**Figure 45.**
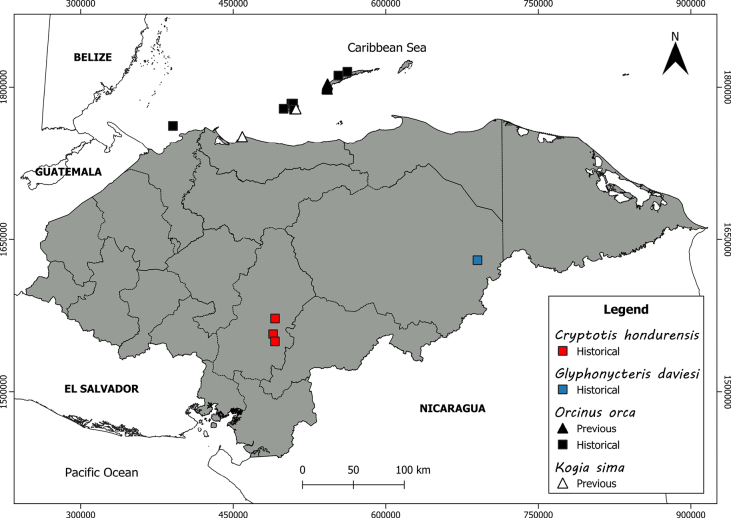
Distribution of *Cryptotis hondurensis*, *Glyphonycteris daviesi*, *Kogia sima*, and *Orcinus orca* in Honduras.

Endemic to central Honduras in the department of Francisco Morazán (Fig. 45); 1900 m a.s.l.

####### Additional notes.

Feral and domestic cats represent a significant threat to this species. Global conservation status is DD and CR in Honduras.

###### 67. Cryptotis magnimanus

Taxon classificationAnimaliaAnuraSoricidae

(Woodman & Timm, 1999)

9B126BB8-16FE-51CE-BE72-3D7D781BCCAF

####### Natural history.

Since the original description of the type from Cordillera of Montecillos in Comayagua (Woodman and Timm 1999; Woodman 2015), no additional records have been reported.

####### Distribution.

**Figure 46.**
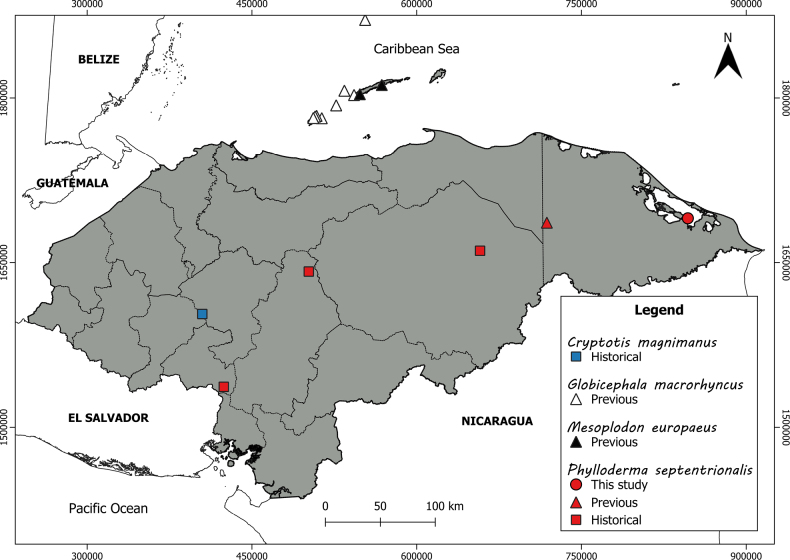
Distribution of *Cryptotis magnimanus*, *Globicephala macrorhynchus*, *Mesoplodon europaeus*, and *Phylloderma septentrionalis* in Honduras.

Endemic to the Cordillera of Montecillos in Comayagua (Fig. 46); 1730 m a.s.l.

####### Additional notes.

Historical records referred to this species as *Cryptotis goodwini magnimana* Woodman & Timm, 1999. Global conservation status is NE and CR in Honduras.

###### 68. Cryptotis mccarthyi

Taxon classificationAnimaliaAnuraSoricidae

Woodman, 2015

0F7FE29E-A285-576F-87D5-AFB0AC9DDF85

####### Natural history.

A single lactating female was captured in December, but no other reproductive individuals were recorded in December or February (Woodman 2015).

####### Distribution.

Endemic to Cortés (Fig. 21); 1500–1620 m a.s.l.

####### Additional notes.

It appears to be endemic to Sierra de Omoa, and no additional records are known. Global conservation status is DD and CR in Honduras.

###### 69. Cryptotis merriami

Taxon classificationAnimaliaAnuraSoricidae

Choate, 1970

51C06222-112B-58FF-AB14-EC06C3D25C5F

####### Natural history.

The northern Central American population appears to be disjunct (Matson and Woodman 2019; Woodman 2019). It may be sympatric with the other endemic species in Honduras.

####### Distribution.

Comayagua, Cortés, El Paraíso, Francisco Morazán, Lempira, Olancho, and Santa Bárbara (Fig. 43); 900–2560 m a.s.l.

####### Additional notes.

Although it is the most common shrew in Honduras, little information is available. Historical specimens are referred to as *Cryptotis goodwini* Jackson, 1933. Global conservation status is LC and DD in Honduras.

###### 70. Cryptotis orophilus

Taxon classificationAnimaliaAnuraSoricidae

(J. A. Allen, 1895)

95A061D8-A2B2-578E-94A2-2481D4B6D198

####### Natural history.

There is no ecological information, even though it is the most common shrew species in Honduras. It may be sympatric with the other endemic species in Honduras.

####### Distribution.

Cortés, El Paraíso, Intibucá, Lempira, and Ocotepeque (Fig. 33); 900–2000 m a.s.l.

####### Additional notes.

Historical specimens may be referred to *Cryptotis parva* (Say, 1823) (see Woodman 2015, 2019). Global conservation status is NE and DD in Honduras.

##### *Sorex* Linnaeus, 1758

###### 71. Sorex cruzi

Taxon classificationAnimaliaEulipotyphlaSoricidae

Andino-Madrid, Mérida Colindres, Pérez-Consuegra & Matson, 2020

2BEB1545-613A-5106-8170-6F50E43F5A91

####### Natural history.

There is no information regarding this species’ ecology and natural history from Honduras. This is the only species of the *Sorex veraepacis* Alston, 1877 species complex occurring from the mixed pine and broadleaf forest of Cusuco National Park (Andino-Madrid et al. 2020).

####### Distribution.

Endemic from Cusuco National Park in Cortés (Fig. 33); 1836 m a.s.l.

####### Additional notes.

There have been no specific studies focusing on this endemic species. Global conservation status is NE and CR in Honduras.

###### 72. Sorex mccarthyi

Taxon classificationAnimaliaEulipotyphlaSoricidae

Matson & Ordóñez-Garza, 2017

64F92E40-767D-51D0-8CD3-BBF185F3BFA6

####### Natural history.

There is no information regarding this species’ ecology and natural history from Honduras.

####### Distribution.

**Figure 47.**
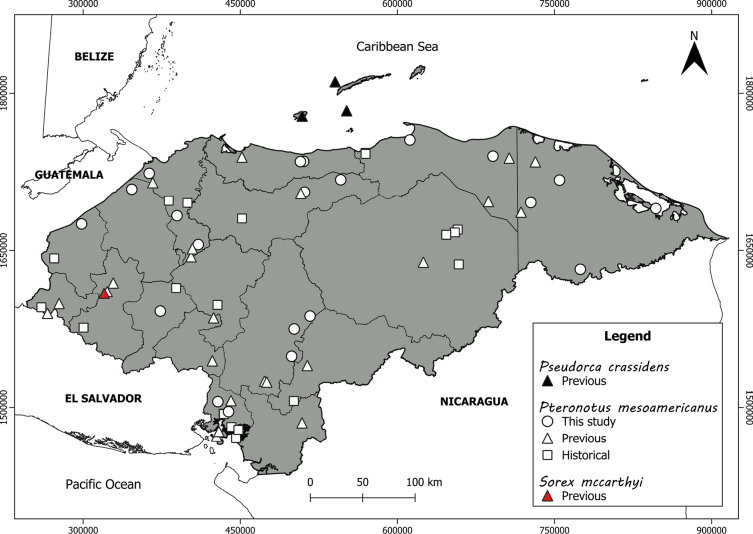
Distribution of *Pteronotus mesoamericanus*, *Pseudorca crassidens*, and *Sorex mccarthyi* in Honduras.

Endemic from the Celaque National Park cloud forest in Lempira, the type locality (Fig. 47); 2560 m a.s.l.

####### Additional notes.

No additional information is available for this endemic species from western Honduras (see Matson and Ordóñez-Garza 2017). Global conservation status is NE and CR in Honduras.

#### Chiroptera Blumenbach, 1779

Names and order follow Simmons and Cirranello (2026) for Honduran distributions. Exceptions and clarifications are noted in relevant species accounts. The distributions presented here have been updated and verified from Turcios-Casco (2021b) with confirmations of new localities.

### Emballonuridae Gervais, 1855

#### *Balantiopteryx* Peters, 1867

##### 73. Balantiopteryx io

Taxon classificationAnimaliaChiropteraEmballonuridae

Thomas, 1904

8B430B0D-EA4B-58EB-BE02-6427A0EE3459

###### Natural history.

Six nonreproductive individuals were captured with a harp trap placed at the entrance of a small side cavern within a cave on 11 March 2011 and subsequently released; additional species found in the cave were *Saccopteryx bilineata* (Temminck, 1838) and *Phyllostomus hastatus* (Pallas, 1767) (Divoll and Buck 2013). These records confirm the species’ first occurrence in the country.

###### Distribution.

The first record of this species in Honduras was from individuals captured and released at the Piedra Cocha cave, Masca, Rio Masca, in the department of Cortés (Fig. 41), at 0–5 m a.s.l. An additional record has also been reported from the Department of Cortés (see www.inaturalist.org/observations/5021955; Divoll and Buck 2013).

###### Acoustic identification.

Call shapes are variations of the Emballonurid hoop or upside-down U shape, usually recorded as narrow band FM decreasing frequency sloping single pulses. 90% of the Fc pulses of the dominant harmonic are 52 kHz.

###### Additional notes.

Considerable disturbance and cave vandalism may adversely affect this species, as has been the case in Belize, where it has been extirpated from several historical cave roosts due to tourism-related adventure caving. This site should be revisited to confirm that this population remains extant, as it is the only known cave in Honduras where it occurs. More acoustic surveys are needed to confirm the distribution of this species in other regions of Honduras. Disturbance is a key threat to this obligate cave-roosting species. Global and national conservation status is VU.

##### 74. Balantiopteryx plicata

Taxon classificationAnimaliaChiropteraEmballonuridae

Peters, 1867

A6A22F27-4294-5E78-88FD-EB6E854AAE90

###### Natural history.

Individuals have been found roosting in caves, rock crevices, and human structures such as abandoned houses and church bell towers. At least 25 individuals were found roosting in crevices between rocks in the ground at Microcuenca Santa Isabel in the department of Choluteca. Another roost of > 50 individuals was found in an abandoned bathroom in a sugar cane plantation in Francisco Morazán. Pregnant females have been captured in May and March (each with one embryo). It has been recorded in pine, broadleaf, mixed, dry, and mangrove forests, pine savannas, agricultural lands, pastures, and near human settlements (Mejía-Quintanilla et al. 2018; Gómez-Corea et al. 2021a).

###### Distribution.

**Figure 48.**
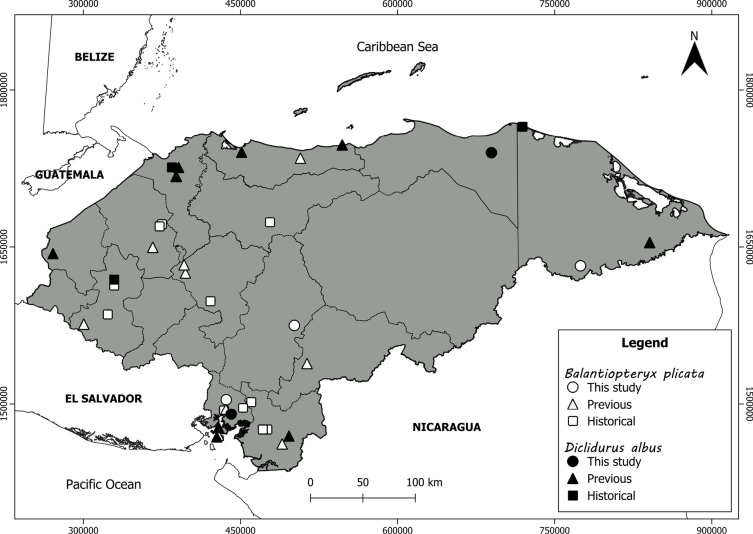
Distribution of *Balantiopteryx plicata* and *Diclidurus albus* in Honduras.

Atlántida, Comayagua, Choluteca, El Paraíso, Francisco Morazán, Gracias a Dios, Lempira, Santa Bárbara, Valle and Yoro" (Fig. 48); 90–630 m a.s.l.

###### Acoustic identification.

Call shapes are variations of the Emballonurid hoop or upside-down U shape, usually recorded as narrow band FM decreasing frequency sloping single pulses. 90% of the Fc pulses of the dominant harmonic are 46 kHz.

###### Additional notes.

Global conservation status is LC and NE in Honduras.

#### *Centronycteris* Gray, 1838

##### 75. Centronycteris centralis

Taxon classificationAnimaliaChiropteraEmballonuridae

Thomas, 1912

65D5ABCE-39D6-552C-8B16-A899F7F33EB3

###### Natural history.

It is found predominantly in undisturbed tropical forests (Espinal et al. 2021).

###### Distribution.

**Figure 49.**
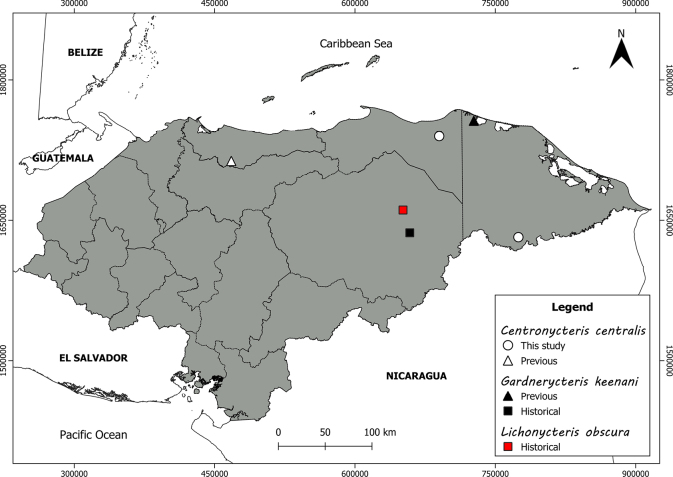
Distribution of *Centronycteris centralis*, *Gardnerycteris keenani*, and *Lichonycteris obscura* in Honduras.

Atlántida, **Colón**, and **Gracias a Dios** (Fig. 49); 150–1005 m a.s.l.

###### Acoustic identification.

Call shapes are single QCF inverted-U pulses that are regularly spaced and maintained at a constant frequency. 90% of the Fc pulses of the dominant harmonic are 41 kHz.

###### Additional notes.

Global conservation status is LC and DD in Honduras.

#### *Cormura* Peters, 1867

##### 76. Cormura brevirostris

Taxon classificationAnimaliaChiropteraEmballonuridae

(Wagner, 1843)

C2F6F078-6727-54ED-9EE0-6701FA489BCF

###### Natural history.

This species was recorded at two locations during exploratory acoustic surveys in the Warunta Conservation Area, along the edges of the Segovia and Rio Warunta rivers, and in the forest understory (Turcios-Casco et al. 2025a).

###### Acoustic identification.

Call shapes are typical Emballonurid upside-down, loosely U-shaped-shaped pulses, distinctive three-note, increasing-frequency pulses often referred to as the “Do, Re, Me” bat, similar to notes on the musical scale. 90% of the combined Fc pulses of the dominant harmonic are 33 kHz.

###### Distribution.

Gracias a Dios (10 m a.s.l.; Fig. 8).

###### Additional notes.

No specimens are known from the country. Global conservation status is LC and NE in Honduras.

#### *Diclidurus* Wied-Neuwied, 1820

##### 77. Diclidurus albus

Taxon classificationAnimaliaChiropteraEmballonuridae

Wied-Neuwied, 1820

50E3C6DB-8215-5A02-99CC-653A59175BBA

###### Natural history.

It has been recorded in broadleaf and dry forests, secondary vegetation, mangrove swamps, near water bodies (Mejía-Quintanilla et al. 2018; Gómez-Corea et al. 2020), and urban areas. It is considered regionally and forages high over the canopy and urban areas and is readily detected acoustically (Miller et al. 2023).

###### Acoustic identification.

Call shapes are single, narrow-band FM, decreasing frequency, sloping pulses. 90% of the Fc pulses of the dominant harmonic are 22.5 kHz.

###### Distribution.

Atlántida, Choluteca, **Colón**, Copán, Cortés, Gracias a Dios, Lempira, and Valle (Fig. 48); 0–680 m a.s.l.

###### Additional notes.

Global conservation status is LC and NE in Honduras.

#### *Peropteryx* Peters, 1867

##### 78. Peropteryx kappleri

Taxon classificationAnimaliaChiropteraEmballonuridae

Peters, 1867

88374B04-2907-5DEC-B192-68BE5684DCEF

###### Natural history.

Pregnant and lactating females with pups have been captured in May (Valdez and LaVal 1971). It has been found in pine, broadleaf, mixed, and mangrove forests, pine savannas, dry forests, agricultural areas, and human settlements (Mejía-Quintanilla et al. 2018). However, it mainly roosts in caves. Other species known to roost in the same cave include *P. macrotis*, *P. hastatus*, *Artibeus* Leach, 1821, and *Glossophaga* E. Geoffroy, 1818.

###### Distribution.

**Figure 50.**
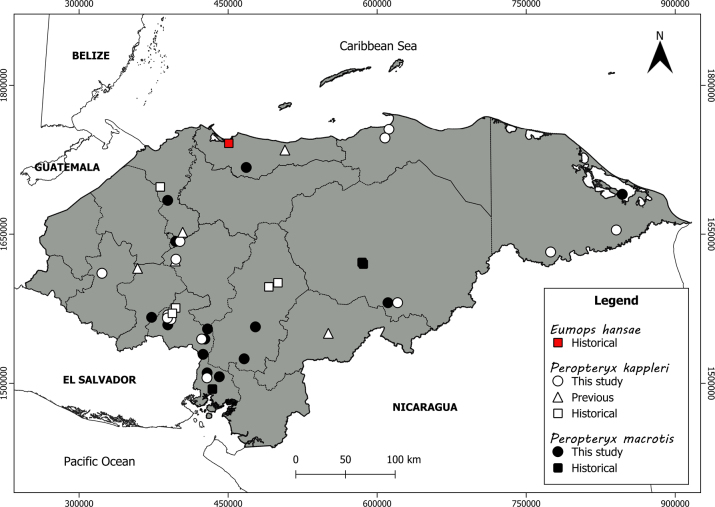
Distribution of *Eumops hansae*, *Peropteryx kappleri*, and *P. macrotis* in Honduras.

Atlántida, **Colón**, Comayagua, Cortés, El Paraíso, Francisco Morazán, **Gracias a Dios**, Intibucá, La Paz, Lempira, **Olancho**, Santa Bárbara, and Valle (Fig. 50); 30–910 m a.s.l.

###### Acoustic identification.

Call shapes are single, narrow-band FM sloping single pulses from high to lower frequency. Unlike other Emballonurids, the genus *Peropteryx* has a distinct size dimorphism between the sexes. The vocal signatures of the smaller males resulted in the Fc being several kHz higher than that of the larger females (Miller et al. 2024). 90% of the Fc pulses of the dominant harmonic are 32 kHz.

###### Additional notes.

Global conservation status is LC and NE in Honduras.

##### 79. Peropteryx macrotis

Taxon classificationAnimaliaChiropteraEmballonuridae

(Wagner, 1843)

C23EEAE7-07A7-526D-B64F-884CCA4801E7

###### Natural history.

The species roosts in caves and rock formations and has been found in croplands, human settlements, and broadleaf, mixed, and pine forests (Mejía-Quintanilla et al. 2018; Turcios-Casco et al. 2021b). A pregnant female with one embryo and lactating females were captured in May.

###### Acoustic identification.

Call shapes are single, narrow-band FM sloping single pulses from high to lower frequency. Unlike other Emballonurids, the genus *Peropteryx* has a distinct size dimorphism between the sexes. The vocal signatures of the smaller males resulted in the Fc being several kHz higher than that of the larger females (Miller et al. 2024). 90% of the Fc pulses of the dominant harmonic are 42 kHz.

###### Distribution.

**Atlántida**, **Francisco Morazán**, **La Paz**, Olancho, and **Valle** (Fig. 50); 0–1005 m a.s.l.

###### Additional notes.

Global conservation status is LC and NE in Honduras.

#### *Rhynchonycteris* Peters, 1867

##### 80. Rhynchonycteris naso

Taxon classificationAnimaliaChiropteraEmballonuridae

(Wied-Neuwied, 1820)

C85503BE-12A6-5012-B165-D4ABEC42DABF

###### Natural history.

It is typically found in riparian habitats, where individuals forage over water bodies. While crepuscular, they are often active during the day, roosting on the undersides of overhanging tree trunks or on rocks at the water’s edge, often in groups of up to six individuals. The only ectoparasitic arthropod recorded on *R. naso* in Honduras is a streblid bat fly, *Trichobius parasiticus* Gervais, 1844. Dick (2013) recorded this streblid bat fly from Olancho, but he considered the occurrence accidental or transitory.

###### Distribution.

**Figure 51.**
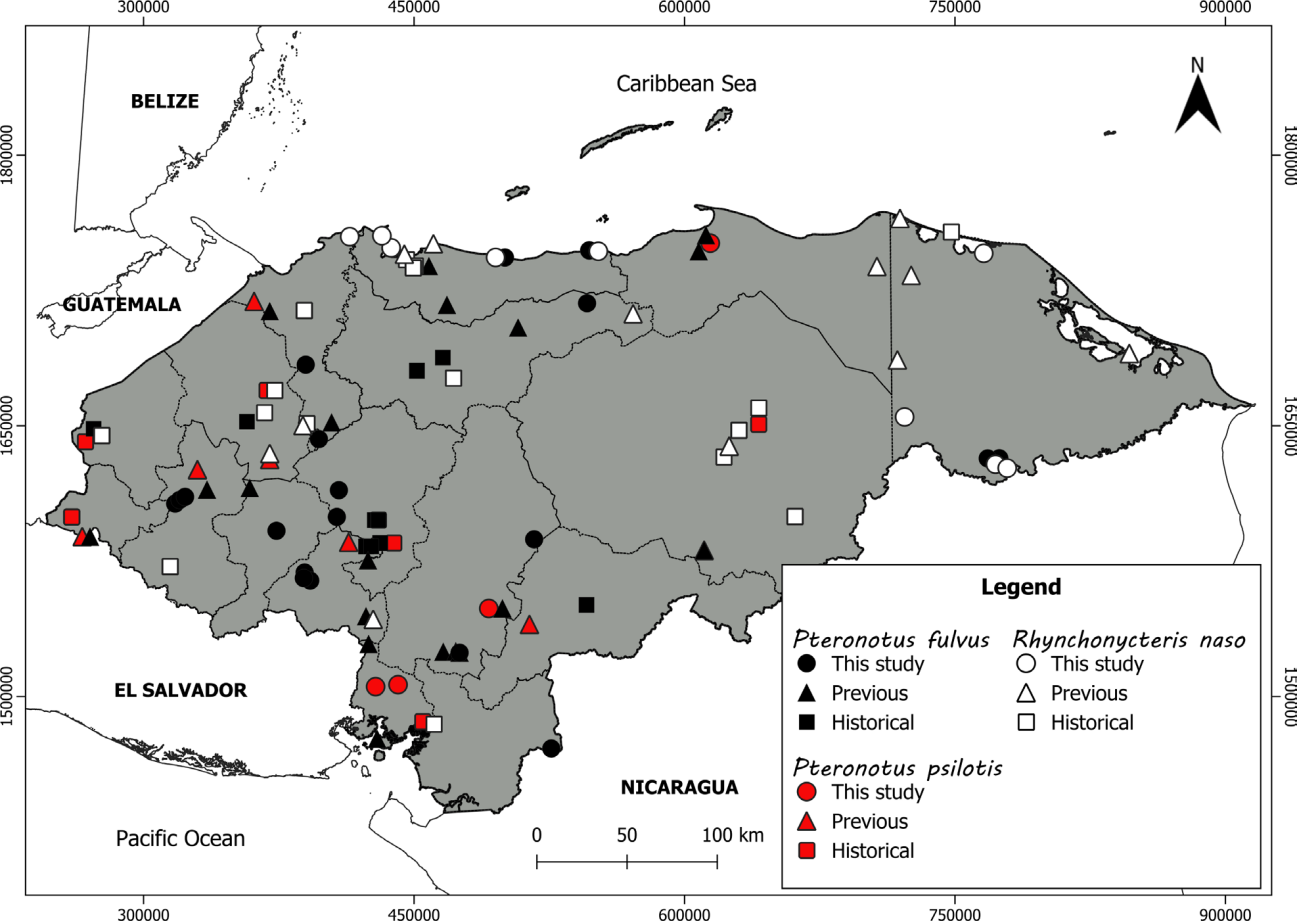
Distribution of *Pteronotus fulvus*, *P. psilotis*, and *Rhynchonycteris naso* in Honduras.

Atlántida, Choluteca, Copán, Cortés, Gracias a Dios, La Paz, Lempira, Olancho, Santa Bárbara, and Yoro (Fig. 51); 10–250 m a.s.l.

###### Acoustic identification.

Call shapes are single, narrow-band FM sloping single pulses from high to lower frequency. If recorded very close to the bats, broadband calls will be recorded. The dominant harmonic is usually the H3. However, this depends on the bats’ distance at the time of recording, so the dominant harmonic may be either H2 or H3 (Miller et al. 2024). 90% of the Fc pulses of the dominant harmonic are 91 kHz.

###### Additional notes.

Goodwin (1942a) referred to this as *Rhynchiscus naso* (J. A. Allen, 1911), and historical records using that name are *Rhynchonycteris naso*. Global conservation status is LC and NE in Honduras.

#### *Saccopteryx* Illiger, 1811

##### 81. Saccopteryx bilineata

Taxon classificationAnimaliaDipteraEmballonuridae

(Temminck, 1838)

7ED346A6-95A9-5C0E-8C3A-F0D8E3A1865C

###### Natural history.

This species has been found roosting in tree cavities (Medina-Fitoria and Turcios-Casco 2019), on tree branches in tree canopies, and on the trunks of hollow trees (*Ficus aurea* Nutt.) in Islas de la Bahía; and it commonly roosts in old buildings (Bermingham et al. 1998; Martinez-Murillo et al. 2021). It occurs in pine forests, broadleaf forests, pine savannas, crops, and pastures (Mejía-Quintanilla et al. 2018).

###### Distribution.

**Figure 52.**
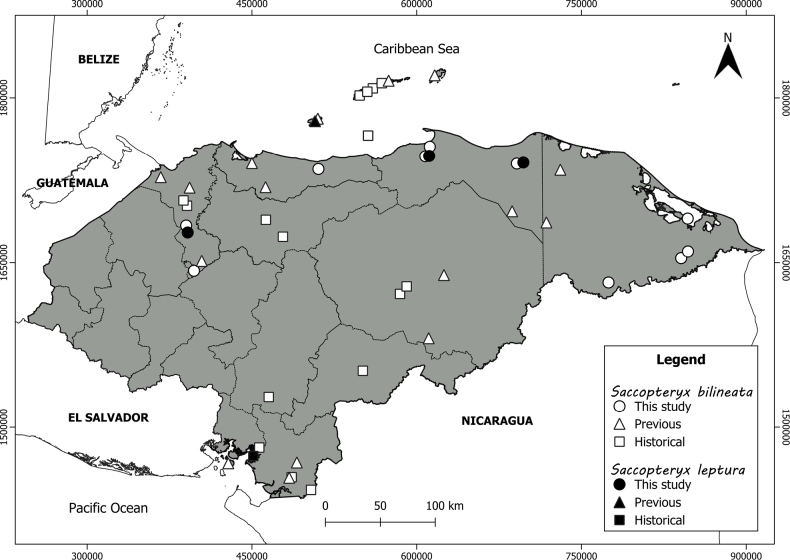
Distribution of *Saccopteryx bilineata* and *S. leptura* in Honduras.

Atlántida, Choluteca, Colón, Cortés, El Paraíso, Francisco Morazán Gracias a Dios, Islas de la Bahía, Olancho, Valle, and Yoro (Fig. 52); 0–605 m a.s.l.

###### Acoustic identification.

Call shapes are distinctive, paired QCF inverted U-shaped ascending pulses, with a higher-frequency pulse followed by a lower-frequency, similarly shaped pulse (Miller et al. 2024). 90% of the combined Fc pulses of the dominant harmonic are 49 kHz.

###### Additional notes.

Global conservation status is LC and NE in Honduras.

##### 82. Saccopteryx leptura

Taxon classificationAnimaliaDipteraEmballonuridae

(Schreber, 1774)

5FB4FAC2-3648-5419-8E99-742D878C93D8

###### Natural history.

It has been found in pine, broadleaf, mixed, and mangrove forests, pine savannas, and agricultural lands. It is likely sympatric with *S. bilineata* in Honduras, as it is throughout its range, although roosting requirements may differ. It has been reported to hang on branches of *Ficus aurea* as a day roost.

###### Distribution.

Choluteca, Colón, Cortés, and Islas de la Bahía (Fig. 52); 0–260 m a.s.l.

###### Acoustic identification.

Call shapes are distinctive: paired QCF inverted U-shaped ascending pulses, with a higher-frequency pulse followed by a lower-frequency, similarly shaped pulse. While sympatric, call shapes are very similar to those of *S. bilineata*, although the Fc is considerably higher and readily identified as distinct. 90% of the combined Fc pulses of the dominant harmonic are 57 kHz.

###### Additional notes.

A green vine snake (*Oxybelis fulgidus* (Daudin, 1803)) apparently died trying to feed on a *S. leptura* in Útila on the Islas de la Bahía department (Brown et al. 2020). Global conservation status is LC and NE in Honduras.

####### Mormoopidae Saussure, 1860

#### *Mormoops* Leach, 1821

##### 83. Mormoops megalophylla

Taxon classificationAnimaliaChiropteraMormoopidae

(Peters, 1864)

647497CC-58C4-57E8-AF16-2DEF6C54CF69

###### Natural history.

They have been captured above streams and near caves and have been documented roosting in caves elsewhere in their range (Rezsutek and Cameron 1993). It is suspected that, like in Belize, it is an obligate cave-roosting species during the reproductive season, using selected caves for nursery colonies.

###### Distribution.

**Figure 53.**
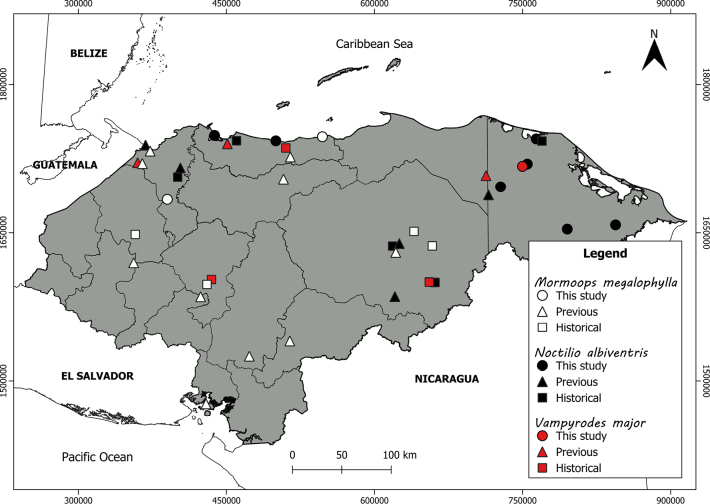
Distribution of *Mormoops megalophylla*, *Noctilio albiventris*, and *Vampyrodes major* in Honduras.

Atlántida, Comayagua, Cortés, El Paraíso, Francisco Morazán, Gracias a Dios, La Paz, Lempira, Olancho, Santa Bárbara, and Yoro (Fig. 53); 115–1600 m a.s.l.

###### Acoustic identification.

Call shapes are single FM pulses of decreasing frequency. Pulses may have a very short decreasing FM portion followed by a Quasi-CF part with a trailing decreasing FM component (Miller et al. 2024). 90% of the Fc pulses of the dominant harmonic are 52 kHz.

###### Additional notes.

See Mejía-Quintanilla et al. (2022) for more comments on their distribution. Global conservation status is LC and NT in Honduras.

#### *Pteronotus* Gray, 1838

##### 84. Pteronotus fulvus

Taxon classificationAnimaliaNeogastropodaMormoopidae

(Thomas, 1892)

E65EB739-F128-59C9-8713-8851AD215C25

###### Natural history.

Individuals have been captured above estuaries, over dry streams, and near caves.

###### Distribution.

**Atlántida**, **Choluteca**, **Colón**, Comayagua, Cortés, El Paraíso, Francisco Morazán, Intibucá, La Paz, Lempira, Ocotepeque, Olancho, Santa Bárbara, Valle, and Yoro (Fig. 51); 10–1900 m a.s.l.

###### Acoustic identification.

Call shapes are distinctive, lazy Z or reversed S single FM pulses of decreasing frequency. Pulses have a very short increasing FM portion, followed by a trailing decreasing FM component, followed by a short increasing FM component. 90% of the Fc pulses of the dominant harmonic are 68 kHz (Miller et al. 2024).

###### Additional notes.

See Mejía-Quintanilla et al. (2022) for additional comments on distribution. Previous records referred to it as *Pteronotus davyi* J. E. Gray, 1838. Following the separation of *P. davyi* and *P. fulvus* based on molecular evidence, historical records attributed to *P. davyi* are now considered to be *P. fulvus* in central and northern Central America and Mexico. However, the species limits of these two remain unclear (Pavan and Marroig 2017). There may be a zone of overlap in Nicaragua that could extend into Honduras. Global and national conservation status is NE in Honduras.

##### 85. Pteronotus gymnonotus

Taxon classificationAnimaliaNeogastropodaMormoopidae

(Wagner, 1843)

A46D8D43-AEDB-5231-87B5-B6DE0A1D2F4C

###### Natural history.

This species appears to be obligate cave-roosting.

###### Distribution.

**Atlántida**, Comayagua, Cortés, El Paraíso, Francisco Morazán, Gracias a Dios, La Paz, Olancho, and **Yoro** (Fig. 42); 10–1600 m a.s.l.

###### Acoustic identification.

Call shapes are similar to those of *P. fulvus*, with call shape having the same distinctive lazy Z or reversed S single FM pulses of decreasing frequency as others in the genus (Miller et al. 2024). Pulses have a very short increasing FM portion, followed by a trailing decreasing FM component, followed by a short increasing FM component. 90% of the Fc pulses of the dominant harmonic are 55 kHz.

###### Additional notes.

See Mejía-Quintanilla et al. (2022) for more comments on their distribution. Global conservation status is LC and NE in Honduras.

##### 86. Pteronotus mesoamericanus

Taxon classificationAnimaliaNeogastropodaMormoopidae

Smith, 1972

D62B4122-BA4F-5BA3-AF50-4B3281CC8FD1

###### Natural history.

Individuals have been captured in dry streambeds, above bodies of water (including dry ponds), and near caves. Lactating females have been captured during March and May. The only known roosts in Honduras are abandoned mines. Ectoparasites reported include *Nycterophilia coxata* Ferris, 1916, from Copán and Olancho; *Trichobius caecus* Edwards, 1918, from Atlántida, Copán, and Olancho; and *Trichobius parasiticus* Gervais, 1844 (Dick 2013), and *Trichobius yunkeri* Wenzel, 1966, in Comayagua (Graciolli et al. 2021).

###### Distribution.

Todos los departamentos, a excepción de Islas de la Bahía, Colón, Comayagua, Copán, Cortés, El Paraíso, Francisco Morazán, Gracias a Dios, Intibucá, La Paz, Lempira, Ocotepeque, Olancho, Negrita, Valle, and Yoro (Fig. 47), 30–1520 m a.s.l.

###### Acoustic identification.

Call sequences are highly distinctive and the only one in Honduras that produces narrowband, long-CF single pulses at a single frequency (Miller et al. 2024). 90% of the Fc pulses of the dominant harmonic are 64 kHz.

###### Additional notes.

See Mejía-Quintanilla et al. (2022) for more comments on their distribution. Historical specimens have been identified as *Chilonycteris parnellii* (Allen, 1911), *Pteronotus parnellii* (Gray, 1843); *Pteronotus parnellii mesoamericanus* Smith, 1972; *Pteronotus parnellii mexicanus* (Miller, 1902); and *Pteronotus rubiginosa* Wagner, 1843. Global conservation status is LC and NE in Honduras.

##### 87. Pteronotus psilotis

Taxon classificationAnimaliaNeogastropodaMormoopidae

(Dobson, 1878)

0C5F4886-00C1-5090-9638-3EFD144982C0

###### Natural history.

Individuals have been captured above bodies of water. Roosts of this species have been found in abandoned mines and caves. Pregnant females with one embryo were captured in March and May.

###### Distribution.

**Colón**, Comayagua, Copán, Cortés, El Paraíso, **Francisco Morazán**, La Paz, Lempira, Ocotepeque, Olancho, Santa Bárbara, and Valle (Fig. 51); 30–235 m a.s.l.

###### Acoustic identification.

Call shapes are distinctive, lazy Z or reversed S, and single FM pulses of decreasing frequency. Pulses have a very short increasing FM portion, followed by a trailing decreasing FM component, followed by a short increasing FM component. 90% of the Fc pulses of the dominant harmonic are 79 kHz.

###### Additional notes.

Historical records of *Pteronotus personatus* (Wagner, 1843) are now recognized as this species. See Mejía-Quintanilla et al. (2022) for more comments on their distribution. Global and national conservation status is NE.

####### Noctilionidae Gray, 1821

#### *Noctilio* Linnaeus, 1766

##### 88. Noctilio albiventris

Taxon classificationAnimaliaChiropteraNoctilionidae

Desmarest, 1818

F779CBF0-A1DA-501E-BE34-1A2699BB637F

###### Natural history.

This species forages over ponds and rivers and is sympatric with *N. leporinus*. A streblid bat fly, *Paradyschiria parvuloides* Wenzel, 1966, was reported from an individual captured in Olancho (Dick 2013).

###### Acoustic identification.

Call shapes are distinctive and similar to those of *Noctilio leporinus* (Linnaeus, 1758), but with a higher Fc, comprising a short Quasi-CF component followed by a broadband FM-decreasing frequency component (Miller et al. 2024). 90% of the Fc pulses of the dominant harmonic are 70 kHz.

###### Distribution.

Atlántida, Cortés, El Paraíso, Francisco Morazán, Gracias a Dios, Olancho, and Valle (Fig. 53); 0–385 m a.s.l.

###### Additional notes.

Global conservation status is LC and NT in Honduras.

##### 89. Noctilio leporinus

Taxon classificationAnimaliaChiropteraNoctilionidae

(Linnaeus, 1758)

6E5BC5D6-5149-5C61-A1D7-2CF408555862

###### Natural history.

As piscivores, most records would be expected near water bodies, often foraging near ocean docks (Martínez-Murillo et al. 2021), tilapia breeding tanks, and under bridges. Three species of streblid bat flies have been found on *N. leporinus*: *Aspidoptera falcata* Wenzel, 1976, *Noctiliostrebla traubi* Wenzel, 1966, and *Paradyschiria fusca* Speiser, 1900 from Olancho (Dick 2013). The first is considered an accidental or transitory occurrence.

###### Distribution.

**Figure 54.**
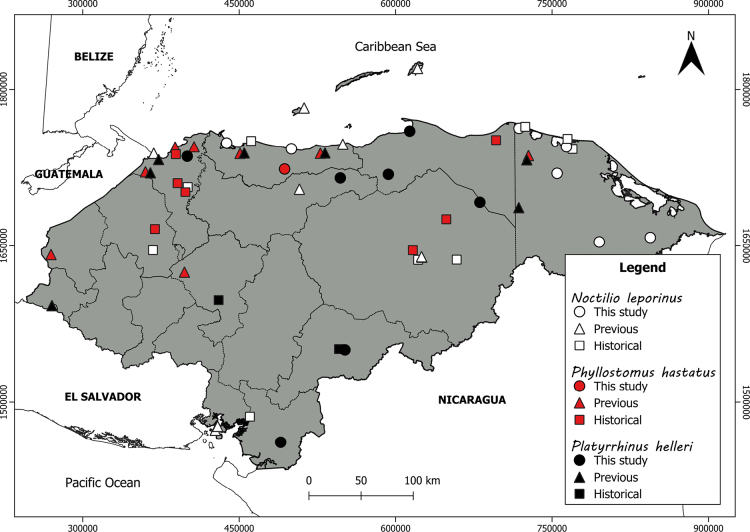
Distribution of *Noctilio leporinus*, *Phyllostomus hastatus*, and *Platyrrhinus helleri* in Honduras.

Atlántida, Cortés, El Paraíso, Gracias a Dios, Islas de la Bahía, Olancho, Santa Bárbara, Valle, and Yoro (Fig. 54); 0–325 m a.s.l.

###### Acoustic identification.

Call shapes are broadband, with a distinctive short Quasi-CF component followed by a broadband FM component decreasing in frequency (Miller et al. 2024). 90% of the Fc pulses of the dominant harmonic are 55 kHz.

###### Additional notes.

Global conservation status is LC and NT in Honduras.

####### Thyropteridae Miller, 1907

#### *Thyroptera* Spix, 1823

##### 90. Thyroptera tricolor

Taxon classificationAnimaliaChiropteraThyropteridae

Spix, 1823

19BFAA39-16BA-5C9E-90C0-D62179C12D00

###### Natural history.

Known to roost in rolled leaves of *Heliconia rostrata* Ruiz and Pav., with up to seven individuals, including young, found in one leaf in February (Turcios-Casco and Medina-Fitoria 2019). A female with two young was captured in another species of *Heliconia* L. in June. Also frequently captured flying above streams. Following an approximately 10-km non-linear route along a tributary of the Wuarska River in Colón, three colonies and five roosts were recorded in *Heliconia* spp., in leaves approximately 5 m above the ground. A refuge was located 6 m from the Zacate River and was occupied by six individuals within a Heliconia plant representing the first record for the Department of Atlántida and Pico Bonito National Park.

###### Distribution.

**Figure 55.**
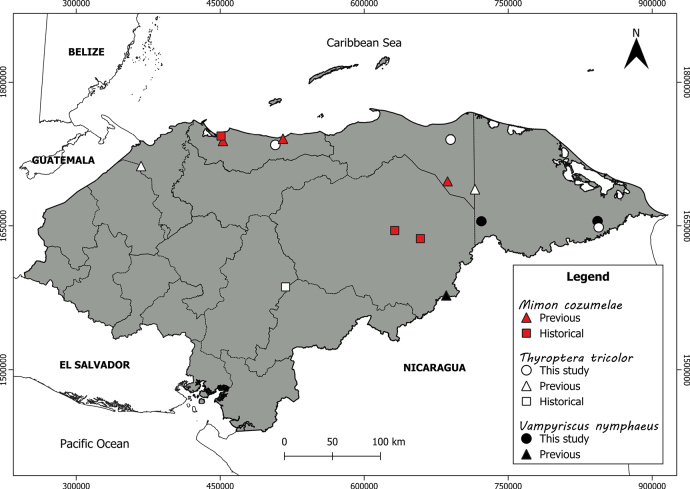
Distribution of *Thyroptera tricolor*, *Mimon cozumelae*, and *Vampyriscus nymphaeus* in Honduras.

**Atlántida**, Colón, Cortés, Francisco Morazán, and Gracias a Dios (Fig. 55); 50–800 m a.s.l.

###### Acoustic identification.

Calls are very low-intensity broadband FM pulses. While it is possible to record this species when very close (< 2 meters), it appears too quiet for free-flying field identification (Miller et al. 2024).

###### Additional notes.

See Gardner (2007) regarding the clarification of a *Thyroptera discifera* record from Honduras that corresponded to a location in Venezuela and was included in error in the Mammals of Honduras (Goodwin 1942a). Deforestation, resource exploitation, land-use changes, and livestock activities are among the biggest threats in Honduras. Global conservation status is LC and DD in Honduras.

####### Phyllostomidae Gray, 1825

#### *Micronycteris* Gray, 1866

Morales-Martínez et al. (2021) suggest there may be an undescribed species of *Micronycteris* in northern Honduras. Additional specimens are needed to confirm the status of all potential species. Medina-Van Berkum et al. (2020) reported *Micronycteris minuta* (Gervais, 1856) from Cortés; however, given the description of *Micronycteris tresamici* Siles & Baker, 2020, these specimens should be carefully reexamined. As *M. tresamici* may be sympatric with *M. minuta* in Central America (Siles and Baker 2020), we assume both species likely occur here, pending additional morphological and ecological clarification of the species’ limits.

##### 91. Micronycteris tresamici

Taxon classificationAnimaliaChiropteraPhyllostomidae

(Siles & Baker, 2020)

A07DC1B6-8601-5A77-BFAD-1B03700758CE

###### Natural history.

It has been captured in banana plantations with large trees nearby. This species may be sympatric with *M. minuta* Gervais, 1856, in Central America (Siles and Baker 2020), While we assume that both species may eventually be documented here, all records used here refer to *M. tresamici*. Pending additional morphological and ecological clarification of their species limits, *M. minuta* remains only probable as areas of sympatry remain unclear.

###### Distribution.

Known only from Atlántida (Fig. 28); 225–930 m a.s.l.

###### Additional notes.

This species may have been identified in previous records as *M. minuta*. Global and national conservation status is NE.

##### 92. Micronycteris schmidtorum

Taxon classificationAnimaliaChiropteraPhyllostomidae

Sanborn, 1935

ACF0AF46-81D6-5E93-AC36-671B2A66AFD7

###### Natural history.

This species has been found roosting in caves (Turcios-Casco et al. 2019a). The population on Islas de la Bahía may be a disjunct population of the species (see Bermingham et al. 1998; Martínez-Murillo et al. 2021; Turcios-Casco et al. 2021b).

###### Distribution.

Atlántida, Copán, Cortés, Islas de la Bahía, and Santa Bárbara (Fig. 26); 0–1000 m a.s.l. Recent surveys (Martínez-Murillo et al. 2021) did not detect this species on the Islas de la Bahía, suggesting that it may have been extirpated from the islands.

###### Additional notes.

Global conservation status is LC and DD for Honduras.

##### 93. Micronycteris hirsuta

Taxon classificationAnimaliaChiropteraPhyllostomidae

(Peters, 1869)

BEAC1631-FD80-524D-A83D-6108B1166777

###### Natural history.

It has been captured above streams, banana plantations, and primary and secondary growth forests.

###### Distribution.

**Choluteca**, El Paraíso, Gracias a Dios, and Olancho (Fig. 25); 30–810 m a.s.l.

###### Additional notes.

Global conservation status is LC and VU in Honduras.

##### 94. Micronycteris minuta

Taxon classificationAnimaliaChiropteraPhyllostomidae

complex

E4FE67EB-B5F1-59D4-B758-DA7508FF02E1

###### Natural history.

The species limits of this complex require confirmation.

###### Distribution.

Probably northern and northeastern Honduras.

###### Additional notes.

Specimens are needed in collections. Although no assessment has been made, it may be threatened by the decline in tropical lowland forests in Honduras. See Siles and Baker (2020) for more information regarding this complex.

##### 95. Micronycteris microtis

Taxon classificationAnimaliaChiropteraPhyllostomidae

Miller, 1898

9D6CE842-BB01-5573-8403-4309D5B20515

###### Natural history.

They have been captured at cave entrances and in the Cusuco National Park, a colony of nine individuals was found occupying a roost along the bank of a small forest stream.

###### Distribution.

Atlántida, Colón, Comayagua, Cortés, Francisco Morazán, Gracias a Dios, Lempira, Olancho, and Santa Bárbara (Fig. 29); 30–1700 m a.s.l.

###### Additional notes.

Previous records from Honduras have been referred to as *Micronycteris megalotis mexicanus* Miller, 1898. The distribution of specimens from Andersen (1906) only listed the Bay of Honduras. Global conservation status is LC and NE in Honduras.

#### *Desmodus* Wied-Neuwied, 1826

##### 96. Desmodus rotundus

Taxon classificationAnimaliaChiropteraPhyllostomidae

(É. Geoffroy Saint-Hilaire, 1810)

6991C2B1-8B1E-5F52-8BCE-8391E48D13AC

###### Natural history.

The common vampire has been captured above bodies of water and near streams (75–300 m), and over corn crops and cattle pastures, and is known to prey on cattle. Pregnant females with one embryo have been captured in January, March, May, and December. Lactating females have been captured in January, September, October, and December. Males with enlarged testes have been captured in March, May, July, September, October, and November. External parasites include streblid bat flies based on Dick (2013) and Gómez-Corea et al. (2022): *Strebla wiedemanni* Kolenati, 1856 from Atlántida, Copán, Cortés, Francisco Morazán, Gracias a Dios, Lempira, Olancho, and Valle; *Trichobius parasiticus* Gervais, 1844 from Atlántida, Cortés, Francisco Morazán, Gracias a Dios, Lempira, Olancho, and Valle; *Trichobius joblingi* Wenzel, 1966 from Gracias a Dios, Lempira and Olancho; and *Trichobius caecus* Edwards, 1918 from Lempira. Two additional streblids have been recently identified during our fieldwork: *S. wiedemanni* and *T. parasiticus* from Ceguaca (Santa Bárbara).

###### Distribution.

**Figure 56.**
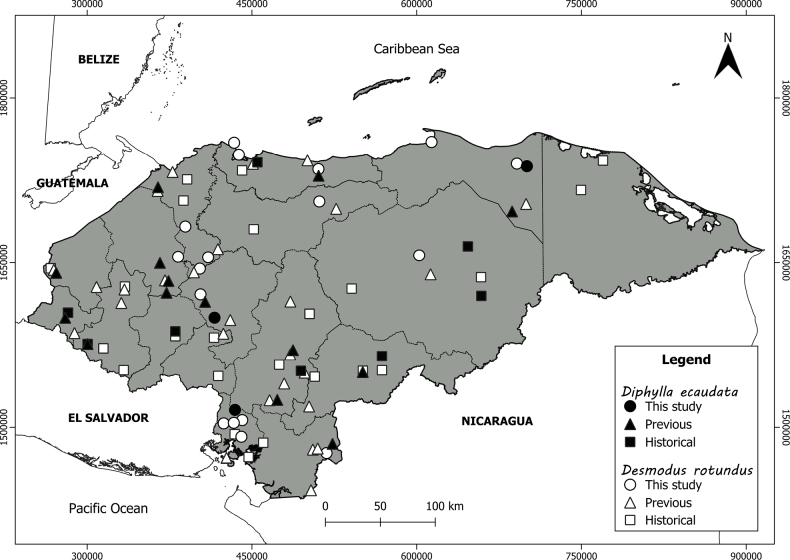
Distribution of *Desmodus rotundus* and *Diphylla ecaudata* in Honduras.

The species has been recorded in all 18 departments of Honduras, and we provide the first record for Intibucá (Fig. 56); 30–1830 m a.s.l.

###### Additional notes.

Vampire bat populations have increased due to anthropogenic habitat modification, resulting in increasing human-wildlife conflicts (Girón-Juárez et al. 2025). Warfarin-based control measures, known as vampiricides (or vampirin in Spanish), have been sold illegally in Honduras. Although these substances are primarily used on cattle, their application negatively impacts other cave-roosting bat communities. Additionally, they are often sold illegally and without regard for environmental consequences. There are reports of people falling asleep in the field while working on their farms and being bitten by vampire bats. In suburban areas, people report that vampire bats enter their homes and bite their toes, generally feeding on blood from the big toe. Global conservation status is LC and NE in Honduras.

#### *Diaemus* Miller, 1906

##### 97. Diaemus youngii

Taxon classificationAnimaliaChiropteraPhyllostomidae

(Jentink, 1893)

F6C1ADB5-C46C-5F7E-A494-3FFE60C8A761

###### Natural history.

Only one record is known: an adult female that escaped after being measured and photographed, representing the first verified record for the country (Turcios-Casco et al. 2020a, 2021b). Reproductive seasons remain unknown.

###### Distribution.

Only Colón (Fig. 44), 80 m a.s.l. It may possibly be found in other lowland areas of Caribbean Honduras.

###### Additional notes.

It is the least known hematophagous species in Honduras. Global conservation status is LC and DD in Honduras.

#### *Diphylla* Spix, 1823

##### 98. Diphylla ecaudata

Taxon classificationAnimaliaChiropteraPhyllostomidae

Spix, 1823

3362AEA9-5DA6-5BC0-8A1F-1B77E7EB8EE3

###### Natural history.

Pregnant females have been captured in August. The species has been captured in coffee plantations, gallery and altered forests, and urban areas. Primary roosts are caves. Two species of streblid bat flies have been reported: *Strebla diphyllae* Wenzel, 1966, and *Trichobius diphyllae* Wenzel, 1966 from Atlántida (Dick 2013).

###### Distribution.

Atlántida, Choluteca, Colón, Comayagua, Copán, Cortés, El Paraíso, Francisco Morazán, Intibucá, Lempira, Ocotepeque, Olancho, Santa Bárbara, and **Valle** (Fig. 56); 550–1900 m a.s.l.

###### Additional notes.

*D. ecaudata* is threatened, as well as *D. rotundus*, due to vampire bat control. Global conservation status is LC and NT in Honduras.

#### *Lonchorhina* Tomes, 1863

##### 99. Lonchorhina aurita

Taxon classificationAnimaliaChiropteraPhyllostomidae

Tomes, 1863

13154327-2547-5B3A-A233-731881040C5A

###### Natural history.

Seasonal reproduction is unknown. Colonies of up to 50 individuals have been found in caverns, mines, and caves (Martínez Murillo et al. 2021). Ectoparasites reported include a streblid bat fly, *Trichobius dugesii* Edwards, 1918, from Islas de la Bahía (Miller 2014).

###### Distribution.

Atlántida, Colón, Cortés, El Paraíso, Francisco Morazán, Islas de la Bahía, **Santa Bárbara**, and Yoro (Fig. 23); 0–1400 m a.s.l.

###### Additional notes.

There is a population in central Honduras that might be disjunct (Ávila-Palma et al. 2020). Agricultural practices resulting in habitat loss are the primary threats. Carboneras (Francisco Morazán) in central Honduras is among the most important areas to conserve the species in Honduras. Global conservation status is LC and VU in Honduras.

#### *Macrophyllum* Gray, 1838

##### 100. Macrophyllum macrophyllum

Taxon classificationAnimaliaPhyllodocidaPhyllostomidae

(Schinz, 1821)

3DF3E2C8-2825-52FC-BDA9-0958EB1B2ADA

###### Natural history.

Historical specimens were captured above the water, near the stream bank.

###### Distribution.

**Figure 57.**
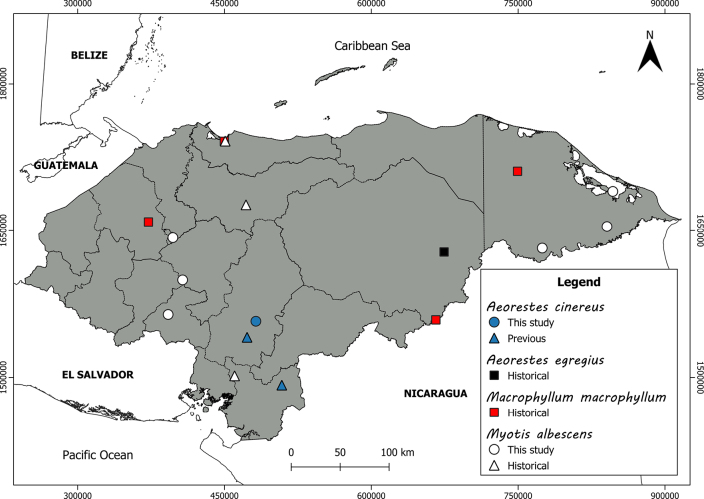
Distribution of *Aeorestes cinereus*, *A. egregius*, *Macrophyllum macrophyllum*, and *Myotis albescens* in Honduras.

Atlántida, El Paraíso, Gracias a Dios, Olancho, and Santa Bárbara (Fig. 57); 130–625 m a.s.l.

###### Additional notes.

Global conservation status is LC and DD in Honduras.

#### *Trachops* Gray, 1847

##### 101. Trachops coffini

Taxon classificationAnimaliaChiropteraPhyllostomidae

Goldman, 1925

35A3D957-321C-59C4-B8F6-02EE43166E5B

###### Natural history.

We have captured the species near large rivers (e.g., the Río Patuca in eastern Honduras). An individual escaped from the net while carrying a frog, *Dendropsophus ebraccatus* (Cope, 1874), in La Moskitia (eastern Honduras).

###### Distribution.

**Figure 58.**
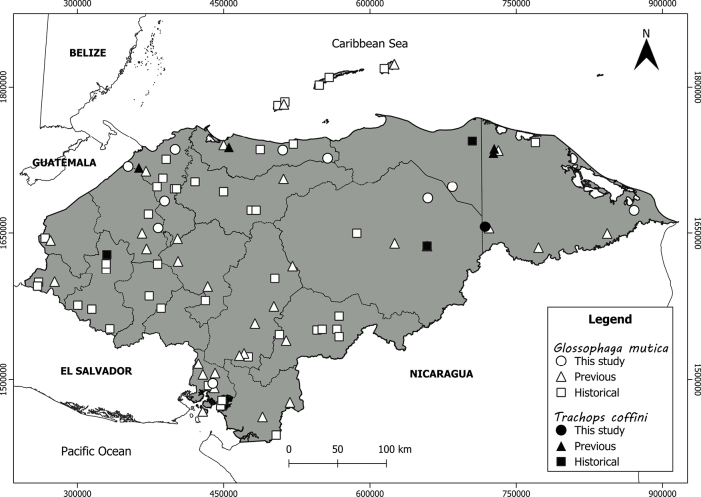
Distribution of *Glossophaga mutica* and *Trachops coffini* in Honduras.

Atlántida, Colón, Cortés, Gracias a Dios, Lempira, and Olancho (Fig. 58); 50–1250 m a.s.l.

###### Additional notes.

Previous records of *Trachops cirrhosus* (Spix, 1823) are now recognized as *T. coffini* Goldman, 1925 (Da Silva Fonseca et al. 2024). Global conservation status is LC and VU in Honduras, both referring to *T. cirrhosus*.

#### *Gardnerycteris* Hurtado & Pacheco, 2014

##### 102. Gardnerycteris keenani

Taxon classificationAnimaliaChiropteraPhyllostomidae

(Handley, 1960)

C20ED864-C815-5018-B1E4-99BB0B715DF9

###### Natural history.

Nothing is known regarding the ecology of this species.

###### Distribution.

Known only from Gracias a Dios and Olancho (Fig. 49); 325 m a.s.l.

###### Additional notes.

An additional record from Gracias a Dios was reported in iNaturalist as *G. crenulata* (É. Geoffroy Saint-Hilaire, 1803) (https://www.inaturalist.org/observations/43038318). Global conservation status is NE and DD in Honduras, as *G. crenulatum*.

#### *Lophostoma* d’Orbigny, 1836

##### 103. Lophostoma nicaraguae

Taxon classificationAnimaliaColeopteraPhyllostomidae

(Goodwin, 1942)

DBEF73F6-990E-5E57-BAB2-B888AFB21E26

###### Natural history.

They have been captured in disturbed forests with tall trees and streams. The only ectoparasite reported was a streblid bat fly, *Mastoptera* sp. (*minuta* complex) from Atlántida (Dick 2013).

###### Distribution.

**Figure 59.**
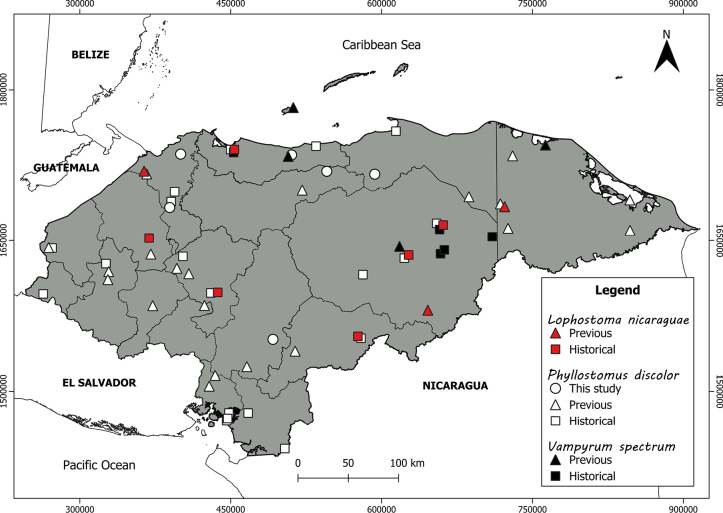
Distribution of *Lophostoma nicaraguae*, *Phyllostomus discolor*, and *Vampyrum spectrum* in Honduras.

Atlántida, Comayagua, Cortés, El Paraíso, Gracias a Dios, Olancho, and Santa Bárbara (Fig. 59); 120–1250 m a.s.l.

###### Additional notes.

We recognize the separation of *Lophostoma brasiliense* Peters, 1867, elevating *L. nicaraguae* to species level (Esquivel et al. 2022). Therefore, historical records for the country referring to *L. brasiliense*, or *Tonatia brasiliense* (Peters, 1867) (see Valdez and LaVal 1971; Turcios-Casco et al. 2021b; Esquivel et al. 2022) are considered *L. nicaraguae*. Global conservation status is NE and VU in Honduras referring to *L. brasiliensis*.

##### 104. Lophostoma evote

Taxon classificationAnimaliaColeopteraPhyllostomidae

(Davis & Carter, 1978)

FB9CDA33-595A-5164-9F85-17C4FF33F7B8

###### Natural history.

Only three records are known in the country; the most recent record dates to 2008.

###### Distribution.

**Figure 60.**
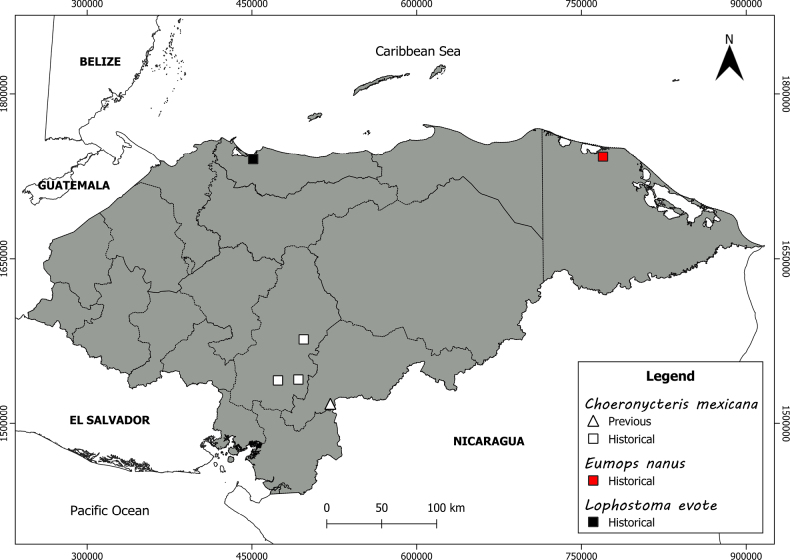
Distribution of *Choeronycteris mexicana*, *Eumops nanus*, and *Lophostoma evote* in Honduras.

Only Atlántida (Fig. 60); 115 m a.s.l.

###### Additional notes.

Historical records originally identified the species as *Tonatia evotis* (Davis & Carter, 1978); however, the name was updated to *Lophostoma evote* to satisfy the rules of zoological nomenclature on the Latin gender agreement. Global conservation status is LC and VU in Honduras, as *L. evotis*.

##### 105. Lophostoma silvicola

Taxon classificationAnimaliaColeopteraPhyllostomidae

d’Orbigny, 1836

90816949-BF1B-5688-950C-0C74D5FEEFDC

###### Natural history.

There are only six records of the species in the country. Reproductive seasonal activity remains unknown. It has been captured above streams. It is the host of two species of streblid bat flies: *Strebla kohlsi* Wenzel, 1966, and *Mastoptera minuta* (Costa Lima, 1921), both from the Atlántida department (Dick 2013). Recently, in November, lactating females and scrotal males were documented in a dry forest in the Sula Valley, northwestern Honduras.

###### Distribution.

Atlántida, **Cortés**, Gracias a Dios, and Olancho (Fig. 40); 145–1415 m a.s.l.

###### Additional notes.

Historical specimens are referred to as *Lophostoma silvicolum* d'Orbigny, 1836 or *Tonatia silvicola* (d’Orbigny, 1836). Habitat loss due to deforestation for agriculture in the La Moskitia region is among the main threats (Turcios-Casco et al. 2020c). Global conservation status is LC and VU in Honduras, as *L. silvicolum*.

#### *Tonatia* Gray, 1827

##### 106. Tonatia bakeri

Taxon classificationAnimaliaChiropteraPhyllostomidae

Williams, Willig & Reid, 1995

AF226B61-92F6-5309-9CBF-5C3AD9B2F590

###### Natural history.

Lactating females have been captured in August. It has been captured above streams. It is known to host two species of streblid bat flies: *Strebla galindoi* Wenzel, 1966, and *Trichobius* sp. (undescribed species) from Atlántida (Dick 2013). This species was previously known from only three locations. Recently during the dry season it was mist netted over a stream adding a location in the department of Yoro.

###### Distribution.

Only Atlántida, Gracias a Dios, Olancho, and **Yoro** (Fig. 41); 0–470 m a.s.l.

###### Additional notes.

Historical records refer to *Tonatia saurophila* Koopman & Williams, 1951 (see Basantes et al. 2020; Turcios-Casco et al. 2020c, 2021b). Habitat loss due to deforestation for agriculture in the La Moskitia region is among the main threats. Global conservation status is NE and DD in Honduras.

#### *Phylloderma* Peters, 1865

We accept the new proposal of Camacho et al. (2026) and the species that should be present in Honduras should be *Phylloderma septentrionalis* Goodwin, 1940.

##### 107. Phylloderma septentrionalis

Taxon classificationAnimaliaChiropteraPhyllostomidae

Goodwin, 1940

21892091-BB56-5BF2-B896-7E3C8FF84005

###### Natural history.

A scrotal male was captured in February. Based on Medina-Fitoria et al. (2020), the presence of this species is indicative of intact forests.

###### Distribution.

Francisco Morazán, Gracias a Dios, and Olancho (Fig. 46); 200–1230 m a.s.l. This species may have been extirpated from Francisco Morazán, and its current occurrence may be only in eastern Honduras.

###### Additional notes.

Deforestation, poaching, and livestock-related impacts are the primary threats in Honduras. Global conservation status is LC and DD in Honduras, as *Phylloderma stenops* Peters, 1865.

#### *Phyllostomus* Lacépède, 1799

##### 108. Phyllostomus discolor

Taxon classificationAnimaliaChiropteraPhyllostomidae

(Wagner, 1843)

52FE6C37-F595-50D2-BE8B-D60D5ECAB908

###### Natural history.

Pregnant females have been captured during July, October, and November. Males with enlarged testes have been captured in October, November, and December. Lactating females have been captured in January, October, and November. Bats have been captured in banana and coffee plantations. We have encountered partial leucism in the dactylopatagium. Based on Dick (2013) and Goméz-Corea et al. (2022), it is host of four species of streblid bat flies: *Strebla hertigi* Wenzel, 1966 from Francisco Morazán and Valle, *Strebla wiedemanni* Kolenati, 1856 from Copán, Cortés, Gracias a Dios, Lempira, and Olancho; *Trichobioides perspicillatus* (Pessoa & Galvão, 1937) from Atlántida, Copán, Cortés, Francisco Morazán, and Valle; *Trichobius caecus* Edwards, 1918 from Lempira; *Trichobius costalimai* Guimarães, 1938 from Atlántida, Copán, Cortés, Francisco Morazán, and Valle; *Trichobius joblingi* Wenzel, 1966 from Gracias a Dios, Lempira, and Olancho; and *Trichobius parasiticus* Gervais, 1844 from Copán, Cortés, Gracias a Dios, Lempira, and Olancho. In addition, we report the occurrence of *T. costalimai* on *P. discolor* from Las Marías Pesh (Gracias a Dios) and El Ocotal (Francisco Morazán).

###### Distribution.

Atlántida, Choluteca, Comayagua, Copán, Cortés, El Paraíso, Francisco Morazán, Gracias a Dios, Intibucá, Lempira, La Paz, Ocotepeque, Olancho, Santa Bárbara, Valle, and Yoro (Fig. 59); 0–1050 m a.s.l.

###### Additional notes.

Global conservation status is LC and NE in Honduras.

##### 109. Phyllostomus hastatus

Taxon classificationAnimaliaChiropteraPhyllostomidae

(Pallas, 1767)

CB069830-CF67-517A-8D74-687D7B65E67D

###### Natural history.

Scrotal males have been captured in May, and lactating females have been captured in August. We have found individuals roosting in caves with *B. io*, *P. kappleri*, *Artibeus* Leach, 1821 and *Glossophaga* spp. (Divoll and Buck 2013). It is the host of two species of streblid bat flies: *Mastoptera guimaraesi* Wenzel, 1966, recorded from Atlántida; *Trichobius longipes* (Rudow, 1871) from Atlántida and Cortés (Dick 2013). Herein, we also report the occurrence of *Strebla hertigi* Wenzel, 1966, *T. longipes*, and *M. guimaraesi* from Las Marías Pesh (Gracias a Dios) and *T. longipes* from Cuevas de Taulabé (Comayagua).

###### Distribution.

Atlántida, Colón, Comayagua, Cortés, El Paraíso, Francisco Morazán, Gracias a Dios, La Paz, Olancho, Santa Bárbara, and Yoro (Fig. 54); 50–1600 m a.s.l.

###### Additional notes.

Global conservation status is LC and NE in Honduras.

#### *Chrotopterus* Peters, 1865

##### 110. Chrotopterus auritus

Taxon classificationAnimaliaChiropteraPhyllostomidae

(Peters, 1856)

8DDD453A-941A-5082-B773-A6E1C438B7A1

###### Natural history.

They have been captured in bamboo thickets. A male and a female were captured simultaneously above a stream. A single species of the streblid bat fly *Strebla chrotopteri* Wenzel, 1976, was documented on an individual captured in Lempira (Dick 2013).

###### Distribution.

**Figure 61.**
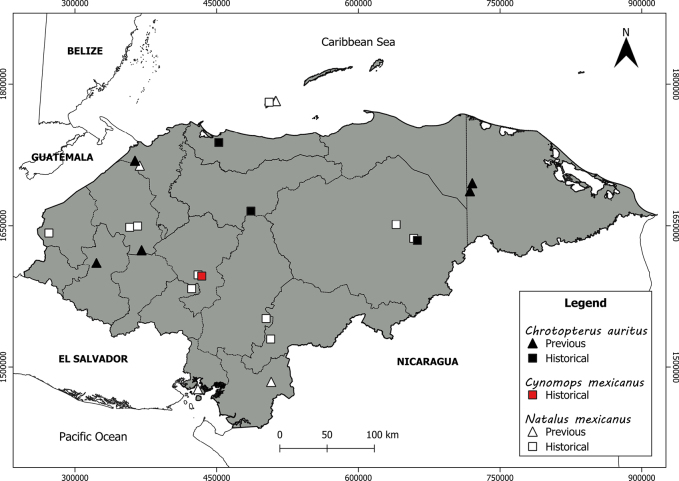
Distribution of *Chrotopterus auritus*, *Cynomops mexicanus*, and *Natalus mexicanus* in Honduras.

Atlántida, Cortés, Gracias a Dios, Lempira, Olancho, Santa Bárbara, and Yoro (Fig. 61); 220–1550 m a.s.l. Perdomo and Vega (2019) recorded the highest elevational record.

###### Additional notes.

Habitat loss due to agricultural deforestation in the La Moskitia region is among the main threats to its distribution. Global conservation status is LC and VU in Honduras.

#### *Mimon* Gray, 1847

##### 111. Mimon cozumelae

Taxon classificationAnimaliaChiropteraPhyllostomidae

Goldman, 1914

A560699B-F6DC-5A10-99EE-AC11A5472EBE

###### Natural history.

A male and a female were captured simultaneously over a stream in Lancetilla, Atlántida. It is the host of three species of streblid bat flies: *Speiseria ambigua* Kessel,1925, *Trichobius dugesii* Townsend, 1891, and *Trichobius uniformis* Curran, 1935 from Atlántida (Dick 2013).

###### Distribution.

Atlántida, Colón, and Olancho (Fig. 55); 40–1000 m a.s.l.

###### Additional notes.

Loss of its habitat due to deforestation is the major threat to the species in eastern Honduras (Ávila-Palma et al. 2019). Previous records of this species for Honduras may be referred to as *Mimon bennettii* (Gray, 1838). Global conservation status is LC and VU in Honduras.

#### *Vampyrum* Rafinesque, 1815

##### 112. Vampyrum spectrum

Taxon classificationAnimaliaChiropteraPhyllostomidae

(Linnaeus, 1758)

423C771A-D8A6-5F13-BEA3-C2AA598DB7FD

###### Natural history.

Dinets (2017) recorded a solitary individual from a cave in the Islas de la Bahía department, observed for less than 10 minutes before it flew out of the cave; this record was cited by Martínez Murillo et al. (2021). This species was recorded using camera traps in Río Zacate, Pico Bonito National Park (Atlántida).

###### Distribution.

Atlántida, Gracias a Dios, Islas de la Bahía, and Olancho (Fig. 59); 115–505 m a.s.l. It is now considered locally extinct from the Islas de la Bahía department (Martínez Murillo et al. 2021).

###### Additional notes.

Mejía-Quintanilla et al. (2020) included a photo of *V. spectrum*, which was killed after entering a house, and was covered with salt in the mistaken association of bats and demons. We requested additional information from the Miskitus, and they reported that, after killing a bat (sakanki in Miskitus), demons may enter the body, so they cover the dead bat with salt to prevent the demons from emerging. Global conservation status is NT and VU in Honduras.

#### *Glossophaga* É. Geoffroy Saint-Hilaire, 1818

##### 113. Glossophaga commissarisi

Taxon classificationAnimaliaChiropteraPhyllostomidae

Gardner, 1962

A3966DB5-1AE3-5787-86B9-E160A0E3EDD7

###### Natural history.

Seeds found in feces include *Solanum erianthum* D. Don in February and *Muntingia calabura* L. in November from bats captured in an urban area in central Honduras (Turcios-Casco et al. 2019b, 2021b). Lactating females have been captured in July. Scrotal males have been captured in February. Pregnant females have been captured in August. Three species of bat flies have been recorded on *G. commissarisi*: from Comayagua, *Paraeuctenodes longipes* Pessôa & Guimarães, 1937. *Megistopoda proxima* (Séguy, 1926) and *Trichobius intermedius* Peterson & Hürka, 1974 (Dick 2013). We report an additional streblid, *M. proxima*, on a bat captured at the Ciudad Universitaria (Francisco Morazán).

###### Distribution.

**Figure 62.**
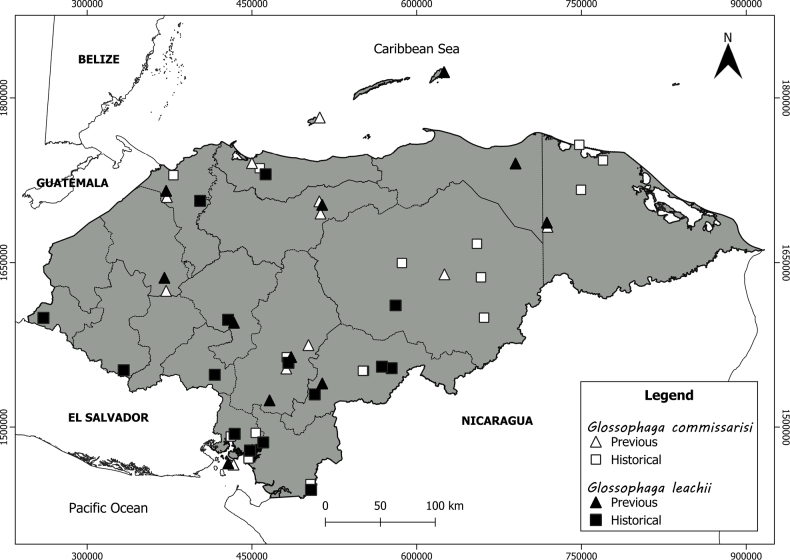
Distribution of *Glossophaga commissarisi* and *G. leachii* in Honduras.

Atlántida, Choluteca, Copán, El Paraíso, Francisco Morazán, Gracias a Dios, Islas de la Bahía, Olancho, Santa Bárbara, Valle, and Yoro (Fig. 62); 200–1080 m a.s.l.

###### Additional notes.

Global conservation status is LC and NE in Honduras.

##### 114. Glossophaga leachii

Taxon classificationAnimaliaChiropteraPhyllostomidae

(Gray, 1844)

173FF578-8F14-53D4-8B29-BD4C02E7ABC2

###### Natural history.

Lactating females have been captured in November. Scrotal males have been captured in January (Turcios-Casco et al. 2021c). Pregnant females have been captured in October (two embryos). It is host to *Trichobius dugesii* Townsend, 1891 from Valle (Dick 2013). Seeds of *M. calabura* have been recorded in feces of adult males in November in an urban area in central Honduras (Turcios-Casco et al. 2019b).

###### Distribution.

Colón, Comayagua, Cortés, El Paraíso, Francisco Morazán, Gracias a Dios, Islas de la Bahía, Lempira, Ocotepeque, Olancho, Valle, and Yoro (Fig. 62); 250–1070 m a.s.l.

###### Additional notes.

Global conservation status is LC and NE in Honduras.

##### 115. Glossophaga mutica

Taxon classificationAnimaliaChiropteraPhyllostomidae

Merriam, 1898

F856A310-9025-59B3-B54D-F8AD98198AD3

###### Natural history.

It has been observed above water bodies, in dry streambeds, and in mango plantations. Seeds of *S. erianthum* and *M. calabura* were found in the feces of adult males captured in urban areas in November (Turcios-Casco et al. 2019b). Based on Medina-Van Berkum et al. (2020) and Martínez Murillo et al. (2021), and our records, pregnant females have been captured in March (one embryo), April, May, and October. Lactating females have been captured in April, July, August, October, and November. Scrotal males have been captured in January, March, April, June, and November. It is host of 10 species of streblid bat flies *Aspidoptera phyllostomatis* (Perty, 1833) from Atlántida; *Anastrebla modestini* Wenzel, 1966 from Francisco Morazán; *Megistopoda aranea* (Coquillett, 1899) from Atlántida; *Megistopoda proxima* (Séguy, 1926) from Olancho; *Paraeuctenodes longipes* Pessôa & Guimarães, 1937 from Olancho; *Strebla curvata* Wenzel, 1976 from Atlántida, Comayagua e Olancho; *Strebla guajiro* (García & Casal, 1965) from Olancho; *Trichobius dugesii* Townsend, 1891 from Atlántida, Comayagua, Isla de Bahía, Lempira, Olancho, and Valle; *Trichobius galei* Wenzel, 1966 from Valle; *Trichobius uniformis* Curran, 1935 from Atlántida, Comayagua, Olancho, and Valle; *T. dugesii* from Atlántida, Comayagua, Lempira, Islas de la Bahía, Olancho, and Valle; *T. uniformis* from Atlántida, Comayagua, Olancho, and Valle (Dick 2013; Miller 2014). In addition, on *G. mutica*, we report the occurrence of *T. dugesii* from El Hatillo and Ciudad Universitaria (Franscisco Morazán) and Islas de la Bahía, *Aspidoptera phyllostomatis* from El Ocotal (Francisco Morazán), and *M. proxima* from Ciudad Universitaria (Francisco Morazán).

###### Distribution.

All the departments of Honduras except Colón (Fig. 58), occurring at elevations ranging from 0 to 2010 m a.s.l.

###### Additional notes.

*G. mutica* is recognized as distinct from *Glossophaga soricina* (Pallas, 1766). Calahorra-Oliart et al. (2021) records formerly identified as *G. soricina* apply to this species. Global conservation status is LC and NE in Honduras, both referring to *G. soricina*.

#### *Leptonycteris* Lydekker, 1891

##### 116. Leptonycteris yerbabuenae

Taxon classificationAnimaliaChiropteraPhyllostomidae

Martínez & Villa-R, 1940

079552E2-3D9C-5980-A5B8-FE95871973A8

###### Natural history.

Mora et al. (2020) noted that this species may be common in certain regions of Honduras; for example, they captured ten individuals in La Anonilla, Choluteca department, suggesting a resident population.

###### Distribution.

**Figure 63.**
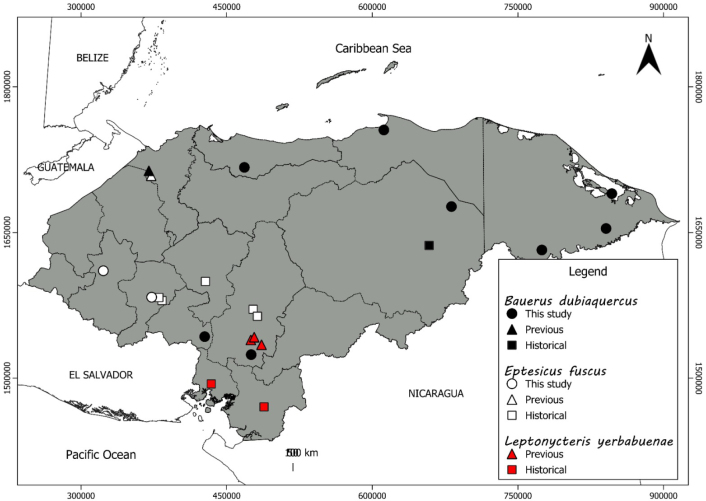
Distribution of *Bauerus dubiaquercus*, *Eptesicus fuscus*, and *Leptonycteris yerbabuenae* in Honduras.

Choluteca, Francisco Morazán, and Valle (Fig. 63); 1480–1710 m a.s.l. Mora et al. (2020) reported a record on iNaturalist from the El Paraíso department (https://www.inaturalist.org/observations/41662024); however, there were no confirming details to support its validity.

###### Additional notes.

Mora et al. (2020) suggested that protection of dry forest habitats is important for maintaining population viability. Historical records of this species have been referred to as *Leptonycteris curasoae* Miller, 1900. Global conservation status is NT and EN in Honduras.

#### *Anoura* Gray, 1838

##### 117. Anoura peruana

Taxon classificationAnimaliaChiropteraPhyllostomidae

(Tschudi, 1844)

F4006BBF-CF92-5EFE-A927-0BBB8239EE87

###### Natural history.

They are known to roost in caves (Turcios-Casco et al. 2020d). Pregnant females and reproductive males have been captured in May. It is the host of *Exastinion clovisi* (Pessôa & Guimarães, 1937) in individuals captured from Comayagua and Francisco Morazán (Dick 2013). In addition, we report the occurrence of *E. clovisi* on *A. peruana* from Cerro Azul (Comayagua).

###### Distribution.

**Figure 64.**
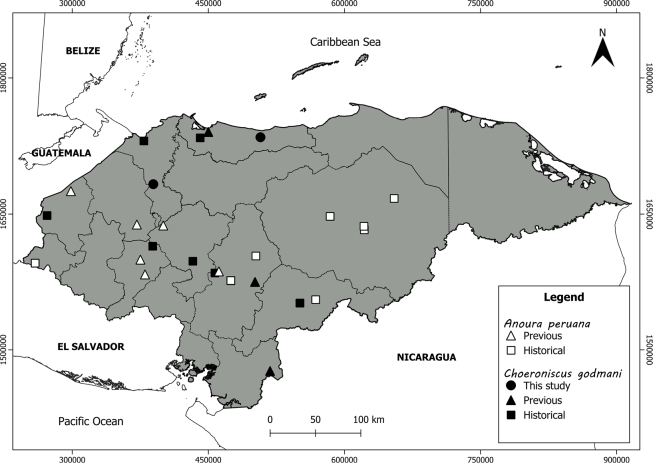
Distribution of *Anoura peruana* and *Choeroniscus godmani* in Honduras.

Comayagua, Copán, El Paraíso, Francisco Morazán, Intibucá, Ocotepeque, Olancho, and Santa Bárbara (Fig. 64); 825–1910 m a.s.l.

###### Additional notes.

In recent years, the agreed-upon name for this taxon has been in flux. Calderón-Acevedo et al. (2022) noted that morphological and molecular analyses to distinguish *A. geoffroyi* Gray, 1838 from *A. peruana*, thereby reestablishing the name of populations in Central America as *A. geoffroyi*. However, a more recent review (Molinari et al. 2023) clarified that the northern Central American and Mexican species is distinct as *Anoura peruana lasiopyga* (Peters, 1868). Therefore, here we treat all historical records of *A. geoffroyi* from Honduras as *A. peruana*. Global conservation status is LC and NT in Honduras, both referring to *A. geoffroyi*.

#### *Hylonycteris* Thomas, 1903

##### 118. Hylonycteris underwoodi

Taxon classificationAnimaliaChiropteraPhyllostomidae

Thomas, 1903

62EBF06D-CC8F-57F5-88B5-B4EDEB69DA06

###### Natural history.

This was among the most common species in the Cusuco National Park, with 112 individuals captured across all elevation ranges surveyed (Medina-Van Berkum et al. 2020). Otherwise, poorly known in the rest of the country.

###### Distribution.

Atlántida, Cortés, and Gracias a Dios (Fig. 23); 230–1900 m a.s.l. The occurrences in Olancho and Santa Bárbara must be confirmed.

###### Additional notes.

Deforestation, land-use changes, and livestock ranching are among the threats across its range (Turcios-Casco and Medina-Fitoria 2019). *H. underwoodi* is often confused with other glossophagines. Global conservation status is LC and DD in Honduras.

#### *Choeroniscus* Thomas, 1928

##### 119. Choeroniscus godmani

Taxon classificationAnimaliaChiropteraPhyllostomidae

(Thomas, 1903)

45CCE386-93CA-58F2-8379-1DDD3FA9E0E7

###### Natural history.

Pregnant females were captured in October, and lactating females in November. *Cecropia* Loefl. Seeds were found in the feces and fur of adult females in October.

###### Distribution.

Atlántida, Choluteca, Comayagua, **Cortés**, El Paraíso, Francisco Morazán, El Paraíso, and Intibucá (Fig. 64); 320–980 m a.s.l.

###### Additional notes.

Global conservation status is LC and DD in Honduras.

#### *Choeronycteris* Tschudi, 1844

##### 120. Choeronycteris mexicana

Taxon classificationAnimaliaChiropteraPhyllostomidae

Tschudi, 1844

8EF1ADB2-B7CD-5E8C-AAB9-D01D1D16DD85

###### Natural history.

There is no information regarding the ecology or natural history of this species in Honduras. A single individual was captured at Francisco Morazán (van Dort et al. 2022).

###### Distribution.

Cortés and Francisco Morazán (Fig. 60); 870–1715 m a.s.l.

###### Additional notes.

Global conservation status is NT and DD in Honduras.

#### *Lichonycteris* Thomas, 1895

##### 121. Lichonycteris obscura

Taxon classificationAnimaliaChiropteraPhyllostomidae

Thomas, 1895

A07E3C8D-133C-58DF-9040-7435DCAC59F9

###### Natural history.

They were reported to feed on Poha Berries (*Physalis peruviana* L.) in plantations. It has not been recorded in Honduras since 1971.

###### Distribution.

Only known from the Olancho department (Fig. 49); 505 m a.s.l.

###### Additional notes.

Global conservation status is LC and DD in Honduras.

#### *Carollia* Gray, 1838

##### 122. Carollia castanea

Taxon classificationAnimaliaChiropteraPhyllostomidae

H. Allen, 1890

650CD244-F353-5768-8768-8810B27FA3D9

###### Natural history.

This species was mainly captured in villages within the buffer zone of the Cusuco National Park at lower altitudes (Medina-Van Berkum et al. 2020). Reproductive males have been captured in May and June, and lactating females in June. It is a host of *Trichobius joblingi* Wenzel, 1966, based on specimens captured in Atlántida (Dick 2013). In addition, *T. joblingi* was found on an individual from Las Marías Pech (Gracias a Dios).

###### Distribution.

**Figure 65.**
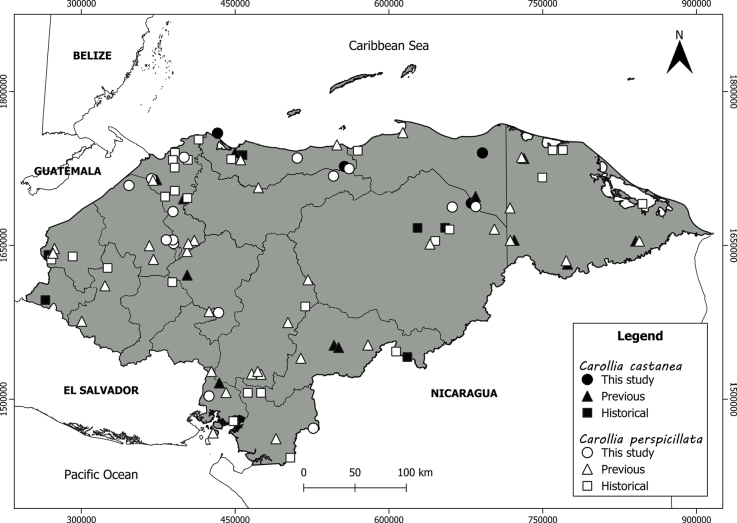
Distribution of *Carollia castanea* and *C. perspicillata* in Honduras.

Atlántida, **Colón**, **Comayagua**, **Cortés**, El Paraíso, Francisco Morazán, Gracias a Dios, Olancho, Santa Bárbara, and Valle (Fig. 65); 40–700 m a.s.l.

###### Additional notes.

However, Simmons and Cirranello (2026) note that this taxon is a species complex comprising two overlapping clades of *C. castanea*, and the distributional limits remain unclear. Global conservation status is LC and NE in Honduras.

##### 123. Carollia subrufa

Taxon classificationAnimaliaChiropteraPhyllostomidae

(Hahn, 1905)

08A91B16-A576-5DE5-AB15-8C43FAA2E779

###### Natural history.

Pregnant females have been captured in July, and lactating females in October. It is a host of *Trichobius joblingi* Wenzel, 1966 on *C. subrufa* from Atlántida (Dick 2013).

###### Distribution.

**Figure 66.**
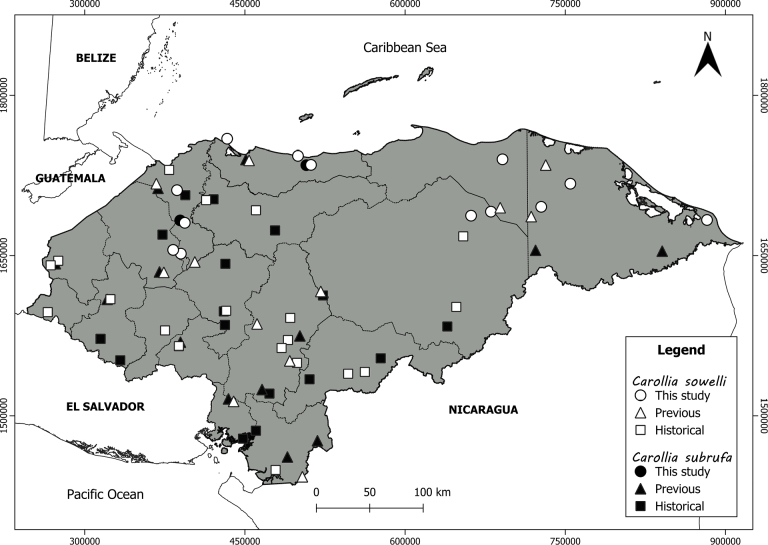
Distribution of *Carollia subrufa* and *C. sowelli* in Honduras.

Atlántida, Choluteca, Comayagua, Copán, Cortés, El Paraíso, Francisco Morazán, Gracias a Dios, La Paz, Lempira, Olancho, Santa Bárbara, Valle, and Yoro (Fig. 66); 230–1220 m a.s.l.

###### Additional notes.

Global conservation status is LC and NE in Honduras.

##### 124. Carollia sowelli

Taxon classificationAnimaliaChiropteraPhyllostomidae

Baker, Solari & Hoffmann, 2002

335247CF-8079-5560-9E1E-787736E2133A

###### Natural history.

It has been captured in corn fields, caves, caverns, and in primary and secondary forests. Reproductive males were captured in April, May, and November, and pregnant females were captured in September (one or two embryos). Based on Dick (2013), it is the host of six species of streblid bat flies: *Metelasmus pseudopterus* Coquillett, 1907, *Speiseria ambigua* Kessel, 1925, *Strebla guajiro* (García & Casal, 1965) and *Trichobius sparsus* Kessel, 1925 from Atlántida; *Trichobius joblingi* Wenzel, 1966 from Atlántida, Comayagua, and Olancho; and *Speiseria peytonae* Wenzel, 1976 from Lempira.

###### Distribution.

Atlántida, Choluteca, Colón, Comayagua, Copán, Cortés, El Paraíso, Francisco Morazán, Gracias a Dios, Lempira, Intibucá, La Paz, Lempira, Ocotepeque, Olancho, Santa Bárbara, Valle, and Yoro (Fig. 66); 40–1800 m a.s.l.

###### Additional notes.

Historical records of this species have been referred to as *Carollia brevicauda* (Schinz, 1821). Global conservation status is LC and NE in Honduras.

##### 125. Carollia perspicillata

Taxon classificationAnimaliaChiropteraPhyllostomidae

(Linnaeus, 1758)

DFD10277-6EFA-5369-8CA3-65FB1D8E77AB

###### Natural history.

It has been captured over dry streambeds, altered and primary forests (Medina-Van Berkum et al. 2020), coffee plantations, caverns and caves, old houses, and buildings (the last four used as roosts). Females with offspring were captured in August. Pregnant females have been captured in March, September, and November, and lactating females in March, October, and November. Active males were in April, June, July, August, September, and November. The species is host to *Strebla guajiro* (García & Casal, 1965) and *Trichobius joblingi* Wenzel, 1966 from individuals captured in Atlántida (Dick 2013). In addition, we also report *T. joblingi* on *C. perspicillata* from El Ocotal (Francisco Morazán).

###### Distribution.

Atlántida, Choluteca, Colón, Comayagua, Copán, Cortés, El Paraíso, Francisco Morazán, Gracias a Dios, Intibucá, La Paz, Lempira, Olancho, Santa Bárbara, and Valle Yoro. (Fig. 65); 30–980 m a.s.l.

###### Additional notes.

Global conservation status is LC and NE in Honduras.

#### *Glyphonycteris* Thomas, 1896

##### 126. Glyphonycteris daviesi

Taxon classificationAnimaliaChiropteraPhyllostomidae

(Hill, 1965)

E88F4E4F-D571-5CEB-9B53-566404219BFB

###### Natural history.

D. C. Carter captured an individual of unknown sex of *G. daviesi* (referred to as *Barticonycteris daviesi* J. Edwards Hill, 1964) on the Perlas River, where a trail to Valencia on the Patuca River crosses the river near Catacamas, Olancho (see McCarthy et al. 1993) in 1967. No reproductive activity has been reported for the species in Honduras.

###### Distribution.

Only known from the Olancho department (Fig. 45); 500 m a.s.l.

###### Additional notes.

Richard K. LaVal captured the bat while netting with D. C. Carter. Unfortunately, the bat escaped after chewing a hole in the bag in which it was held. The live bat was photographed by LaVal, and the photograph was stored in the Mammal Slide Library (no. 378) of the American Society of Mammalogists (https://www.mammalogy.org/committees/mammal-images-library). No voucher specimens are known from Honduras. Global conservation status is LC and DD in Honduras.

##### 127. Glyphonycteris sylvestris

Taxon classificationAnimaliaChiropteraPhyllostomidae

Thomas, 1896

6FB8A627-A5C7-5F5F-9E8B-C24A0C7D8933

###### Natural history.

McCarthy et al. (1993) noted that B. H. Gaskell collected six specimens (five males and one male) of *Micronycteris* (Glyphonycteris) sylvestris (Sanborn, 1949) (= *G. sylvestris*) from Laguna de Bacalar, Gracias a Dios department, in 1982. There is no reproductive information from Honduras.

###### Distribution.

Only Gracias a Dios department (Fig. 32); 0 m a.s.l.

###### Additional notes.

A recent record of this species was made in 1998 during a survey in Sierra de Agalta National Park. Global conservation status is LC and DD in Honduras.

#### *Artibeus* Leach, 1821

##### 128. Artibeus inopinatus

Taxon classificationAnimaliaChiropteraPhyllostomidae

Davis & Carter, 1964

A94B7D5D-8A01-59E3-A0B7-D854F5172BC2

###### Natural history.

They have been captured in dry steam beds, streams, under fig trees (*Ficus* sp.) and mango trees (*Mangifera indica* L.), near abandoned mines, active mines, and pine forests: *Pinus oocarpa* Schiede ex Schltdl. and *Pinus maximinoi* H. E. Moore are abundant, as are thorn-scrub habitats. Pregnant females have been captured in March, May, July, September, and November. Lactating females have been captured in March, June, August, October, November, and December. Post-lactating females have been captured in March. Reproductive males have been captured in March, May, August, October, and December. Seeds of the trumpet tree (*Cecropia peltata* L.) have been found in feces and in their fur in October from adult females. It is the host of *Trichobius intermedius* Peterson & Hurka, 1974, from individuals captured in Valle (Dick 2013). In addition, we found an undescribed species of *Megistopoda* Macquart, 1852 on individuals of *A. inopinatus* captured in El Ocotal (Francisco Morazán). We have noticed partial leucism of the uropatagium in post-lactating females.

###### Distribution.

**Figure 67.**
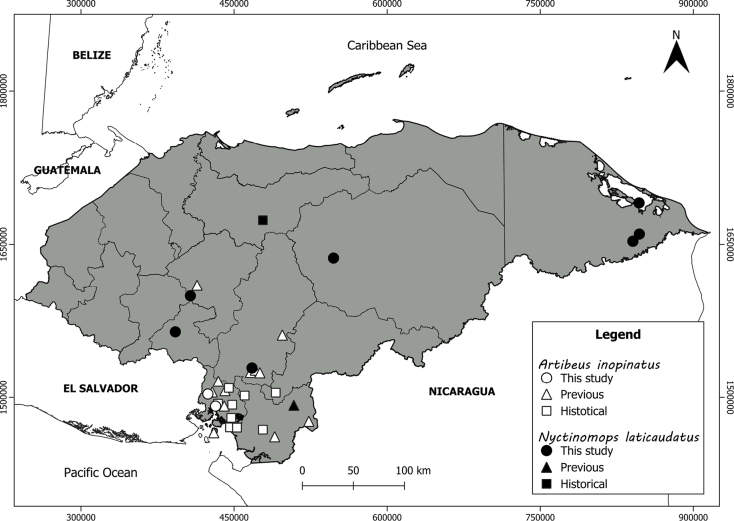
Distribution of *Artibeus inopinatus* and *Nyctinomops laticaudatus* in Honduras.

Choluteca, Comayagua, El Paraíso, Francisco Morazán, and Valle (Fig. 67); 30–1435 m a.s.l.

###### Additional notes.

Sabanagrande in central Honduras appears to be the most important region for the conservation of the species as it is very common there (Turcios-Casco et al. 2020a, 2020e, 2020f, 2021b), but rare throughout its range (Davis and Carter 1964). Global conservation status is DD and VU in Honduras.

##### 129. Artibeus intermedius

Taxon classificationAnimaliaChiropteraPhyllostomidae

J.A. Allen, 1897

70F573E1-CBB2-5526-A3F6-E7B6F9FBFA60

###### Natural history.

One of the most widely distributed species, although often confused with *Artibeus lituratus* (Olfers, 1818), its natural history is poorly known. It is the host of four species of streblid bat flies: *Megistopoda aranea* (Coquillett, 1899) from Olancho, *Paratrichobius longicrus* (Miranda-Ribeiro, 1907), *Trichobius intermedius* Peterson & Hurka, 1974, and *Trichobius parasiticus* Gervais, 1844 from Comayagua (Dick 2013).

###### Distribution.

**Figure 68.**
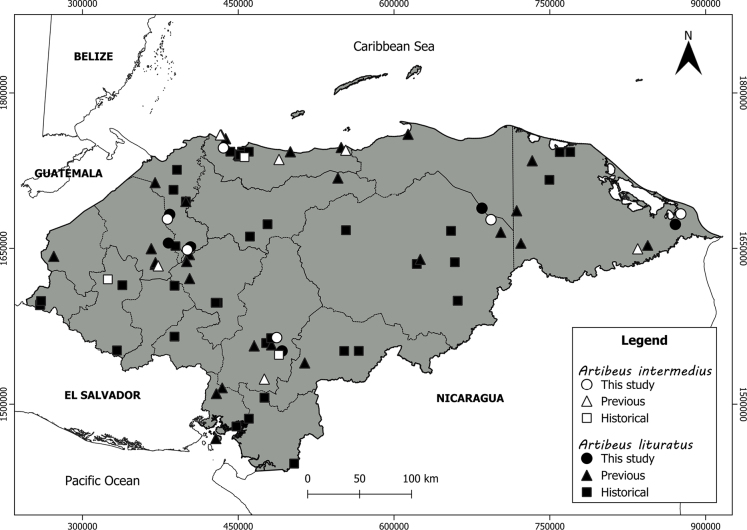
Distribution of *Artibeus intermedius* and *A. lituratus* in Honduras.

Atlántida, Cortés, Francisco Morazán, Gracias a Dios, Lempira, Olancho, and Santa Bárbara (Fig. 68); 10–625 m a.s.l.

###### Additional notes.

Historical specimens have been referred to as *Artibeus lituratus intermedius* J. A. Allen, 1897. Several specimens in collections should be carefully reviewed. Global and national conservation status is NE.

##### 130. Artibeus jamaicensis

Taxon classificationAnimaliaChiropteraPhyllostomidae

Leach, 1821

D837B8C9-CEB3-5C4C-B6E8-8BC4D1004911

###### Natural history.

This was reported as one of the dominant species captured, with an average of 80 individuals per year during long-term surveys in Cusuco National Park (Medina-Van Berkum et al. 2020). This species appears to be reproductively active year-round. In addition to Davis and Carter (1964), Medina-Van Berkum et al. (2020), and Turcios-Casco et al. (2019b, 2021b), pregnant females have been captured during January (one embryo), February, March (one embryo), May, October, and December with lactating females found in January, April, May, July, October, November, and December. Active males have been captured in March, April, May, July, October, November, and December. It has been recorded in mango and coffee plantations, above dry streambeds and water bodies in urban areas (Turcios-Casco et al. 2019b), and in both altered and primary forests (Medina-Van Berkum et al. 2020). Seeds of *Psidium guajava* L. and *S. erianthum* were found in feces in March, while *M. calabura* and *S. erianthum* were recorded in December in adult females from urban areas of central Honduras (Turcios-Casco et al. 2019c). Individuals of both sexes captured in Atlántida and Francisco Morazán in October carried *Ficus* fruits and *C. peltata* seeds. Additional records include *Solanum* sp. seeds in December (Santa Bárbara) and direct observations of fruit consumption of *P. guajava*, *Syzygium cumini* (L.) Skeels, *Terminalia catappa* L., and *M. indica*. In the Caribbean islands of Honduras, consumption of *Spondias mombin*, *T. catappa*, *Carica papaya*, and *C. peltata* has also been reported (Bermingham et al. 1998; Martínez-Murillo et al. 2021). In urban and rural areas, Araceae species, ornamental plants, are used as tents, but in the Caribbean islands, they prefer the palms *Acoelorrhaphe wrightii* and *Sabal* sp., and old *Dermanura phaeotis* G. S. Miller, 1902 tents. Martínez-Murillo et al. (2021) reported individuals with a smaller forearm (50.0 mm) in Útila. We doubt that these specimens are *A. jamaicensis* due to the extremely small forearms, which put it into the lower part of the size range of the smaller species. *A. inopinatus*. External parasites include one species of spinturnicid mite *Periglischrus iheringi* Oudemans, 1902 from Francisco Morazán and eight species of streblid bat flies: *Strebla matsoni* Wenzel, 1976 from Santa Barbara (Graciolli et al. 2021); *Aspidoptera phyllostomatis* (Perty, 1833), *Metelasmus pseudopterus* Coquillett, 1907 and *Trichobius uniformis* Curran, 1935 from Atlántida; *Trichobius intermedius* Peterson & Hurka, 1974 from Lempira; *M. aranea* and *Trichobius angulatus* Wenzel, 1976 from Islas de la Bahía (Dick 2013; Miller 2014). In addition, we report herein an undescribed species of *Megistopoda* Macquart, 1852 from El Ocotal in Francisco Morazán (same species as *A. inopinatus*) and *M. pseudopterus* from Ceguaca (Santa Bárbara); *Paratrichobius longicrus* (Miranda-Ribeiro, 1907) from El Ocotal (Francisco Morazán); *P. longicrus* and *T. intermedius* from Ciudad Universitaria (Francisco Morazán).

###### Distribution.

**Figure 69.**
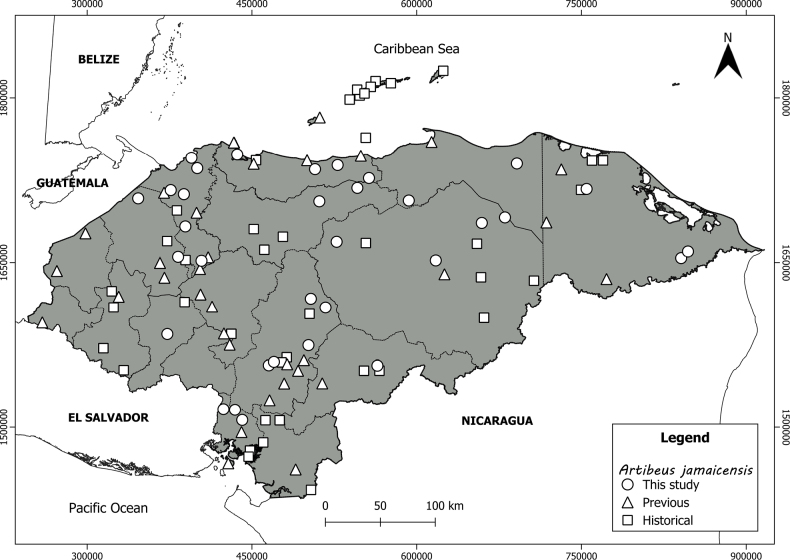
Distribution of *Artibeus jamaicensis* in Honduras.

In the 18 departments of Honduras (Fig. 69); 25–1475 m a.s.l.

###### Acoustic identification.

This is one of the most frequently recorded phyllostomid species, usually as hand-released bats. Call shapes are broadband, multiharmonic, decreasing FM pulses, typical of the family. Recordings have been primarily hand-released individuals and not free-flying (Miller et al. 2024).

###### Additional notes.

This species is highly adapted to urban environments and is generally the phyllostomid bat most commonly involved in human–wildlife conflicts, as it frequently roosts in houses and is often killed due to local superstitions. Global conservation status is LC and NE in Honduras.

##### 131. Artibeus lituratus

Taxon classificationAnimaliaChiropteraPhyllostomidae

(Olfers, 1818)

9963C7C0-BD96-586D-9F96-BD5304AE0C47

###### Natural history.

This species has been captured above and near streams (20–100 m), in mango and coffee plantations, in dry streambeds and water bodies, in urban areas, and in both altered and primary forests. It has been reported that seeds of *S. erianthum* have been found in feces in October, November, and December (up to 71 seeds) in an urban area near Tegucigalpa, and up to 516 seeds of *M. calabura* (capulín) in a single fecal sample (Turcios-Casco et al. 2019b). In June, we recorded seeds of *C. peltata* from adult males in Olancho. This species appears to be reproductively active all year. Reproductive males have been captured in February, March, April, May, September, and November. Pregnant females have been captured in February, March (one embryo), April, May, June, and September. Lactating females were captured in February, April, March, July, September, October, November, and December, with post-lactating females captured in March. Dick (2013) recorded eight Streblidae on *A. lituratus. Aspidoptera phyllostomatis* (Perty, 1833), *Megistopoda aranea* (Coquillett, 1899), *M. proxima* (Séguy, 1926), *Metelasmus pseudopterus* Coquillett, 1907, *Strebla curvata* Wenzel, 1976, and *S. guajiro* (García & Casal, 1965) from Atlántida; *Paratrichobius longicrus* (Miranda-Ribeiro, 1907) from Atlántida and Valle; *Trichobius intermedius* Peterson & Hurka, 1974 from Comayagua and Valle. He considered contaminants *M. proxima*, *S. curvata*, and *S. guajiro*. Rudnick (1960) reported a spinturnid mite *Periglischrus iheringi* Oudemans, 1902 on *A. lituratus* from Atlántida. In addition, for *A. lituratus*, we report the occurrence of *Paratrichobius dunni* (Curran, 1935) from Ciudad Universitaria (Francisco Morazán) and *P. longicrus* from Ciudad Universitaria (Francisco Morazán) and Las Marías Pech (Gracias a Dios).

###### Distribution.

Occurs in all departments except for Islas de la Bahía (Fig. 68); 30–1750 m a.s.l.

###### Acoustic identification.

One of the few species in the family to have been recorded. Call shapes are typical of Phyllostomidae and consist of broad-band, multiharmonic, decreasing-FM pulses, with bats recorded as free-flying individuals (Miller et al. 2024).

###### Additional notes.

Global conservation status is LC and NE in Honduras.

#### *Dermanura* Gervais, 1856

##### 132. Dermanura azteca

Taxon classificationAnimaliaChiropteraPhyllostomidae

(K. Andersen, 1906)

06087770-2D0E-57F1-A178-3B2291C787EB

###### Natural history.

It has been found roosting in Arecaceae palms. This species has been captured along riverbanks.

###### Distribution.

**Figure 70.**
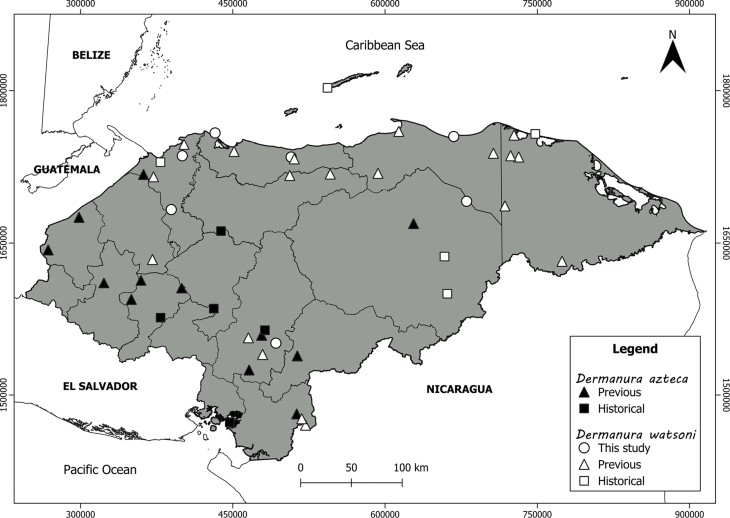
Distribution of *Dermanura azteca* and *D. watsoni* in Honduras.

Choluteca, Comayagua, Copán, Cortés, El Paraíso, Francisco Morazán, Intibucá, Lempira, Olancho, and Valle (Fig. 70); 0–1520 m a.s.l.

###### Additional notes.

Global conservation status is LC and NE in Honduras.

##### 133. Dermanura phaeotis

Taxon classificationAnimaliaChiropteraPhyllostomidae

Miller, 1902

324C9528-7155-5FFB-AD45-40CAFE37D53D

###### Natural history.

On the Caribbean islands, it roosts in the Paurotis Palm (*Acoelorrhaphe wrightii* Griseb. and H. Wendl.), using leaves as tents (Martínez-Murillo et al. 2021). This species has been captured above and near streams, as well as primary and secondary forests. Pregnant females have been captured with two offspring in August. Active males have been captured in June. Dick (2013) reported three species of streblid bat flies from Atlántida: *Neotrichobius stenopterus* Wenzel & Aitken, 1966, and two species considered accidental or transitory occurrences, *Megistopoda proxima* (Séguy, 1926) and *Paratrichobius dunni* (Curran, 1935).

###### Distribution.

**Figure 71.**
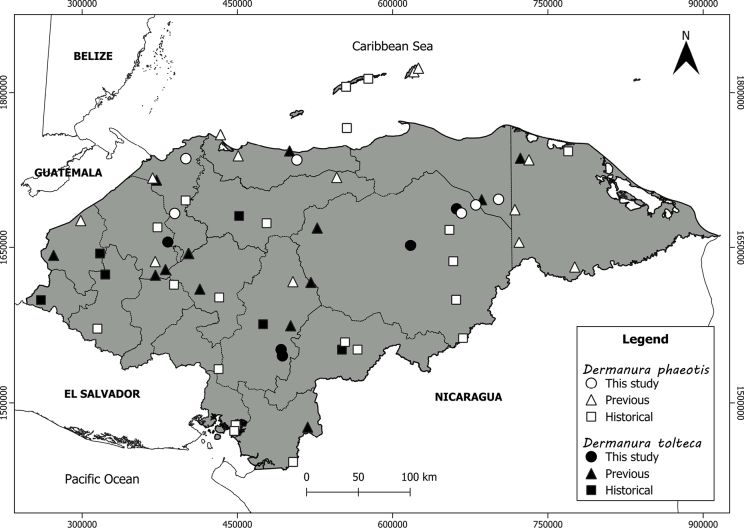
Distribution of *Dermanura phaeotis* and *D. tolteca* in Honduras.

Atlántida, Choluteca, Comayagua, Copán, Cortés, El Paraíso, Francisco Morazán, Gracias a Dios, Intibucá, Islas de la Bahía, Olancho, Santa Bárbara, Valle, and Yoro (Fig. 71); 225–790 m a.s.l.

###### Additional notes.

Global conservation status is LC and NE in Honduras.

##### 134. Dermanura tolteca

Taxon classificationAnimaliaChiropteraPhyllostomidae

(Saussure, 1860)

C69B407E-9C2E-57B8-91C0-7E3616EEC171

###### Natural history.

Reproductive males have been captured in March and April. Pregnant females have been captured in April, with lactating females in March, April, and May. It is a host of *Neotrichobius stenopterus* Wenzel & Aitken, 1966 from an individual captured in Atlántida (Dick 2013).

###### Distribution.

Atlántida, Choluteca, Colón, Comayagua, Copán, Cortés, El Paraíso, Francisco Morazán, Gracias a Dios, Lempira, Ocotepeque, Olancho, Santa Bárbara, Valle, and Yoro (Fig. 71); 220–1480 m a.s.l.

###### Additional notes.

Based on Simmons and Cirranello (2026), the correct epithet is tolteca, not toltecus. Global conservation status is LC and NE in Honduras.

##### 135. Dermanura watsoni

Taxon classificationAnimaliaChiropteraPhyllostomidae

(Thomas, 1901)

3B3908A8-199D-5EB7-8C38-06F7AA4B9EF2

###### Natural history.

Reproductive males have been captured in November. Males captured in Santa Bárbara in December had *M. calabura* seeds in their feces.

###### Distribution.

Atlántida, Choluteca, Colón, Copán, Cortés, Francisco Morazán, Gracias a Dios, Islas de la Bahía, Lempira, Olancho, Santa Bárbara, and Yoro (Fig. 70); 40–1350 m a.s.l. Martínez-Murillo et al. (2021) did not find *D. watsoni* in Islas de la Bahía, suggesting that the species may be extirpated in these islands.

###### Additional notes.

Global conservation status is LC and NE in Honduras.

#### *Chiroderma* Peters, 1860

##### 136. Chiroderma gorgasi

Taxon classificationAnimaliaChiropteraPhyllostomidae

Handley, 1960

BABCD59C-FA5F-51FF-AC86-89F49DE39E62

###### Natural history.

There are only two records, one of which escaped after body mass and forearm measurements were recorded. Both were from the core zone of the Río Plátano Biosphere Reserve Gracias a Dios (Turcios-Casco et al. 2020f). There is no reproductive information. Both specimens were captured above a creek.

###### Distribution.

Only the Gracias a Dios department at 250 m a.s.l. (Fig. 15). It may be found in other regions within the tropical moist forests in the Caribbean lowlands.

###### Additional notes.

Deforestation and fires for extensive cattle ranching and agriculture are among their main threats. Global conservation status is NE and DD in Honduras.

##### 137. Chiroderma salvini

Taxon classificationAnimaliaChiropteraPhyllostomidae

Dobson, 1878

77417671-71C3-538A-81FE-FA7DCA9B1B24

###### Natural history.

Lactating females have been found in November and December. Individuals have been captured in the middle of coffee and corn plantations, as well as in areas where legal pine extraction occurs.

###### Distribution.

**Figure 72.**
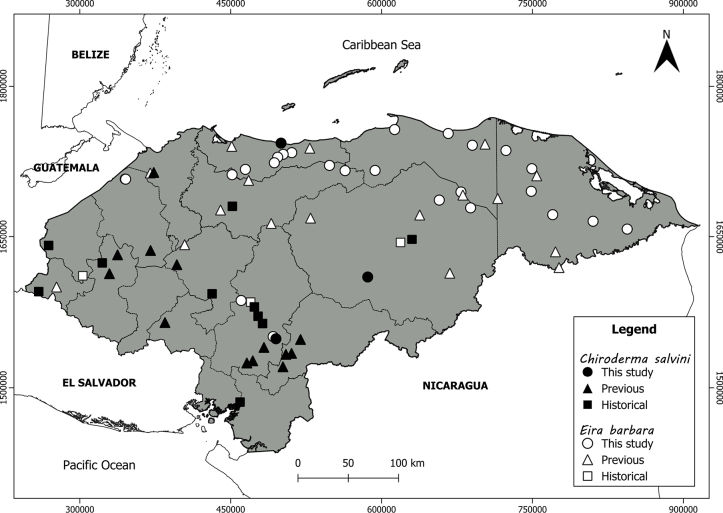
Distribution of *Chiroderma salvini* and *Eira barbara* in Honduras.

**Atlántida**, Choluteca, Comayagua, Copán, Cortés, El Paraíso, Francisco Morazán, Lempira, La Paz, Ocotepeque, Olancho, Santa Bárbara, and Yoro (Fig. 72); 540–1500 m a.s.l.

###### Additional notes.

Global conservation status is LC and NE in Honduras.

##### 138. Chiroderma villosum

Taxon classificationAnimaliaChiropteraPhyllostomidae

Peters, 1860

77CE09C4-74CB-56FC-A748-2A2DFD495D2B

###### Natural history.

Active males have been captured in November, October, and December. Lactating females have been found in November and December. Individuals have been captured with fig fruits in their mouth (*Ficus* spp.). Individuals have been captured in the middle of coffee and corn plantations.

###### Distribution.

Atlántida, Choluteca, Comayagua, Copán, Cortés, Francisco Morazán, Gracias a Dios, **Intibucá**, **Olancho**, Santa Bárbara, Valle, and **Yoro** (Fig. 10); 10–1400 m a.s.l.

###### Additional notes.

Global conservation status is LC and NE in Honduras.

#### *Centurio* Gray, 1842

##### 139. Centurio senex

Taxon classificationAnimaliaChiropteraPhyllostomidae

Gray, 1842

0D2B00EA-A0F0-56D9-B93F-5581069AE252

###### Natural history.

They have been captured in coffee groves, deciduous forests, and mango plantations. Lactating females have been captured in May and December. Active males have been captured in October. An adult male captured in February in an urban area had *S. erianthum* seeds in its feces. They have been seen with fruits of *Ficus* sp. and Sapotaceae. Trujillo et al. (2022) reviewed the regional distribution.

###### Distribution.

**Figure 73.**
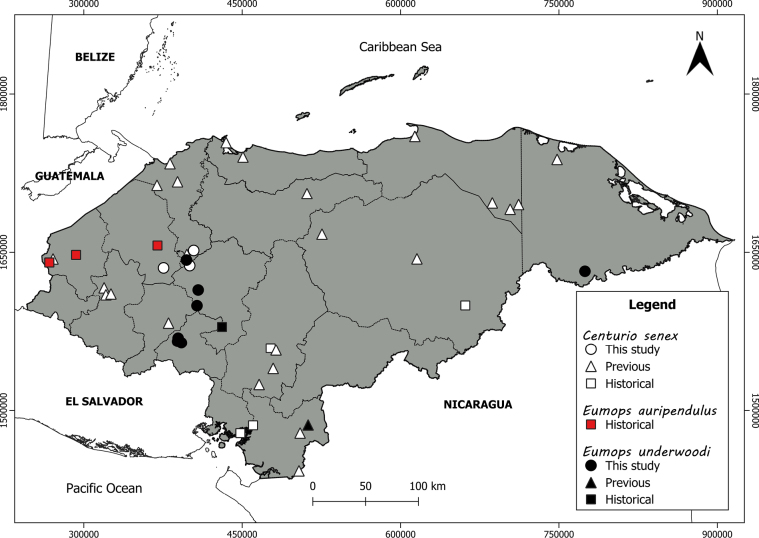
Distribution of *Centurio senex*, *Eumops auripendulus*, and *E. underwoodi* in Honduras.

Atlántida, Choluteca, Colón, **Comayagua**, Copán, Cortés, El Paraíso, Francisco Morazán, Gracias a Dios, Intibucá, Lempira, Olancho, **Santa Bárbara**, Valle, and Yoro (Fig. 73); 245–750 m a.s.l.

###### Additional notes.

Global conservation status is LC and NE in Honduras.

#### *Ectophylla* H. Allen, 1892

##### 140. Ectophylla alba

Taxon classificationAnimaliaChiropteraPhyllostomidae

H. Allen, 1892

A248298D-F966-5F7E-8698-37CF1192EA8D

###### Natural history.

Leaf tent roosts have been found in Colón and Gracias a Dios. A pregnant female was captured in August. It has been captured over rivers, but see Allen (1892).

###### Distribution.

**Figure 74.**
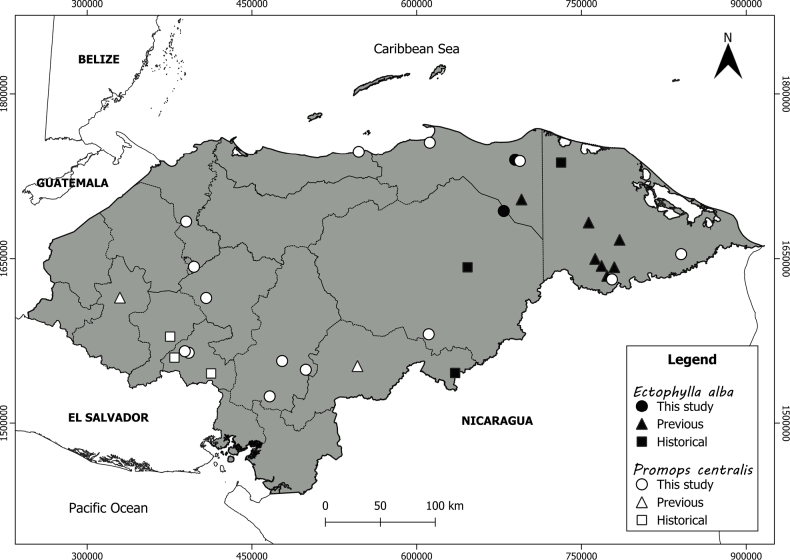
Distribution of *Ectophylla alba* and *Promops centralis* in Honduras.

**Colón**, El Paraíso, Olancho, and Gracias a Dios (Fig. 74); 80–150 m a.s.l.

###### Additional notes.

They are known as skanki (sakanki) pihini by the Miskitus in eastern Honduras. The northernmost record of the species is in Parque Nacional Sierra de Río Tinto, Colón department, a proposed protected area, and further north than the distribution predicted by Portillo-Reyes et al. (2015). We hypothesize that there may be two disjunct populations: one in Colón, northern Olancho, and Gracias a Dios, and another in southern Muskitia (referred to geographically as the La Moskitia region). It appears likely that this species has been extirpated from El Paraíso, as the connection to the forest in the Honduran Moskitia has been lost. The southern population is restricted to Mabita, Rus Rus. However, further sampling is needed to confirm the species’ occurrence in this region; unfortunately, this population is threatened by both legal and illegal logging (Portillo Reyes et al. 2015). Global conservation status is NT and CR in Honduras.

#### *Enchisthenes* K. Andersen, 1906

##### 141. Enchisthenes hartii

Taxon classificationAnimaliaChiropteraPhyllostomidae

(Thomas, 1892)

FB258AFF-BF68-588A-A4EE-680AFCBECFDD

###### Natural history.

Lactating females have been captured in September, and during the same month, Solanaceae seeds have been found in the feces of adult females. An individual captured in San Buenaventura, Francisco Morazán department, was injured by barbed wire fencing in a finca. Individuals have been captured in the middle of sugarcane plantations and suburban areas.

###### Distribution.

**Figure 75.**
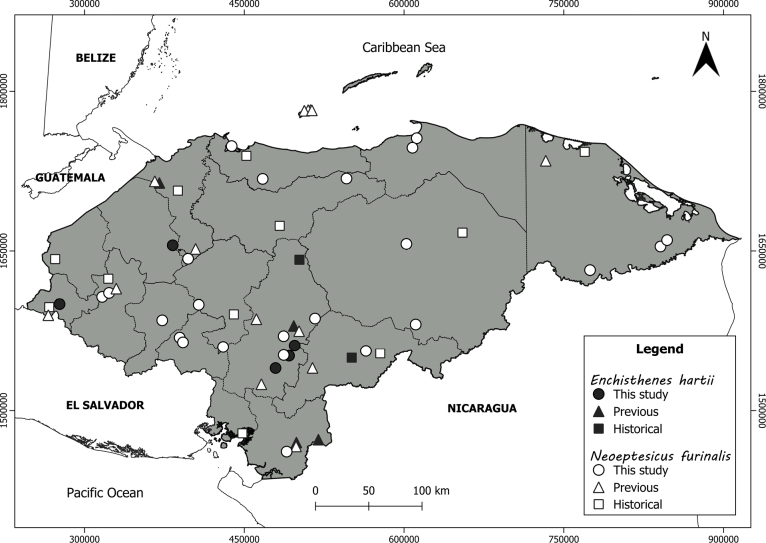
Distribution of *Enchisthenes hartii* and *Neoeptesicus furinalis* in Honduras.

Choluteca, Cortés, El Paraíso, Francisco Morazán, **Ocotepeque**, and **Santa Bárbara** (Fig. 75); 750–2005 m a.s.l.

###### Additional notes.

Global conservation status is LC and DD in Honduras.

#### *Platyrrhinus* Saussure, 1860

##### 142. Platyrrhinus helleri

Taxon classificationAnimaliaChiropteraPhyllostomidae

(Peters, 1866)

DEE01037-E12A-5A0B-8C7B-C9974172EB69

###### Natural history.

Lactating females have been captured in May and October. Individuals have been captured above and in proximity to streams and rivers. It is a host of *Paratrichobius* sp. (complex *salvini*) on *P. helleri* from Atlántida.

###### Distribution.

Atlántida, Choluteca, Colón, Comayagua, Cortés, El Paraíso, **Francisco Morazán**, Gracias a Dios, Lempira, and Olancho (Fig. 54); 50–1600 m a.s.l.

###### Additional notes.

Global conservation status is LC and NE in Honduras.

#### *Sturnira* Gray, 1842

##### 143. Sturnira hondurensis

Taxon classificationAnimaliaChiropteraPhyllostomidae

Goodwin, 1940

F28EBA73-A10E-58A5-9836-DC1C162536F4

###### Natural history.

This was reported as one of the dominant species captured, with an average of 70 individuals per year during long-term surveys in Cusuco National Park (Medina-Van Berkum et al. 2020). Reproductive males have been captured in February and April. Pregnant females have been captured in February and November, and lactating females in February, June, October, and November. Seeds of *C. peltata* have been found in feces. They have been captured in coffee, citrus, and mango plantations. Four species of streblid bat flies are reported on *S. hondurensis*. Dick (2013) reported *Aspidoptera falcata* Wenzel, 1976, and *Paratrichobius longicrus* (Miranda-Ribeiro, 1907) from Lempira and *Megistopoda proxima* (Séguy, 1926) from Francisco Morazán, Lempira, and Olancho. Being *P. longicrus* is considered an accidental or transitory occurrence. Graciolli et al. (2021) reported *Aspidoptera delatorrei* Wenzel, 1966 from Olancho. In addition, we report the occurrence of *M. proxima* on *S. hondurensis* from Parque Nacional La Tigra (Fracisco Morazán).

###### Distribution.

**Figure 76.**
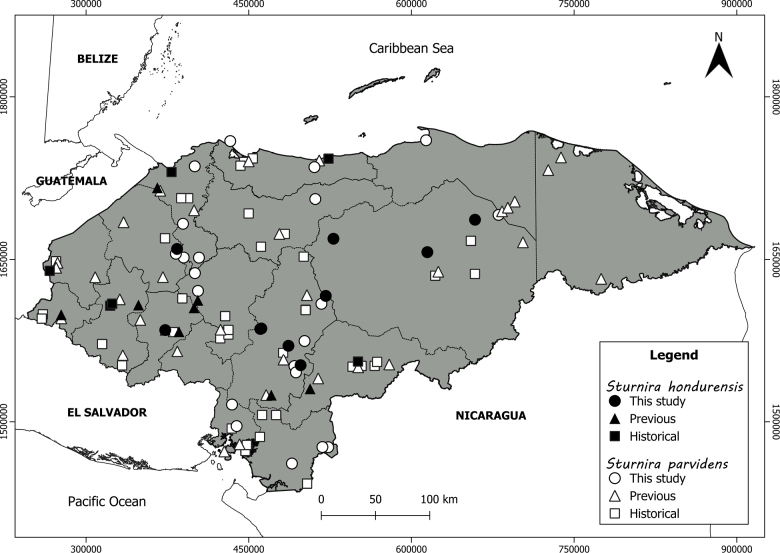
Distribution of *Sturnira hondurensis* and *S. parvidens* in Honduras.

Atlántida, Comayagua, Copán, Cortés, El Paraíso, Francisco Morazán, Intibucá, Lempira, Ocotepeque, Olancho, and Santa Bárbara (Fig. 76); 970–1785 m a.s.l.

###### Additional notes.

Historical specimens were referred to as *Sturnira ludovici* Anthony, 1924, or *Sturnira bogotensis* Shamel, 1927. Global conservation status is LC and NE in Honduras.

##### 144. Sturnira parvidens

Taxon classificationAnimaliaChiropteraPhyllostomidae

Goldman, 1917

9A0CBF9C-7C4E-5339-9729-64A1424EBFFE

###### Natural history.

Bats have been captured in coffee and mango plantations close to water bodies (1.5 km). Pregnant females have been captured in March (one embryo), April, May, October, and December, and lactating females in March, April, May, June, October, November, and December. Active males have been captured in March and May. In March, males captured in urban areas in central Honduras had seeds of *M. calabura* and *S. erianthum* in their feces (Turcios-Casco et al. 2021b). It hosts five species of streblid bat flies; two are accidental or transitory occurrences: *Nycterophilia coxata* Ferris, 1916, and *Trichobius intermedius* Peterson & Hurka, 1974, from Comayagua. And other records include: *Aspidoptera falcata* Wenzel, 1976 from Atlántida, Comayagua, Olancho, & Valle, and *Megistopoda proxima* (Séguy, 1926) (cited as *Megistopoda theodori* Wenzel, 1966) from Atlántida, Comayagua, Olancho, Valle; *Aspidoptera delatorrei* Wenzel, 1966 from Comayagua; and a spinturnicid mite *Periglischrus ojastii* Machado-Allison, 1964 from Comayagua and Francisco Morazán. New records include *M. proxima* from Cerro Azul (Comayagua), Las María Pesh (Gracias a Dios), and Ciudad Universitaria (Francisco Morazán).

###### Distribution.

All the departments except Islas de la Bahía (Fig. 76), 60–1475 m a.s.l.

###### Additional notes.

Previous records have referred to this species as *Sturnira lilium* (É. Geoffroy Saint-Hilaire, 1810), see (Davis 1968). Global conservation status is LC and NE in Honduras.

#### *Uroderma* Peters, 1865

##### 145. Uroderma convexum

Taxon classificationAnimaliaChiropteraPhyllostomidae

Lyon, 1902

967B6281-D67B-5665-81ED-1C25EC6A5441

###### Natural history.

It has been captured above streams. Leaves of Arecaceae and Musaceae are used as tent roosts. Pregnant females were captured during March, and lactating females in September. A female was captured with a fig (Moraceae) in its mouth weighing ~ 9.4% of her body weight. It is host to three species of streblid bat flies: *Megistopoda proxima* (Séguy, 1926) (cited as *M. theodori* Wenzel, 1966) and *Paratrichobius dunni* (Curran, 1935) from Atlántida; *Aspidoptera falcata* Wenzel, 1976 and *Megistopoda proxima* from Francisco Morazán, Lempira, and Olancho; and *Aspidoptera delatorrei* Wenzel, 1966 from Olancho.

###### Distribution.

**Figure 77.**
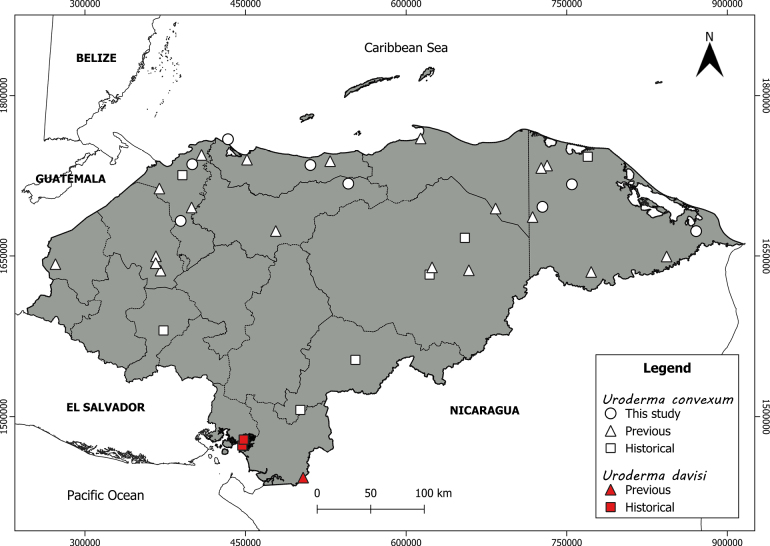
Distribution of *Uroderma convexum* and *U. davisi* in Honduras.

Atlántida, Colón, Copán, Cortés, El Paraíso, Gracias a Dios, Intibucá, Olancho, Santa Bárbara, and Yoro (Fig. 77); 20–530 m a.s.l.

###### Additional notes.

Historical records refer to *Uroderma bilobatum* Peters, 1866. Global and national conservation status is NE.

##### 146. Uroderma davisi

Taxon classificationAnimaliaChiropteraPhyllostomidae

Baker & McDaniel, 1972

002B425A-678D-5A26-B4E2-47E827D888BA

###### Natural history.

No ecological information is known for the species, and it is easily confused with *U. convexum*.

###### Distribution.

Choluteca and Valle (Fig. 77); 50–560 m a.s.l. It is expected to be sympatric with *U. convexum* in Francisco Morazán and El Paraíso.

###### Additional notes.

Historical records have been referred to as *Uroderma bilobatum* Peters, 1866. Global conservation status is NE and DD in Honduras.

##### 147. Uroderma magnirostrum

Taxon classificationAnimaliaChiropteraPhyllostomidae

Davis, 1968

8E718195-E8CA-53A6-A490-6103A410D757

###### Natural history.

This species has not been recorded in the country since 1983.

###### Distribution.

**Figure 78.**
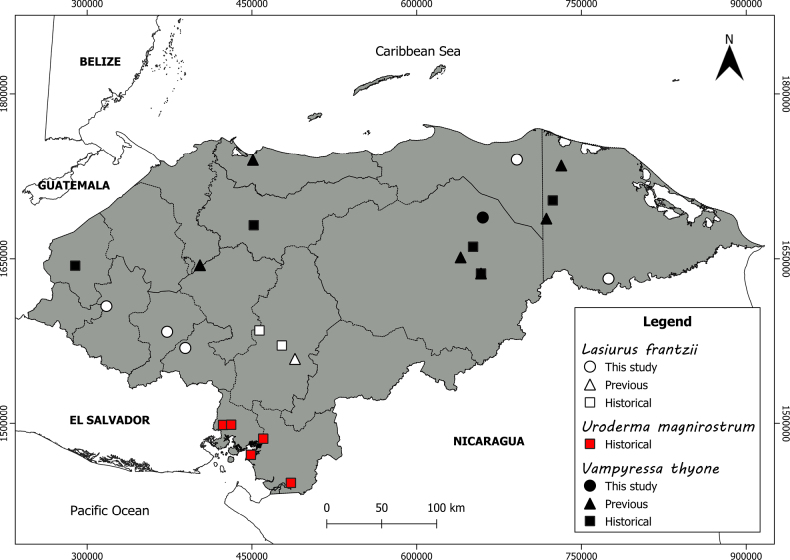
Distribution of *Lasiurus frantzii*, *Uroderma magnirostrum*, and *Vampyressa thyone* in Honduras.

Known only from Choluteca and Valle (Fig. 78); 0–560 m a.s.l.

###### Additional notes.

Global conservation status is LC and DD in Honduras.

#### *Vampyressa* Thomas, 1900

##### 148. Vampyressa thyone

Taxon classificationAnimaliaChiropteraPhyllostomidae

Thomas, 1909

5022FAC7-268D-53CC-BB1A-5766B5E3E875

###### Natural history.

Pregnant females have been captured in April. It is a host of *Neotrichobius bisetosus* Wenzel, 1976, based on a new record for the country; Gracias a Dios (Graciolli et al. 2021). However, the authors suggested that the association with this species may have been accidental or transitory.

###### Distribution.

Atlántida, Cortés, Copán, Gracias a Dios, Olancho, and Yoro (Fig. 78); 60–870 m a.s.l.

###### Additional notes.

It has been referred to under several names, including *Vampyressa pusilla* (Wagner, 1843), *Vampyressa minuta* Miller, 1912, or *Artibeus nanus* (Andersen, 1906), in historical records (Ordoñez-Mazier et al. 2020). Global conservation status is LC and NT in Honduras.

#### *Vampyriscus* Thomas, 1900

##### 149. Vampyriscus nymphaeus

Taxon classificationAnimaliaChiropteraPhyllostomidae

(Thomas, 1909)

ED0BEDA8-89D8-53AC-B4C3-E9C31EF66897

###### Natural history.

The first record of this species in Honduras was in 2014, from a very humid subtropical forest (Mora et al. 2014). Pregnant females have been captured in July and August.

###### Distribution.

Only Olancho and **Gracias a Dios** (Fig. 55); 10–180 m a.s.l. Recent records include one from the Auka community in southern Moskitia and the other from the core zone of the Tawahka Asagni Biosphere Reserve, in a tropical moist forest.

###### Additional notes.

Previous records are referred to *Vampyriscus nymphaea* (Thomas, 1909). Global conservation status is LC and DD in Honduras, both as *V. nymphaea*.

#### *Vampyrodes* Thomas, 1900

##### 150. Vampyrodes major

Taxon classificationAnimaliaChiropteraPhyllostomidae

G. M. Allen, 1908

45C99DD4-9234-509A-98C5-56BC341A8E46

###### Natural history.

Captures have been adjacent to streams in tall secondary growth and rainforest (Valdez and LaVal 1971; Medina-Van Berkum et al. 2020).

###### Distribution.

Atlántida, Comayagua, Cortés, El Paraíso, Gracias a Dios, and Olancho (Fig. 53); 245–700 m a.s.l.

###### Additional notes.

Global conservation status is LC and DD in Honduras.

####### Natalidae Gray, 1866

#### *Natalus* Gray, 1838

##### 151. Natalus mexicanus

Taxon classificationAnimaliaChiropteraNatalidae

Miller, 1902

5507FD62-1B83-55D1-955A-499315E0196D

###### Natural history.

Adults were reported reproductively active from May to July, and maternity roosts 25 m deep into mines are known to be used on Útila Island in the Islas de la Bahía department; females carry their newborn until they reach 5–7 days of age, while the hairless pup is in a communal maternity colony; juveniles become independent within one month of birth. This species also uses caverns, abandoned mines, and caves as primary roosts. Pregnant females with one embryo were captured in March and April. Parasites include *Trichobius galei* Wenzel, 1966 on *N. mexicanus*, reported as *Natalus stramineus* J. E. Gray, 1838, from Francisco Morazán, and *T. galei* from Islas de la Bahía (Miller 2014).

###### Acoustic identification.

The few verified recordings are of constrained bats recorded at close range; calls are very high-frequency, > 90–130 kHz, broadband, short-duration pulses. Pending additional field work and based on limited recordings from known species, *Natalus* appears too quiet to be recorded for free-flying field identification.

###### Distribution.

Comayagua, Copán, Cortés, El Paraíso, Francisco Morazán, Islas de la Bahía, La Paz, Olancho, and Santa Bárbara (Fig. 61); 0–1960 m a.s.l.

###### Additional notes.

As *Natalus lanatus* Tejedor, 2005 is a junior synonym of *N. mexicanus*, we retain the latter. See López-Wilchis et al. (2012), Turcios-Casco et al. (2020a, 2021b), and Simmons and Cirranello (2026) for further discussion. Global conservation status is NE and DD in Honduras referring to *N. lanatus*.

####### Molossidae Gervais, 1856

#### *Molossus* É. Geoffroy Saint-Hilaire, 1805

##### 152. Molossus alvarezi

Taxon classificationAnimaliaChiropteraMolossidae

González-Ruiz, Ramírez-Pulido & Arroyo-Cabrales, 2011

16791061-E455-5A09-A13B-E3070F77F1C9

###### Natural history.

Pregnant females with one embryo were captured in February and March, and active males were captured in March and December. We found roosts inside church attics and house roofs in urban and suburban areas.

###### Acoustic identification.

Call shapes are distinctive for the genus and, when recorded in open, free-flying conditions, are always paired narrow-band FM pulses, with a higher-frequency pulse followed by a lower-frequency pulse. When in proximity (formerly referred to as clutter) to objects in their environment, calls such as hand-released bats or those leaving and entering roosts may be recorded as triplets. Prior to the understanding of the nature of proximity calls in this genus, diagnostic calls of this species were initially reported as triplets (O’Farrell and Miller 1999). This has added confusion to published accounts, for example Jung et al. (2014), which state that some species of *Molossus* have three pulses, presented as high, middle, and low. The diagnostic vocal signature pulses for all verified calls of species in the genus *Molossus* are paired. 90% of the combined paired Fc pulses of the dominant harmonic are 40 kHz.

###### Distribution.

**Figure 79.**
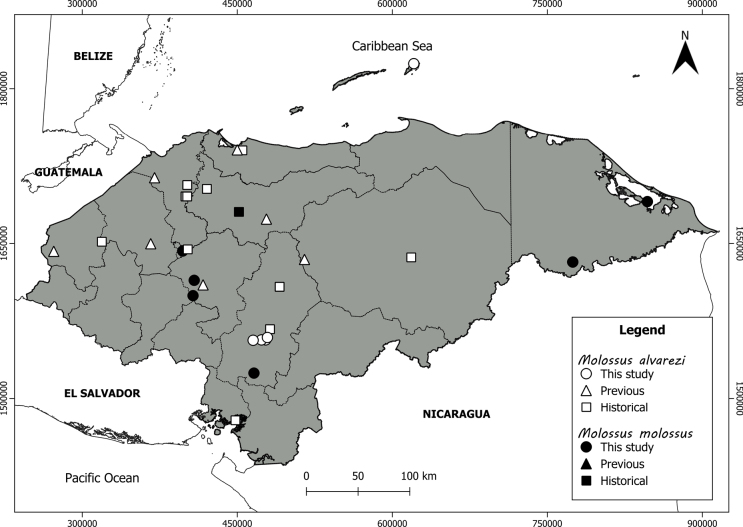
Distribution of *Molossus alvarezi* and *M. molossus* in Honduras.

Atlántida, Comayagua, Copán, Cortés, Francisco Morazán, Islas de la Bahía, Olancho, Santa Bárbara, and Yoro (Fig. 79); 0–1125 m a.s.l.

###### Additional notes.

Previous records have been referred to as *Molossus sinaloae* J. A. Allen, 1906. Global and national conservation is DD.

##### 153. Molossus aztecus

Taxon classificationAnimaliaChiropteraMolossidae

Saussure, 1860

D3BE232E-227B-59DF-A930-508B432D23F5

###### Natural history.

No ecological information is available for this species.

###### Distribution.

**Figure 80.**
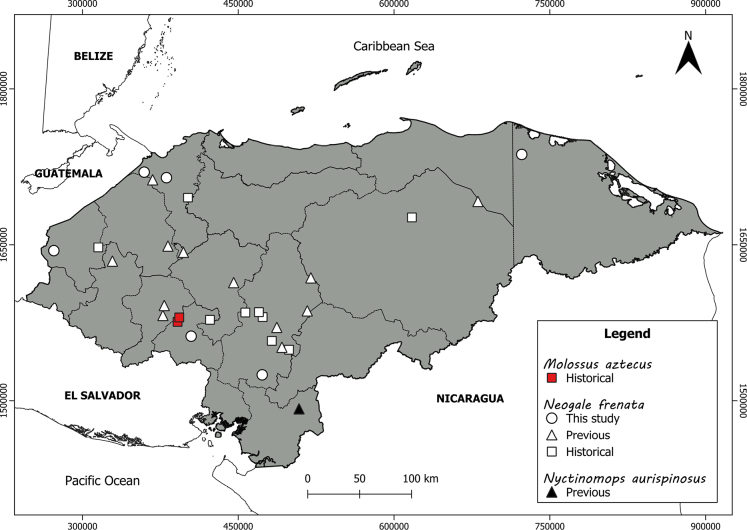
Distribution of *Molossus aztecus*, *Neogale frenata*, and *Nyctinomops aurispinosus* in Honduras.

Only known from the La Paz department (Fig. 80); 685–930 m a.s.l.

###### Acoustic identification.

There are no verified recordings of this species. Call shapes are presumed to be similar to those of other species in the genus, with decreasing FM pulses that are paired with the first at a higher frequency and followed by an identical-looking pulse at a lower frequency.

###### Additional notes.

A group of nine specimens in the MCZ collection, identified as *M. aztecus* needs verification. Global conservation status is LC and DD in Honduras.

##### 154. Molossus bondae

Taxon classificationAnimaliaChiropteraMolossidae

J.A. Allen, 1904

617B8CCA-298F-5358-8B38-008FCEF667C9

###### Natural history.

We have captured these bats over streams. Active males have been captured in September.

###### Acoustic identification.

No verified recordings. Call shapes are presumed to be similar to those of other species in the genus, with decreasing FM pulses paired with the first at a higher frequency, followed by an identical-looking pulse at a lower frequency.

###### Distribution.

**Figure 81.**
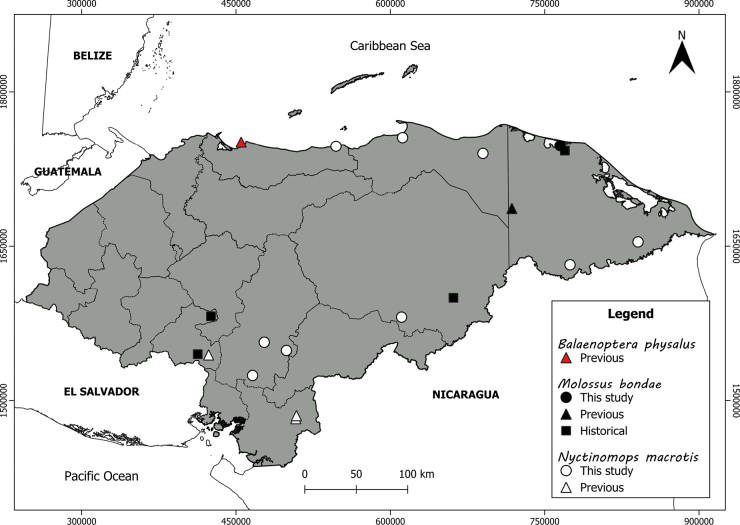
Distribution of *Balaenoptera physalus*, *Molossus bondae*, and *Nyctinomops macrotis* in Honduras.

Gracias a Dios, La Paz, and Olancho (Fig. 81); 0–930 m a.s.l.

###### Additional notes.

Global conservation status is LC and DD in Honduras.

##### 155. Molossus molossus

Taxon classificationAnimaliaChiropteraMolossidae

(Pallas, 1766)

EB834DF2-16B0-5E9D-AFFE-708F98819AD3

###### Natural history.

Roosts have been found in the roofs of houses in urban and suburban areas. We have captured these bats above ponds. The scorpion *Centruroides exilimanus* Teruel & Tockwell, 2002 (Buthidae) has been documented feeding on *M. molossus* (Espinal et al. 2020).

###### Distribution.

Atlántida, Choluteca, Colón, El Paraíso, Francisco Morazán, Gracias a Dios, Islas de la Bahía, Valle, and Yoro (Fig. 79); 6–930 m a.s.l.

###### Acoustic identification.

Call shapes are distinctive within the genus and consist of paired narrowband FM pulses, with a higher-frequency pulse followed by a lower-frequency pulse. Under heavy clutter conditions, calls emitted when leaving or entering a roost may be observed in triplets. 90% of the combined paired Fc pulses of the dominant harmonic are 38 kHz.

###### Additional notes.

Global conservation status is LC and NE in Honduras.

##### 156. Molossus nigricans

Taxon classificationAnimaliaChiropteraMolossidae

Miller, 1902

AA42635B-2DCD-5864-9A9A-BD548C7D679A

###### Natural history.

Pregnant females have been captured with one embryo in March. We have found this species roosting in the attics of houses in Ocotepeque and on roofs in Francisco Morazán. Leucism has been reported on this species (Gómez-Corea et al. 2021b).

###### Acoustic identification.

Call shapes are distinctive within the genus and consist of paired narrowband FM pulses, with a higher-frequency pulse followed by a lower-frequency pulse. Under heavy clutter conditions, leaving and entering roost pulses may be observed in triplets. 90% of the combined paired Fc pulses of the dominant harmonic are 32 kHz.

###### Distribution.

**Figure 82.**
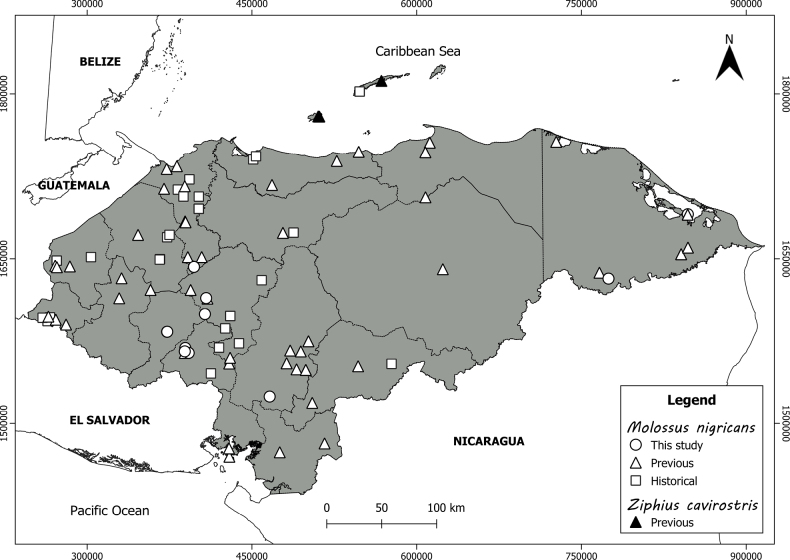
Distribution of *Molossus nigricans* and *Ziphius cavirostris* in Honduras.

The species has been recorded in all 18 departments of Honduras, and we provide the first record for Intibucá (Fig. 82); 0–2010 m a.s.l.

###### Additional notes.

Historical records may refer to *M. rufus* or *M. ater*. Global and national conservation status is NE.

##### 157. Molossus pretiosus

Taxon classificationAnimaliaChiropteraMolossidae

Miller, 1902

6216FAFF-CBD3-5849-916C-0D68C621E723

###### Natural history.

This species is represented by only two specimens from the Caribbean lowlands of Honduras (Turcios-Casco et al. 2024); this species might be confused with other molossids in the Caribbean of Honduras.

###### Acoustic identification.

No acoustic calls have been verified for this species.

###### Distribution.

Islas de la Bahía (Fig. 34); 4 m a.s.l.

###### Additional notes.

This species was expected to occur in the country and was confirmed by a single individual from Roatán Island, but it may be confused with other molossids in the Caribbean (Turcios-Casco et al. 2024). Global conservation status is LC and NE in Honduras.

#### *Promops* Gervais, 1856

##### 158. Promops centralis

Taxon classificationAnimaliaChiropteraMolossidae

Thomas, 1915

7AFC3964-EF23-5975-84B6-F2F81F22A057

###### Natural history.

Probably common throughout the country, but as a high-flying Molossid that avoids capture, it is known only from acoustic and historical records. A dead individual was found in a football court in a urban area (see record www.inaturalist.org/observations/5176151).

###### Distribution.

**Atlántida**, Choluteca, **Colón**, **Comayagua**, **Cortés**, El Paraíso, **Francisco Morazán**, **Gracias a Dios**, Intibucá, La Paz, and Olancho (Fig. 74); 30–1910 m a.s.l.

###### Acoustic identification.

Call shapes are distinctive and are either single FM pulses of increasing frequency or paired pulses with a decreasing FM pulse followed by an increasing-frequency pulse at a much lower frequency. Commuting calls are nearly CF and have a very long duration, often with an increasing FM pulse. 90% of the Fc pulses of the dominant harmonic are 28 kHz.

###### Additional notes.

Global conservation status is LC and NE in Honduras.

#### *Nyctinomops* Miller, 1902

##### 159. Nyctinomops aurispinosus

Taxon classificationAnimaliaChiropteraMolossidae

(Peale, 1849)

715D49D5-1811-5943-8A27-68EE61585F45

###### Natural history.

No information is known from Honduras.

###### Acoustic identification.

No verified calls. Call shapes are presumed to be similar to those of other species in the genus, with single, narrow-band, decreasing-frequency FM pulses at relatively low frequencies.

###### Distribution.

Only the Choluteca department (Mora et al. 2016; Fig. 80); 1240 m a.s.l.

###### Additional notes.

Global conservation status is LC and DD in Honduras.

##### 160. Nyctinomops laticaudatus

Taxon classificationAnimaliaChiropteraMolossidae

(É. Geoffroy Saint-Hilaire, 1805)

08EE88CE-1BDB-5559-8BF4-167ED79E3E84

###### Natural history.

No ecological information is known from Honduras.

###### Acoustic identification.

Call shapes are single, low-frequency, decreasing narrowband FM pulses. 90% of the Fc pulses of the dominant harmonic are 23 kHz.

###### Distribution.

Choluteca, **Gracias a Dios**, **Olancho**, and Yoro departments (Fig. 67); 10–600 m a.s.l.

###### Additional notes.

Global conservation status is LC and DD in Honduras.

##### 161. Nyctinomops macrotis

Taxon classificationAnimaliaChiropteraMolossidae

(Gray, 1839)

4FDBB5F5-33C1-5E6C-B570-A1798E472DF6

###### Natural history.

Only two males are known from Honduras.

###### Distribution.

Choluteca (Fig. 81), 0–1325 m a.s.l.

###### Acoustic identification.

Call shapes are single, decreasing, narrow-bandwidth FM pulses at relatively low frequencies. Given their low frequency, calls of this species may be mistaken for those of *Eumops*. 90% of the Fc pulses of the dominant harmonic are 18 kHz.

###### Additional notes.

Global conservation status is LC and DD in Honduras.

#### *Cynomops* Thomas, 1920

We concur with Simmons and Cirranello (2026) that *Cynomops mexicanus* (Jones & Genoways, 1967) and *C. greenhalli* Goodwin, 1958 are distinct species, and there is insufficient evidence to conclude they are conspecific and include them separately here.

##### 162. Cynomops greenhalli

Taxon classificationAnimaliaChiropteraMolossidae

Goodwin, 1958

08BF5A0E-F626-50E1-8087-45C8CBDC4684

###### Natural history.

Pregnant females have been captured in August.

###### Distribution.

Known only from Atlántida (Fig. 40); 0–1910 m a.s.l.

###### Acoustic identification.

There are no verified calls for this species. Call shapes remain unknown.

###### Additional notes.

The calls attributed to this species by Espinal and Mora (2012) were misidentified and correspond to those of *P. centralis*. Global conservation status is LC and DD in Honduras.

##### 163. Cynomops mexicanus

Taxon classificationAnimaliaChiropteraMolossidae

(Jones & Genoways, 1967)

019D40B5-0559-5224-9B9C-DCDE58EC23A9

###### Natural history.

No ecological information is known from Honduras.

###### Distribution.

Known only from Comayagua (Fig. 61); 10–580 m a.s.l.

###### Acoustic identification.

There are no verified calls for this species. Call shapes remain unknown.

###### Additional notes.

Specimens TCWC 49757–59, which are also from the Atlántida department, should be reviewed to determine which of the two *Cynomops* species they correspond to. Global conservation status is LC and NE in Honduras.

#### *Eumops* Miller, 1906

##### 164. Eumops hansae

Taxon classificationAnimaliaChiropteraMolossidae

Sanborn, 1932

85804258-4ECB-5864-BA37-9A920A8F0F93

###### Natural history.

No information is available on the Honduran population.

###### Distribution.

Only known from the Atlántida department (Fig. 50); 15 m a.s.l.

###### Acoustic identification.

No verified calls. They are presumed to be single, with narrowband FM pulses, as in other known *Eumops* species.

###### Additional notes.

Global conservation status is LC and DD in Honduras.

##### 165. Eumops nanus

Taxon classificationAnimaliaChiropteraMolossidae

(Miller, 1900)

F590598E-EB0A-5162-8E19-A08B4E99EAE2

###### Natural history.

No ecological information is known.

###### Distribution.

Known only in the Gracias a Dios department (Fig. 60); 5 m a.s.l.

###### Acoustic identification.

No verified calls. They are presumed to be single, decreasing-frequency FM pulses, like those of other known *Eumops* species.

###### Additional notes.

Historical specimens are referred to as *Eumops bonariensis* (Peters, 1874). Global conservation status is LC and DD in Honduras.

##### 166. Eumops auripendulus

Taxon classificationAnimaliaChiropteraMolossidae

(Shaw, 1800)

7AFA019F-0DE1-595B-9555-9C1D85C0A3A6

###### Natural history.

No ecological information is known.

###### Distribution.

Only known from Copán and Santa Bárbara (Fig. 73); 620–700 m a.s.l.

###### Acoustic identification.

Call shapes are single, decreasing-frequency, narrow-band FM pulses at very low frequencies. 90% of the Fc pulses of the dominant harmonic are 17 kHz.

###### Additional notes.

Global conservation status is LC and DD in Honduras.

##### 167. Eumops underwoodi

Taxon classificationAnimaliaChiropteraMolossidae

Goodwin, 1940

FAC53BD6-DA3C-5CDF-9C14-36E6E1C93B93

###### Natural history.

No information is available on the Honduran population.

###### Distribution.

Only known from Choluteca, **Comayagua**, **Cortés**, **Gracias a Dios**, and La Paz (Fig. 73); 920–1110 m a.s.l.

###### Acoustic identification.

Call shapes are single, decreasing-frequency, narrow-band FM pulses at low frequencies. 90% of the Fc pulses of the dominant harmonic are 21 kHz.

###### Additional notes.

Global conservation status is LC and NE in Honduras.

##### 168. Eumops ferox

Taxon classificationAnimaliaChiropteraMolossidae

(Gundlach in Peters, 1861)

0D100255-D967-5B53-817F-D7891C98C006

###### Natural history.

LaVal (1969) reported lactating females of *Eumops glaucinus* (Wagner, 1843) in August, based on individuals captured over a shallow river.

###### Distribution.

Comayagua, **Copán**, Francisco Morazán, and La Paz (Fig. 31); 560–930 m a.s.l.

###### Acoustic identification.

No verified calls. They are presumed to be single, with narrow-band FM pulses, as in other species in the genus.

###### Additional notes.

Referred to as *E. glaucinus* by Goodwin (1942a). Global conservation status is LC and DD in Honduras.

#### *Tadarida* Rafinesque, 1814

##### 169. Tadarida brasiliensis

Taxon classificationAnimaliaChiropteraMolossidae

(I. Geoffroy Saint-Hilaire, 1824)

7666E97E-D00A-5A6C-BAEA-388B7BEF9B0B

###### Natural history.

Probably occurs throughout the country but is known only from historically preserved specimens, and continued acoustic surveys will likely expand the known range.

###### Distribution.

Choluteca, **Comayagua**, **Cortés**, Francisco Morazán, **Intibucá**, **La Paz**, Ocotepeque, and Yoro (Fig. 84). But presumably well distributed in the country; 600–960 m a.s.l.

**Figure 83.**
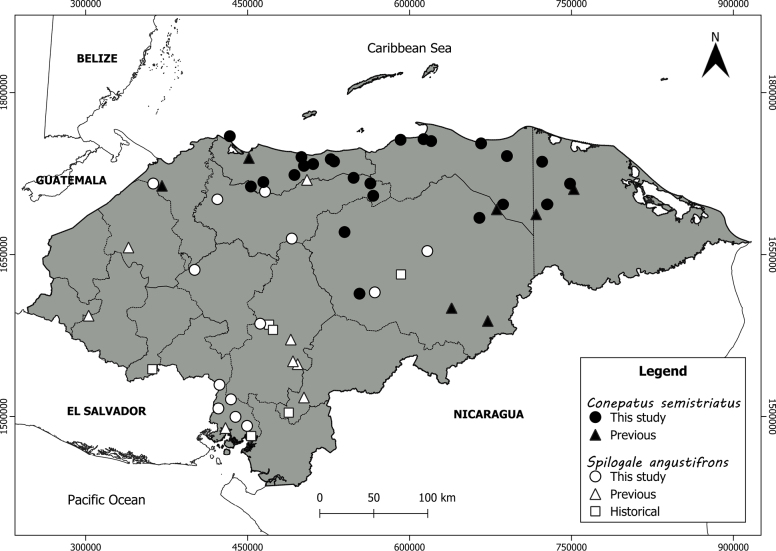
Distribution of *Conepatus semistriatus* and *Spilogale angustifrons* in Honduras.

**Figure 84.**
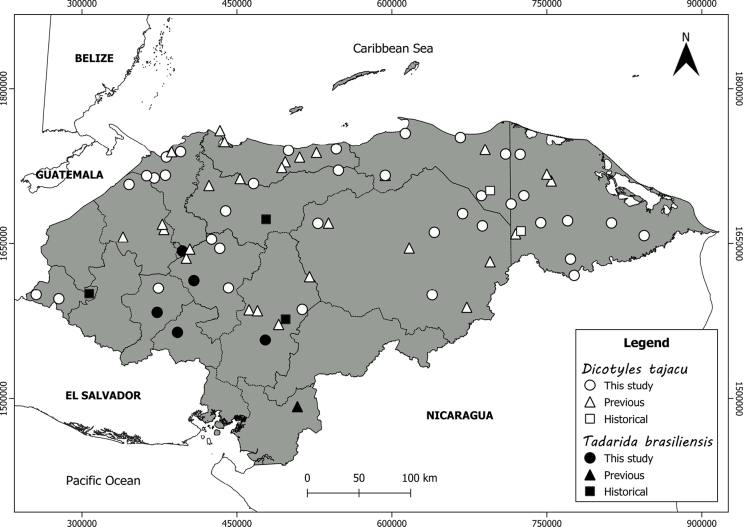
Distribution of *Tadarida brasiliensis* and *Dicotyles tajacu* in Honduras.

###### Acoustic identification.

Generally, single FM pulses of decreasing frequency. However, calls are highly variable, ranging from narrow to broadband pulses, as bats transition from the search to the approach phase and when flying in proximity to objects in their environment. 90% of the Fc pulses of the dominant harmonic are 28 kHz.

###### Additional notes.

Global conservation status is LC and DD in Honduras.

####### Vespertilionidae Gray 1821

#### *Aeorestes* Fitzinger, 1870

##### 170. Aeorestes cinereus

Taxon classificationAnimaliaChiropteraVespertilionidae

(Palisot de Beauvois, 1796)

E235EC5C-8A9B-53D8-A5ED-B243E8F6CBB0

###### Natural history.

It has been captured in urban areas.

###### Distribution.

Choluteca and Francisco Morazán (Mora and López 2010) (Fig. 57); 960–1660 m a.s.l.

###### Acoustic identification.

Calls are typically Lasiurine-shaped, with a pronounced hook at the lower parts of the call pulses, and a relatively short, broadband FM-decreasing component. All known Lasiurines exhibit a distinct oscillatory pattern, with the Fc of the dominant harmonic varying irregularly in frequency. 90% of the Fc pulses of the dominant harmonic are 23 kHz. The acoustic record of *A. cinereus* reported from Choluteca, identified as *Lasiurus cinereus* (Palisot de Beauvois, 1796) (Espinal and Mora 2016), was found to be invalid. This identification was likely due to misinterpretation by an automated identification algorithm, and the sequence was verified as pulses that were part of a feeding buzz sequence of a Molossid.

###### Additional notes.

We find the elevation of the three subgenera of the Lasiurines to full genera by Baird et al. (2015, 2017, 2021) compelling and recognize *A. cinereus*. Global conservation status is LC and DD in Honduras, both referring to *L. cinereus*.

##### 171. Aeorestes egregius

Taxon classificationAnimaliaChiropteraVespertilionidae

(Peters, 1870)

AF303C80-4CD6-5A94-8BC9-173660473415

###### Natural history.

No ecological information is known for this species.

###### Distribution.

Only known from Olancho (Fig. 57); 300 m a.s.l.

###### Acoustic identification.

No verified calls are available for this species. Call shapes are presumed to be typical Lasiurine pulses.

###### Additional notes.

We find the elevation of the three subgenera of the Lasiurines to full genera by Baird et al. (2015, 2017, 2021) compelling and recognize this species as *A. egregius*. It was first reported by Mora (2012). Global and national conservation status DD, both referring to *L. egregius* (Peters, 1870).

#### *Bauerus* Van Gelder, 1959

##### 172. Bauerus dubiaquercus

Taxon classificationAnimaliaChiropteraVespertilionidae

(Van Gelder, 1959)

97069B66-7123-5F26-BE40-4191F19F1459

###### Natural history.

This species was reported to be among the most common in the Cusuco National Park, with 118 individuals captured *across all* elevation ranges surveyed (Medina-Van Berkum et al. 2020). It is the host of *Basilia dubiaquercus* Graciolli & Dick, 2009, based on a specimen from Olancho (Dick 2013).

###### Distribution.

**Atlántida**, **Colón**, Cortés, **Francisco Morazán**, **Gracias a Dios**, **La Paz**, and Olancho (Fig. 63); 10–1950 m a.s.l.

###### Acoustic identification.

Call shapes are typical vespertilionid pulses. Relatively short-duration broadband FM decreasing frequency component, usually with a small hook or upturned end of the pulse. 90% of the Fc pulses of the dominant harmonic are 45 kHz.

###### Additional notes.

Global conservation status is LC and DD in Honduras.

#### *Eptesicus* Rafinesque, 1820

##### 173. Eptesicus fuscus

Taxon classificationAnimaliaChiropteraVespertilionidae

(Palisot de Beauvois, 1796)

39358229-D15E-50CD-A478-EF8064B3B19A

###### Natural history.

This species has been captured over streams. Sexually active males have been recorded in December.

###### Distribution.

**Comayagua**, Francisco Morazán, Intibucá, La Paz, and **Lempira** (Fig. 63); 560–1910 m a.s.l.

###### Acoustic identification.

Call shapes are typical vespertilionid pulses. Relatively short-duration broadband FM decreasing frequency component, usually with a small hook or upturned end of the pulse, appearing like a reversed J. 90% of the Fc pulses of the dominant harmonic are 30 kHz.

###### Additional notes.

We retain the name *E. fuscus* following Simmons and Cirranello (2026), but find insufficient evidence to support elevating the subspecies *Eptesicus fuscus miradorensis* Bangs, 1902 to a full species (Ramírez-Chaves et al. 2023). Global conservation status is LC and NE in Honduras.

#### *Neoeptesicus* Cláudio, Novaes, Gardner, Nogueira, Wilson, Maldonado, Oliveira & Moratelli, 2023

##### 174. Neoeptesicus brasiliensis

Taxon classificationAnimaliaChiropteraVespertilionidae

(Desmarest, 1819)

B5903E8A-1981-5635-B8C0-30E1A73199E4

###### Natural history.

No information on the Honduran populations is available.

###### Acoustic identification.

Call shapes are typical vespertilionid pulses. Relatively short-duration broadband FM decreasing frequency component, usually with a small hook or upturned end of the pulse appearing, like a reversed J. 90% of the Fc pulses of the dominant harmonic are 35 kHz.

###### Distribution.

Choluteca, **Comayagua**, Cortés, El Paraíso, **Francisco Morazán**, **Gracias a Dios**, **La Paz**, and **Lempira** (Fig. 25); 1080–1950 m a.s.l.

###### Additional notes.

The establishment of the genus *Neoeptesicus* (Cláudio et al. 2023) led to the reassignment of all previously identified records of *E. brasiliensis* to that genus. Historical specimens are referred to as *Eptesicus brasiliensis* (Desmarest, 1819). Global conservation status is LC and DD in Honduras, both referring to *E. brasiliensis*.

##### 175. Neoeptesicus furinalis

Taxon classificationAnimaliaChiropteraVespertilionidae

(d'Orbigny & Gervais, 1847)

15BE4A30-D600-5376-BB29-58BA1335CE2E

###### Natural history.

It has been captured above streams. Scrotal males have been recorded in December. One roost in a hollow, dead black mangrove trunk (2.5 cm high) held up to five individuals. Graciolli et al. (2021) reported the nycteribiid bat fly *Basilia ortizi* Machado-Allison, 1963, on *N. furinalis* from Gracias a Dios. Regionally, this species is known to roost in buildings. Martínez Murillo et al. (2021) found five individuals in a 2.5-cm-high hollow dead black mangrove trunk on the island of Útila.

###### Distribution.

Atlántida, Choluteca, **Colón**, Comayagua, Copán, Cortés, El Paraíso, Francisco Morazán, Gracias a Dios, **Intibucá**, Islas de la Bahía, **La Paz**, Lempira, Ocotepeque, Olancho, Valle, and Yoro (Fig. 75); 240–1700 m a.s.l.

###### Acoustic identification.

Call shapes are typical vespertilionid pulses. Relatively short-duration broadband FM decreasing frequency component, usually with a small hook or upturned end of the pulse appearing, like a reversed J. 90% of the Fc pulses of the dominant harmonic are 36 kHz.

###### Additional notes.

The first record was apparently a male, collected by Underwood on July 12, 1933, from Comayagua, reported as *Eptesicus albigularis* (Goodwin, 1946) by Goodwin (1942b). This is now considered a synonym of *E. furinalis* (Mies et al. 1996).

Additionally, specimens identified as *Eptesicus montosus* (Thomas, 1920), were found to have been misidentified as *Eptesicus andinus* J. A. Allen, 1914 and should be thoroughly reviewed (see Mies et al. 1996). With the establishment of the genus *Neoeptesicus* (Cláudio et al. 2023) we now consider previous records of *E. furinalis* to be recognized as this genus. Global conservation status as *E. furinalis* is LC and NE in Honduras.

#### *Perimyotis* Menu, 1984

##### 176. Perimyotis subflavus

Taxon classificationAnimaliaChiropteraVespertilionidae

(F. Cuvier, 1832)

82000BEF-2B2A-531C-914D-C57404CF4FDB

###### Natural history.

It has been found roosting alongside *Anoura peruana* in caves in very humid lower premontane forests of western Honduras (Turcios-Casco et al. 2020d).

###### Distribution.

Atlántida, Choluteca, Copán, Cortés, and Olancho (Fig. 30); 0–1650 m a.s.l.

###### Acoustic identification.

Call shapes are typical vespertilionid pulses. Relatively short-duration broadband FM decreasing frequency component, usually with a small hook or upturned end of the pulse appearing, like a reversed J. 90% of the Fc pulses of the dominant harmonic are 44 kHz.

###### Additional notes.

Historical records referred to this species as *Pipistrellus subflavus* (Cuvier, 1832). This species is considered migratory; it may migrate to other regions of Central America (Leivers et al. 2019), including other caves of Honduras. Global conservation status is LC and VU in Honduras.

#### *Rhogeessa* H. Allen, 1866

Records of *Rhogeessa tumida* H. Allen, 1866, mentioned by Goodwin (1942a) were not included and should be reviewed.

##### 177. Rhogeessa bickhami

Taxon classificationAnimaliaChiropteraVespertilionidae

Baird, Marchan-Rivadeneira, Perez & Baker, 2012

B7E9E544-28AB-5BAA-8BCF-83B33F42E4CA

###### Natural history.

Drowned specimens have been found in swimming pools in urban areas in Francisco Morazán. Lactating females have been captured in May.

###### Distribution.

**Figure 85.**
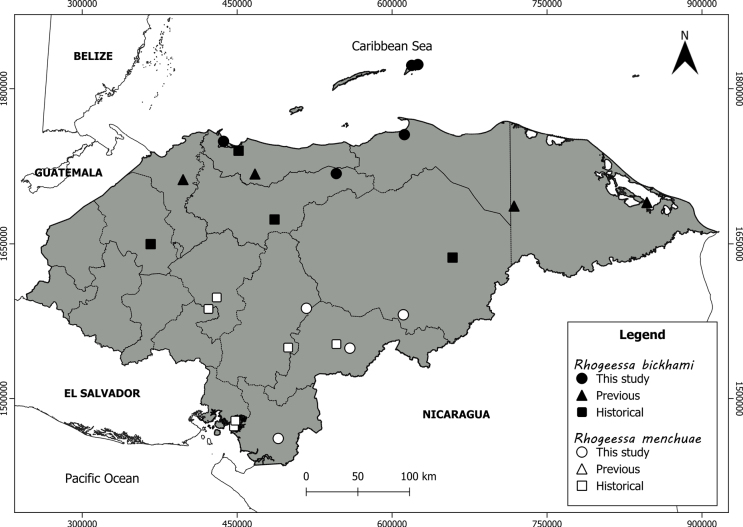
Distribution of *Rhogeessa bickhami* and *R. menchuae* in Honduras.

Atlántida, Choluteca, Comayagua, El Paraíso, Francisco Morazán, Gracias a Dios, and La Paz (Fig. 85); 240–1290 m a.s.l.

###### Acoustic identification.

Call shapes are assumed to be similar to those of verified *Rhogeessa* species. While there are no verified calls for this species yet, we have presumptive recordings matching the genus within its distribution range in Honduras and Nicaragua. These are typical vespertilionid shapes with a relatively short, broadband FM-decreasing component, typically with a small hook or upturned end of the pulse.

###### Additional notes.

No external morphological differences have been found to distinguish *R. bickhami* from *Rhogeessa menchuae* Baird, Marchan-Rivadeneira, Perez & Baker, 2012 (Baird et al. 2012); the key difference may lie in geographic distribution. Neither species has been verified acoustically. Global conservation status is LC and DD in Honduras.

##### 178. Rhogeessa menchuae

Taxon classificationAnimaliaChiropteraVespertilionidae

Baird, Marchan-Rivadeneira, Perez & Baker, 2012

C82F11EB-9256-58D2-9DB4-3D00C25974BA

###### Natural history.

Pregnant females have been captured in March (two embryos).

###### Distribution.

Atlántida, Colón, Cortés, Gracias a Dios, Islas de la Bahía, Lempira, Olancho, Santa Bárbara, and Yoro (Fig. 85); 0–1015 m a.s.l.

###### Acoustic identification.

Call shapes are assumed to be similar to those of known *Rhogeessa* species. While there are no verified calls for this species yet, we have presumptive recordings matching the genus within its distribution range in Honduras and Nicaragua. These are typical vespertilionid shapes, with a relatively short broadband FM-decreasing frequency component, typically with a small hook or upturned end of the pulse.

###### Additional notes.

No external morphological differences have been found to distinguish *R. bickhami* from *R. menchuae*; the key difference may lie in geographic distribution. Neither species has been verified acoustically. It is anticipated that vocal signatures will differ and may differentiate the species in the field. Global and national conservation status is DD.

#### *Dasypterus* Peters, 1870

##### 179. Dasypterus ega

Taxon classificationAnimaliaChiropteraVespertilionidae

(Gervais, 1856)

1E615234-62DE-5A23-BA0C-DA6D93645508

###### Natural history.

No information is available on the Honduran population of this species.

###### Distribution.

**Atlántida**, Choluteca, **Colón**, Comayagua, Cortés, Francisco Morazán, **Gracias a Dios**, Intibucá, **La Paz**, Lempira, Ocotepeque, Valle, and Yoro (Fig. 17); 0–1600 m a.s.l.

###### Acoustic identification.

Calls are typically Lasiurine-shaped, with a pronounced hook at the lower parts of the call pulses, and a relatively short, broadband FM-decreasing component. 90% of the Fc pulses of the dominant harmonic are 33 kHz.

###### Additional notes.

It is one of the species that is affected by air turbine accidents (Mora et al. 2018). We find the elevation of the three subgenera of the Lasiurines to full genera, as proposed by Baird et al. (2015, 2017, 2021), compelling, and recognize this species as *D. ega*. Global conservation status is LC and NT in Honduras, both referring to *Lasiurus ega* (Gervais, 1855).

##### 180. Dasypterus intermedius

Taxon classificationAnimaliaChiropteraVespertilionidae

H. Allen, 1862

7D51E3AA-E78B-5612-9E88-F9332442DB6C

###### Natural history.

No information is available on the Honduran population of this species.

###### Distribution.

**Atlántida**, **Cortés**, Francisco Morazán, Gracias a Dios, Lempira, Ocotepeque, and Valle (Fig. 31); 0–1500 m a.s.l.

###### Acoustic identification.

Calls are typically Lasiurine-shaped, with a pronounced hook at the lower parts of the call pulses and a relatively short, broadband FM-decreasing component. 90% of the Fc pulses of the dominant harmonic are 29 kHz.

###### Additional notes.

We find the elevation of the three subgenera of the Lasiurines to full genera by Baird et al. (2015, 2017, 2021) compelling and recognize this species as *D. intermedius*. Global conservation status is LC and DD in Honduras, both referring to *L. intermedius* H. Allen, 1862.

#### *Lasiurus* Gray, 1831

##### 181. Lasiurus frantzii

Taxon classificationAnimaliaPoalesVespertilionidae

(Peters, 1871)

3DB3250B-F836-5B79-94B2-AD70BDAC88CB

###### Natural history.

It is common to find this species killed by wind turbines and in houses in central Honduras during the migratory season. They have been captured above small ponds in urban areas.

###### Distribution.

**Colón**, Comayagua, Francisco Morazán, **Gracias a Dios**, **Intibucá**, **La Paz**, and **Lempira** (Fig. 78); 920–1600 m a.s.l.

###### Acoustic identification.

No verified recordings. Calls are assumed to have a typical Lasiurine shape, with a pronounced hook at the lower parts of the call pulses and a relatively short, broadband FM-decreasing component.

###### Additional notes.

Some previous records referred to *Lasiurus borealis mexicana* Lima, 1926 or *Lasiurus blossevillii* (Lesson & Garnot, 1826). Global conservation status is NE and NT in Honduras.

#### *Myotis* Kaup, 1829

##### 182. Myotis albescens

Taxon classificationAnimaliaChiropteraVespertilionidae

(É. Geoffroy Saint-Hilaire, 1806)

E9D97B52-619B-552D-906E-BC8E5B6FDBCD

###### Natural history.

Lactating females have been captured in May.

###### Distribution.

**Figure 86.**
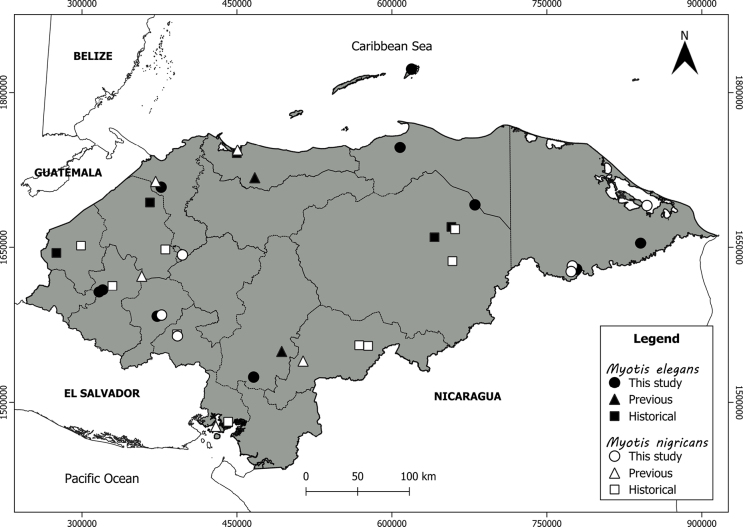
Distribution of *M. elegans* and *M. nigricans* in Honduras.

Atlántida, Choluteca, **Comayagua**, **Cortés**, **Gracias a Dios**, **La Paz**, and Santa Bárbara (Fig. 86); 40–1600 m a.s.l.

###### Acoustic identification.

Call shapes are typically reversed J vespertilionid pulses. Relatively short broadband FM decreasing frequency components, which usually will have a small hook or upturned end of the pulse (called a toe in most Neotropical species of *Myotis*). 90% of the Fc pulses of the dominant harmonic are 48 kHz.

###### Additional notes.

Global conservation status is LC and NE in Honduras.

##### 183. Myotis elegans

Taxon classificationAnimaliaChiropteraVespertilionidae

Hall, 1962

4AA0D23B-19B0-545F-9D43-C34936AD0CE5

###### Natural history.

It has been recorded foraging over streams.

###### Distribution.

Atlántida, Colón, Copán, **Francisco Morazán**, Gracias a Dios, **Intibucá**, Isla de la Bahía, **Lempira**, Olancho, and Santa Bárbara (Fig. 86); 0–1005 m a.s.l.

###### Acoustic identification.

Call shapes are typically reversed J vespertilionid pulses. Relatively short broadband FM decreasing frequency components, which usually will have a small hook or upturned end of the pulse (called a toe in most Neotropical species of *Myotis*). 90% of the Fc pulses of the dominant harmonic are 68 kHz.

###### Additional notes.

Global conservation status is LC and DD in Honduras.

##### 184. Myotis nigricans

Taxon classificationAnimaliaChiropteraVespertilionidae

(Schinz, 1821)

D929CB17-E390-5982-AA40-D79F7CF09C3F

###### Natural history.

Known to roost in caves. They have been captured on riversides. It is the host of *Basilia rondanii* Guimarães & D'Andretta, 1956, on an individual captured in Gracias a Dios (Guimarães and D'Andretta 1956).

###### Distribution.

Atlántida, Copán, Cortés, El Paraíso, **Gracias a Dios**, **Intibucá**, La Paz, Lempira, Olancho, Santa Bárbara, and Valle (Fig. 86); 1900 en Cusuco m a.s.l.

###### Acoustic identification.

Call shapes are typically reversed J vespertilionid pulses. Relatively short broadband FM decreasing frequency components, which usually will have a small hook or upturned end of the pulse (called a toe in most Neotropical species of *Myotis*). Given potential misidentifications of this species with bats in hand, the acoustic identification of the vocal signature remains uncertain, possibly due to cryptic species remaining throughout the putative range under this name. 90% of the Fc pulses of the dominant harmonic are 54 kHz.

###### Additional notes.

Global conservation status is LC and NE in Honduras.

##### 185. Myotis pilosatibialis

Taxon classificationAnimaliaChiropteraVespertilionidae

LaVal, 1973

034C5BDA-03CE-563C-8F4F-B5FC34A967FA

###### Natural history.

A cluster of hundreds of these bats was found on the roof of a cave in central Honduras. It is the host of *Anatrichobius scorzai* Wenzel, 1966 from Francisco Morazán and Olancho and *Trichobius hirsutulus* Bequaert, 1933 from Cortés (Dick 2013).

###### Distribution.

**Figure 87.**
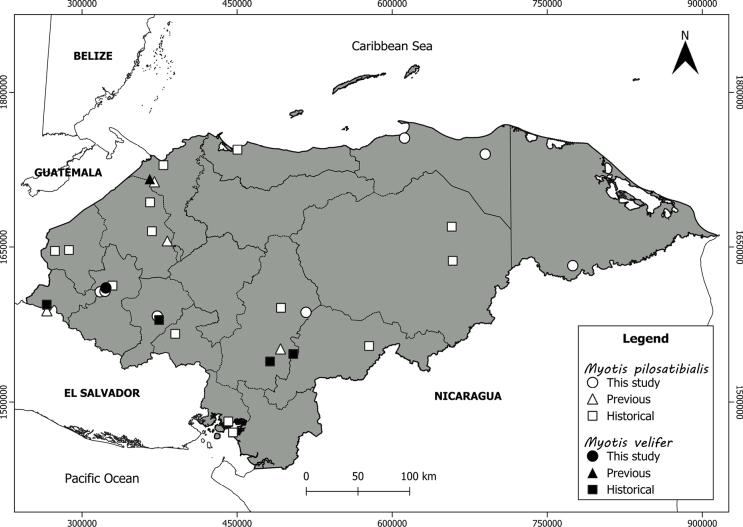
Distribution of *Myotis pilosatibialis* and *M. velifer* in Honduras.

Atlántida, **Colón**, Copán, Cortés, El Paraíso, Francisco Morazán, **Gracias a Dios**, La Paz, Lempira, Olancho, Santa Bárbara, and Valle (Fig. 87); 230–2080 m a.s.l.

###### Acoustic identification.

Call shapes are typically reversed J vespertilionid pulses. Relatively short broadband FM decreasing frequency components, which usually will have a small hook or upturned end of the pulse (called a toe in most Neotropical species of *Myotis*). 90% of the Fc pulses of the dominant harmonic are 64 kHz.

###### Additional notes.

Previous records of this species may have been identified as *M. nigricans* (Schinz, 1821) or *Myotis keaysi* J. A. Allen, 1914. Global and national conservation status is NE.

##### 186. Myotis riparius

Taxon classificationAnimaliaChiropteraVespertilionidae

Handley, 1960

A9879997-EB0E-5B46-A9DD-5A5EAB025C9F

###### Natural history.

No ecological information from Honduras has been reported for this species.

###### Distribution.

Colón, Cortés, El Paraíso, Gracias a Dios, and Olancho (Fig. 87); 10–1015 m a.s.l.

###### Acoustic identification.

Call shapes are typically reversed J vespertilionid pulses. Relatively short broadband FM decreasing frequency components, which usually will have a small hook or upturned end of the pulse (called a toe in most Neotropical species of *Myotis*). 90% of the Fc pulses of the dominant harmonic are 56 kHz.

###### Additional notes.

Global conservation status is LC and NE in Honduras.

##### 187. Myotis velifer

Taxon classificationAnimaliaChiropteraVespertilionidae

(J. A. Allen, 1890)

852E6F4C-10D6-5559-ADBB-D6D09615C96B

###### Natural history.

No information is available on the Honduran population for this species.

###### Distribution.

Francisco Morazán, Intibucá, **Lempira**, and Ocotepeque (Fig. 87); 440–1400 m a.s.l.

###### Acoustic identification.

Call shapes are typically reversed J vespertilionid pulses. Relatively short broadband FM decreasing frequency components, which usually will have a small hook or upturned end of the pulse (called a toe in most Neotropical species of *Myotis*). 90% of the Fc pulses of the dominant harmonic are 48 kHz.

###### Additional notes.

Skeletal remains of a bat were identified as *M. cf. velifer* from McGrew Cave, Copan District, Woodman and Croft (2005). Global conservation status is LC and DD in Honduras.

####### Carnivora Bowdich, 1821

We follow Segura et al. (2013) for the taxonomic arrangement of the genus *Herpailurus*, and Patterson et al. (2021) in accepting the genus *Neogale* rather than *Mustela*.

####### Felidae Fischer de Waldheim, 1817

#### *Panthera* Oken, 1816

##### 188. Panthera onca

Taxon classificationAnimaliaCarnivoraFelidae

(Linnaeus, 1758)

CBA72673-92AB-5B8F-895F-8F63D99B4690

###### Natural history.

An early record is from a specimen (MVZ: Mamm:179920) reported from the Buena Vista Prop., in the Gulf of Fonseca, Choluteca. However, there is no metadata on the collector, location, or date. The potential collection date spanned **>**; 40 years, from 1907 to 1950. Given the uncertainty, we cannot verify that this record was from southern Honduras.

The first camera-trap record was from 2007 at the Sikre River, also near Brus Lagun in La Moskitia (Gonthier and Castañeda 2013). The next camera-trap record was of a female accompanied by a cub (1–3 months old) from the Jeannette Kawas National Park, in October 2011. Approximately 10 months later, and 8 kilometers northeast of the previous record, another image of the same female, accompanied by an adult-sized male, was taken.

We documented that larger prey species had been extirpated from this National Park and that jaguars were feeding on freshwater turtles, green iguanas, white-tailed deer, and, in one instance, a marine dolphin carcass (Castañeda et al. 2013). In an effort to get a historical perspective on the prey base in 2009, an elderly woman in the village of Agua Chiquita, near the Laguna de Los Micos, in Jeannette Kawas, was interviewed. She credibly described white-lipped peccaries in the area and recalled that her husband had hunted them when she was younger. It appears that Tapirs and collared peccaries were extirpated from this park 50 and 20 years ago, respectively. Extensive camera-trap surveys in the park from 2010 to 2021 did not record any of these species.

In 2022, Panthera NGO, in conjunction with the government, launched a reintroduction of collared peccaries to the Jeannette Kawas National Park. Twenty-three individuals were released between 2022 and 2023 and were monitored using VHF radio telemetry, camera traps, and tracks. By 2026, the original group had split into at least four herds, occupying an area of ~ 17 km^2^. In 2011, a camera-trap survey covered ~ 100 km^2^, using 63 stations for two months. Camera trap data provided information on jaguars in the park: three adult females, two adult males, and one cub. One female was noted to have lost one eye. In December 2011, a pair spent > 72 hours mating in the range of a video camera station. More than 200 sexual encounters were noted, culminating with the male biting the back of the female's neck at the end of each encounter.

In April of 2023, a melanistic jaguar was documented in Honduras, reputedly the first report for northern Central America (Martínez et al. 2023). Although in 2010, a melanistic jaguar was said to have been killed in the village of Rus Rus, confirmed by reputable observers. No skin was recovered, nor were photos available to confirm this record.

However, Silva (2017) citing a photographed of a black female in 2004, from the El Fuerte River Valley, near Sinaloa, Mexico (Dinets and Polechla 2005), two records from Belize: Ek Balan and El Rancho Grande River and one record from Costa Rica (Bolaños et al. 2015) suggests the 2023 Honduran record was not actually the first for Central America.

Prior to the ban of the commercial pelt trade under Appendix I of CITES in 1975, many hunters worked full-time in the jungles of Honduras collecting jaguar pelts. There were two main collection centers in La Moskitia, one in Puerto Lempira and one in Ahuas. Some hunters worked for one or two decades building the family's patrimony. These hunters often provide insights into the numbers and distribution of these big cats. One such hunter claimed he had killed > 500 spotted cats (both jaguars and ocelots) for two decades. He noted that he initially hunted in Nicaragua in the 1960s, but as jaguars became scarce, he moved to Honduras. He also noted that he had seen melanistic individuals only five times, all in Nicaragua.

Although the anecdote from the same hunter was not credible, it was interesting. He described a method to take jaguars, which consisted of killing a spider monkey, dragging its carcass across the jungle for ~ 1 km. Then he would nail it to a tree and insert dynamite into the body with a movement-sensitive tiger. He would then move a safe distance away and wait in his hammock until he heard the explosion. When asked whether other monkey species or bait prey could be used, he strongly replied that “it had to be a spider monkey”.

The first confirmed jaguar record for the binational Merendón Mountain Range of Honduras and Guatemala was in 2015 at Cerro El Jilinco, the highest peak within Cusuco National Park. A young male was the first photographic evidence of the species in the region, despite extensive wildlife surveys conducted over many years.

In February 2026, a second young male jaguar was recorded in the same mountain range, more than a decade after the initial detection. To date, these two young males constitute the only confirmed jaguar records for the entire Merendón Range over more than ten years of systematic and intensive monitoring efforts. The scarcity of records suggests that the area is not currently supporting a resident jaguar population; rather, it likely functions as a dispersal corridor used by transient individuals moving between larger source populations. Nevertheless, these records highlight the conservation importance of the Merendón Range for maintaining landscape connectivity and facilitating gene flow among jaguar populations in northern Central America.

###### Distribution.

**Figure 88.**
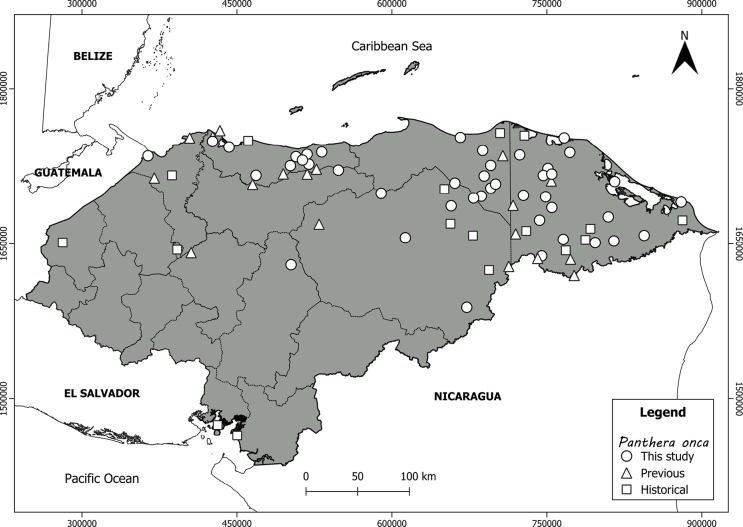
Distribution of *Panthera onca* in Honduras.

Atlántida, Choluteca, Colón, Copán, Cortés, Comayagua, Francisco Morazán, Gracias a Dios, Olancho, Valle, and Yoro (Fig. 88), at elevations of 0–2200 m a.s.l. Records from southern Honduras (Choluteca and Valle) are historical.

###### Additional notes.

Camera trap data provided information on jaguars in the park:. Wells (1857) reported jaguars on the island of Amapala, Golfo de Fonseca, in the mid-19^th^ century and noted that the Olancho government offered bounties for their elimination, as they were deemed a significant economic burden on cattle ranches. In March 2015, one juvenile male jaguar was killed near the town of Orica, Francisco Morazán. They are known as limni bulni or limni tara by the Miskitus in eastern Honduras or limni sixa for a melanistic phase. There is also evidence of jaguar poaching near the city of San Pedro Sula, as well as at Lake Yojoa, Santa Bárbara, and Copán during the 1950s and 1960s. Global conservation status is NT and CR in Honduras.

#### *Leopardus* Gray, 1842

##### 189. Leopardus wiedii

Taxon classificationAnimaliaCarnivoraFelidae

(Schinz, 1821)

D30D2EB7-07B2-51E2-AD63-1BB0A8109BB7

###### Natural history.

It is active both during the day and night. López Gallego et al. (2021) reported an individual opportunistically feeding on a a netted bat, *A. jamaicensis* late at night in northwestern Honduras. The margay is one of the most arboreal felids in Honduras, although much of its ecology remains poorly documented. Dietary observations include individuals feeding on Virginia Opossums (*Didelphis* spp.), while records from the Cusuco National Park indicate that rodents also constitute an important component of the species’ diet. In April, a female accompanied by a dependent offspring was observed along the road connecting the Merendón mountain range to Cusuco National Park.

###### Distribution.

**Figure 89.**
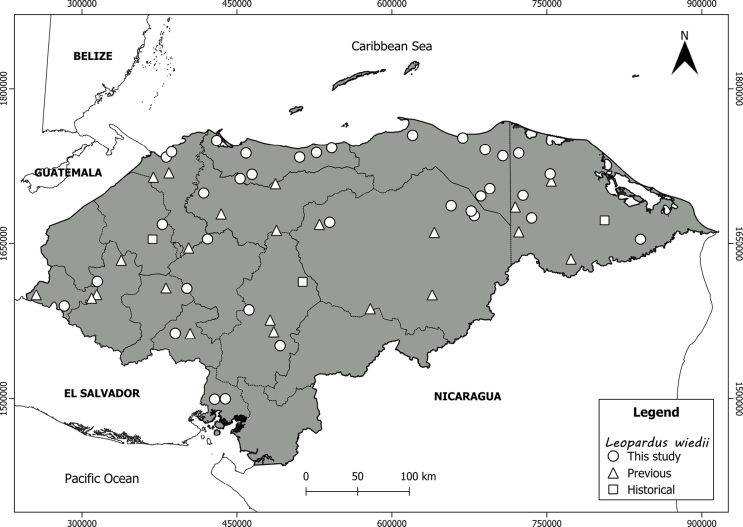
Distribution of *Leopardus wiedii* in Honduras.

**Atlántida**, **Comayagua**, **Colón**, Cortés, Francisco Morazán, Gracias a Dios, Intibucá, La Paz, Lempira, Ocotepeque, Olancho, Santa Bárbara, **Valle**, and Yoro (Fig. 89); 150**–**2135 m a.s.l.

###### Additional notes.

They are known as limi wayata among the Miskitus in eastern Honduras (Jones 1965) and also as limni sirpi. In general, it is known as gato de monte or tigrillo. Global conservation status is NT and VU in Honduras.

##### 190. Leopardus pardalis

Taxon classificationAnimaliaCarnivoraFelidae

(Linnaeus, 1758)

26308179-E4FA-5A04-BC7C-1B477A6C6715

###### Natural history.

It is primarily nocturnal. It is sympatric with *L. wiedii* in other regions, with which it is most often confused. The ocelot is one of the most common medium-sized felids in eastern Honduras and frequently occurs near agricultural areas. In Cusuco National Park, we observed females with two cubs in March, and feeding on *Peromyscus* spp. and *T. naudicaudus*.

###### Distribution.

**Figure 90.**
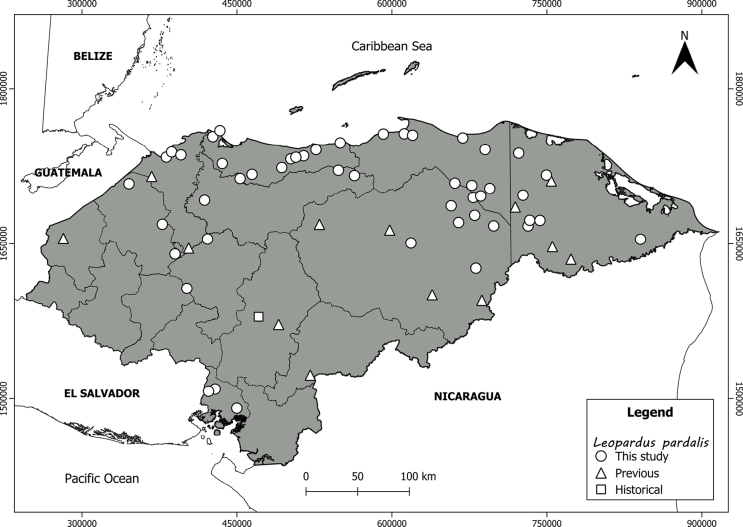
Distribution of *Leopardus pardalis* in Honduras.

**Atlántida**, **Colón**, Comayagua, Copán, Cortés, El Paraíso, Francisco Morazán, Gracias a Dios, Intibucá, Olancho, **Santa Bárbara**, **Valle**, and **Yoro** (Fig. 90); 250–1550 m a.s.l.

###### Additional notes.

They are known as kruhbu by the Miskitus in eastern Honduras and as limi sirpi (Jones 1965), and in Spanish are known as ocelots, tigrillos, and gatos de monte. Unlike several herbivorous mammals that can be deterred by artificial lighting systems, Ocelots appear largely unaffected by such measures and continue to move through agricultural landscapes regardless of light presence. This behavioral tolerance suggests that light-based deterrents are unlikely to reduce Ocelot activity in areas where conflicts with domestic animals occur. Global conservation status is LC and VU in Honduras.

#### *Puma* Jardine, 1834

##### 191. Puma concolor

Taxon classificationAnimaliaCarnivoraFelidae

(Linnaeus, 1771)

923E7A87-B162-59FD-80EF-29F85D79E829

###### Natural history.

Activity includes both diurnal and nocturnal. Wells (1857) reported that pumas were hunted in Olancho for their meat and sometimes competed with coyotes; he documented conflicts over cattle in Olancho; and he noted that they preferred the mountains and the country’s Pacific slope. Camera-trap data confirmed the presence of juvenile pumas, indicating reproduction in the dry forest of southern Honduras near the border with the Republic of El Salvador. Recently, HDAP recorded two pumas in unprotected areas of southern Honduras.

###### Distribution.

**Figure 91.**
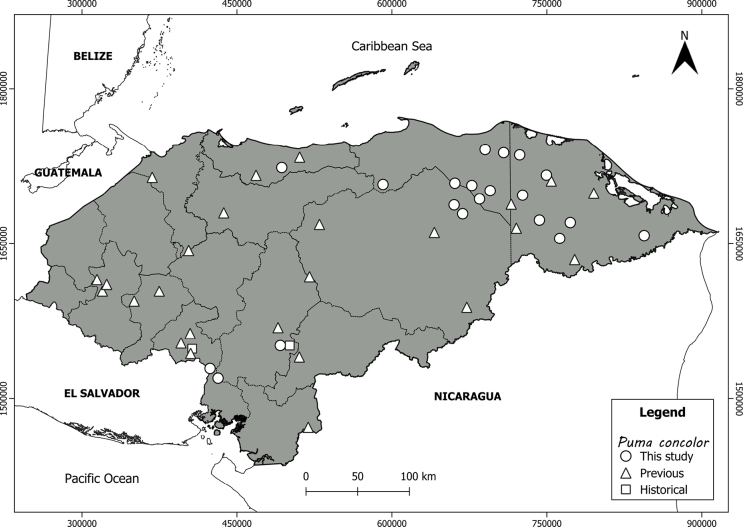
Distribution of *Puma concolor* in Honduras.

Atlántida, Choluteca, **Colón**, Comayagua, Cortés, El Paraíso, Gracias a Dios, Intibucá, Francisco Morazán, La Paz, Lempira, Olancho, **Valle**, and Yoro (Fig. 91); 250–2095 m a.s.l.

###### Additional notes.

Sánchez et al. (2023) list records for western Honduras, specifically in the department of Lempira, based on a specimen collected in 1946 by Archie F. Carr (MCZ43013) in San Francisco, Yeguare River Valley; however, the record should actually be attributed to Francisco Morazán. Recently, Peñalva Morales et al. (2025) confirmed the presence of pumas in Celaque National Park, Department of Lempira, using camera traps. The distribution of big cats in western Honduras remains poorly understood (see Castañeda et al. 2023). However, based on our review of unpublished records from the region, we believe these large cats inhabit the area, despite the lack of targeted surveys. They are known as limni pauni among the Miskitus in eastern Honduras. Historical records refer to them as *Felis concolor* Linnaeus, 1771. Global conservation status is LC and EN in Honduras.

#### *Herpailurus* Severtzov, 1858

We follow Segura et al. (2013) for the taxonomic rearrangement of the genus *Herpailurus*.

##### 192. Herpailurus yagouaroundi

Taxon classificationAnimaliaCarnivoraFelidae

(É. Geoffroy Saint-Hilaire, 1803)

1778F4D6-581E-5F58-817D-F056C10F37E1

###### Natural history.

The detection rate from camera traps was reported to be low, at 0.57 per 100 trap-nights (Harmsen et al. 2024). However, in most studies, cameras were mounted higher to primarily detect large felids (jaguars and pumas) and were unlikely to be triggered by these smaller cats, thereby likely under representing the true density.

###### Distribution.

**Figure 92.**
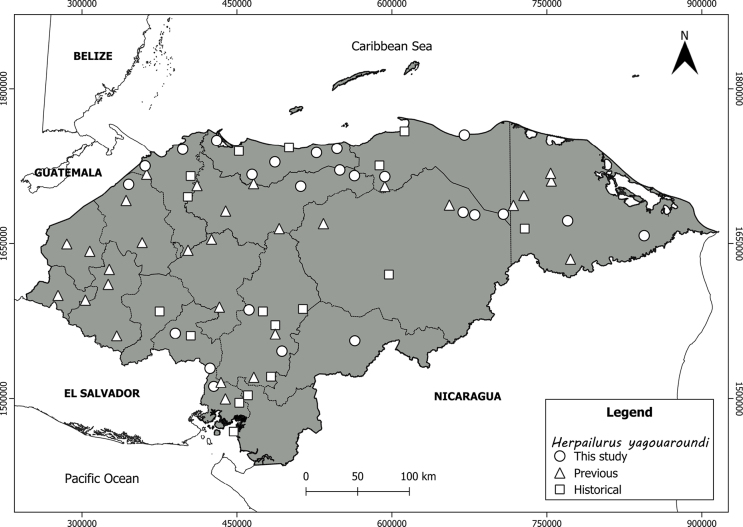
Distribution of *Herpailurus yagouaroundi* in Honduras.

Atlántida, Choluteca, Colón, Comayagua, Copán, Cortés, **El Paraíso**, Francisco Morazán, Gracias a Dios, Intibucá, La Paz, Lempira, Ocotepeque, Olancho, Santa Bárbara, Valle, and Yoro (Fig. 92); 150–2050 m a.s.l. It is the only Ifelid that is documented throughout the country.

###### Additional notes.

Historical records refer to this species as *Puma yagouaraoundi* (É. Geoffroy Saint-Hilaire, 1803) or *Felis yagouaraoundi* (Desmarest, 1816). Aceituno (2016) described the courtship of this species in captivity in Honduras. The jaguarundi exhibits a similar response to artificial lighting as the ocelot. Camera-trap records from eastern Honduras indicate that individuals continue to use areas equipped with light-based wildlife deterrent systems, suggesting that these measures do not significantly alter movement patterns. The species’ apparent indifference to artificial illumination may reflect its ecological flexibility and broad habitat tolerance across both natural and human-modified environments. It has the highest roadkill count of the five cats in the country. Global and national status is NT.

####### Canidae G. Fischer von Waldheim, 1817

#### *Canis* Linnaeus, 1758

##### 193. Canis latrans

Taxon classificationAnimaliaCarnivoraCanidae

Say, 1823

B4E61685-C053-59EA-B9B7-895DAA3E60DF

###### Natural history.

They are usually recorded as solitary animals, but groups of up to three adults have been recorded by camera traps, including eating bananas. Females with two pups have been seen in May and September. The deer *O. virginianus* are often preyed on by coyotes; also, a group of coyotes lured a puma, enticing it to climb a tree, waiting for it, and bringing it down, possibly to devour it.

###### Distribution.

Atlántida, Comayagua, Cortés, El Paraíso, Francisco Morazán, La Paz, Lempira, Ocotepeque, Olancho, Valle, and Yoro (Fig. 15); 10–1820 m a.s.l.

###### Additional notes.

Historical specimens are referred to as *Canis hondurensis* Goldman, 1936. Individuals have been found as roadkill on main roads in eastern Honduras. In 2018, individuals were reported for the first time to have reproduced in captivity in Honduras, giving birth to two females and two males. The coyote has undergone a notable expansion throughout Honduras during recent decades. Evidence suggests that the species is expanding into new areas across the country and becoming more common in varied habitats, despite remaining uncommon in dense forest habitats. Camera-trapping in Jeannette Kawas National Park from 2012 to 2026 provided the first confirmed record in 2021: an old adult male, likely a dispersing individual. In 2021, coyotes were also documented in PANACAM, where puma activity is common. Camera trap surveys conducted from May 2018 to August 2019 in the Montaña de Comayagua National Park, Montecillos Biological Reserve, Uyuca Biological Reserve, Texiguat Wildlife Refuge, and the Volcán Pacayitas Biological Reserve collected 1,056 photographs during 8,657 trap nights, with coyotes accounting for 36.9% of carnivore records. Global conservation status is LC and NE in Honduras.

#### *Urocyon* Baird, 1857

##### 194. Urocyon cinereoargenteus

Taxon classificationAnimaliaCarnivoraCanidae

(Schreber, 1775)

222BB22D-275A-5035-8945-787964A5A7D7

###### Natural history.

Dietary observations include the consumption of fruits from the family Sapotaceae, highlighting the species’ omnivorous and opportunistic feeding habits. Most camera-trap records consist of solitary individuals or pairs, which is consistent with the species’ generally solitary behavior outside the breeding season. Reports from residents indicate that gray foxes occasionally enter residential areas on the outskirts of Tegucigalpa, reflecting the species’ adaptability to suburban environments.

###### Distribution.

**Figure 93.**
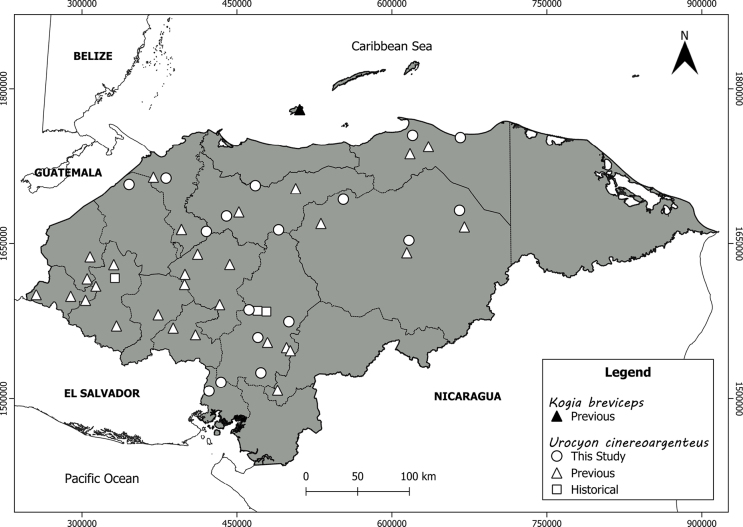
Distribution of *Kogia breviceps* and *Urocyon cinereoargenteus* in Honduras.

Copán, **Cortés**, El Paraíso, Francisco Morazán, La Paz, Lempira, Ocotepeque, **Olancho**, Santa Bárbara, Valle, and Yoro (Fig. 93); 70–2050 m a.s.l.

###### Additional notes.

It is a species associated with human-livestock conflicts in rural areas, likely involving chickens and other small animals. They are often known as zorras or comadrejas. The gray fox is a widespread mesocarnivore in Honduras that occupies a broad range of habitats, from highly disturbed landscapes to relatively well-preserved forests. Although commonly associated with human-modified environments, recent records demonstrate that the species also occurs within the core zones of several protected areas, including the Sierra de Agalta National Park, the Corralitos Wildlife Refuge, and forested areas adjacent to the Texiguat Wildlife Refuge. Road mortality appears to be an important threat, with individuals documented along the Southern Dry Canal corridor, secondary dirt roads between Olancho and Yoro, and, notably, a road-killed individual recorded in 2025 on the Tegucigalpa Ring Road (Anillo Periferico) within a highly urbanized landscape near the recently established Suyapa Luis Hernán Baca Valladares ‘Mero’ Wildlife Refuge. Global conservation status is LC and NE in Honduras.

####### Mephitidae Bonaparte, 1845

All four skunk species are likely killed for medicinal purposes. The following comments apply to all species, but are most frequently observed in *Mephitis macroura* Lichtenstein, 1832, and the genus *Conepatus* Gray, 1837. Local people usually kill skunks based on the belief that their fat has medicinal purposes, usually related to pulmonary diseases. In Central America, in the 1930s, cough and flu syrups typically included skunk fat (Elvir Valle and Marineros Sanchez 2021). Camera traps documented three of the four species (*Conepatus leuconotus* (Lichtenstein, 1832), 1832, *M. macroura*, and *Spilogale angustifrons* Howell, 1902) at the same water source in southern Honduras during the dry season.

#### *Conepatus* Gray, 1837

Molinari (2026) proposed taxonomic changes for the genus *Conepatus*, building upon the hypothesis of Rodrigues (2013), who suggested the existence of two distinct taxonomic units in Central America separated by the densely forested regions of Nicaragua and Honduras. Under this treatment, *Conepatus semistriatus* (Boddaert, 1785) is restricted to mid- to high-elevation areas of Colombia and Venezuela, whereas populations from northern Central America, including Honduras, are assigned to *Conepatus conepatl* Gmelin, 1788. Although this revised taxonomy is plausible, we prefer to adopt a conservative treatment until the proposal gains broader acceptance and is supported by additional morphological, molecular, and biogeographic evidence. Therefore, we retain the species currently recognized for Honduras, *C. leuconotus* and *C. semistriatus*. Nevertheless, we agree with Molinari (2026) and Rodrigues (2013) that *Conepatus* populations in northern Central America warrant comprehensive taxonomic studies to clarify their evolutionary relationships and species boundaries.

##### 195. Conepatus leuconotus

Taxon classificationAnimaliaCarnivoraMephitidae

(Lichtenstein, 1832)

2BAA8E1A-0907-5974-8C85-7F721DFFEB6B

###### Natural history.

It has been recorded at ponds in the dry season in southern Honduras. This skunk is very poorly documented.

###### Distribution.

**Figure 94.**
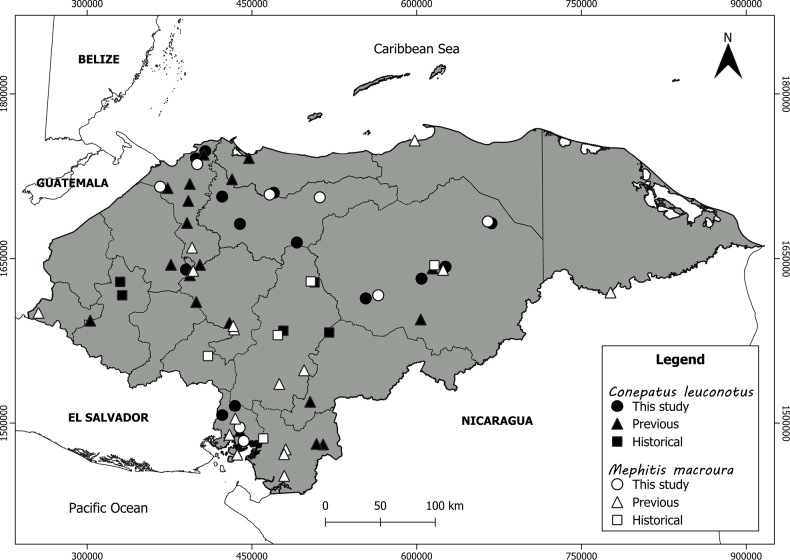
Distribution of *Conepatus leuconotus* and *Mephitis macroura* in Honduras.

Choluteca, Cortés, El Paraíso, Francisco Morazán, Lempira, Olancho, Santa Bárbara, **Valle**, and **Yoro** (Fig. 94); 150–1750 m a.s.l.

###### Additional notes.

Historical specimens from Honduras are referred to as *Conepatus mesoleucus* (Lichtenstein, 1832) or *Conepatus nicaraguae* J. A. Allen, 1910. Referred by Goodwin (1942a) as *Conepatus nicaraguae* J. A. Allen, 1910. Roadkill is one of the main threats to the species in the country. Elvir Valle and Marineros Sanchez (2021) reported that in western Honduras, they are eaten in stew, and native people in northern (Yoro) and central (Francisco Morazán) also confirmed that they eat them in stew after removing the fat. We have confirmed that this species is affected by roadkills. Global conservation status is LC and NE in Honduras.

##### 196. Conepatus semistriatus

Taxon classificationAnimaliaCarnivoraMephitidae

(Boddaert, 1785)

979125A5-F47F-5551-95D1-DE4564404E36

###### Natural history.

We consider it the least known skunk in Honduras. It is restricted to the Caribbean slope of the country.

###### Distribution.

Historically, it is known only from Colón, but we have additional records for **Atlántida**, **Colón**, **Cortés**, Olancho, Gracias a Dios, and Yoro (Fig. 83) at elevations of 220–1900 m a.s.l. The species may also occur in Santa Bárbara.

###### Additional notes.

Occasionally is affected by roadkills. Global conservation status is LC and NE in Honduras.

#### *Mephitis* É. Geoffroy Saint-Hilaire & F. G. Cuvier, 1795

##### 197. Mephitis macroura

Taxon classificationAnimaliaCarnivoraMephitidae

Lichtenstein, 1832

E9898C8B-6AC5-5DB7-A39A-5987FB711C4E

###### Natural history.

The species’ home range varies from 3.4–32.4 ha on the campus of a university in Valle del Yeguare, Francisco Morazán (Mora et al. 2010). It has been recorded at ponds in the dry season in southern Honduras. We have found individuals eating the fruits of *Crescentia alata* Kunth and in southern Honduras they are associated with this type of tree. They are most often trapped when sardines are used as bait. The species is relatively frequently recorded in the Cusuco National Park, and *M. macroura* exhibits greater levels of daytime activity than other skunk species occurring in Honduras, a pattern repeatedly documented through direct observations and camera-trap records.

###### Distribution.

**Atlántida**, **Choluteca**, **Comayagua**, Francisco Morazán, Olancho, Valle, and Yoro (Fig. 94); 170–555 m a.s.l.

###### Additional notes.

The Hooded Skunk is particularly common in seasonally dry environments of southern Honduras, where it frequently enters human dwellings and agricultural areas. It is a common road-kill in Valle del Yeguare and on the main road in the Yoro department. In the department of El Paraíso, individuals have been observed using burrows located in heavily cracked and desiccated soils, indicating an affinity for xeric habitats. Global conservation status is LC and NE in Honduras.

#### *Spilogale* Gray, 1865

##### 198. Spilogale angustifrons

Taxon classificationAnimaliaCarnivoraMephitidae

Howell, 1902

8CCDCEFE-1FB0-560F-B8E8-0B4DD8F5084D

###### Natural history.

They have been observed at ponds in southern Honduras during the dry season. Young individuals were captured in August. Although this species is generally uncommon in camera-trap surveys across Honduras, it seems to exist at moderate densities in the Uyuca Biological Reserve and in pine forests near Langue, Valle. Most detections involve solitary individuals, and there are no confirmed records of pairs traveling together from the camera-trap surveys.

###### Distribution.

Atlántida, Comayagua, Cortés, El Paraíso, Francisco Morazán, Intibucá, La Paz, Lempira, Santa Bárbara, Valle, and **Yoro** (Fig. 83); 20–1950 m a.s.l.

###### Additional notes.

Five specimens reported in GBIF.org (2023) were identified as *Spilogale putorius* (Linnaeus, 1758): one from Intibucá (AMNH M-126181) and four from Francisco Morazán (AMNH M-123381, 126187, 130031; MCZ 43009). However, they are likely *S. angustifrons*; confirmation is required. Global conservation status is LC and NE in Honduras.

####### Mustelidae G. Fischer von Waldheim, 1817

#### *Eira* C. E. H. Smith, 1842

##### 199. Eira barbara

Taxon classificationAnimaliaCarnivoraMustelidae

(Linnaeus, 1758)

EF724584-EDFF-51E4-A512-9BCD9B239366

###### Natural history.

The tayra is one of the most adaptable and behaviorally versatile carnivores in Honduras. Individuals have been observed moving through the forest canopy at heights exceeding 10 m above ground, reflecting their exceptional climbing abilities. Camera-trap records indicate that the dark-bodied morph with a pale head is the most frequently observed coloration pattern throughout the country, whereas completely melanistic individuals have thus far been documented only in the department of Yoro. Although most camera-trap detections consist of solitary individuals, the species can occasionally occur in social groups. An unusual observation in Santa Bárbara involved four individuals displaying aggressive behavior toward a field team near a forest stream, repeatedly blocking passage through the area. Such encounters support reports that Tayras can exhibit highly territorial and defensive behavior when protecting resources or group members. We have recorded an individual feeding on *Cyclopes dorsalis*.

###### Distribution.

Atlántida, Comayagua, Copán, Colón, Cortés, Francisco Morazán, Gracias a Dios, Ocotepeque, Olancho, **Santa Bárbara**, and Yoro (Fig. 72); 400–1890 m a.s.l.

###### Additional notes.

According to folklore, the two color morphs, a lighter (whitish) one, is considered benevolent, and the darker (black) one is regarded as malevolent. Therefore, encountering a black form is often associated with bad luck. The species is also commonly linked to conflicts with domestic poultry. They are usually called cadejos or comadrejas. Global conservation status is LC and NT in Honduras.

#### *Galictis* Bell, 1826

##### 200. Galictis vittata

Taxon classificationAnimaliaCarnivoraMustelidae

(Schreber, 1776)

3995C351-B914-5FA1-AF2D-03D7553D2AEB

###### Natural history.

It has been recorded in secondary riparian forests surrounded by pineapple fields and oil palm plantations. We consider that is an uncommon mustelid throughout Honduras, although local concentrations may occur in suitable habitats. Camera-trap records occasionally document up to three individuals at a single station, suggesting that small family groups or temporary social associations may occur. Most records were along streams and riparian corridors, particularly within the Merendón mountain range (northwestern Honduras), where the species appears to be more frequently detected than elsewhere in the country.

###### Distribution.

**Figure 95.**
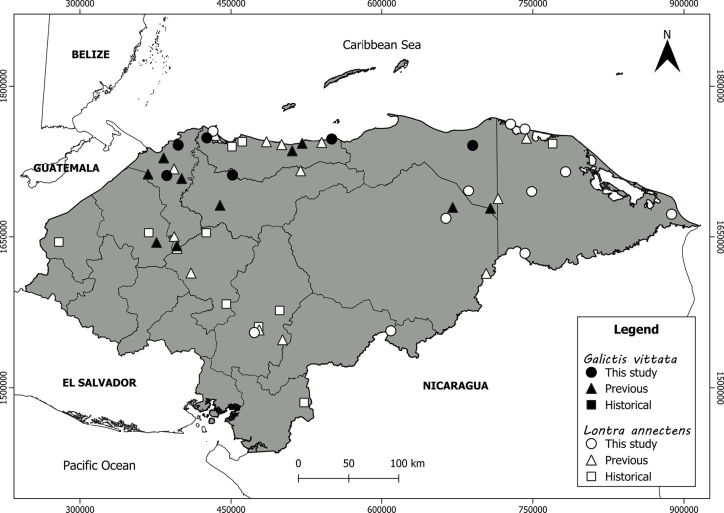
Distribution *Galictis vittata* of and *Lontra annectens* in Honduras.

Atlántida, **Colón**, Cortés, Olancho, Santa Bárbara, and **Yoro** (Fig. 95); 10–1450 m a.s.l. It is likely to be found in other regions in the Caribbean lowlands of Honduras.

###### Additional notes.

This species is often roadkill however, it is poorly known. Martínez and Sánchez (2020) confirmed the presence of *G. vittata* in the Río Plátano Biosphere Reserve, where an individual was kept as a pet. Illegal possession as a pet has also been documented, and wildlife authorities have confiscated captive individuals in Puerto Cortés. Despite its wide distribution, the ecology and population status of *G. vittata* in Honduras remain poorly understood due to the species’ naturally low detection rates and secretive behavior. Global conservation status is LC and NT in Honduras.

#### *Lontra* Gray, 1843

Based on new evidence presented by de Ferran et al. (2024), the species that occurs in Mesoamerica is *Lontra annectens* (Forsyth Major, 1897).

##### 201. Lontra annectens

Taxon classificationAnimaliaCarnivoraMustelidae

(Major, 1897)

F375852F-9C5F-56DA-822C-2B21568EF358

###### Natural history.

Reports indicate that several individuals have died in the large rivers of Francisco Morazán, including the Río Grande and Río Choluteca. An apparently unstudied population occurs upstream of the Los Laureles Reservoir in Francisco Morazán. Aceituno et al. (2015) reported measurements of a juvenile. It frequently creates conflicts with local shrimp farms and fish-farming operations (e.g., tilapia) within its range, but especially in Trojes (El Paraíso). The northern neotropical otter is infrequently detected by camera traps in Honduras despite being locally present in suitable aquatic habitats. Records have been obtained in the vicinity of wetlands within the Jeannette Kawas National Park, as well as in the Apalca lagoons, a complex of water bodies surrounded by seasonally flooded forest. Interestingly, several detections in these lagoons were obtained from camera-trap stations originally established to monitor jaguar movements, suggesting that otters may regularly use travel routes shared with other large vertebrates.

###### Distribution.

Atlántida, Choluteca, **Colón**, Copán, Cortés, **El Paraíso**, Francisco Morazán, Gracias a Dios, Olancho, Santa Bárbara, and Yoro (Fig. 95); 0–940 m a.s.l.

###### Additional notes.

Historical records from Honduras refer to the northern neotropical otter as either *Lutra longicaudis* (Olfers, 1818) or *Lontra longicaudis* (Olfers, 1818). They are known as mamu by the Miskitus in eastern Honduras (Jones 1965). Global conservation status is NT and VU in Honduras, referring to *L. longicaudis*.

#### *Neogale* Gray, 1865

##### 202. Neogale frenata

Taxon classificationAnimaliaCarnivoraMustelidae

(Lichtenstein, 1831)

62E7D318-2417-58C7-A376-3FD563C0547E

###### Natural history.

It has been recorded in urban and suburban areas of Francisco Morazán, where it is sometimes killed on roads and occasionally enters houses. Additional records include a daytime observation along a seasonal stream near Sabanagrande in southern Honduras. Reports from central and western Honduras further indicate that the species occasionally enters suburban areas and human dwellings, demonstrating a degree of tolerance to anthropogenic environments. It feeds on rodents in the genera *Peromyscus* and *Sigmodon*, as well as rabbits.

###### Distribution.

Comayagua, Copán, Cortés, Intibucá, El Paraíso, Francisco Morazán, Gracias a Dios, Lempira, **La Paz**, and Olancho (Fig. 80); 950–2000 m a.s.l.

###### Additional notes.

We follow Patterson et al. (2021) for the genus *Neogale*, rather than using *Mustela* Linnaeus, 1758. Historical specimens have been referred to as *Mustela frenata* Lichtenstein, 1831, and are commonly known as comadrejas. The Long-tailed Weasel (*Neogale frenata*) remains one of the most difficult small carnivores to document in Honduras. Its rapid movements and small body size likely contribute to its underrepresentation in camera-trap surveys. One of the first camera-trap records for the species was obtained in the northern sector of the Cusuco National Park (also, see Hoskins et al. 2018). Most records, however, derive from opportunistic sightings. For example, personnel from the Honduran HDAP observed an individual crossing the Merendón highway during daylight hours. Global conservation status is LC and NE in Honduras, referring to *M. frenata*.

####### Procyonidae Gray, 1825

#### *Bassaricyon* J. A. Allen, 1876

##### 203. Bassaricyon gabbii

Taxon classificationAnimaliaCarnivoraProcyonidae

J. A. Allen, 1876

BD7D06A0-FEC6-5F42-937B-E810B1886332

###### Natural history.

It was observed an individual searching for epiphytes in a pine tree *P. oocarpa*. A new record for Yoro is presented in Uchapa-Pimienta Micro-watershed, where I found it sleeping during the day in an open pine forest.

###### Distribution.

**Figure 96.**
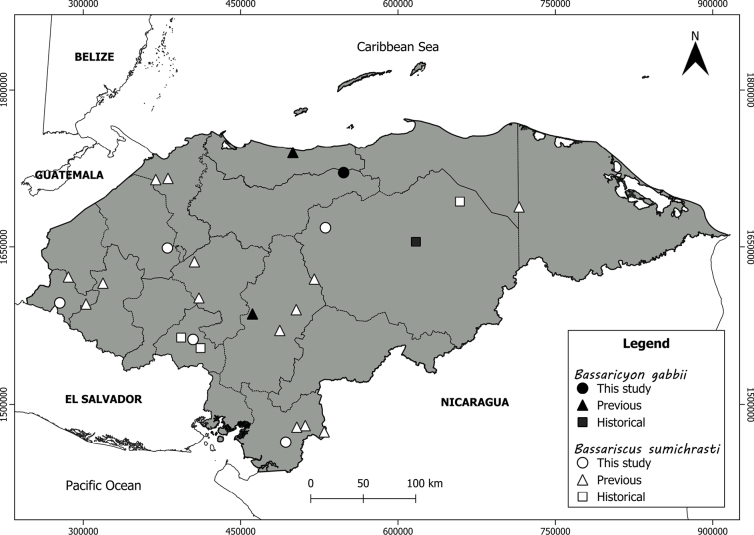
Distribution of *Bassaricyon gabbii* and *Bassariscus sumichrasti* in Honduras.

Atlántida, Francisco Morazán, Olancho, and **Yoro** (Fig. 96). It is only known from three protected areas, Sierra de Agalta National Park, Cuero y Salado Wildlife Refuge, and Corralitos Wildlife Refuge; 50–1950 m a.s.l.

###### Additional notes.

It is considered to be the least well-known procyonid in the country. This species has likely been misidentified as *Potos flavus* Schreber, 1774, in some regions of the country (Turcios-Casco et al. 2023c). Deforestation due to cattle ranching, poaching, and logging are the main threats. Occurrence in Honduras requires further study, as it is currently known only from the three protected areas noted above. Global conservation status is LC and DD in Honduras.

#### *Bassariscus* Coues, 1887

##### 204. Bassariscus sumichrasti

Taxon classificationAnimaliaCarnivoraProcyonidae

(Saussure, 1860)

1E43BEEE-3556-517C-8267-8F9672EC111D

###### Natural history.

Although arboreal, it has been documented on the ground by camera traps and observed in the canopy at night. It has been recorded feeding on *Citrus* sp. on the ground. The cacomistle (*Bassariscus sumichrasti*) is another highly arboreal procyonid that is infrequently recorded on the ground. Nevertheless, terrestrial activity has been documented in several montane forests of Honduras, including the Cusuco National Park, the Montaña de Santa Bárbara National Park, and the Misoco Biological Reserve. Additional observations from non-protected landscapes indicate that ground use may occur under specific environmental conditions. For example, an individual was observed near a temporary pond on the ground during a heavy rainfall event in Opatoro, La Paz. In the El Niño year of 1998, one individual was observed passing through Campamento Naranjo, at 2560 m a.s.l., located near the top of the eastern slope of Cerro de las Minas, Celaque National Park, ca. 11 km SW of Gracias, Lempira (Woodman et al. 2012). Also was confirmed for PANACAM by Midence (2020a).

###### Distribution.

Choluteca, Comayagua, Copán, Cortés, La Paz, Lempira, Francisco Morazán, Gracias a Dios, Ocotepeque, Olancho, and **Santa Bárbara** (Fig. 96); 230–2185 m a.s.l.

###### Additional notes.

Global conservation status is LC and NT in Honduras.

#### *Nasua* Storr, 1780

##### 205. Nasua narica

Taxon classificationAnimaliaCarnivoraProcyonidae

(Linnaeus, 1766)

26D3AF9C-E708-5792-AEAB-77645DBE7491

###### Natural history.

They are active both diurnally and nocturnally. Groups of up to 14 individuals, including offspring, have been observed in central Honduras in February, with young also noted in January, June, and July. Within the Jaguar Corridor, it is frequently the most commonly detected species at camera-trap stations, often occurring in social groups of 15 or more individuals accompanied by juveniles. In the Jeannette Kawas National Park, the species represents an important prey resource for jaguars, particularly because other large-bodied prey species occur at relatively low densities.

###### Distribution.

**Figure 97.**
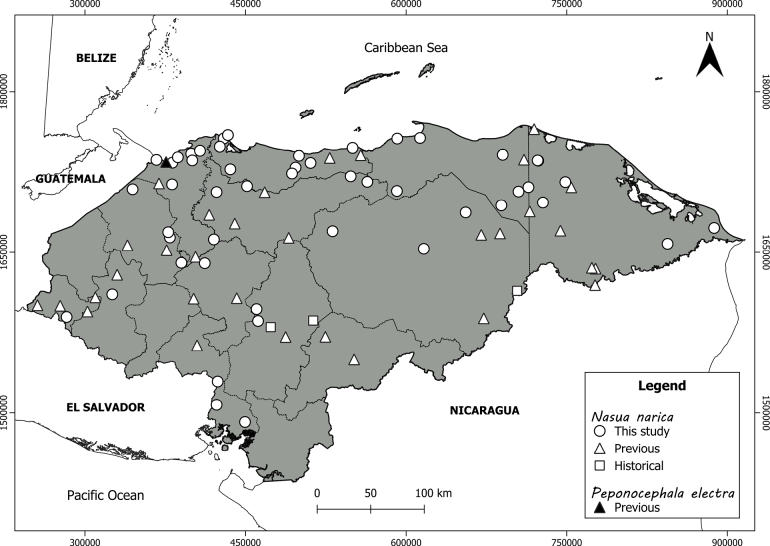
Distribution of *Nasua narica* and *Peponocephala electra* in Honduras.

Atlántida, Colón, Comayagua, Cortés, Francisco Morazán, El Paraíso, Gracias a Dios, Intibucá, La Paz, Lempira, Ocotepeque, Olancho, Santa Bárbara, **Valle**, and Yoro (Fig. 97); 0–2140 m a.s.l.

###### Additional notes.

Specimens in GBIF.org (2023) from La Paz, Olancho, Atlántida, two from Francisco Morazán (MCZ 29042 and MCZ 42788), one in Tela (UMNZ 55866), and one without a location (AMNH M-124870) are erroneously identified as *Nasua nasua* (Linnaeus, 1766). However, we included these individuals as *N. narica*. They are known as wistiting by the Miskitus in eastern Honduras. This species is occasionally roadkill on primary roads. Coatis readily adapt to human presence and frequently approach visitors in recreational and ecotourism areas in search of food. Records also indicate that individuals are occasionally captured and maintained as pets, a practice that may alter natural behaviors and increase human–wildlife conflicts. Global conservation status is LC and VU in Honduras.

#### *Potos* É. Geoffroy Saint-Hilaire & F. G. Cuvier, 1795

##### 206. Potos flavus

Taxon classificationAnimaliaCarnivoraProcyonidae

(Schreber, 1774)

31CB210E-DBC6-560F-828F-1F1220934FA0

###### Natural history.

While primarily arboreal, there are records of individuals on the ground. Up to four individuals have been observed during the same night. It has been found eating bird eggs in the canopy forest. Females with offspring have been observed in March. The Kinkajou is a predominantly arboreal and nocturnal procyonid that rarely descends to the ground. However, camera-trap surveys in the Cusuco National Park have repeatedly documented individuals traveling along forest trails at approximately 2,000 m a.s.l., suggesting that terrestrial movements may be more common in high-elevation cloud forests than previously recognized.

###### Distribution.

**Figure 98.**
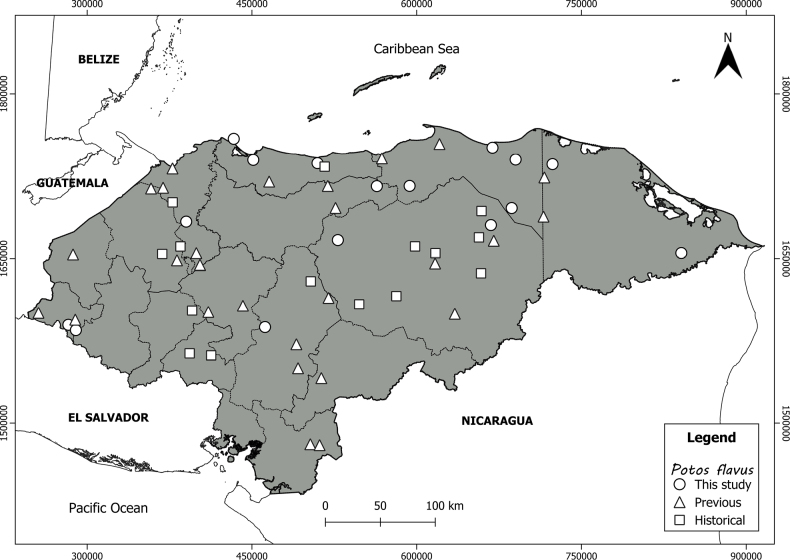
Distribution of *Potos flavus* in Honduras.

Atlántida, Choluteca, Colón, Comayagua, Copán, Cortés, El Paraíso, Francisco Morazán, Gracias a Dios, Intibucá, La Paz, Ocotepeque, Olancho, Santa Bárbara, and Yoro (Fig. 98); 210–2140 m a.s.l.

###### Additional notes.

They are known as uyuk by the Miskitus in eastern Honduras. Global conservation status is LC and NE in Honduras.

#### *Procyon* Storr, 1780

##### 207. Procyon lotor

Taxon classificationAnimaliaCarnivoraProcyonidae

(Linnaeus, 1758)

30F7F02C-A146-52C3-BC08-425FC7C1C87B

###### Natural history.

We have recorded groups of up to three individuals. They are considered pests in Islas de la Bahía and are associated with human-wildlife conflicts throughout the country, particularly on plantations, especially cornfields, and are closely associated with agricultural landscapes and human settlements in northern Honduras. In the municipalities of Omoa and Tela, the species is commonly hunted because of its frequent raids on maize fields and other crops. In some coastal communities, animals killed for crop protection are subsequently consumed, with local preparations including traditional dishes cooked with coconut.

###### Distribution.

All the departments (Fig. 35); 0–1750 m a.s.l. It seems that they have been introduced in Utila within the Islas de la Bahía department.

###### Additional notes.

They are known as suksuk by the Miskitus in eastern Honduras and Mapachín in other parts of Honduras. Raccoons have problems with plantations, especially cornfields. Global conservation status is LC and NT in Honduras.

####### Perissodactyla Owen, 1848

**Tapiridae Gray, 1821**

#### *Tapirus* Gray, 1821

Based on the Mammal Diversity Database (2026) taxonomy, we retain the genus Tapirus rather than the *Tapirella* Palmer, 1903 proposed by Groves and Grubb (2011), noting that the latter is based on limited morphological evidence and few phylogenetic evidence (see Holbrook 2013; Gutiérrez and Garbino 2018 for further discussions).

##### 208. Tapirus bairdii

Taxon classificationAnimaliaPerissodactylaTapiridae

(Gill, 1865)

2DD5653C-BC6C-5790-B04B-D67C321F17C4

###### Natural history.

This is the largest terrestrial mammal in Honduras (Meyer et al. 2022). It is primarily nocturnal, although diurnal activity has been recorded in areas with low poaching pressure. Reproduction in Honduras has been documented in April; camera-trap records from Cusuco National Park captured females accompanied by spotted calves; and in November in La Moskitia. In Olancho, Agalta National Park, a total of 35 plant species consumed by *Tapirus bairdii* were identified through direct field observations and fecal analysis. These food resources were recorded in primary and secondary forest stands and along forest edges, indicating habitat-use plasticity in foraging behavior. Analysis of undigested remains from 26 fecal samples revealed a predominance of fibrous material (60.5%), followed by leaves (18.6%), fine organic matter (11.6%), twigs (6.13%), and seeds (2.9%) (Estrada 2006a).

Baird’s tapir is a generalist frugivore that feeds on fruits, leaves, buds, and bark and plays a key role as a seed disperser in tropical forest ecosystems (Meyer et al. 2020). Two palatability trials conducted with a captive individual at the Zoológico Rosy Walther in Tegucigalpa evaluated 53 plant species; 46 species (86.8%) were accepted, and 16 were identified as having high potential for cultivation (López et al. 2022).

In eastern Honduras, there have been occasional reports of defensive behavior toward humans, which can lead to rare attacks. One documented incident involved a farmer who was injured while trying to hunt a tapir, indicating a defensive reaction (Estrada 2006b).

In western Honduras, the last known population persists within the Cusuco National Park, whereas the species is considered extirpated from the Merendón Reserve system, which no longer maintains functional connectivity with Cusuco. Consequently, the remaining western population may be geographically isolated. The species has also been absent from the Jeannette Kawas National Park for more than two decades and is considered locally extinct there. In contrast, relatively stable populations continue to occur throughout the Cordillera Nombre de Dios and the extensive forests of La Moskitia. Historically, tapirs were believed to be among the primary wildlife species responsible for damaging cassava fields in Indigenous communities of La Moskitia. Although occasional crop raiding does occur, recent field observations suggest that artificial light systems can function as effective deterrents, reducing tapir incursions into agricultural plots in southern sectors of the region.

###### Distribution.

**Figure 99.**
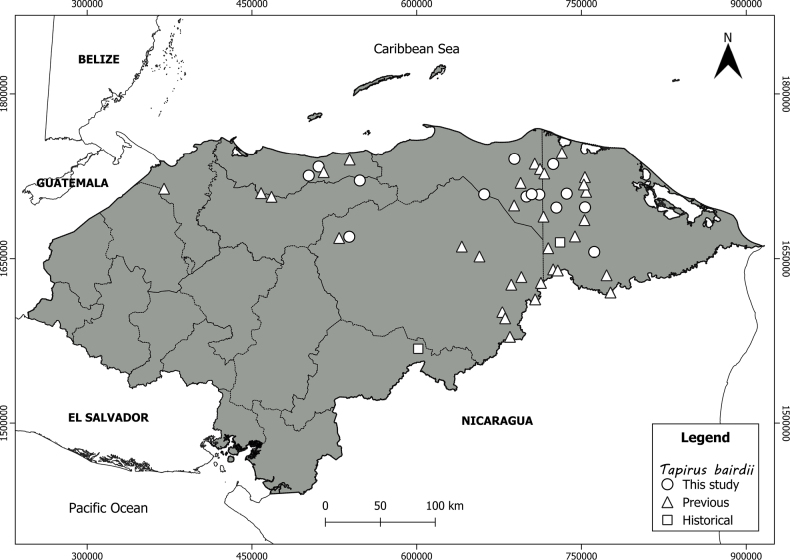
Distribution of *Tapirus bairdii* in Honduras.

Atlántida, Colón, Cortés, El Paraíso, Gracias a Dios, Olancho, Santa Bárbara, and Yoro (Fig. 99). Tapirs have been reported in the Nombre de Dios mountain range in northern Honduras. The population in the Cusuco National Park is likely the only remaining population in the northwestern region (Hoskins et al. 2018) and we recently documented within the same protected area for the department of Santa Bárbara. The largest population is in the Moskitia of eastern Honduras (Gonthier and Castañeda 2013; Schank et al. 2015). They still occur in some areas of Olancho (Estrada 2006a; Sagastume-Espinoza and Romero 2017). Although in lowland coastal areas such as the Jeannette Kawas National Park, it appears to have been extirpated around 50 years ago. Extensive camera-trap surveys during the past 13 years have failed to detect the species. Tapirs have not been recorded for decades in the Pacific region and protected areas of central and southwestern Honduras.

###### Additional notes.

They are known as tilba among the Miskitus in eastern Honduras (Jones 1965), Pánh-ka among the Tawahka, Dandei among the Garifuna, and Chajú among the Pesh. Illegal poaching, deforestation, and habitat loss are the primary threats to the species. More than a decade ago, McCann et al. (2012) cautioned that tapirs in Cusuco National Park (Honduras) faced extirpation within 10 years without significant conservation intervention. Today, thanks to a collaborative conservation effort led by PANTHERA, Wallacea, and ICF, and supported by the Honduran Armed Forces, which have patrolled > 20,000 km^2^, tapirs continue to thrive in this protected area. Global conservation status is EN and CR in Honduras.

####### Artiodactyla Owen, 1848

As Artiodactyla has priority over Cetartiodactyla, we follow Asher and Helgen (2010) and Burgin et al. (2018).

####### Cervidae Goldfuss, 1820

Wells (1857) noted that deer were very common throughout the country, that poachers could readily take them with rifles, and that local people distinguished between antelope and deer.

#### *Mazama* Rafinesque, 1817

##### 209. Mazama temama

Taxon classificationAnimaliaArtiodactylaCervidae

(Kerr, 1792)

84C0DD54-7C4B-5156-A484-76C25528C1B0

###### Natural history.

The Central American Red Brocket is a secretive forest deer that remains poorly studied in Honduras. Camera-trap records have documented females accompanied by offspring during February, suggesting that reproduction may occur during multiple periods of the year. In eastern Honduras, however, the species faces intense hunting pressure and is considered one of the most heavily targeted ungulates in many rural communities. Although typically regarded as solitary, groups of up to seven or eight individuals have occasionally been recorded, indicating that temporary aggregations may occur under certain ecological conditions.

###### Distribution.

**Figure 100.**
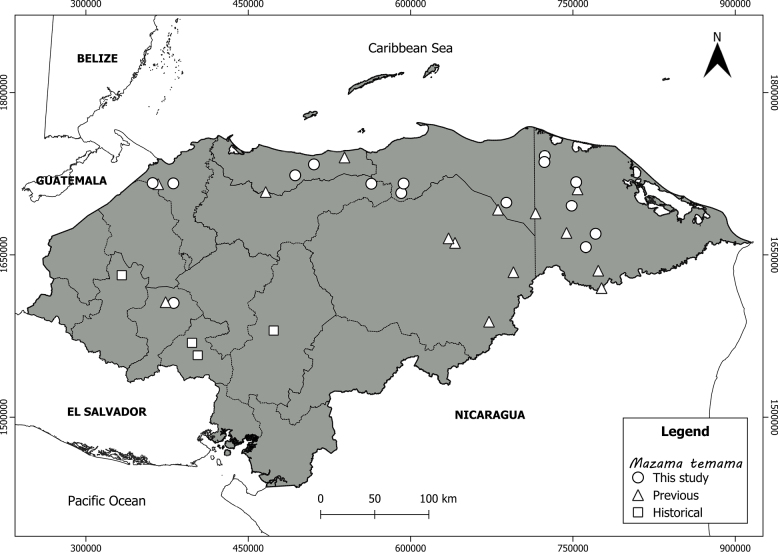
Distribution of *Mazama temama* in Honduras.

Atlántida, **Colón**, Cortés, Francisco Morazán, Gracias a Dios, Intibucá. La Paz, Lempira, Olancho, and Yoro (Fig. 100); 240–2090 m a.s.l.

###### Additional notes.

Individuals have been kept in captivity in Olancho (a juvenile male) from Sierra de Agalta National Park and Montaña Verde Wildlife Reserve (two males and three females), with uncertain departments (Lempira or Intibucá). Commonly known as tilopo, they are known as snapuka and sula pauni by the Miskitus in eastern Honduras (Jones 1965); and as sánah-paunia by Tawahkas, icha pawá by Pesh, and usari by Garifunas. It is a bush meat preferred by local people in eastern Honduras. Global conservation status is LC and DD in Honduras.

#### *Odocoileus* Rafinesque, 1832

##### 210. Odocoileus virginianus

Taxon classificationAnimaliaArtiodactylaCervidae

(Zimmermann, 1780)

31EDEEDA-2E4C-50AA-8193-C26FE6584B7A

###### Natural history.

It is noteworthy that four specimens from the Islas de la Bahía department (TCWC 14825-28) were collected on Roatán Island in 1965. On the Caribbean islands, caves are known to be used by pregnant females. Predators include jaguars, pumas, ocelots, and coyotes. The White-tailed Deer remains widespread in Honduras and continues to maintain stable populations in portions of the southern region of the country. Field observations from eastern Honduras, especially in southern La Moskitia, suggest the presence of individuals exhibiting an unusually reddish pelage coloration that differs from the more typical coloration observed elsewhere. This variation warrants further morphological and genetic investigation. Indigenous Miskitu hunters traditionally recognize what they believe to be three distinct types of deer occurring in Honduras, including the Red Brocket Deer and two putative forms of White-tailed Deer. Although these classifications have not been formally evaluated, they may reflect regional variation that deserves additional study.

###### Distribution.

**Figure 101.**
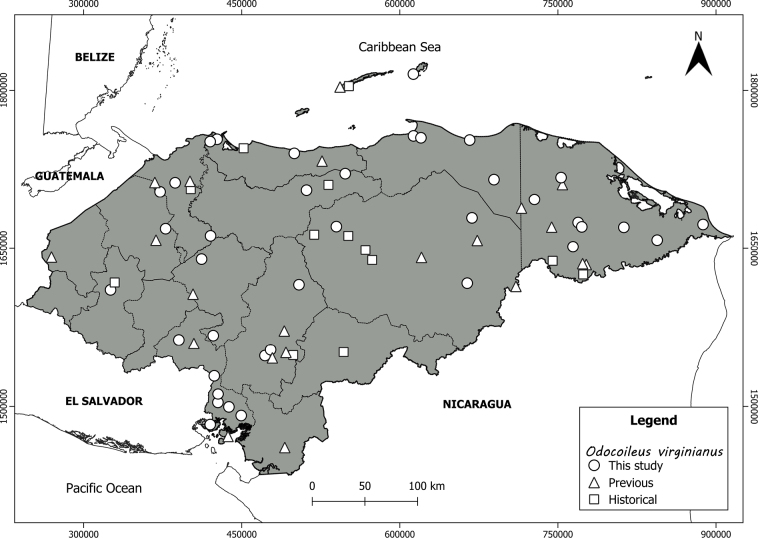
Distribution of *Odocoileus virginianus* in Honduras.

Atlántida, Choluteca, Comayagua, Copán, Colón, Cortés, Islas de la Bahía, El Paraíso, Francisco Morazán, Gracias a Dios, La Paz, Lempira, Olancho, Santa Bárbara, **Valle**, and Yoro (Fig. 101); 0–1840 m a.s.l.

###### Additional notes.

They are known as sula by the Miskitus in eastern Honduras (Jones 1965). In the past, White-tailed deer faced hunting pressure from residents of large cities in Honduras, including organized illegal hunting and sporting groups; therefore, local people were not primarily responsible for hunting in these areas. *O. virginianus* is among the principal sources of proteins in rural areas and among indigenous people in Honduras (Portillo et al. 2015). In the past, white-tailed deer fur trading centers were located in Trujillo, northern Honduras; Guayape; and Juticalpa, in the Olancho department. Similar to pacas and agoutis, white-tailed deer are also vulnerable to forest fires, particularly in pine-dominated forests and seasonally dry tropical ecosystems where fire events are relatively frequent. Following severe fires, whether of natural or human origin, burned carcasses have occasionally been documented, suggesting that wildfires can represent a significant source of mortality for local populations, especially when escape routes are limited or fire intensity is high. Global conservation status is LC and VU in Honduras.

####### Tayassuidae Palmer, 1897

#### *Dicotyles* G. Cuvier, 1817

##### 211. Dicotyles tajacu

Taxon classificationAnimaliaArtiodactylaTayassuidae

(Linnaeus, 1758)

6A8997AE-185E-5160-8E2E-412BFFC43A9C

###### Natural history.

Usually recorded in herds ranging from 3 to 30 and rarely solitary in the La Moskitia region. They like to feed on Arecaceae and Sapotacaeae fruits. Collared peccaries were recorded throughout the year; however, females accompanied by offspring were documented in August, October, November, and January, indicating successful reproduction within the area. See jaguar section to know about the reintroduction of peccaries in Jeanette Kawas National Park. This species had experienced a severe population decline in the park and surrounding areas for more than a decade, primarily due to habitat loss, urban expansion, and hunting pressure. Following conservation interventions and population recovery efforts, current evidence suggests that the species is re-establishing viable populations within the protected area.

Despite extensive survey efforts exceeding 3,000 camera-trap nights in southern Honduras, no recent confirmed records of the species have been obtained. While occasional anecdotal reports from local communities suggest that individuals may still persist at very low densities, these observations lack verification. The species is believed to be severely affected by illegal hunting, and there are currently no confirmed records from the dry forest ecosystems of Honduras.

In several protected areas of central Honduras, groups of 15–20 individuals have been observed, especially in montane forests above 1,500 m a.s.l. Nevertheless, habitat loss caused by deforestation and persistent illegal hunting continue to threaten these populations. Hunters commonly keep trophies or body parts of the species as mementos, reflecting the ongoing hunting pressure faced by the species throughout the region.

###### Distribution.

Atlántida, **Colón**, **Comayagua**, Cortés, Francisco Morazán, Gracias a Dios, **Intibucá**, **Lempira**, Ocotepeque, Olancho, Santa Bárbara, and Yoro (Fig. 84); 0–2140 m a.s.l.

###### Additional notes.

We follow Acosta et al. (2020) in using *Dicotyles* Cuvier, 1816, rather than *Pecari as the* genus. Historical records refer to both *Pecari tajacu* (Linnaeus, 1758) and *Tayassu tajacu* (Linnaeus, 1758). They are mainly threatened by habitat loss due to agriculture, such as intensive cultivation of pineapple, banana, and African (oil) palms on the Caribbean slope of Honduras. Hunting remains a threat as the species provides the only meat for many people. Wells (1857) noted that a popular dish among Englishmen on the Atlantic slope of Honduras was smoked pecari. Portillo and Vásquez (2009) reported evidence that collared peccaries were being hunted near Krautara (eastern Honduras in the Gracias a Dios department). They are known as bursa among the Miskitus in eastern Honduras (Jones 1965) and as quequeos or chanchos de monte in other regions of the country. They have not been recorded in Copán recently. However, there has been little effort to survey mammals in western Honduras. This record from Copán is from Woodman and Croft (2005). Peccaries, along with other large- and medium-sized mammal species (e.g., tapirs, deer), play a crucial role in maintaining ecological balance in the forests and ecosystems. Global conservation status is LC and VU in Honduras, both referring to *D. tajacu*.

#### *Tayassu* G. Fischer von Waldheim, 1814

##### 212. Tayassu pecari

Taxon classificationAnimaliaArtiodactylaTayassuidae

(Link, 1795)

E3B0E98D-FB68-5C81-AB8A-7CDA6FD59B4E

###### Natural history.

*Tayassu pecari* is a highly social ungulate restricted mainly to the extensive broadleaf forests of La Moskitia and a few adjacent areas. It is primarily diurnal, although it may become more nocturnal in response to hunting pressure (Portillo Reyes and Elvir 2016; Elvir Valle and Portillo Reyes 2019; Portillo-Reyes and Martínez 2024). This species forms large, cohesive herds that historically ranged from 40 to **>**; 150 individuals in Honduras, although smaller groups are more frequently detected by camera traps; it depends on continuous forest cover, high humidity, and access to water sources, often moving long distances across the landscape, which suggests wide home ranges and complex spatial dynamics (Portillo Reyes and Elvir 2016; Elvir Valle and Portillo Reyes 2019; Portillo-Reyes and Martínez 2024).

It is predominantly frugivorous but opportunistic, feeding on fruits, seeds, roots, and other plant material, as well as invertebrates and some vertebrates as coral snakes. This flexible diet makes it both an important seed predator and disperser, playing a key ecological role in shaping tropical forest composition and regeneration. It is also a major prey species for apex predators such as the *P. onca*, contributing significantly to trophic dynamics. Reproductive data in Honduras are scarce, but regional evidence indicates seasonal reproduction linked to resource availability, with females typically producing one or two offspring and occasionally being recorded with young in camera-trap studies (Portillo Reyes and Elvir 2016; Elvir Valle and Portillo Reyes 2019; Portillo-Reyes and Martínez 2024).

White-lipped peccaries exhibit strong social cohesion and coordinated defensive behavior when threatened. However, they are highly sensitive to human disturbance, particularly hunting, habitat loss, and fragmentation. In Honduras, historical accounts and recent studies indicate a marked decline in herd size and encounter rates, with increased hunting effort required over time and a drastic reduction in suitable habitat. Currently, the species persists mainly in remote forest areas, making it a key indicator of ecosystem integrity.

###### Distribution.

Colón, El Paraíso, Gracias a Dios, and Olancho (Fig. 12); 120–450 m a.s.l.

###### Additional notes.

Commonly known as jagüillas and they are known as wuaree by the Miskitus in eastern Honduras (Jones 1965). Thornton et al. (2020) discussed threats. Global conservation status is VU and CR in Honduras.

####### Balaenopteridae Gray, 1864

The waters of Honduras or other areas in the Western Caribbean Sea are not recognized as primary habitat for baleen whales. Sightings of free-swimming baleen whales are rare in the region, and most have been documented from strandings in shallow coastal habitats (Niño-Torres et al. 2015; Ramos et al. 2016).

#### *Balaenoptera* de Lacépède, 1804

##### 213. Balaenoptera physalus

Taxon classificationAnimaliaCetaceaBalaenopteridae

(Linnaeus, 1758)

6AC21565-A1C4-55DB-8686-F1AD8849CAC6

###### Natural history.

Fin whales are the second-largest mammal species in the world. They feed primarily on schools of fish, krill, and squid (Cooke 2018a). An adult stranded in Honduras, and the skeleton is currently on public display at the Tela Marine Aquarium in Tela.

###### Distribution.

Found in all the major oceans, from tropical to polar waters. Considered rare in the Caribbean Sea, only a few individuals have been recorded. In Honduras, a single record exists from an adult that was stranded in 2019 on the coast near Punta Sal, Department of Atlántida (Fig. 81).

###### Additional notes.

Global conservation status is VU and NE in Honduras.

#### *Megaptera* Gray, 1846

##### 214. Megaptera novaeangliae

Taxon classificationAnimaliaCetaceaBalaenopteridae

(Borowski, 1781)

0CD59AF0-C30A-5DEE-92CB-63B52371BE40

###### Natural history.

They are known for complex feeding behaviors, including bubble-net feeding and cooperative hunting with other humpback whales. Humpback whales are highly social animals and are known for their complex vocalizations, which they use to communicate with one another. They are also known for their acrobatic displays, including breaching, spy-hopping, and slapping the water with their tail flukes.

###### Distribution.

**Figure 102.**
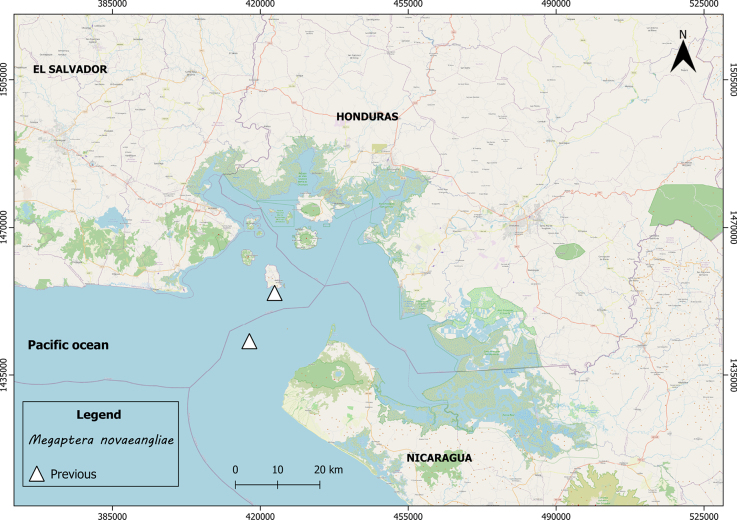
Distribution of *Megaptera novaeangliae* in Honduras. Note a record near the Pacific Coast of Nicaragua.

All deep-sea habitats in the Caribbean Sea and regularly visit the Pacific Ocean coast (Cooke 2018b; Fig. 102). The Caribbean waters of Honduras are not recognized as core habitat for this species, and sightings in this area are considered extralimital. A single humpback whale was documented in the coastal waters of Guatemala in 2016 and was later found stranded along the southern Belize coast (Ramos et al. 2016). Humpback whales are regular visitors to the Pacific Coast of Nicaragua, to the south of Honduras and El Salvador, and to the north of Honduras (de Weerdt and Ramos 2019; de Weerdt et al. 2020, 2021, 2022). There is a report of humpback whales in the Gulf of Fonseca: a group of 12 was sighted and recorded acoustically in 2004 during a seismic survey (Holst et al. 2017), and numerous reports of whales along the borders of Nicaragua and El Salvador (Rasmussen 2006). One of the whales in the group was record singing (Holst et al. 2017), which supports the idea that the Gulf of Fonseca, like the rest of the Pacific Coast of Mexico and Central America, is likely an important area for the endangered Central America Distinct Population Segment of the North Pacific population of humpback whales which use the area for breeding and calving (Bettridge et al. 2015).

###### Additional notes.

Global conservation status is LC and NE in Honduras.

####### Delphinidae Gray, 1821

#### *Globicephala* Lesson, 1828

##### 215. Globicephala macrorhynchus

Taxon classificationAnimaliaCetaceaDelphinidae

Gray, 1846

41B1E187-9F2C-5852-90F5-B4BD8A80298E

###### Natural history.

Short-finned pilot whales are known for their robust, stocky body shape, short, rounded dorsal fin, and a distinctive bulging forehead (or melon). In terms of their diet, short-finned pilot whales feed primarily on squid and small fish species, diving to depths of up to 1000 meters to hunt their prey. Mating and calving occur year-round, with females giving birth to a single calf every three to five years. They have a low reproductive rate, which makes them vulnerable to overexploitation by human activities, such as bycatch in fishing gear. Further research and conservation efforts are needed to protect this species and ensure its long-term survival.

###### Distribution.

This species inhabits deep-sea waters throughout the Atlantic Ocean and Caribbean Sea (Minton et al. 2018). In Honduras, opportunistic sightings of short-finned pilot whales have occurred in the department of Islas de la Bahía, primarily off the islands of Roatán and Utila (Fig. 46). Sparse sightings of these species have been recorded in neighboring Guatemala, Belize, and Mexico (Niño-Torres et al. 2015; Ramos et al. 2016; Ortiz-Wolford et al. 2022), possibly indicating Honduras is an area of importance for them.

###### Additional notes.

Global conservation status is LC and NE in Honduras.

#### *Peponocephala* Nishiwaki & Norris, 1966

##### 216. Peponocephala electra

Taxon classificationAnimaliaCetaceaDelphinidae

(Gray, 1846)

BAD49BA3-65CB-54AB-B005-F03E83EA7D5C

###### Natural history.

Mellon-headed whales primarily feed at night on small fish and squid from the mesopelagic zone. They are highly social and can travel in large groups of 100 to 500 individuals.

###### Distribution.

Mellon-headed whales are primarily found offshore in the pelagic zone of tropical and subtropical oceanic waters (Perryman and Danil 2018). They are not known to be migratory. In Honduras, only one record exists, a stranding of a female in the Bay of Omoa, Department of Cortés (Vallejo-Ham et al. 2026; Fig. 97).

###### Additional notes.

Global conservation status is NT and NE in Honduras.

#### *Pseudorca* Reinhardt, 1862

##### 217. Pseudorca crassidens

Taxon classificationAnimaliaCetaceaDelphinidae

(Owen, 1846)

9F92E546-90B1-5113-8F5D-21B0E22E53C4

###### Natural history.

False Killer whales feed on a variety of prey, including fish and squid, and are known to hunt in groups to maximize their chances of capturing food. False killer whales are social animals that form strong bonds within groups of up to 100 individuals.

###### Distribution.

False killer whales are widespread in tropical and subtropical regions and inhabit deep-sea oceanic habitats. In Honduras, only a few records exist, including one stranding, all from the department of Islas de la Bahía (Murcia et al. 2024; Fig. 47).

###### Additional notes.

Global conservation status is NT and NE in Honduras.

#### *Orcinus* Fitzinger, 1860

##### 218. Orcinus orca

Taxon classificationAnimaliaCetaceaDelphinidae

(Linnaeus, 1758)

7BB52264-5CD6-59AB-80A2-42ACB206F554

###### Natural history.

Killer whales are intelligent apex predators that exhibit complex social behaviors, including matrilineal family groups and unique vocal dialects within populations. They feed on a diverse range of prey, including fish, squid, seabirds, seals, sea lions, and even larger whale species. Their hunting tactics are highly coordinated. The diet of killer whales in Honduras is unknown; however, in 2023, a small group of three was observed attacking and consuming a sperm whale (*Physeter macrocephalus* Linnaeus, 1758) in Guanaja, Islas de la Bahía (Ochoa et al. 2025).

###### Distribution.

In Honduras, killer whales have been sighted in deep-sea habitats of the departments of Cortés and Islas de la Bahía (Bolaños‐Jiménez et al. 2014; Ochoa et al. 2025; Fig. 45); however, most sightings are from the islands of Roatan and Utila in the Islas de la Bahía department. Their regular occurrence in the area indicates that the killer whales that visit Honduras are part of a larger oceanic population with a distribution range across the Caribbean Sea Basin (Bolaños‐Jiménez et al. 2014). It is unknown whether they occur along the Pacific coast of Honduras, and given its relatively small size and topography, it is unlikely they inhabit this region; they are more likely found offshore (Bolaños‐Jiménez et al. 2014; Ramos et al. 2016).

###### Additional notes.

Global conservation status is DD and NE in Honduras.

#### *Steno* Gray, 1846

##### 219. Steno bredanensis

Taxon classificationAnimaliaCetaceaDelphinidae

(Lesson, 1828)

9977C378-5688-5F4E-924C-05BDAD86732D

###### Natural history.

Rough-toothed dolphins are typically found in offshore waters, beyond the continental shelf, and are known to form small, tight-knit social groups. They primarily feed on fish and squid and dive to depths of up to 300 meters in search of food.

###### Distribution.

All deep-sea habitats and nearshore areas in the Caribbean Sea, primarily around the Islas de la Bay Islands (Fig. 32), and possibly along the Pacific coast. This species is regularly observed around the Bay Islands and has historically been the most commonly observed dolphin in the area (Kuczaj and Yeater 2007). Kuczaj and Yeater (2007) surveyed several individuals around Utila with high levels of association, suggesting that at least some of the population may be residents of the area. Unlike many deep-sea areas frequented by rough-toothed dolphin, populations in Honduras are regularly observed in coastal, shallow-water habitats. This is similar to areas such as Guerrero, Mexico, where rough-toothed dolphins are sighted nearshore in the Bay of Petatlan (Ramos et al. 2021), indicating that this species enters shallow-water habitats. In contrast to most locations, these shallow water habitats are dominated by coral reefs and seagrass beds.

###### Additional notes.

Global conservation status is LC and NE in Honduras.

#### *Stenella* Gray, 1866

##### 220. Stenella longirostris

Taxon classificationAnimaliaMycosphaerellalesDelphinidae

(Gray, 1828)

AABB15B3-187E-5627-91FD-1D1CD5D6E7E1

###### Natural history.

Spinner dolphins are named for their distinctive behavior of leaping out of the water and spinning several times in mid-air before returning to the water, hence the name spinner. These dolphins are known for their dynamic, energetic personalities, characterized by long, slender rostra, streamlined, hydrodynamic physiques, and highly communicative, social behavior. Spinner dolphins are highly social animals that form complex social structures, consisting of stable groupings of individuals that engage in a variety of vocal and physical communication behaviors. These behaviors, ranging from synchronized swimming to body rubbing, are believed to play a critical role in maintaining social bonds within these groups. They feed on a variety of small fish, squid, and crustaceans. They are known to employ a variety of feeding strategies, including herding schools of fish, diving to depths of up to 100 meters to pursue prey, and foraging at night, when many of their preferred prey are most active.

###### Distribution.

Spinner dolphins are common in offshore waters and inshore waters, typically associated with banks or islands, in the Caribbean Sea and Pacific Ocean (Braulik and Reeves 2018). *Stenella longirostris* are common throughout the Caribbean Sea and more likely to occur in offshore habitats near oceanic islands or atolls (Barragán-Barrera et al. 2019). In Honduras (Fig. 33), spinner dolphins have been sighted opportunistically during tourist recreational diving and NGO surveys in the department of Islas de la Bahía. Sightings of this species in Honduras have generally involved small numbers of animals, possibly indicating spinner dolphins in this region use near-shore areas for daytime resting, as seen around several islands in the South Pacific (Oremus et al. 2007) and around the Hawaiian Islands (Tyne et al. 2015).

###### Additional notes.

Global conservation status is LC and NE in Honduras.

##### 221. Stenella attenuata

Taxon classificationAnimaliaMycosphaerellalesDelphinidae

(Gray, 1846)

B2EFF904-DAD2-5B48-9DCC-8520C6EA8E60

###### Natural history.

Pantropical spotted dolphins are recognized by their distinguishable dorsal spots, which become more pronounced as they age. These dolphins have a well-adapted physique for rapid swimming and agile maneuvers. The diet of the spotted dolphin primarily consists of small fish, squid, and some cephalopods. Their varied hunting tactics range from pursuing individual prey to coordinating with members of their pod to encircle and herd prey.

###### Distribution.

Pantropical spotted dolphins are widespread in warm oceanic waters around the world, from the Atlantic to the Pacific and the Indian Ocean. They frequent the waters surrounding Central America, particularly in deep-offshore environments (Mignucci-Giannoni et al. 2003). In Honduras (Fig. 44), they have been observed during recreational maritime activities and during environmental conservation studies, primarily in the department of Islas de la Bahía. Most of these observations report moderately sized pods, suggesting that these dolphins may use the coastal regions of Honduras as transient corridors during migration or for seasonal foraging, consistent with their behavior in regions such as the Eastern Tropical Pacific.

###### Additional notes.

Global conservation status is LC and NE in Honduras.

#### *Tursiops* Gervais, 1855

##### 222. Tursiops truncatus

Taxon classificationAnimaliaCetaceaDelphinidae

(Montagu, 1821)

78CA42FB-8B77-5F4D-A22A-C0799D38690E

###### Natural history.

Bottlenose dolphins are found in temperate and tropical waters worldwide, from the coasts to the open ocean (Wells and Scott 2018), and feed on a variety of prey, including fish, squid, and crustaceans. They employ a range of foraging strategies, including solo hunting, cooperative hunting with conspecifics, and scavenging. They are also known for using tools, such as seaweed to corral fish or their tails to create a bubble wall to trap prey.

###### Distribution.

All deep-sea habitats and coastal habitats, including lagoons and rivers in the Caribbean Sea waters of Honduras and the Pacific Ocean coastal waters. In the Caribbean Sea, bottlenose dolphins are regularly sighted along the shallow coast of Honduras and in deep-sea waters (Fig. 43), often opportunistically observed during recreational SCUBA diving trips in the Islas de la Bahía. Bottlenose dolphins are cosmopolitan and highly adaptable to a wide array of habitats (Wells et al. 2019); thus, in Honduras, they likely occur in rivers, lagoons, along the coast, in the deep sea, and around the Bay Islands. There have been no dedicated studies of this species in Honduras; however, in nearby Guatemala, Belize, and Mexico, populations are small and occur primarily in shallow coastal waters (Campbell et al. 2002; Dick and Hines 2010; Ramos et al. 2018; Ortiz-Wolford et al. 2022). In the Golfo de Fonseca on the Pacific coast of Honduras, bottlenose dolphins were once reported as common, but there have been few sightings in recent years (Barraza 2022).

###### Additional notes.

Global conservation status is LC and NE in Honduras.

####### Physeteridae Gray, 1821

#### *Physeter* Linnaeus, 1758

##### 223. Physeter macrocephalus

Taxon classificationAnimaliaCetaceaPhyseteridae

Linnaeus, 1758

CFED897E-FFC3-504B-B502-258D07C6941E

###### Natural history.

Sperm whales are found in oceans all over the world and feed primarily on squid, diving to great depths (~ 3 km) to hunt.

###### Distribution.

All deep-sea habitats in the Caribbean Sea (Whitehead et al. 2025). Only a handful of opportunistic sightings of sperm whales in the waters of Honduras in the Caribbean Sea have been recorded during tourist boat-based activities around the Bay Islands (Ochoa et al. 2025; Fig. 41). In Honduras, they are known to be preyed upon by killer whales (Ochoa et al. 2025). Their repeated occurrence for decades suggests they regularly visit the region or partially inhabit it. Numerous studies of sperm whale populations throughout the Caribbean Sea support the presence of sperm whales in the region (Gero et al. 2007). However, most studies of this species have been conducted around the islands in the Eastern and Southern Caribbean, which are recognized as critical habitat for the species (Gero et al. 2007, 2014; Gero and Whitehead 2016). Northwest of Honduras, sperm whales are regularly sighted in the offshore waters of Belize (Ramos et al. 2016) and Mexico (Niño-Torres et al. 2015). Nothing is known about their occurrence along the Pacific Coast of Honduras, but given the shallow habitats in the Golfo de Fonseca and the distance of this area from deep-water habitats, it is unlikely that sperm whales occur in the region.

###### Additional notes.

In the past, sperm whales were hunted for their valuable oil, which was used for lamp oil, lubricants, and other products; this hunting led to a significant decline in their population, but international protection measures have resulted in a recovery in recent decades. However, sperm whales are still considered vulnerable due to the ongoing threats from commercial whaling, ocean pollution, and ship strikes. Global conservation status is LC and NE in Honduras.

### Kogiidae Gill, 1871

#### *Kogia* Gray, 1846

##### 224. Kogia breviceps

Taxon classificationAnimaliaCetaceaKogiidae

(Blainville, 1838)

5ACA61F4-188E-5AC5-80E3-95D20ADA941D

###### Natural history.

Pygmy sperm whales are small cetaceans that are found in tropical and subtropical oceanic waters (Kiszka and Braulik 2020a). Much of what we know about pygmy sperm whales comes from standings. They are rarely seen at sea because they do not approach vessels and are found on the surface only briefly. Based on stomach content, they feed on cephalopods, fish, and crustaceans (Matsuda et al. 2023).

###### Distribution.

Pygmy sperm whales inhabit deep-sea habitats. They can be confused with the closely related dwarf sperm whale. In Honduras, there is only one report of this species from the island of Utila, Islas de la Bahía department (Brown et al. 2026; Fig. 93).

###### Additional notes.

Global conservation status is LC and NE in Honduras.

##### 225. Kogia sima

Taxon classificationAnimaliaCetaceaKogiidae

(Owen, 1866)

A0A4740B-FB41-5AF6-A8AF-5B161C50346F

###### Natural history.

Dwarf sperm whales occur throughout deep oceanic waters and insular and continental slope waters (Kiszka and Braulik 2020b). In the Bahamas, this species is frequently observed in both slope and deep-oceanic habitats and moves between them seasonally (Dunphy-Daly et al. 2008).

###### Distribution.

Dwarf sperm whales inhabit deep-sea habitats, and their cryptic behavior makes them extremely difficult to detect at sea. In Honduras, there are two reports of dwarf sperm whale strandings, one in the Islas de la Bahía department (Ramos et al. 2020; Fig. 45) and a record in the Gamma River in the Punta Izopo National Park in the Atlántida department (Marineros et al. 2013).

###### Additional notes.

Global conservation status is LC and NE in Honduras.

####### Ziphiidae Gray, 1865

#### *Mesoplodon* Gervais, 1850

##### 226. Mesoplodon europaeus

Taxon classificationAnimaliaCetaceaZiphiidae

(Gervais, 1855)

BB735CE8-5982-5B57-B9B8-91CFEA9D2F80

###### Natural history.

Gervais’ beaked whales are a species of beaked whale that are found in the deep waters of the Atlantic Ocean and Mediterranean Sea. These elusive whales feed on a diet composed primarily of squid. *M. europaeus* are typically found in offshore waters, beyond the continental shelf, where they are known to form small, tight-knit social groups. They are known to dive to great depths, often exceeding 1000 meters, in search of food and to avoid predators. However, based on the few scientific observations and strandings, they are believed to be relatively uncommon and to occur at low density within their range.

###### Distribution.

All deep-sea habitats in the Caribbean Sea (Pitman and Brownell Jr 2020). In Honduras, there are three records of *M. europaeus*, all from the island of Roatan in the department of Islas de la Bahía (Fig. 46; Marineros-Sánchez et al. 2020).

###### Additional notes.

Global conservation status is LC and NE in Honduras.

#### *Ziphius* G. Cuvier, 1823

##### 227. Ziphius cavirostris

Taxon classificationAnimaliaCetaceaZiphiidae

G. Cuvier, 1823

FE5A6674-C3A3-570C-9576-A9A5A2D3FAF3

###### Natural history.

Like many of the other beaked whales, little is known about the natural history of the goose-beaked whale. However, they are known to be deep-diving whales and currently hold the record for the longest and deepest dive (~ 9000 m) of any mammal (Schorr et al. 2014; Quick et al. 2020). Cephalopods constitute the majority of their diet (West et al. 2017).

###### Distribution.

The goose-beaked whale is found in deep-sea habitats of the tropics, including the Caribbean Sea and the Eastern Tropical Pacific Ocean. Two goose-beaked whale strandings have been documented in the Caribbean waters of Honduras, both from the department of Islas de la Bahía (Fig. 82). In other areas within their distribution range, this species is found along the edges of continental shelves and deep-sea trenches (Baird et al. 2013). The deep-sea territory of Honduras represents an ideal habitat for beaked whales, but nothing is known about the population in the region.

###### Additional notes.

Global conservation status is LC and NE in Honduras.

### Extinct and extirpated species

There are two recently extinct species that were formerly found in Honduras, the regionally distributed Caribbean monk seal (*Neomonachus tropicalis* (Gray, 1850)) and the endemic Little Swan Island hutia (*Geocapromys thoracatus* (F. W. True, 1889)) (Clough 1976; LeBoeuf et al. 1986). We also assume that Alston’s mouse opossum (*M. alstoni*) has been extirpated.

Kenyon (1977) considered the Caribbean or Tropical monk seal, *Monachus tropicalis (Gray, 1850)* (= *N. tropicalis*), as the first large mammal discovered by Columbus on his second voyage to the New World in 1494 (see Kerr 1811). According to Kenyon (1977), some people, such as Gilmore (1959) and Rice (1960, 1973), made significant efforts, including spending months in the Caribbean searching for this seal. It appears that the most recent record cited by Rice (1973) was made by C. B. Lewis in 1952, in which a small colony was found on Seranilla Bank in the Western Caribbean Sea. From 10–15 March 1973, the Seal Group of Mexico and the Caribbean patrolled the islands and atoll from Campeche to Quintana Roo in Mexico, British Honduras (Belize), Honduras, Nicaragua, and the central Caribbean to Jamaica, and they reported that the remotest habitats of the Caribbean monk seal have now been invaded by fishermen (Kenyon 1977), and this is considered the main reason that *N. tropicalis* has been extinct since 1950’s (Kenyon 1977). Documented locations in Honduras for the Caribbean monk seal include islands in the Gulf of Honduras, Isla Roatán, and Isla Barbareta (Timm et al. 1997). The aptly named Seal Keys (department of Colon) and Seal Cays off the coast of Belize (Miller and Miller 2025) likely derive their names from the former presence of *N. tropicalis* (Timm et al. 1997).

The Little Swan Island hutia was considered endemic to the Swan Islands (Gracias a Dios). Morgan (1989) noted that Lowe (1911) visited Little Swan Island for several days in 1908, observed more than a dozen individuals, and collected 15 in March and April 1912 for the Museum of Comparative Zoology. However, the last person to collect five specimens was Moyne (1938), who found the hutías so abundant that he obtained 12 live specimens in ~ 2 hours. By that time, goats had already been introduced into the area, and there were concerns that the destruction of the area was only a matter of time (Morgan 1989). Morgan (1989) also reported a final attempt by five Cayman Islanders to search for hutías on Little Swan Islands, who found none, a pattern that had persisted since the 1955 hurricane. Morgan (1989) confirmed that the species was extinct by the 1960’s due to the hurricane of 1955, and the introduction of cats and goats in the late 1950s. Finally, Morgan (1989) noted other survey efforts in the 1970’s that yielded no evidence of hutias. In 1997, one of us, FC, made a similar effort, but no evidence was found. See Turcios-Casco and López (2024) for a discussion regarding the recent situation of these important islands.

The case of the *M. alstoni* is also unique. The species has not been extirpated from its overall distribution (which currently still includes Nicaragua) but has not been reported in Honduras for > 80 years (Bautista et al. 2022), and according to Voss et al. (2021), the two juveniles collected by Tate (1933) in eastern Honduras represent the only record of the species for Honduras. However, Voss et al. (2021) considered the specimens to be ambiguously identified as *M. alstoni*. While it may be discovered in the future, we found no credible evidence of its presence. Marineros-Sánchez et al. (2016) reported the occurrence of an individual *M. alstoni*; however, we were unable to examine this specimen to confirm its identity because it is in a private collection. Given that Voss et al. (2021) noted that both *M. alstoni* and *M. nicaraguae* may be present, distinguishing between them from a photograph may be difficult. We recommend increased effort in Monte Carmelo, Olancho, the locality presented by Marineros-Sánchez et al. (2016), to confirm the current status of the specimens described by them.

### Expected species

We did not include the black-tailed mouse (*P. melanurus*) on the national list as its distributional limits remain unclear (Astúa et al. 2023). Additional undescribed species of the Alstoni Group seem likely to exist based on the evidence at hand, and we emphasize the need for renewed collecting (Voss et al. 2021). Therefore populations of *Marmosa* in eastern Honduras warrants a thorough review, and additional specimens are needed to confirm the species’ occurrence. We assume that both *M. alstoni* and *M. nicaraguae* are probable but require verification (see Bautista et al. 2022; Astúa et al. 2023).

*Reithrodontomys gracilis* was considered to occur here by the Mammal Diversity Database (2026), and Hoskins et al. (2018) reported a record identified as *Reithrodontomys gracilis* J. A. Allen & F. M. Chapman, 1897. However, there is insufficient evidence to conclude that it was distinguishable from conspecifics. We also consider *Habromys lophurus* (Osgood, 1904) as expected (it was also considered a possible occurrence in Honduras by Contreras-Medina et al. 2022). *Heteromys pictus* O. Thomas, 1893 (based on Rogers and González (2010) and on other *Heteromys* clades that may be present in Honduras). *Peromyscus gymnotis* O. Thomas, 1894, is also expected (Reid 2009). We do not consider *Peromyscus nudipes* (J. A. Allen, 1891) as suggested by Bradley et al. (2016) and Kilpatrick et al. (2021), for northern Central America, because the species is currently known only from Costa Rica and Panama (Bradley et al. 2016).

As noted previously, the following shrews are expected to occur in the country: *Cryptotis mam*, *C. montecristo*, and *C. tropicalis*, as they occur in neighboring countries and in similar habitats (see Woodman 2011); also see the comments of *C. copanensis* in the *Cryptotis* section. Based on Turcios-Casco et al. (2020a) and Mora et al. (2021), the following bats are expected to occur in Honduras: *Lampronycteris brachyotis* (Dobson, 1879), *Mesophylla macconnelli* Thomas, 1901 (see Silva et al. 2023), *Trinycteris nicefori* (Sanborn, 1949), *Thyroptera discifera* (Lichtenstein & Peters, 1854), and *Molossus coibensis* J. A. Allen, 1904.

The only carnivore expected is *Zalophus californianus* (Lesson, 1828) (Mammal Diversity Database 2026). Aurioles-Gamboa and Hernández-Camacho (2015) expected it to occur in the Pacific waters as extant and vagrant; the species has been confirmed in El Salvador (Ibarra Portillo et al. 2017) and Costa Rica (Mora and Ruedas 2023; Ramírez-Fernández et al. 2023).

There are 28 species of marine mammals, excluding the recently extinct Caribbean monk seal (*N. tropicalis*), listed as residents of Honduras in the IUCN Red List’s species accounts. These include two species of Mysticeti (baleen whales), 24 species of Odontoceti (toothed whales), one marine carnivore (sea lion), and one species of sirenian (manatee). Despite this apparent diversity, only 16 extant marine mammal species have been confirmed to occur in the territorial waters of Honduras in the Caribbean Sea and the Eastern Tropical Pacific, based on direct evidence such as osteological remains, stranding records, or photographic records. An additional species, the fin whale (*Balaenoptera physalus* (Linnaeus, 1758)), is not among the 28 resident species listed by the IUCN; however, an individual stranded in Tela in early 2019. This record likely represents a vagrant individual, as fin whale occurrences in tropical waters are rare. The sole record of a fin whale in neighboring Guatemala was from a stranded individual back in 1904 (Ortiz-Wolford et al. 2022). Even so, there is a clear discrepancy between the confirmed and presumed species of marine mammals in Honduras, given that hypothesized distributions have yet to be confirmed in many countries where they have been reported.

The 13 marine species listed as residents of Honduras, but for which we currently lack direct evidence are the following: *Zalophus californianus* (Lesson, 1828), *Balaenoptera edeni* Anderson, 1879, *Balaenoptera ricei* Rosel, Wilcox, Yamada and Mullin, 2021, *Stenella clymene* (Gray, 1850), *Stenella coeruleoalba* (Meyen, 1833), *Stenella frontalis* (G. Cuvier, 1829), *Delphinus delphis* Linnaeus, 1758, *Lagenodelphis hosei* Fraser, 1956, *Sotalia guianensis* (P. J. Van Beneden, 1864), *Feresa attenuata* Gray, 1974, *Grampus griseus* (G. Cuvier, 1812), *Mesoplodon densirostris* (Desmarest, 1817), and the *Mesoplodon peruvianus* Reyes, Mead and Van Waerebeek, 1991.

Many of those species are likely rare in Honduras if they are indeed present; however, some may be relatively common, and we do not yet have records due to the lack of baseline studies of marine mammals along most of Honduras’s Caribbean and Pacific coasts. Based on studies in neighboring countries, it is likely that as interest in the topic grows and additional studies are conducted, the gap between the two numbers will decrease as we confirm additional species. For example, Guiana dolphins (*S. guianensis*) were reported along the Miskitu coast of Nicaragua and in regions bordering Honduras, and were speculated to occur in riverine habitats (Edwards and Schnell 2001). Similarly, Clymene dolphins (*S. clymene*) have been sighted in the offshore waters of nearby Belize (Ramos et al. 2016) and are likely present in Honduras’ offshore waters. Pinnipeds—seals and sea lions—have not been identified in the Pacific Ocean coast of Honduras, but were recently documented in nearby Nicaragua (de Weerdt et al. 2024), suggesting they may occur in Honduras as well. In the Pacific, Bryde’s whale and Risso’s dolphin have stranded in El Salvador, less than 150 km from Honduras (Portillo et al. 2021). The Caribbean Sea coast of Honduras is substantially longer (~700 km) than its Pacific Ocean coast (~100 km) and encompasses a variety of coastal habitats, including rivers, lagoons, extensive coastline, and the Bay Islands (Harborne et al. 2001). To the best of our knowledge, there have not been any focused studies on marine mammals in the Pacific Ocean waters of Honduras in the Gulf of Fonseca. Its Pacific coast has three large bays: Bahía de la Unión, Bahía de Chismuyo, and Bahía de San Lorenzo. Bottlenose dolphins (*T. truncatus*) and striped dolphins (*S. coeruleoalba*) were sighted in 2021 in the El Salvador side of the Gulf of Fonseca during boat-based surveys. The Gulf is unlikely to be visited by many species of marine mammals and is likely inhabited by various dolphin species commonly found in coastal areas north and south of Honduras, in Mexico, El Salvador, and Nicaragua (de Weerdt et al. 2021). Most of the species identified in the Caribbean Sea territory of Honduras (except for Greater Caribbean manatees and short-finned pilot whales) inhabit the coastal and offshore waters of the Eastern Tropical Pacific Ocean. Of the species for which we have sightings and confirmation of occurrence, only the Greater Caribbean manatee (*T. m. manatus*) and rough-toothed dolphin (*S. bredanensis*) have been the focus of scientific studies in Honduras (e.g., Kuczaj and Yeater 2007; Gonzalez-Socoloske et al. 2009, 2011).

There are also other groups of mammals that have not been recorded for decades (between 20 and 80 years). These include rodents: *Neotoma ferruginea*, *N. chrysomelas*, *C. rostratus*, and shrews as *C. cavatorculus* and *C. magnimanus*. Many bat records fall into this category, e.g., *M. macrophyllum*, *L. obscura*, *G. daviesi*, *G. sylvestris*, *U. magnirostrum*, and *M. aztecus*. Other species recently documented from only a few records have no subsequent records, including the bats *D. youngii*, *M. cozumelae*, *V. spectrum*, *C. mexicana*, *C. gorgasi*, several marine mammals, and the northern olingo (*B. gabbii*).

### A conservation summary

Conservation is a recurring theme throughout this review, given ongoing challenges to mammal populations. The data presented here illustrate the stark realities of conservation in Honduras. The national red list includes 26.5% of Honduran mammals as being of conservation concern, and 9.8% are regionally threatened (WCS 2021). Of those species, six are endemic shrews: *S. mccarthyi*, *S. cruzi*, *C. celaque*, *C. cavatorculus*, *C. hondurensis*, *C. mccarthyi*, and *C. magnimanus*. Four other taxa are considered regional endemics also occurring in neighboring countries: *N. chrysomelas* (Nicaragua), *H. cherriei* (Nicaragua), and *A. inopinatus* (El Salvador, Nicaragua, and Costa Rica). In addition, the *Rheomys* spp. suggested by Martin et al. (2021) may represent a new taxon endemic to the country. Given Honduras’s geography, there may be more endemic species, particularly rodents and shrews (Bradley et al. 2016; Woodman 2019; López et al. 2024). The extinction risk for certain species may now be greater than the likelihood of their discovery. Additionally, the number of species discussed here, particularly the endemic mammals of Honduras, is likely underestimated. Considering Honduras’s geographical position and the rich diversity of its ecosystems, the actual number of mammal species may be higher than currently recognized.

## Conclusions

**Figure 103.**
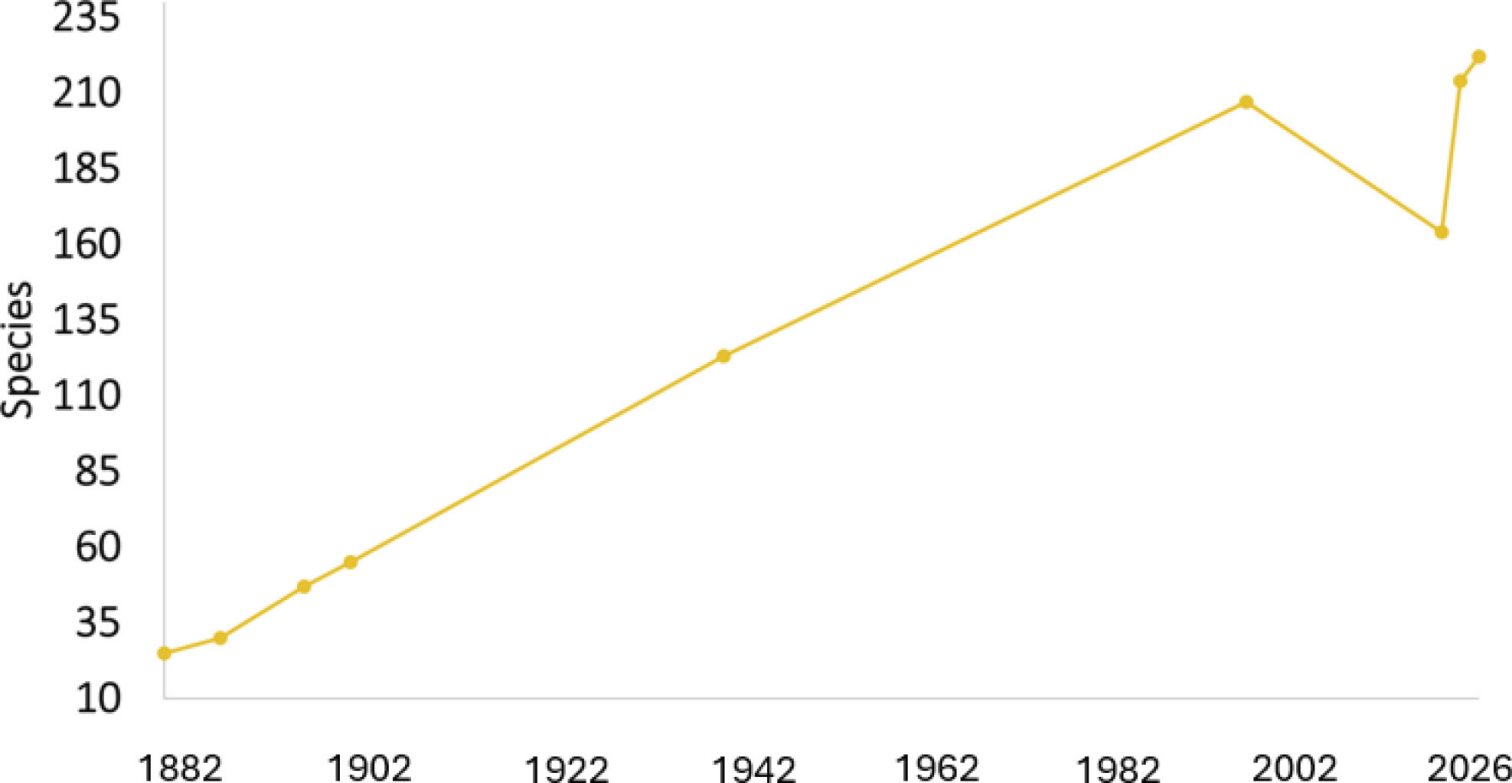
Historical increase in the number of mammal species recorded in Honduras from 1882 to 2026. The line graph illustrates the cumulative growth in documented species richness through time, reflecting the progression of taxonomic research, biological exploration, and improved survey efforts in the country. The number of recorded species increased steadily from fewer than 30 species in the late nineteenth century to > 200 species in recent decades. Although some fluctuations appear due to taxonomic revisions and changes in species recognition, the overall trend shows a marked increase in documented diversity, highlighting the continuous advancement of mammalogical research and biodiversity inventories in Honduras.

**Figure 104.**
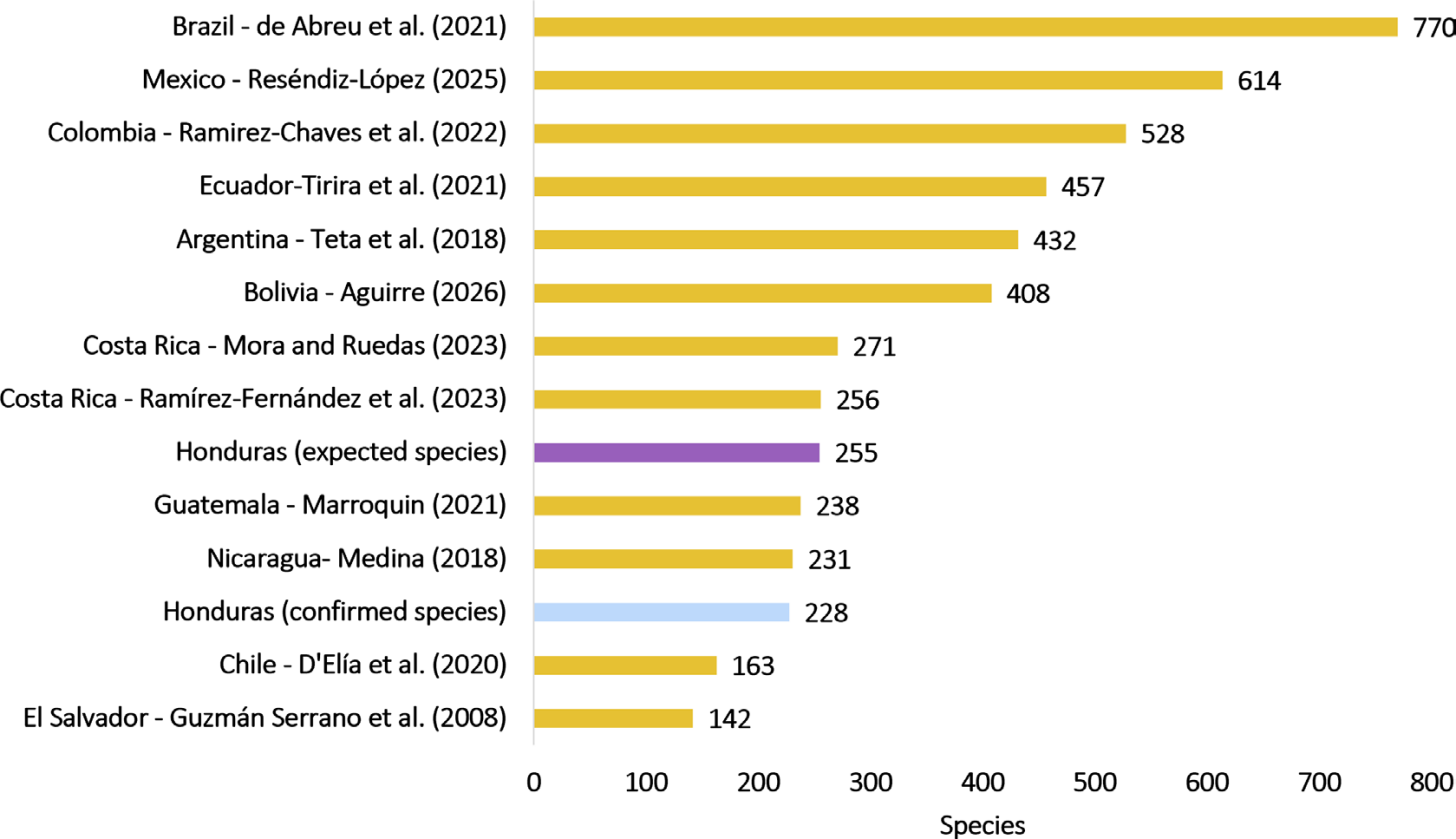
Comparison of mammalian species richness reported for selected Latin American countries based on published national inventories and checklists. Brazil exhibits the highest reported richness with 770 species (de Abreu et al. 2021), followed by Mexico with 614 species (Reséndiz-López 2025) and Colombia with 528 species (Ramírez-Chaves et al. 2021). Ecuador (456 species; Tirira et al. 2021), Argentina (432 species; Teta et al. 2018), and Bolivia (408 species; Aguirre et al. 2026) also show high mammalian diversity. Intermediate values are reported for Costa Rica, with 271 species according to Mora and Ruedas (2023) and 256 species according to Ramírez-Fernández et al. (2023). Guatemala (238 species; Marroquín 2021) and Nicaragua (231 species; Medina-Fitoria (2018)) present moderately lower richness values. Honduras is represented by both the number of confirmed species (227) and the estimated total richness (255), suggesting that additional species are likely to occur in the country but remain undocumented. Chile (163 species; D'Elía et al. 2020) and El Salvador (142 species; Guzmán Serrano et al. 2008) exhibit the lowest reported mammalian richness among the countries included in the comparison. For Honduras, the figure distinguishes between confirmed species (227) and the expected total richness (255), highlighting a potential gap between documented and estimated diversity.

Honduras is home to a diverse range of mammal species, accounting for 3.3% of the world’s total mammalian diversity, with 6,871 species as reported by the Mammal Diversity Database (2026). Currently, Honduras has fewer confirmed species than its neighboring countries: Nicaragua, Costa Rica, and Guatemala. There has been an historical shift regarding the final number of mammals in Honduras (Fig. 103), but is anticipated that the number of mammal species in Honduras will reach 255 (Fig. 104), which equates to approximately 0.002 species per square kilometer. If this projection is accurate, Honduras will closely follow Costa Rica, which has between 256 and 271 species (respectively, Mora and Ruedas 2023; Ramírez-Fernández et al. 2023).

Many species are currently facing various threats, such as habitat destruction, cattle grazing, intensive agriculture, and illegal trade. These issues require immediate attention and action. Unfortunately, the threats to mammals are on the rise, and even common species are facing local threats. In this historical and comprehensive review, we provide an overview of Honduran mammalian diversity, focusing on taxonomy, distribution, natural history, conservation status, and ecology, and building on the work of Marineros and Martínez (1998). This effort represents a significant milestone, offering the most up-to-date and thorough account of the country’s mammal fauna.

The comprehensive taxonomic account of Honduran mammals presented in this review documents 227 species, including three introduced rodents, representing 146 genera and 42 families. Considering the 28 probable species noted, we anticipate that the number of mammals may exceed the 255 documented here in the future. Given the paucity of records for many taxa, and with regions of Honduras that remain underexplored, there is an urgent need for further study.

## Additional information

### Artificial Intelligence (AI) use

The authors accept full responsibility for the content of the manuscript, including the disclosure of any use of AI.

No AI tools were used in the preparation of this manuscript.

### Author ORCIDs

M.A. Turcios-Casco https://orcid.org/0000-0002-3198-3834

F.E. Castañeda https://orcid.org/0009-0004-6639-955X

D.E. Wilson https://orcid.org/0000-0003-4228-5334

B. Miller https://orcid.org/0000-0001-5719-1942

D. Gonzalez-Socoloske https://orcid.org/0000-0002-4838-1426

E.A. Ramos https://orcid.org/0000-0003-4803-3170

T.W. King https://orcid.org/0000-0001-7974-8828

R.K. LaVal https://orcid.org/0000-0001-7726-9882

N. Ordóñez-Garza https://orcid.org/0000-0002-7732-7824

D. Ordoñez https://orcid.org/0009-0003-7234-0143

G. Graciolli https://orcid.org/0000-0002-1144-3486

D. Thornton https://orcid.org/0000-0002-2497-346X

M. Martínez https://orcid.org/0000-0003-3307-6848

N.M. Estrada-Andino https://orcid.org/0000-0002-0731-6521

H.D. Ávila-Palma https://orcid.org/0000-0002-7098-7635

### Data availability

All of the data that support the findings of this study are available in the main text.

## References

[B1] Aceituno F (2016) Cortejo y Apareamiento del *Puma yagouaroundi* en el Zoológico Rosy Walther, Honduras. Ceiba 53(2): 73–76. 10.5377/ceiba.v53i2.2369

[B2] Aceituno F, Trochez D, Nuñez C (2015) Recent record of the neotropical river otter (*Lontra longicaudis*) in the Choluteca river, Tegucigalpa, Honduras. IUCN Otter Specialist Group Bulletin 32(1): 25–29. https://www.iucnosgbull.org/Volume32/Aceituno_et_al_2015.html

[B3] Acosta LE (2015) Valoración económica del proceso de implementación de la Convención CITES en Honduras. Universidad Internacional de Andalucía, Baeza.

[B4] Acosta LE, Garbino GST, Gasparini GNM, Dutra PR (2020) Unraveling the nomenclatural puzzle of the collared and white-lipped peccaries (Mammalia, Cetartiodactyla, Tayassuidae). Zootaxa 4851(1): 60–80. 10.11646/zootaxa.4851.1.2 33056737

[B5] Acosta R (2011) Distributional data and taxonomic notes on the flea *Strepsylla* (Siphonaptera: Ctenophthalmidae: Neopsyllinae: Phalacropsyllini). Revista Mexicana de Biodiversidad 82(4): 1154–1162. 10.22201/ib.20078706e.2011.4.710

[B6] Aguirre LF, Wallace R, Bernal Hoverud N, Siles L, Aliaga - Rossel E, Salazar-Bravo J, Nogales-Ascarrunz P (2026) Lista Actualizada de los Mamíferos de Bolivia, 2026. Zenodo. 10.5281/zenodo.20398082

[B7] Allen H (1892) Description of a new genus of phyllostome bats. Proceedings of the United States National Museum 15(913): 441–442. 10.5479/si.00963801.15-913.441

[B8] Allen JA (1908) Mammals from Nicaragua. Bulletin of the American Museum of Natural History 24(34): 647–670.

[B9] Almendra AL, González-Cózatl FX, Engstrom MD, Rogers DS (2018) Evolutionary relationships and climatic niche evolution in the genus *Handleyomys* (Sigmodontinae: Oryzomyini). Molecular Phylogenetics and Evolution 128: 12–25. 10.1016/j.ympev.2018.06.018 29906608

[B10] Alston ER (1879–1882) Biologia Centrali-Americana. Mammalia. Taylor and Francis London, 220 pp.

[B11] Alvarado-Ortíz WJ, López CM, Soler-Orellana JA, Turcios-Casco MA (2024) Camera-trapping may help to understand the natural history of the deficiently known *Glaucomys volans* (Rodentia: Sciuridae) in Honduras. Biodiversity 25(4): 297–299. 10.1080/14888386.2024.2409454

[B12] Álvarez-Castañeda ST (2024) Mammals of North America - Vol. 1: Systematics and Taxonomy. Springer Nature Switzerland. 10.1007/978-3-031-41661-3

[B13] Amman BR, Bradley RD (2004) Molecular evolution in *Baiomys* (Rodentia: Sigmodontinae): evidence for a genetic subdivision in *B. musculus*. Journal of Mammalogy 85(1): 162–166. 10.1644/1545-1542(2004)085<0162:MEIBRS>2.0.CO;2

[B14] Andersen K (1906) XIII.—On the bats of the genera *Micronycteris* and *Glyphonycteris*. Journal of Natural History 18(103): 50–65. 10.1080/00222930608562579

[B15] Andino-Madrid AJ, Colindres JEM, Peréz-Consuegra SG, Matson JO (2020) A new species of long-tailed shrew of the genus *Sorex* (Eulipotyphla: Soricidae) from Sierra de Omoa, Honduras. Zootaxa 4809(1): 56–70. 10.11646/zootaxa.4809.1.3 33055948

[B16] Apanaskevich DA, Bermúdez SE (2013) Description of a new *Dermacentor* (Acari: Ixodidae) species, a parasite of wild mammals in Central America. Journal of Medical Entomology 50(6): 1190–1201. 10.1603/ME13121 24843922

[B17] Arbogast BS, Schumacher KI, Kerhoulas NJ, Bidlack AL, Cook AJ, Kenagy GJ (2017) Genetic data reveal a cryptic species of New World flying squirrel: *Glaucomys oregonensis*. Journal of Mammalogy 98(4): 1027–1041. 10.1093/jmammal/gyx055

[B18] Arellano E, Almendra AL, Martínez-Borrego D, González-Cózatl FX, Rogers DS (2023) Revisiting species delimitation within *Reithrodontomys sumichrasti* (Rodentia: Cricetidae) using molecular and ecological evidence. Therya 14(1): 161–179. https://therya.mastozoologiamexicana.com/index.php/THERYA/article/view/2236

[B19] Asher RJ, Helgen KM (2010) Nomenclature and placental mammal phylogeny. BMC Evolutionary Biology 10: 102. 10.1186/1471-2148-10-102 PMC2865478 20406454

[B20] Astúa D, Cherem JJ, Teta P (2023) Taxonomic checklist of living American marsupials. In: Cáceres NC, Dickman CR (Eds) American and Australasian Marsupials. An evolutionary biogeographical and ecological approach. Springer, Cham, 115–162. 10.1007/978-3-031-08419-5_31

[B21] Aurioles-Gamboa D, Hernández-Camacho J (2015) *Zalophus californianus*. The IUCN Red List of Threatened Species 2015: e.T41666A45230310. 10.2305/IUCN.UK.2015-4.RLTS.T41666A45230310.en [accessed 03 March 2026]

[B22] Ávila-Palma HD, Turcios-Casco MA, Bautista DJO, Martínez M, Ordoñez-Mazier DI (2019) First records of *Mimon cozumelae* Goldman, 1914 (Chiroptera, Phyllostomidae) in the Río Plátano Biosphere Reserve in northeastern Honduras. Check List 15(6): 1113–1118. 10.15560/15.6.1113

[B23] Ávila-Palma HD, Turcios-Casco MA, Velásquez A (2020) The Tomes’s Sword-nosed bat (*Lonchorhina aurita*) in Honduras, with new records in Sabanagrande, Francisco Morazán. Bat Research News 61(1): 1–6.

[B24] Baird AB, Marchán-Rivadeneira MR, Pérez SG, Baker RJ (2012) Morphological analysis and description of two new species of *Rhogeessa* (Chiroptera: Vespertilionidae) from the Neotropics. Occasional Papers Museum of Texas Tech University 307: 1–25. https://www.biodiversitylibrary.org/page/57756021

[B25] Baird AB, Braun JK, Mares MA, Morales JC, Patton JC, Tran CQ, Bickham JW (2015) Molecular systematic revision of tree bats (Lasiurini): doubling the native mammals of the Hawaiian Islands. Journal of Mammalogy 96(6): 1255–1274. 10.1093/jmammal/gyv135

[B26] Baird AB, Braun JK, Engstrom MD, Holbert AC, Huerta MG, Lim BK, Maers MA, Patton JC, Bickham JW (2017) Nuclear and mtDNA phylogenetic analyses clarify the evolutionary history of two species of native Hawaiian bats and the taxonomy of Lasiurini (Mammalia: Chiroptera). PLoS ONE 12(10): e0186085. 10.1371/journal.pone.0186085 PMC5636129 29020097

[B27] Baird AB, Braun JK, Engstrom MD, Lim BK, Mares MA, Patton JC, Bickham JW (2021) On the utility of taxonomy to reflect biodiversity: the example of Lasiurini (Chiroptera: Vespertilionidae). Therya 12(2): 283–289. 10.12933/therya-21-1117

[B28] Baird RW, Webster DL, Aschettino JM, Schorr GS, McSweeney DJ (2013) Odontocete cetaceans around the main Hawaiian Islands: Habitat use and relative abundance from small-boat sighting surveys. Aquatic Mammals 39(3): 253–269. 10.1578/AM.39.3.2013.253

[B29] Barragán-Barrera DC, do Amaral KB, Chávez-Carreño PA, Farías-Curtidor N, Lancheros-Neva R, Botero-Acosta N, Bueno P, Moreno IB, Bolaños-Jímenez J, Bouveret L, Castelblanco-Martínez DN, Luksenburg JA, Mellinger J, Mesa-Gutiérrez R, de Montgolfier B, Ramos EA, Ridoux V, Palacios DM (2019) Ecological niche modeling of three species of *Stenella* dolphins in the Caribbean Basin, with application to the Seaflower Biosphere Reserve. Frontiers in Marine Science 6(10): 1–17. 10.3389/fmars.2019.00010

[B30] Barraza JE (2022) La ausencia del delfín *Tursiops truncatus* (Montagu, 1821) en el Golfo de Fonseca, El Salvador. Revista Minerva 5: 85–90. 10.5377/revminerva.v5i1.15697

[B31] Barthe M, Rancilhac L, Arteaga MC, Feijó A, Tilak MK, Justy F, Loughry WJ, McDonough CM, de Thoisy B, Catzeflis F, Billet G, Hautier L, Benoit N, Delsuc F (2025) Exon capture museomics deciphers the nine-banded armadillo species complex and identifies a new species endemic to the Guiana Shield. Systematic Biology 74(2): 177–197. 10.1093/sysbio/syae027 PMC11958936 38907999

[B32] Basantes M, Tinoco N, Velazco PM, Hofmann MJ, Rodríguez-Posada ME, Camacho MA (2020) Systematics and taxonomy of *Tonatia saurophila* Koopman and Williams, 1951 (Chiroptera, Phyllostomidae). ZooKeys 915: 59–86. 10.3897/zookeys.915.46995 PMC7052022 32148423

[B33] Bautista LFA, Wilson DE, Turcios-Casco MA (2022) Annotations on the taxonomy of the opossums (Didelphimorphia: Didelphidae) of Honduras. Mammalia 86(6): 641–643. 10.1515/mammalia-2022-0027

[B34] Berkenhout J (1769) Outlines of the natural history of Great Britain and Ireland (Vol. 1). P. Elmsly, London, 233 pp.

[B35] Bermingham E, Coates A, Cruz DG, Emmons L, Foster RB, Leschen R, Seutin G, Thorn S, Wcislo W, Werfel B (1998) Geology and terrestrial flora and fauna of Cayos Cochinos, Honduras. Revista de Biologia Tropical 46: 15–37.

[B36] Bettridge SOM, Baker CS, Barlow J, Clapham P, Ford MJ, Gouveia D, Mattila DK, Pace RM, Rosel PE, Silber GK (2015) Status review of the humpback whale (*Megaptera novaeangliae*) under the Endangered Species Act. US Department of Commerce NOAA Technical Memorandum NMFS-SWFSC-540. https://repository.library.noaa.gov/view/noaa/4883

[B37] Bolaños CS, Montalvo V, Fuller TK, Carrillo E (2015) Records of black jaguars at Parque Nacional Barbilla, Costa Rica. CATnews 62: 38–39.

[B38] Bolaños‐Jiménez J, Mignucci‐Giannoni AA, Blumenthal J, Bogomolni A, Casas JJ, Henríquez A, Bessega MI, Khan J, Landrau-Giovannetti N, Rinaldi C, Rinaldi R, Rodríguez-Ferrer G, Sutty L, Ward N, Luksenburg JA (2014) Distribution, feeding habits and morphology of killer whales *Orcinus orca* in the Caribbean Sea. Mammal Review 44(3–4): 177–189. 10.1111/mam.12021

[B39] Boubli JP, Rylands AB, Farias IP, Alfaro ME, Alfaro JL (2012) *Cebus* phylogenetic relationships: a preliminary reassessment of the diversity of the untufted capuchin monkeys. American Journal of Primatology 74(4): 381–393. 10.1002/ajp.21998 22311697

[B40] Bradley RD, Ensink JK (1987) Karyotypes of five cricetid rodents of Honduras. Texas Journal of Science 39: 171–175.

[B41] Bradley RD, Henson DD, Durish ND (2008) Re-evaluation of the geographic distribution and phylogeography of the *Sigmodon hispidus* complex based on mitochondrial DNA sequences. Southwestern Naturalist 53(3): 301–310. 10.1894/MRD-03.1 PMC2897183 20613884

[B42] Bradley RD, Nuñez-Tabares M, Soniat TJ, Kerr S, Raymond RW, Ordóñez-Garza N (2016) Molecular systematics and phylogeography of *Peromyscus nudipes* (Cricetidae: Neotominae). Special Publications Museum of Texas Tech University 65: 201–213.

[B43] Braulik GT, Reeves R (2018) *Stenella longirostris* (errata version published in 2019). The IUCN Red List of Threatened Species 2018: e.T20733A156927622. 10.2305/IUCN.UK.2018-2.RLTS.T20733A156927622.en

[B44] Brown T, Hill J, Nielsen SV (2020) Attempted ingestion of a bat *Saccopteryx leptura* results in the death of a Green Vine snake *Oxybelis fulgidus* (Squamata; Colubridae) on Utila Island, Honduras. Sauria Berlin 42(1): 66–72.

[B45] Brown T, Herrera JM, Izaguirre AP, Narvaez SF, Garcia A (2026) Stranding of a juvenile Pygmy Sperm whale (*Kogia breviceps*) in Utila Island, Honduras, with evidence of plastic ingestion in the Western Caribbean. Latin American Journal of Aquatic Mammals 21: 1.

[B46] Brown TW (2018) *Lampropeltis abnorma* (Colubridae; Squamata): documenting a regurgitated prey item from Cusuco National Park, Honduras. Captive and Field Herpetology 2(1): 25–28.

[B47] Buckley JS (1983) The feeding behavior, social behavior, and ecology of the white-faced monkey, *Cebus capucinus*, at Trujillo, Northern Honduras, Central America. PhD Thesis, The University of Texas, Austin.

[B48] Burgin CJ, Colella JP, Kahn PL, Upham NS (2018) How many species of mammals are there? Journal of Mammalogy 99(1): 1–14. 10.1093/jmammal/gyx147

[B49] Calahorra-Oliart A, Ospina-Garcés SM, León-Paniagua L (2021) Cryptic species in *Glossophaga soricina* (Chiroptera: Phyllostomidae): do morphological data support molecular evidence? Journal of Mammalogy 102(1): 54–68. 10.1093/jmammal/gyaa116

[B50] Calderón-Acevedo CA, Bagley JC, Muchhala N (2022) Genome-wide ultraconserved elements resolve phylogenetic relationships and biogeographic history among Neotropical leaf-nosed bats in the genus *Anoura* (Phyllostomidae). Molecular Phylogenetics and Evolution 167: 107356. 10.1016/j.ympev.2021.107356 34774763

[B51] Camacho MA, Burneo SF, Cadar D, Horváth B, Tóth GE, Murienne J (2026) Geographic structuring and species limits in *Phylloderma stenops* (Chiroptera: Phyllostomidae): Revalidation of *P. septentrionalis* in Central America. Vertebrate Zoology 76: 339–359. 10.3897/vz.76.e166549

[B52] Campbell GS, Bilgre BA, Defran RH (2002) Bottlenose dolphins (*Tursiops truncatus*) in Turneffe Atoll, Belize: occurrence, site fidelity, group size, and abundance. Aquatic Mammals 28(2): 170–180.

[B53] Carroll DS, Bradley RD (2005) Systematics of the genus *Sigmodon* DNA sequences from Beta-Fibrinogen and Cytochrome B. The Southwestern Naturalist 50(3): 342–349. 10.1894/0038-4909(2005)050[0342:SOTGSD]2.0.CO;2

[B54] Carter CH, Genoways HH (1978) *Liomys salvini*. Mammalian Species (84): 1–5. 10.2307/3503987

[B55] Castañeda FE, Pereira SC (2013) Behaviour of two male jaguars scavenging on a marine dolphin in Honduras. CATnews 58: 11–12.

[B56] Castañeda FE, Pereira S, Herrera L, Estrada N (2013) Diversidad de mamíferos terrestres en fincas de cacao de Olancho, Honduras. Reporte PANTHERA/HELVETAS-HONDURAS/APROSACAO. Tegucigalpa 22 pp.

[B57] Castañeda FE, McCann NP, Ávila-Palma HD (2023) Confirmed presence of a puma in the binational Merendón Mountain Range. Cat News 77: 33–35.

[B58] Castelblanco-Martínez DN, Nourisson C, Quintana-Rizzo E, Padilla-Saldivar J, Schmitter-Soto JJ (2012) Potential effects of human pressure and habitat fragmentation on population viability of the Antillean manatee *Trichechus manatus*: a predictive model. Endangered Species Research 18(2): 129–145. 10.3354/esr00439

[B59] Castelblanco‐Martínez DN, Padilla‐Saldívar J, Hernández‐Arana HA, Slone DH, Reid JP, Morales‐Vela B (2013) Movement patterns of Antillean manatees in Chetumal Bay (Mexico) and coastal Belize: A challenge for regional conservation. Marine Mammal Science 29(2): 166–182. 10.1111/j.1748-7692.2012.00602.x

[B60] Cláudio VC, Novaes RL, Gardner AL, Nogueira MR, Wilson DE, Maldonado JE, Oliveira JÁ, Moratelli R (2023) Taxonomic re-evaluation of New World *Eptesicus* and *Histiotus* (Chiroptera: Vespertilionidae), with the description of a new genus. Zoologia (Curitiba) 40: e22029. 10.1590/S1984-4689.v40.e22029

[B61] Clough GC (1976) Current status of two endangered Caribbean rodents. Biological Conservation 10(1): 43–47. 10.1016/0006-3207(76)90023-9

[B62] Cobo‐Simón I, Méndez‐Cea B, Portillo H, Elvir F, Vega H, Gallego FJ, Fontecha G (2018) Testing the effectiveness of conservation management within biosphere reserves: the case of the Mexican deer mouse (*Peromyscus mexicanus*) as a bioindicator. Integrative Zoology 14(5): 422–434. 10.1111/1749-4877.12371 30585414

[B63] Contreras-Medina R, Santiago-Alvarado M, Espinosa D, Rivas G, Luna-Vega I (2022) Distributional patterns and conservation of the genus *Habromys* (Rodentia: Cricetidae) in Mesoamerica. Studies on Neotropical Fauna and Environment 175–191. 10.1080/01650521.2022.2085071

[B64] Cooke JG (2018a) *Megaptera novaeangliae*. The IUCN Red List of Threatened Species 2018: e.T13006A50362794. 10.2305/IUCN.UK.2018-2.RLTS.T13006A50362794.en

[B65] Cooke JG (2018b) *Balaenoptera physalus*. The IUCN Red List of Threatened Species 2018: e.T2478A50349982. 10.2305/IUCN.UK.2018-2.RLTS.T2478A50349982.en

[B66] Corley MS, Ordóñez-Garza N, Rogers DS, Bradley RD (2011) Molecular evidence for paraphyly in *Nyctomys sumichrasti*: support for a new genus of vesper mice? Occasional Paper, Museum Texas Tech University 306: 1–12. https://www.depts.ttu.edu/nsrl/publications/downloads/OP306.pdf

[B67] Da Silva Fonseca B, Soto-Centeno JA, Simmons NB, Ditchfield AD, Leite YLR (2024) A species complex in the Iconic Frog-Eating bat *Trachops cirrhosus* (Chiroptera, Phyllostomidae) with high variation in the heart of the neotropics. American Museum Novitates 4021: 1–27. 10.1206/4021.1

[B68] Davis WB (1968) Review of the genus *Uroderma* (Chiroptera). Journal of Mammalogy 49(4): 676–698. 10.2307/1378728

[B69] Davis WB, Carter DC (1964) A new species of fruit-eating bat (genus *Artibeus*) from Central America. Proceedings of the Biological Society of Washington 77: 119–122. 10.5281/zenodo.17260729

[B70] de Abreu EF, Pavan SE, Tsuchiya MT, Wilson DE, Percequillo AR, Maldonado JE (2020) Museomics of tree squirrels: a dense taxon sampling of mitogenomes reveals hidden diversity, phenotypic convergence, and the need of a taxonomic overhaul. BMC Evolutionary Biology 20(77): 1–25. 10.1186/s12862-020-01639-y PMC7320592 32590930

[B71] de Abreu EF, Casali D, Costa-Araújo R, Garbino GST, Libardi GS, Loretto D, Loss AC, Marmontel M, Moras LM, Nascimento MC, Oliveira ML, Pavan E, Tirelli FP (2021) Lista de Mamíferos do Brasil (2021–2). Zenodo.

[B72] de Abreu EF, Pavan SE, Tsuchiya MT, McLean BS, Wilson DE, Percequillo AR, Maldonado JE (2022) Old specimens for old branches: Assessing effects of sample age in resolving a rapid Neotropical radiation of squirrels. Molecular Phylogenetics and Evolution 175: 107576. 10.1016/j.ympev.2022.107576 35809853

[B73] D’Elía G, Canto J, Ossa G, Verde-Arregoitia LD, Bostelmann E, Iriarte A, Amador L, Quiroga-Carmona M, Hurtado N, Cadenillas R, Valdez L (2020) Lista actualizada de los mamíferos vivientes de Chile. Boletín del Museo Nacional de Historia Natural 69(2): 67–98. 10.54830/bmnhn.v69.n2.2020.6

[B74] de Ferran V, Vieira Figueiró H, Silveira Trinca C, Hernández-Romero PC, Lorenzana GP, Gutiérrez-Rodríguez C, Koepfli K-P, Eizirik E (2024) Genome-wide data support recognition of an additional species of Neotropical river otter (Mammalia, Mustelidae, Lutrinae). Journal of Mammalogy 105(3): 534–542. 10.1093/jmammal/gyae009

[B75] de Weerdt J, Ramos EA (2019) Feeding behavior of humpback whales (*Megaptera novaeangliae*) on the Pacific coast of Nicaragua. Marine Mammal Science 36(1): 285–292. 10.1111/mms.12613

[B76] de Weerdt J, Ramos EA, Cheeseman T (2020) Northernmost records of Southern Hemisphere humpback whales (*Megaptera novaeangliae*) migrating from the Antarctic Peninsula to Nicaragua. Marine Mammal Science 36(3): 1015–1021. 10.1111/mms.12677

[B77] de Weerdt J, Ramos EA, Pouplard E, Kochzius M, Clapham P (2021) Cetacean strandings along the Pacific coast of Nicaragua from 2014 to 2019. Marine Biodiversity Records 14(13): 3–9. 10.1186/s41200-021-00209-5

[B78] de Weerdt J, Calambokidis J, Pouplard E, Pouey-Santalou V, Patulny C, Vanshoenwinkel B, Kochzius M, Clapham P (2022) Abundance, distribution and behaviour of humpback whales (*Megaptera novaeangliae*) along the Pacific coast of Nicaragua, Central America. Marine and Freshwater Research 73(8): 1041–1055. 10.1071/MF21326

[B79] de Weerdt J, Ramos EA, Acosta T (2024) First record of pinnipeds along the Pacific Coast of Nicaragua. Aquatic Mammals 50(1): 1–7. 10.1578/AM.50.1.2024.1

[B80] Deutsch CJ, Castelblanco-Martínez DN, Groom R, Cleguer C (2022) Movement behavior of manatees and dugongs: I. Environmental challenges drive diversity in migratory patterns and other large-scale patterns. In: Marsh H (Eds) Ethology and behavioral ecology of Sirenia. Ethology and behavioral ecology of marine mammals. Springer, Cham, 155–174. 10.1007/978-3-030-90742-6_5

[B81] Dick CW (2013) Review of the bat flies of Honduras, Central America (Diptera: Streblidae). Journal of Parasitology Research 1: 437696. 10.1155/2013/437696 PMC3619636 23634295

[B82] Dick DM, Hines EM (2010) Using distance sampling techniques to estimate bottlenose dolphin (*Tursiops truncatus*) abundance at Turneffe Atoll, Belize. Marine Mammal Science 27(3): 606–621. 10.1111/j.1748-7692.2010.00435.x

[B83] Dickey DR (1928) Five new mammals of the genus *Peromyscus* from El Salvador. Proceedings of the Biological Society of Washington 41: 1–6.

[B84] Dinets V (2017) Long-term cave roosting in the spectral bat (*Vampyrum spectrum*). Mammalia 81(5) 529–530. 10.1515/mammalia-2016-0038

[B85] Dinets V, Polechla PJ (2005) First documentation of melanism in the jaguar (*Panthera onca*) from northern Mexico. Cat News 42: 18.

[B86] Divoll TJ, Buck DG (2013) Noteworthy field observations of cave roosting bats in Honduras. Mastozoología Neotropical 20(1): 149–151.

[B87] Dunphy‐Daly MM, Heithaus MR, Claridge DE (2008) Temporal variation in dwarf sperm whale (*Kogia sima*) habitat use and group size off Great Abaco Island, Bahamas. Marine Mammal Science 24: 171–182. 10.1111/j.1748-7692.2007.00183.x

[B88] Edwards HH, Schnell GD (2001) Status and ecology of *Sotalia fluviatilis* in the Cayos Miskito Reserve, Nicaragua. Marine Mammal Science 17(3): 445–472. 10.1111/j.1748-7692.2001.tb00998.x

[B89] Elvir Valle FA, Portillo Reyes HO (2019) The ungulates of Honduras. In: Gallina-Tessaro S (Ed.) Ecology and conservation of tropical ungulates in Latin America. Springer, Cham, 161–178. 10.1007/978-3-030-28868-6_8

[B90] Elvir Valle FA, Marineros Sanchez LE (2021) Consumo tradicional de zorrillo grande (*Conepatus leuconotus*) en el Occidente de Honduras. Scientia hondurensis 4(2): 26–29.

[B91] Elvir Valle FA, Núñez-Figueroa LJ, Díaz-Sánchez MA (2021) Primer registro de albinismo en la guatusa (*Dasyprocta punctata* Gray, 1942) para Honduras. Revista Mexicana de Mastozoología (Nueva Época) 11(2): 17–21. 10.22201/ie.20074484e.2021.11.2.341

[B92] Espinal M, Mora JM (2012) Ampliación en la distribución de cinco especies de murciélagos en Honduras basada en detección por medios acústicos. Ceiba 53(1): 30–37. 10.5377/ceiba.v53i2.2433

[B93] Espinal M, Mora JM (2016) Noteworthy record of *Eptesicus brasiliensis* (Vespertilionidae) in Honduras. Ceiba 53(2): 77–80. 10.5377/ceiba.v53i2.2433

[B94] Espinal M, Mora JM, Ruedas LA, López LI, Marineros L (2016) A case of albinism in the Central American spider monkey, *Ateles geoffroyi*, in Honduras. Mastozoología Neotropical 23(1): 63–69.

[B95] Espinal MR, López LI, Mora JM (2020) Consumption event of the Pallas’s mastiff bat (*Molossus molossus*) by the Central America bark scorpion (*Centruroides exilimanus*) in Honduras. Therya Notes 1: 110–114. 10.12933/therya_notes-20-24

[B96] Espinal MR, Miller BW, Mora JM (2021) First record of *Centronycteris centralis* (Chiroptera: Emballonuridae) from Honduras. Notas sobre Mamíferos Sudamericanos 3: e21.11.1. 10.31687/saremNMS.21.11.1

[B97] Espinoza Montero MA (2015) Persistencia de cadáveres de aves y murciélagos situados en pastizales y bosques del campus Zamorano, Honduras. Graduate Dissertation, Escuela Agrícola Panamericana, Zamorano, 26 pp.

[B98] Esquivel DA, Pereira MJR, Stuhler JD, Rossoni DM, Velazco PM, Bianchi FM (2022) Multiples lines of evidence unveil cryptic diversity in the *Lophostoma brasiliense* (Chiroptera: Phyllostomidae) complex. Systematics and Biodiversity 20(1): 1–21. 10.1080/14772000.2022.2110172

[B99] Estrada N (2006a) Ecología del danto (*Tapirus bairdii*) en el Parque Nacional Sierra de Agalta, Olancho, Honduras. Proyecto USAID-MIRA.

[B100] Estrada N (2006b) Humans attacked by a Baird’s Tapir (*Tapirus bairdii*) in the Sierra de Agalta National Park, Olancho, Honduras. Tapir Conservation 15(20): 13–14. http://www.tapirconservation.org/index.php/tc/article/view/91

[B101] Ferran V, Vieira Figueiró H, Silveira Trinca C, Hernández-Romero PC, Lorenzana GP, Gutiérrez-Rodríguez C, Koepfli K-P, Eizirik E (2024) Genome-wide data support recognition of an additional species of Neotropical river otter (Mammalia, Mustelidae, Lutrinae), Journal of Mammalogy 105(3): 534–542 10.1093/jmammal/gyae009

[B102] Gamero I (1978) Mamíferos de mi tierra. Volumen 2. Banco Central de Honduras, 234 pp.

[B103] Gardner AL (1983) *Proechimys semispinosus* (Rodentia: Echimyidae): distribution, type locality, and taxonomic history. Proceedings of the Biological Society of Washington 96(1): 134–144.

[B104] Gardner AL [Ed.] (2007) Mammals of South America, Vol. 1: Marsupials, Xenarthrans, Shrews, and Bats. University of Chicago Press, 690 pp. 10.7208/chicago/9780226282428.001.0001

[B105] Gardner AL, Ramírez-Pulido J (2020) Type localities of Mexican land mammals, with comments on taxonomy and nomenclature. Special Publications, Museum of Texas Tech University 73: 1–134. https://www.depts.ttu.edu/nsrl/publications/downloads/SP73.pdf

[B106] GBIF.org (2023) GBIF Occurrence Download 10.15468/dl.tbbsw8 [Accessed 28 September 2023]

[B107] Genoways HH (1973) Systematics and evolutionary relationships of spiny pocket mice, genus *Liomys*. Special Publication, Museum, Texas Tech University 5: 1–368. 10.5962/bhl.title.156484

[B108] Gero S, Whitehead H (2016) Critical decline of the Eastern Caribbean sperm whale population. PLoS ONE 11(10): e0162019. 10.1371/journal.pone.0162019 PMC5051958 27706153

[B109] Gero S, Gordon J, Carlson C, Evans P, Whitehead H (2007) Population estimate and inter-island movement of sperm whales, *Physeter macrocephalus*, in the Eastern Caribbean Sea. Journal of Cetacean Research and Management 9(2): 143–150. 10.47536/jcrm.v9i2.682

[B110] Gero S, Milligan M, Rinaldi C, Francis P, Gordon J, Carlson C, Steffen A, Tyack P, Evans PGH, Whitehead H (2014) Behavior and social structure of the sperm whales of Dominica, West Indies. Marine Mammal Science 30(3): 905–922. 10.1111/mms.12086

[B111] Gilmore RM (1959) Is the West Indian monk seal extinct? Sea Frontiers 5: 225–236.

[B112] Gilroy DL, Jones S, Vulinec K, Thompson P, Hoskins H, Creedy T, Jacque M, Green S, Lonsdale G, Phipps C, Burdekin O, Brown T (2017) Operation Wallacea Cusuco National Park, Honduras 2016 and 2017: End of season report. Operation Wallacea, 81 pp.

[B113] Girón-Juárez DM, Contreras-Díaz RG, Medellín RA, Rojas-Soto O, Díaz-Martínez S, Nori J, Martín G, Osorio-Olvera L (2025) Beyond climate: Anthropogenic pressures reshape vampire bat distributions across the neotropics. Biological Conservation 311: 111443. 10.1016/j.biocon.2025.111443

[B114] Godman FD (1915) Biologia Centrali-Americana. Zoology, Botany, and Archaeology. Introductory Volume. Taylor and Francis, London, 149 pp.

[B115] Gómez-Corea W, Marineros L, España FG (2019) Primer registro de miasis en *Ototylomys phyllotis* (Marriam, 1901) (Cricetidae/Rodentia) por *Cuterebra* sp. (Oestridae/Díptera) en Honduras. Scientia hondurensis 2(2): 15–19.

[B116] Gómez-Corea W, Mejía-Quintanilla D, Hernández J, Vallejo-Ham AE, Flores R, Figueroa-Grande A (2020) *Diclidurus albus* Wied-Neuwied, 1819 (Mammalia, Chiroptera): geographic distribution in Honduras, with new records inferred from acoustic evidence and morphology. Caribbean Journal of Science 50(2): 265–274. 10.18475/cjos.v50i2.a10

[B117] Gómez-Corea W, Hernández J, Antúnez-Fonseca C, Mejía-Quintanilla D, Vega H (2021b) Leucism and updated geographic distribution of *Molossus nigricans* Miller, 1902 (Chiroptera: Molossidae) in Honduras. Mammalia 85(1): 64–70. 10.1515/mammalia-2019-0153

[B118] Gómez-Corea W, España FG, Mejía-Quintanilla D, Alvarez MR (2022) Bat fly (Diptera: Streblidae) and common vampire bat (Chiroptera: Phyllostomidae) association in Honduras: prevalence, mean intensity, infracommunities and influence of the biological characteristics of the host. Zoologia (Curitiba) 39: e21018. 10.1590/s1984-4689.v39.e21018

[B119] Gómez-Corea WN, España FG, Mejía-Quintanilla DJ, Figueroa-Grande AN (2021a) Bats (Mammalia, Chiroptera) from Yuscarán in Eastern Honduras: Conservation and acoustic characterization for the insectivorous species. Papéis Avulsos de Zoologia 61: e20216179. 10.11606/1807-0205/2021.61.79

[B120] Gómez-Corea WN, Marineros L, Vega HL, López-Aguilar TP (2023) Geographic distribution of pocket gophers (Rodentia, Geomyidae) of Honduras. Check List 19(4): 581–588. 10.15560/19.4.581

[B121] Gonthier DJ, Castañeda FE (2013) Large- and medium-sized mammal survey using camera traps in the Sikre River in the Río Plátano Biosphere Reserve, Honduras. Tropical Conservation Science 6(4): 584–591. 10.1177/194008291300600409

[B122] Gonzalez-Socoloske D, Snarr KA (2010) An incident of swimming in a large river by a mantled howling monkey (*Alouatta palliata*) on the North Coast of Honduras. Neotropical Primates 17(1): 28–31. 10.1896/044.017.0102

[B123] Gonzalez-Socoloske D, Olivera-Gomez LD, Ford RE (2009) Detection of free ranging West Indian manatees *Trichechus manatus* using side-scan sonar. Endangered Species Research 8: 249–257. 10.3354/esr00232

[B124] Gonzalez-Socoloske D, Taylor CR, Rendon Thompson OR (2011) Distribution and conservation status of the Antillean manatee (*Trichechus manatus manatus*) in Honduras. Latin American Journal of Aquatic Mammals 9(2): 123–131. 10.5597/lajam00176

[B125] Goodwin GG (1932) Two new mammals from Guatemala. American Museum Novitates 528: 1–2.

[B126] Goodwin GG (1937) Two new harvest mice from Honduras. American Museum novitates 921: 1–2.

[B127] Goodwin GG (1941) A new *Peromyscus* from western Honduras. American Museum novitates 1121.

[B128] Goodwin GG (1942a) Mammals of Honduras. Bulletin of the American Museum of Natural History 79: 107–195.

[B129] Goodwin GG (1942b) *Eptesicus albigularis* (Peters) from Honduras. American Museum Novitates 1199: 1–4.

[B130] Graciolli G, Ávila-Palma HD, Ordoñez-Trejo EJ, Soler-Orellana JA, Ordoñez-Mazier DI, Martínez M, LaVal R, Turcios-Casco MA (2021) Additions of host associations and new records of bat ectoparasites of the families Spinturnicidae, Nycteribiidae and Streblidae from Honduras. Check List 17(2): 459–469. 10.15560/17.2.459

[B131] Griffin DR (1986) Listening in the Dark Cornell University Press, Ithaca, 415 pp.

[B132] Groves C, Grubb P (2011) Ungulate taxonomy. The John Hopkins University Press, Baltimore, 317 pp. 10.56021/9781421400938

[B133] Guimarães LR, D’Andretta MAV (1956) Sinopse dos Nycteribiidae (Diptera) do Novo Mundo. Arquivos de Zoologia do Estado de São Paulo Brasil 10: 1–184. 10.11606/issn.2176-7793.1956101-184

[B134] Gutiérrez EE, Garbino G (2018) Species delimitation based on diagnosis and monophyly, and its importance for advancing mammalian taxonomy. Zoological Research 39(5): 301–308. 10.24272/j.issn.2095-8137.2018.037 PMC6102684 29551763

[B135] Guzmán Serrano V, Henríquez S, Rodríguez M, Lara K (2008) Mamíferos de El Salvador: Fichas técnicas. Fundación Zoológica de El Salvador (FUNZEL) – Universidad de El Salvador (UES /Escuela de Biología), San Salvador, 299 pp.

[B136] Hall ER (1981) The Mammals of North America, Vol. 1 (2^nd^ edn.). John Wiley and Sons.

[B137] Harborne AR, Afzal DC, Andrews MJ (2001) Honduras: Caribbean coast. Marine Pollution Bulletin 42(12) 1221–1235. 10.1016/S0025-326X(01)00239-9 11827108

[B138] Harmsen BJ, Williams S, Abarca M, Álvarez Calderón FS, Araya‐Gamboa D, Avila HD, Barrantes‐Núñez M, Bravata‐de la Cruz Y, Broadfield J, Cabral‐Araújo V, Calderón AP, Castañeda F, Corrales‐Gutiérrez D, do Couto‐Peret Dias B, Dantas Marinho PH, Devlin AL, Escobar‐Anleu BI, Espinoza‐Muñoz D, Esser HJ, Foster RJ, Fragoso CE, Friedeberg D, Herrera LA, Hidalgo‐Mihart MG, Hoogesteijn R, Jansen PA, Jędrzejewski W, Jesus‐de la Cruz A, de Jesus Rodrigues D, Jordan CA, Juárez‐Lopez R, Kadosoe V, Kelly MJ, King TW, Lugarini C, Venticinque EM, da Matta Nigro G, McPhail DKT, Meyer N, Morales‐Rivas A, Nepomuceno V, Nipko RB, Noronha J, de Oliveira‐Vasquez M, Ouboter P, Paemelaere EAD, Payán E, dos Santos TP, Salom‐Pérez R, Sanchez EE, Santos‐Simioni S, Schmidt K, Stasiukyans D, Tortato FR, Urbina‐Ruiz E, Urquhart GR, Wong W, Robinson H (2024) Estimating species distribution from camera trap by-catch data, using jaguarundi (*Herpailurus yagouaroundi*) as an example. Diversity and Distributions 30: e13831. 10.1111/ddi.13831

[B139] Henson DD, Bradley RD (2009) Molecular systematics of the genus *Sigmodon*: results from mitochondrial and nuclear gene sequences. Canadian Journal of Zoology 87(3): 211–220. 10.1139/Z09-005 PMC2855974 20407590

[B140] Hernández-Canchola G, León-Paniagua L (2021) About the specific status of *Baiomys musculus* and *B. brunneus*. Therya 12(2): 291–301. 10.12933/therya-21-1150

[B141] Hernández-Canchola G, León-Paniagua L, Esselstyn JA (2022) Paraphyletic relationships revealed by mitochondrial DNA in the *Peromyscus mexicanus* species group (Rodentia: Cricetidae). Revista Mexicana de Biodiversidad 93: e933811. 10.22201/ib.20078706e.2022.93.3811

[B142] Herrera-Martínez A, Alberico MS, Daza JD (2026) The genera *Hoplomys* and *Proechimys* (Rodentia: Echimyidae) synonymized. Mastozoología Neotropical 33(1): e01244. 10.31687/saremMN.26.33.02.01.e1244

[B143] Holbrook LT (2013) The taxonomy of ungulates in the 21^st^ century. Journal of Mammalogy 94: 1304–1306.

[B144] Holst M, Smultea MA, Koski WR, Sayegh AJ, Pavan G, Beland J, Goldstein HH (2017) Cetacean sightings and acoustic detections during a seismic survey off Nicaragua and Costa Rica, November-December 2004. Revista de Biología Tropical 65(2): 599–611. 10.15517/rbt.v65i2.25477

[B145] Hooper ET (1952) A systematic review of the harvest mice (genus *Reithrodontomys*) of Latin America. Miscellaneous Publications Museum of Zoology 77: 1–255.

[B146] Hoskins HMJ, Burdekin OJ, Dicks K, Slater KY, McCann NP, Jocque M, Castañeda F, Reid N (2018) Non-volant mammal inventory of Cusuco National Park, north-west Honduras: reporting the presence of jaguar, *Panthera onca* (Linnaeus, 1758), and demonstrating the effects of zonal protection on mammalian abundance. Check List 14(5): 877–891. 10.15560/14.5.877

[B147] Ibarra Portillo RE, Herrera Serrano NO, Paniagua Palacios WC, Pérez Chávez JE (2017) Presencia de leones marinos (Carnivora, Pinnipedia, Otariidae) en El Salvador. Revista Comunicaciones Científicas y Técnológicas 2(1): 82–90.

[B148] IUCN (2026) The IUCN Red List of Threatened Species. Version 2025-2. https://www.iucnredlist.org

[B149] Jones JK (1965) Some Miskito Indian names for mammals. Journal of Mammalogy 46(2): 353–354. 10.2307/1377878

[B150] Jung K, Molinari J, Kalko EKV (2014) Driving factors for the evolution of species-specific echolocation call design in New World free-tailed bats (Molossidae). PLoS ONE 9: e85279. 10.1371/journal.pone.0085279 PMC3891751 24454833

[B151] Kenyon KW (1977) Caribbean monk seal extinct. Journal of Mammalogy 58(1): 97–98. 10.2307/1379738

[B152] Kerr R (1811) A general history and collection of voyages and travels. Vol. 3. Edinburgh.

[B153] Kilpatrick CW, Pradhan N, Norris RW (2021) A re-examination of the molecular systematics and phylogeography of taxa of the *Peromyscus aztecus* species group, with comments on the distribution of *P. winkelmanni*. Therya 12(2): 331–346. 10.12933/therya-21-1115

[B154] Kiszka J, Braulik GT (2020a) *Kogia breviceps*. The IUCN Red List of Threatened Species 2020: e.T11047A50358334. 10.2305/IUCN.UK.2020-2.RLTS.T11047A50358334.en

[B155] Kiszka J, Braulik GT (2020b) *Kogia sima*. The IUCN Red List of Threatened Species 2020: e.T11048A50359330. 10.2305/IUCN.UK.2020-2.RLTS.T11048A50359330.en

[B156] Klein EH (1979) Review of the status of manatee (*Trichechus manatus*) in Honduras, Central America. Ceiba 23(1): 21–28.

[B157] Kuczaj Il SA, Yeater DB (2007) Observations of rough-toothed dolphins (*Steno bredanensis*) off the coast of Utila, Honduras. Journal of the Marine Biological Association of the United Kingdom 87(1): 141–148. 10.1017/S0025315407054999

[B158] Kunz TH, Kurta A (1988) Capture methods and holding devices. In: Kunz TH (Ed.) Ecological and behavioral methods for the study of bats. Smithsonian Institution Press, Washington D. C.

[B159] La Gaceta (2017) Decreto No. 021-2017. Periodo de registro de Modalidades de Manejo y/o Aprovechamiento de Vida Silvestre (Flora y Fauna). https://faolex.fao.org/docs/pdf/hon176179.pdf

[B160] Lawrence B (1933) A new pocket gopher of the genus *Orthogeomys*. Proceedings of the New England Zoological Club 8: 65–67.

[B161] LeBoeuf BJ, Kenyon KW, Villa‐Ramirez B (1986) The Caribbean monk seal is extinct. Marine Mammal Science 2(1): 70–72. 10.1111/j.1748-7692.1986.tb00028.x

[B162] Leivers SJ, Meierhofer MB, Pierce BL, Evans JW, Morrison ML (2019) External temperature and distance from nearest entrance influence microclimates of cave and culvert‐roosting tri‐colored bats (*Perimyotis subflavus*). Ecology and Evolution 9(24): 14042–14052. 10.1002/ece3.5841 PMC6953682 31938502

[B163] López CM, Turcios-Casco MA (2025) An overview of caudal autotomy in rodents, with new records for Cricetidae and Heteromyidae. Notas de Mamíferos Sudamericanos 7: 1. 10.31687/SaremNMS25.1095

[B164] López CM, Salas WFC, Andino NE (2022) Especies potenciales para jardines alimenticios de dantos (*Tapirus bairdii*) en cautiverio. Portal de la Ciencia 1(17): 20–31. 10.5377/pc.v1i17.16075

[B165] López CM, Turcios-Casco MA, Ordóñez-Garza N, van Den Berghe E, del Valle Alvarez M (2024) A taxonomic odyssey: A commented checklist of genus *Peromyscus* (Cricetidae) in Honduras. ZooKeys 1218: 315–332. 10.3897/zookeys.1218.126535 PMC11607588 39619476

[B166] López CM, Ordóñez-Garza N, Ordóñez-Mazier D, Bautista L, Soler-Orellana J, Tur-cios-Casco MA (2026) Distribution, defensive behavior, and natural history notes of the genus *Heteromys* (Rodentia) in Honduras. Mammalia aequatorialis 8(1): 63–77.

[B167] López Gallego Z, Medina-van Berkum P, Martin TE (2021) Opportunistic predation events of bats entangled in mist nets by margay *Leopardus wiedii* (Schinz, 1821) in northwest Honduras: recommendations to avoid preventable casualties. Journal of Bat Research and Conservation 14(1): 33–37. 10.14709/BarbJ.14.1.2021.04

[B168] López-Wilchis R, Guevara-Chumacero LM, Pérez NÁ, Juste J, Ibáñez C, Sosa IBD (2012) Taxonomic status assessment of the Mexican populations of funnel-eared bats, genus *Natalus* (Chiroptera: Natalidae). Acta Chiropterologica 14(2): 305–316. 10.3161/150811012X661639

[B169] Lowe PR (1911) A naturalist on desert islands. Witherby and Co., London. 10.5962/bhl.title.85337

[B170] Mammal Diversity Database (2026) Mammal Diversity Database. 10.5281/zenodo.4139722 [Accessed 28 April 2026]

[B171] Marineros L, Elvir F (2019) Dos nuevos registros de *Marmosa mexicana* (Marsupialia: Didelphidae) en las Islas de la Bahía. Scientia hondurensis 2(2): 25–28.

[B172] Marineros L, Martínez F (1998) Guía de campo de los mamíferos de Honduras. Instituto Nacional de Ambiente y Desarrollo (INADES). 374 pp

[B173] Marineros L, Portillo Reyes HO (2015) Actualización de la distribución y notas sobre el perezoso de tres garras *Bradypus variegatus castaneiceps* (Pilosa: Bradypodidae) en Honduras. Revista Mexicana de Mastozoología (Nueva Época) 5: 1–14. 10.22201/ie.20074484e.2015.5.1.202

[B174] Marineros L, Mondragón ME, Matamoros W (2013) First report of *Kogia sima* (Cetacea: Kogiidae) on the Caribbean coast of Honduras. UNED Cuadernos de Investigación UNED 5(2): 227–230. 10.22458/urj.v5i2.295

[B175] Marineros LE, Vega H, Elvir F, Espinal M, Hernandez J, Calix E (2015) Notas y nuevos sitios de encuentro de *Philander oposum fuscogriseus* (Marsupialia: Didelphidae) en Honduras. Bioma 33: 39–48.

[B176] Marineros LE, Vega H, Adams J, Mejia MM (2016) Notas y nuevos sitios de encuentro de *Caluromys derbianus* (Marsupialia: Didelphidae) en Honduras. Revista Biodiversidad Neotropical 6(1): 77–84. 10.18636/bioneotropical.v6i1.337

[B177] Marineros LE, O’Reilly C, Rodríguez O (2019) Nuevo registro de *Metachirus nudicaudatus* (Desmarest, 1817) en Honduras. Scientia hondurensis 2(2): 38–41.

[B178] Marineros-Sánchez L, Portillo-Reyes H, Vega H, Hernández J (2018) Registros y Distribución Potencial del Puercoespín (*Coendou mexicanus*), (Rodentia: Erethizontidae) en Honduras. Revista Mexicana De Mastozoología (Nueva Época) 8(2): 93–102. 10.22201/ie.20074484e.2018.1.2.265

[B179] Marineros-Sánchez L, Amaya J, Bolton T, Bolton E, Brady-Ramos MG, Flores-Moncada CC, Talbott J (2020) Primeros registros de *Mesoplodon europaeus* (Cetacea: Ziphiidae) para Honduras. Scientia hondurensis 3(1): 44–48.

[B180] Marineros-Sánchez LE, Downing R, Espinal M (2016) Nuevo registro de *Marmosa alstoni* (Allen, 1900) (Didelphiomorpda, Didelphidae) para Honduras. Scientia hondurensis 1(1): 1–6.

[B181] Marroquin C (2021) Listado Actualizado de los Mamíferos de Guatemala. FLAAR Mesoamérica.

[B182] Marroquín C [Ed.] (2021) Listado actualizado de los mamíferos de Guatemala. FLAAR Mesoamérica, 58 pp.

[B183] Marsh CJ, Sica YV, Burgin CJ, Dorman WA, Anderson RC, del Toro Mijares I, Vigneron JG, Barve V, Dombrowik VL, Duong M, Guralnick R, Hart JA, Maypole JK, McCall K, Ranipeta A, Schuerkmann A, Torselli MA, Lacher T, Mittermeier RA, Rylands AB, Sechrest W, Wilson DE, Abba AM, Aguirre LF, Arroyo‐Cabrales J, Astúa D, Baker AM, Braulik G, Braun JK, Brito J, Busher PE, Burneo SF, Camacho MA, Cavallini P, de Almeida Chiquito E, Cook JA, Cserkész T, Csorba G, Cuéllar Soto E, da Cunha Tavares V, Davenport TRB, Deméré T, Denys C, Dickman CR, Eldridge MDB, Fernandez‐Duque E, Francis CM, Frankham G, Franklin WL, Freitas T, Friend JA, Gadsby EL, Garbino GST, Gaubert P, Giannini N, Giarla T, Gilchrist JS, Gongora J, Goodman SM, Gursky‐Doyen S, Hackländer K, Hafner MS, Hawkins M, Helgen KM, Heritage S, Hinckley A, Hintsche S, Holden M, Holekamp KE, Honeycutt RL, Huffman BA, Humle T, Hutterer R, Ibáñez Ulargui C, Jackson SM, Janecka J, Janecka M, Jenkins P, Juškaitis R, Juste J, Kays R, Kilpatrick CW, Kingston T, Koprowski JL, Kryštufek B, Lavery T, Lee TE, Leite YLR, Novaes RLM, Lim BK, Lissovsky A, López‐Antoñanzas R, López‐Baucells A, MacLeod CD, Maisels FG, Mares MA, Marsh H, Mattioli S, Meijaard E, Monadjem A, Morton FB, Musser G, Nadler T, Norris RW, Ojeda A, Ordóñez‐Garza N, Pardiñas UFJ, Patterson BD, Pavan A, Pennay M, Pereira C, Prado J, Queiroz HL, Richardson M, Riley EP, Rossiter SJ, Rubenstein DI, Ruelas D, Salazar‐Bravo J, Schai‐Braun S, Schank CJ, Schwitzer C, Sheeran LK, Shekelle M, Shenbrot G, Soisook P, Solari S, Southgate R, Superina M, Taber AB, Talebi M, Taylor P, Vu Dinh T, Ting N, Tirira DG, Tsang S, Turvey ST, Valdez R, Van Cakenberghe V, Veron G, Wallis J, Wells R, Whittaker D, Williamson EA, Wittemyer G, Woinarski J, Zinner D, Upham NS, Jetz W (2022) Expert range maps of global mammal distributions harmonised to three taxonomic authorities. Journal of Biogeography 49: 979–992. 10.1111/jbi.14330 PMC9060555 35506011

[B184] Martin T, Jones S, Hoskins H, McCann N, Batke S, Kelly D, Kolby J, Downing R, Zelaya S, Green S, Lonsdale G, Brown T, Waters S, Rodriguez-Vasquez F, McCravy K, D’Souza M, Crace D, Nunez-Mino J, Haelewaters D, Berkum P, Phipps C, Bakker R, Castaneda F, Reid N, Jocque M (2021) A review of the ecological value of Cusuco National Park an urgent call for conservation action in a highly threatened Mesoamerican cloud forest. Journal of Mesoamerican Biology 1: 6–50.

[B185] Martínez M, Velásquez A, Pacheco-Amador S, Cabrera N, Acosta I, Turcios-Casco MA (2020) El perezoso de dos dedos (*Choloepus hoffmanni*) en Honduras: distribución, historia natural y conservación. Notas sobre Mamíferos Sudamericanos 2(1): 2–9. 10.31687/saremNMS.20.0.25

[B186] Martínez M, King TW, Thornton D, Miranda F, Turcios-Casco MA (2022) An offspring from the northernmost population of *Myrmecophaga tridactyla* (Xenarthra: Myrmecophagidae). Edentata 23: 23–28. 10.2305/IUCN.CH.2o22.Edentata-23-1-6.en

[B187] Martínez M, Cabrera N, Guzmán E, Casco G, Sánchez Ávila-Palma HD, King T, Thornton D, Castañeda F (2023) A phantom cat at last: first melanistic jaguar record in northern Central America. Cat News 78: 31–33.

[B188] Martínez MA, Sánchez I (2020) Confirmación de la ocurrencia de *Galictis vittata* (Mustelidae) en la Reserva del Hombre y La Biosfera del Río Plátano en tiempos de la COVID-19. Scientia hondurensis 3(2): 11–14.

[B189] Martínez-Borrego D, Arellano E, González-Cózatl FX, Castro-Arellano I, León-Paniagua L, Rogers DS (2022) Molecular systematics of the *Reithrodontomys tenuirostris* group (Rodentia: Cricetidae) highlighting the *Reithrodontomys microdon* species complex. Journal of Mammalogy 103(1): 29–44. 10.1093/jmammal/gyab133 PMC8789765 35087329

[B190] Martinez-Murillo AM, Brown TW, Clayson SM (2021) The Bats (Chiroptera) of Utila Island, Honduras: species checklist and recommendations to conserve a cave roost. Journal of Bat Research and Conservation 14(1): 222–235. 10.14709/BarbJ.14.1.2021.19

[B191] Matson JO, Ordóñez-Garza N (2017) The taxonomic status of Long-tailed shrews (Mammalia: genus *Sorex*) from Nuclear Central America. Zootaxa 4236(3): 461–483. 10.11646/zootaxa.4236.3.3 28264313

[B192] Matson JO, Woodman N (2019) Shrews (Eulipotyphla, Soricidae) of Guatemala. Musarañas (Eulipotyphla, Soricidae) de Guatemala. Perspectivas de investigación sobre los mamíferos silvestres de Guatemala (Research Perspectives on the Wild Mammals of Guatemala).

[B193] Matson JO, Eckerlin RP, Consuegra SGP, Ordóñez-Garza N (2016) Small mammals from three mountain ranges in nuclear central America. Annals of Carnegie Museum 83(4): 269–285. 10.2992/007.083.0403

[B194] Matsuda AT, Matsuishi TF, Noto F, Amano M, Tajima Y, Yamada TK (2023) Notes on stomach contents of pygmy and dwarf sperm whales (*Kogia* spp.) from around Japan. In Advances in Marine Biology (Vol. 96, 1–24). Academic Press. 10.1016/bs.amb.2023.08.001 37980126

[B195] McCain CM (2001) First evidence of the giant anteater (*Myrmecophaga tridactyla*) in Honduras. The Southwestern Naturalist 46: 252–254. 10.2307/3672542

[B196] McCann NP, Wheeler PM, Coles T, Bruford MW (2012) Rapid ongoing decline of Baird’s tapir in Cusuco National Park, Honduras. Integrative Zoology 7(4): 420–428. 10.1111/j.1749-4877.2012.00312.x 23253373

[B197] McCarthy TJ, Davis WB, Hill JE, Jones Jr JK, Cruz GA (1993) Bat (Mammalia: Chiroptera) records, early collectors, and faunal lists for Northern Central America. Annals of Carnegie Museum 62(3): 191–228. 10.5962/p.226650

[B198] McCarthy TJ, Anderson D, Cruz GA (1999) Tree sloths (Mammalia: Xenarthra) in Nicaragua and Honduras, Central America. The Southwestern Naturalist 44: 410–414.

[B199] McCranie JR, Castañeda FEC (2007) Corregir esta referencia: Debe ser: McCranie JR, Castañeda FEC (2007) Guía de Campo de los Anfibios de Honduras. Bibliomania!, Salt Lake City, Utah. [x +] 304 pp. [+ 147 plates].

[B200] McCranie JR, Castañeda FE, Estrada N, Ferrufino L, Germer D, Matamoros W, Sagastume-Espinoza KO (2018) Biodiversity in Honduras: The environment, flora, bats, medium and large-sized mammals, birds, freshwater fishes, and the amphibians and reptiles. In: Pullaiah T (Ed.) Global biodiversity Vol. 4: Selected countries in the Americas and Australia. Apple Academic Press, 231–284. 10.1201/9780429433634-7

[B201] Medina-Fitoria A (2008) Evaluación Mastozoológica en la Reserva de Biosfera Río Plátano en el noreste de Honduras. Expedición de Historia Natural, Río Plátano. MOPAWI/SERNA (Proyecto Corazón), Tegucigalpa, Honduras, 21 pp.

[B202] Medina-Fitoria A [Ed.] (2018) Libro Rojo de los Mamíferos de Nicaragua. 1ª edición. Asociación Mastozoológica Nicaragüense AMAN - Centro de Investigación, Capacitación y Conservación CICFA. y Ministerio del Ambiente MARENA. Revista Nicaragüense de Biodiversidad 30: 1–85.

[B203] Medina-Fitoria A, Turcios-Casco MA (2019) Micromamíferos (Mammalia: Chiroptera y Rodentia) de Ciudad Blanca, La Moskitia, Honduras. In: Larsen TH (Ed.) Evaluación biológica rápida en Ciudad del Jaguar, Ciudad Blanca, La Moskitia, Honduras, Conservation International, 179–203.

[B204] Medina-Fitoria A, Martínez-Fonseca J, Gutiérrez A, van den Berghe E, Jarquín O, Aguirre Y, Salazar M, Robleto S, Toval N, Tórrez M, Díaz F (2018) Las ardillas de Nicaragua (Rodentia, Sciuridae). Revista Mexicana de Mastozoología (nueva época) 8(2): 48–80. 10.22201/ie.20074484e.2018.1.2.264

[B205] Medina-Fitoria A, Ávila-Palma HD, Martínez M, Ordoñez-Mazier DI, Turcios-Casco MA (2020) Los murciélagos (Chiroptera) del Caribe de Honduras y Nicaragua: una comparación sobre su diversidad en cinco diferentes coberturas vegetales. Neotropical Biodiversity 6(1): 147–161. 10.1080/23766808.2020.1804748

[B206] Medina-Van Berkum P, Vulinec K, Crace D, López-Gallego Z, Martin TE (2020) Community Composition of Bats in Cusuco National Park, Honduras, a Mesoamerican Cloud Park, Including new regional and Altitudinal Records. Neotropical Naturalist 3: 1–23.

[B207] Mejía-Ordoñez TM, House P (2002) Mapa de ecosistemas vegetales de Honduras. Preparado para el Proyecto PAAR, Tegucigalpa MDC, Honduras.

[B208] Mejía-Quintanilla DJ, Rodríguez-Herrera B, Spinola-Parallada M, Suazo-Euceda JPSE, Marineros L, Elvir F (2018) Situación del uso de la tierra en la distribución de cinco especies de murciélagos de la familia Emballonuridae en Honduras, Centroamérica. Revista Mexicana de Mastozoología (Nueva Época) 8(2): 13–21. 10.22201/ie.20074484e.2018.1.2.248

[B209] Mejía-Quintanilla DJ, Suazo-Euceda JP, Flores A, Gabriel Jackson M, Marineros L (2020) Dos nuevos registros de *Vampyrum spectrum* in Honduras. Revista Mexicana De Mastozoología (Nueva Época) 10(2): 59–64. 10.22201/ie.20074484e.2020.10.2.311

[B210] Mejía-Quintanilla DJ, Hernández J, Vega H, Marineros L, Spinolla-Parallada M, Rodríguez B, Reid F, Farrera M, Medina A, Mejía-Paniagua A, Elvir-Valle F (2022) Distribution of the bat family Mormoopidae in Honduras. Revista Mexicana de Mastozoología (Nueva Época) 12(2) 17–30. 10.22201/ie.20074484e.2022.12.2.372

[B211] Mérida JE, Cruz GA (2015) First record of the brown four-eyed opossum marsupial in Honduras (Río Plátano Biosphere Reserve). UNED Research Journal 7(2): 337–339. 10.22458/urj.v7i2.1163

[B212] Mérida Colindres JE, Cruz GA (2014) Confirmación de la presencia del oso hormiguero gigante *Myrmecophaga tridactyla centralis* (Xenarthra: Myrmecophagidae) en la Reserva Biósfera Río Plátano, Departamento de Gracias a Dios, Honduras, con descripción y comentarios sobre su estatus taxonómico. Edentata 15(2014): 9–15. 10.5537/020.015.0112

[B213] Merriam CH (1902) Twenty new pocket mice (*Heteromys* and *Liomys*) from Mexico. Proceedings of the Biological Society of Washington 41–50.

[B214] Meyer NFV, Moreno R, Sutherland C, de la Torre JA, Esser HJ, Jordan CA, Olmos M, Ortega J, Reyna-Hurtado R, Valdes S, Jansen PA (2020) Effectiveness of Panama as an intercontinental land bridge for large mammals. Conservation Biology 34(1): 207–219. 10.1111/cobi.13384 31385631

[B215] Meyer NFV, Brenes-Mora E, Dans AJ, Estrada N, Cabrera V, García MJ, Martínez W, Poot C, Reyna-Hurtado R, Rivero M, Jordan CA (2022) Ecology and conservation of the Baird’s Tapir in Mesoamerica. Imperiled: The Encyclopedia of Conservation 144–154. 10.1016/B978-0-12-821139-7.00173-2

[B216] Meza DE (2018) Línea base de fauna silvestre Reserva Biológica Montecillos. ASIDE, Siguatepeque, Honduras.

[B217] Midence S (2020) Primer registro fotográfico del cacomistle (*Bassariscus sumichrasti*) en el Parque Nacional Azul Meábear, Honduras. Scientia hondurensis 3(1): 26–28.

[B218] Midence López S (2020) Notas sobre mono araña (*Ateles geoffroyi*) en el Parque Nacional Azul Meámbar. Scientia hondurensis 3(2): 23–25.

[B219] Mies R, Kurta A, King DG (1996) *Eptesicus furinalis*. Mammalian Species 526: 1–7. 10.2307/3504316

[B220] Mignucci-Giannoni A, Swartz SL, Martinez A, Burks CM, Watkins WA (2003) First records of the pantropical spotted dolphin (*Stenella attenuata*) for the Puerto Rican Bank, with a review of the species in the Caribbean. Caribbean Journal of Science 39(3): 381–391.

[B221] Mignucci-Giannoni AA, Gonzalez-Socoloske D, Alvarez-Aleman A, Aquino J, Caicedo-Herrera D, Castelblanco-Martinez DN, Claridge D, Corona-Figueroa MF, Debrot AO, de Thoisy B, Espinoza-Marin C, Galves JA, Gracia-Alfonso E, Guzman HM, Khan JA, Kiszka JJ, Luna FO, Marmontel M, Olivera-Gomez LD, O’Sullivan C, Powell JA, Pugibet-Bobea E, Roopsing I, Silva CJ (2024) What’s in a name? Standardization of vernacular names for *Trichechus manatus*. Caribbean Naturalist 98: 1–17.

[B222] Miller BW (2001) Beyond mist-nets: what the rest of the bats can tell us about forests. The consequences of timber exploitation for bat communities in tropical America. In: Grajal A, Robinson JG (Eds) Cutting Edge: Conserving Wildlife in Logged Tropical Forest.s (Columbia University Press, New York, 154–156.

[B223] Miller BW, Miller CM (2000) Capiro y Calentura National Park and Laguna de Guaimoreto Wildlife Refuge during a survey of bats of the Capiro y Calentura National Park, Trujillo. Wildlife Conservation Society, Belize, 26 pp.

[B224] Miller BW, Miller CM (2025) History of Mammalogy in Belize. In: Melletti M, Tessaro SG, Ortega J, Tirira DG (Eds) Mammals of Middle and South America: History, Biogeography, Conservation. Springer, Cham, 1–24. 10.1007/978-3-031-43163-0_3-1

[B225] Miller BW, Ochoa J, Garcia FJ, O’Farrell MJ (2023) Distributional status in Venezuela of ghost bats of the genus *Diclidurus* Wied-Neuwied 1820 (Chiroptera: Emballonuridae), with a review of their acoustic vocal signatures. Acta Chiropterologica 25: 279–298. 10.3161/15081109ACC2023.25.2.008

[B226] Miller BW, Turcios-Casco MA, Ávila-Palma HD, Corben C, Ordoñez-Mazier DI (2024) Species accounts and acoustic keys for the Bats of Honduras. [online publication] 10.22541/au.169946248.82598189/v1

[B227] Miller C (2014) Host specificity and ectoparasite load of bat flies in Utila, Honduras. Senior Honors thesis, University of New Orleans, USA.

[B228] Minton G, Braulik GT, Reeves R (2018) *Globicephala macrorhynchus*. The IUCN Red List of Threatened Species 2018: e.T9249A50355227. 10.2305/IUCN.UK.2018-2.RLTS.T9249A50355227.en

[B229] Miranda R, Casalli DM, Perini FA, Machado FA, Santos FR (2017) Taxonomic review of the genus *Cyclopes* Gray (Xenarthra: Pilosa), with the revalidation and description of new species. Zoological Journal of the Linnean Society 183: 687–721. 10.1093/zoolinnean/zlx079

[B230] Molinari J (2026) A provisional classification of Hog-nosed Skunks, genus *Conepatus* Gray, 1837 (Mammalia, Carnivora, Mephitidae), from the Nearctic and Neotropical regions. Anartia 42: 63–106. 10.5281/zenodo.21417825

[B231] Molinari J, Gutiérrez EE, Lim BK (2023) Systematics and biogeography of *Anoura cultrata* (Mammalia, Chiroptera, Phyllostomidae): a morphometric, niche modeling, and genetic perspective, with a taxonomic reappraisal of the genus. Zootaxa 5297(2): 151–188. 10.11646/zootaxa.5297.2.1 37518801

[B232] Mora JM (2012) Big red bat *Lasiurus egregius* (Vespertilionidae) in Honduras. The Southwestern Naturalist 57(1): 104–105. 10.2307/41407881

[B233] Mora JM, López LI (2010) First record of the hoary bat (*Lasiurus cinereus*, Vespertilionidae) for Honduras. Ceiba 51(2): 89–90. 10.5377/ceiba.v51i2.1188

[B234] Mora JM, Ruedas LA (2023) Updated list of the mammals of Costa Rica, with notes on recent taxonomic changes. Zootaxa 5357(4): 451–501. 10.11646/zootaxa.5357.4.1 38220635

[B235] Mora JM, Montes de Oca G, Brenes BR (2010) Home range and movements of the hooded skunk (*Mephitis macroura*) in the rainy season on the Zamorano campus, Honduras. Ceiba 51(2): 45–53. 10.5377/ceiba.v51i2.953

[B236] Mora JM, Marineros L, López LI (2014) First record of the Striped Yellow-Eared bat, *Vampyriscus nymphaea*, (Stenodermatinae, Phyllostomidae) in Honduras. Caribbean Journal of Science 48(1): 49–51. 10.18475/cjos.v48i1.a7

[B237] Mora JM, Espinal MR, Ruedas LA, López LI (2016) The big free-tailed bat, *Nyctinomops macrotis* (Gray, 1839), in Central America. Mastozoología neotropical 23(2): 551–556.

[B238] Mora JM, López LI, Espinal M, Marineros L, Ruedas L (2018) Diversidad y conservación de los murciélagos de Honduras. Master Print S. de RL, Tegucigalpa.

[B239] Mora JM, Espinal MR, López LI (2020) New records on distribution and habitat of the lesser long-nosed bat (*Leptonycteris yerbabuenae*) in Honduras. Neotropical Biology and Conservation 15(4): 521–531. 10.3897/neotropical.15.e57376

[B240] Mora JM, López LI, Espinal MR (2021) Clave de campo para la identificación de los murciélagos de Honduras. Notas sobre Mamíferos Sudamericanos 3: e21. 10.31687/saremNMS.21.6.1

[B241] Morales-Martínez DM, López-Arévalo HF, Vargas-Ramírez M (2021) Beginning the quest: phylogenetic hypothesis and identification of evolutionary lineages in bats of the genus *Micronycteris* (Chiroptera, Phyllostomidae). ZooKeys 1028: 135–159. 10.3897/zookeys.1028.60955 PMC8044067 33889049

[B242] Morgan GS (1989) *Geocapromys thoracatus*. Mammalian Species (341): 1–5. 10.2307/3504186

[B243] Moyne WEG (1938) Atlantic Circle. Blackie and Sons Ltd. London and Glasgow.

[B244] Murcia FV, Brown TW, Ramos EA (2024) Stranding of a newborn false killer whale on Utila Island, Honduras. Caribbean Journal of Science 54(2): 421–427. 10.18475/cjos.v54i2.a20

[B245] Musser GG (1969) Notes on *Peromyscus* (Muridae) of Mexico and Central America: No. 2357. American Museum of Natural History.

[B246] Niño-Torres CA, García-Rivas MDC, Castelblanco-Martínez DN, Padilla-Saldívar JA, Blanco-Parra MDP, de la Parra-Venegas R (2015) Aquatic mammals from the Mexican Caribbean; a review. Hidrobiológica 25(1): 127–138.

[B247] Ochoa GM, Ramos EA, Gonzalez-Socoloske D (2025) Predation of a sperm whale by killer whales (*Orcinus orca*) in Guanaja, Honduras. Aquatic Mammals 51(1): 31–36. 10.1578/AM.51.1.2025.31

[B248] Ochoa J, O’Farrell MJ, Miller BW (2000) Contribution of acoustic methods to the study of insectivorous bat diversity in protected areas from northern Venezuela. Acta Chiropterologica 2: 171–183.

[B249] O’Farrell MJ, Gannon WL (1999) A comparison of capture versus acoustic techniques for bat inventories. Journal of Mammalogy 80: 24–30. 10.2307/1383204

[B250] O’Farrell MJ, Miller BW (1997) A new examination of echolocation calls of some Neotropical bats (Emballonuridae and Mormoopidae). Journal of Mammalogy 87: 954–963. 10.2307/1382955

[B251] O’Farrell MJ, Miller BW (1999) Use of vocal signatures for the inventory of free-flying Neotropical bats. Biotropica 31(3): 507–516. 10.1111/j.1744-7429.1999.tb00394.x

[B252] Olivera-Gómez LD, Mellink E (2005) Distribution of the Antillean manatee (*Trichechus manatus manatus*) as a function of habitat characteristics in Bahía de Chetumal, Mexico. Biological Conservation 121(1): 127–133. 10.1016/j.biocon.2004.02.023

[B253] Ordóñez-Garza N, Thompson CW, Unkefer MK, Edwards CW, Owen JG, Bradley RD (2014) Systematics of the Neotoma mexicana species group (Mammalia: Rodentia: Cricetidae) in Mesoamerica: new molecular evidence on the status and relationships of *N. ferruginea* Tomes, 1862. Proceedings of the Biological Society of Washington 127(3): 518–532. 10.2988/0006-324X-127.3.518

[B254] Ordoñez-Mazier DI, Ávila-Palma HD, Soler-Orellana JA, Ordoñez-Trejo EJ, Martínez M, Turcios-Casco MA (2020) Rediscovery of *Vampyressa thyone* (Chiroptera, Phyllostomidae) in Honduras: updated distribution and notes on its conservation. Neotropical Biology and Conservation 15(3): 379–390. 10.3897/neotropical.15.e53766

[B255] Oremus M, Poole M, Steel D, Baker C (2007) Isolation and interchange among insular spinner dolphin communities in the South Pacific revealed by individual identification and genetic diversity. Marine Ecology Progress Series 336: 275–289. 10.3354/meps336275

[B256] Ortiz-Wolford JS, Corona-Figueroa MF, Coronado OHM, Petersen AG (2022) Cetacean sightings in the Caribbean Sea of Guatemala. Latin American Journal of Aquatic Mammals 17(1): 51–58. 10.5597/lajam00279

[B257] Pardiñas UFJ, Ruelas D, Brito J, Bradley LC, Bradley RD, Garza NO, Kryštufek B, Cook J, Cuéllar Soto E, Salazar-Bravo J (2017) Cricetidae (true hamsters, voles, lemmings and new world rats and mice) - species accounts of Cricetidae. In: Wilson DE, Lacher Jr TE, Mittermeier RA (Eds) Handbook of the mammals of the world. Rodents II 7: 280–535. [accessed Jun 02 2026]

[B258] Patterson BD, Ramírez-Chaves HE, Vilela JF, Soares AER, Grewe F (2021) On the nomenclature of the American clade of weasels (Carnivora: Mustelidae). Journal of Animal Diversity 3(2): 1–8. 10.52547/JAD.2021.3.2.1

[B259] Pavan AC, Marroig G (2017) Timing and patterns of diversification in the Neotropical bat genus *Pteronotus* (Mormoopidae). Molecular Phylogenetics and Evolution 108: 61–69. 10.1016/j.ympev.2017.01.017 28189619

[B260] Paz-Sánchez MA (2023) Comportamiento de frotamiento sobre el pelaje de monos araña centroamericanos (*Ateles geoffroyi* Kuhl, 1820; Atelidae) en entorno de cautiverio en Honduras. Neotropical Primates 29: 2. 10.62015/np.2023.v29.794

[B261] Peñalva Morales K, Vega Rodríguez H, Orellana Canales W (2025) First photographic records of *Puma concolor* (Carnivora: Felidae) in Celaque Mountain National Park, Honduras. Therya Notes 6: 184–189. 10.12933/therya_notes-24-218

[B262] Peppers LL, Bradley RD (2000) Cryptic species in *Sigmodon hispidus*: evidence from DNA sequences. Journal of Mammalogy 81(2): 332–343. 10.1644/1545-1542(2000)081<0332:CSISHE>2.0.CO;2

[B263] Perdomo K, Vega H (2019) Registro de *Chrotopterus auritus* (Phyllostomidae) en el Parque Nacional Montaña de Celaque. Scientia hondurensis 2(2): 20–22.

[B264] Pérez-Consuegra SG, Vázquez-Domínguez E (2015) Mitochondrial diversification of the *Peromyscus mexicanus* species group in Nuclear Central America: biogeographic and taxonomic implications. Journal of Zoological Systematics and Evolutionary Research 53(4): 300–311. 10.1111/jzs.12099

[B265] Pérez-Consuegra SG, Vázquez-Domínguez E (2016) Intricate evolutionary histories in montane species: a phylogenetic window into craniodental discrimination in the *Peromyscus mexicanus* species group. Journal of Zoological Systematics and Evolutionary Research 55(1): 57–72. 10.1111/jzs.12155

[B266] Pérez-Consuegra SG, Sánchez-Tovar L, Rodríguez-Tapia G, Castañeda-Rico S, Vázquez-Domínguez E (2023) Late Pleistocene altitudinal segregation and demography define future climate change distribution of the *Peromyscus mexicanus* species group: Conservation implications. Animals 13(11): 1753. 10.3390/ani13111753 PMC10251973 37889659

[B267] Perryman WL, Danil K (2018) Melon-headed whale: *Peponocephala electra*. In: Perrin WF, Würsig B, Thewissen JGM (Eds) Encyclopedia of Marine Mammals (3^rd^ edn.). Elsevier, San Diego, 593–595. 10.1016/B978-0-12-804327-1.00171-0

[B268] Pinel Ramos EJ (2020) Population density estimate for the white-faced capuchin monkey (*Cebus imitator*) in Honduras. Neotropical Primates 26(1): 47–55. 10.62015/np.2020.v26.79

[B269] Pitman RL, Brownell Jr RL (2020) Mesoplodon europaeus. The IUCN Red List of Threatened Species 2020: e.T13245A50365198. 10.2305/IUCN.UK.2020-3.RLTS.T13245A50365198.en

[B270] Portillo HO, Elvir F, Hernández J, Leiva F, Flores ME, Martínez I, Vega H (2015) Datos preliminares de la densidad poblacional de *Odocoileus virginianus* en el Parque Nacional La Tigra, Honduras. Mesoamericana 19(2): 23–30.

[B271] Portillo HOR, Vásquez M (2009) Expedición y evaluación de la Reserva de la Biosfera Tawahka. WCS–ICF, Honduras.

[B272] Portillo RI, Sandoval JEB, Pineda L, de Navas EM, Mejía MGP, Fuentes REM (2021) Registros de varamientos de cetáceos en El Salvador (1995–2019) Realidad y Reflexión 54: 218–240. 10.5377/ryr.v54i54.12080

[B273] Portillo Reyes HO, Elvir F (2016) Distribución potencial de la jagüilla (*Tayassu pecari*) en Honduras. Revista Mexicana de Mastozoología (nueva época) 6(1): 15–23. 10.22201/ie.20074484e.2016.6.1.217

[B274] Portillo Reyes HO, Hernández J, Manzanares T, Elvir F, Vega H (2015) Registros y distribución potencial del murciélago blanco hondureño (*Ectophylla alba*) en La Moskitia, Honduras. Revista Mexicana de Mastozoología 5(1): 25–32. 10.22201/ie.20074484e.2015.5.1.205

[B275] Portillo Reyes HO, Marineros L, Mejía D, Perla J, Manzanares T (2021) Distribución potencial de *Cyclopes dorsalis* en Honduras. Scientia hondurensis 4(2): 1–7.

[B276] Portillo-Reyes HO, Martínez M (2024) Urgent prioritization of conservation sites for the jagüilla (*Tayassu pecari*) in the Honduran Moskitia region. Therya 15(1): 71–75. 10.12933/therya-24-5879

[B277] Puckett EE, Park J, Combs M, Blum MJ, Bryant JE, Caccone A, Costa F, Deinum EE, Esther A, Himsworth CG, Keightley PD, Ko A, Lundkvist Å, McElhinney LM, Morand S, Robins J, Russell J, Strand TM, Suarez O, Yon L, Munshi-South J (2016) Global population divergence and admixture of the brown rat (*Rattus norvegicus*). Proceedings of the Royal Society B: Biological Sciences 283(1841): 20161762. 10.1098/rspb.2016.1762 PMC5095384 27798305

[B278] Quick NJ, Cioffi WR, Shearer JM, Fahlman A, Read AJ (2020) Extreme diving in mammals: first estimates of behavioural aerobic dive limits in Cuvier’s beaked whales. Journal of Experimental Biology 223(18): jeb222109. 10.1242/jeb.222109 32967976

[B279] Quintana-Rizzo E (2005) Estudio sinóptico de la distribución y abundancia relativa del manatí (*Trichechus manatus*) en el Golfo de Honduras en el periodo de mayo–junio 2005. Reporte técnico para el Comitato Internazionale per lo Sviluppo dei Popoli (CISP), Ciudad de Guatemala, Guatemala.

[B280] Ramírez Fernández JD, Sánchez R, May-Collado LJ, González-Maya JF, Rodríguez-Herrera B (2023) Revised checklist and conservation status of the mammals of Costa Rica. Therya 14(2): 233–244. 10.12933/therya-23-2142

[B281] Ramírez-Chaves H, Morales-Martínez DM, Rodríguez-Posada ME, Suárez-Castro AF (2021) Checklist of the mammals (Mammalia) of Colombia. Mammalogy Notes 7(2): 253–253. 10.47603/mano.v7n2.253

[B282] Ramírez-Chaves HE, Cifuentes MA, Noguera-Urbano EA, Pérez WA, Torres-Martínez MM, Ossa-López PA, Rivera-Páez FA, Morales-Martínez DM (2023) Systematics, morphometry, and distribution of *Eptesicus fuscus miradorensis* (Chiroptera: Vespertilionidae), with notes on baculum morphology and natural history. Therya 14(2): 299–311. 10.12933/therya-23-2290

[B283] Ramírez-Fernández JD, Sánchez R, May-Collado LJ, González-Maya JF, Rodríguez-Herrera B (2023) Revised checklist and conservation status of the mammals of Costa Rica. Therya 14(2): 233–244. 10.12933/therya-23-2142

[B284] Ramos EA, Castelblanco-Martínez DN, Niño-Torres CA, Jenko K, Gómez NA (2016) A review of the aquatic mammals of Belize. Aquatic Mammals 42(4): 476–497. 10.1578/AM.42.4.2016.476

[B285] Ramos EA, Castelblanco-Martínez DN, García J, Arias JR, Foley JR, Audley K, Van Aerebeek K, Van Bressem MF (2018) Lobomycosis-like disease in common bottlenose dolphins (*Tursiops truncatus*) from Belize and Mexico: bridging the gap between the Americas. Diseases of Aquatic Organisms 128(1): 1–12. 10.3354/dao03206 29565249

[B286] Ramos EA, Izaguirre Luque AP, Herrera L, Antúnez E, Brown TW, Castelblanco-Martínez DN (2020) Stranding of a pregnant dwarf sperm whale (*Kogia sima*) in Utila, Honduras. Latin American Journal of Aquatic Mammals 15(1): 25–29. 10.5597/lajam00258

[B287] Ramos EA, Kiszka JJ, Pouey‐Santalou V, Ramirez Barragan R, Garcia Chavez AJ, Audley K (2021) Food sharing in rough‐toothed dolphins off southwestern Mexico. Marine Mammal Science 37(1): 352–360. 10.1111/mms.12727

[B288] Rasmussen K (2006) Comparison of two distinct populations of humpback whales (*Megaptera novaeangliae*) off Pacific Central America. M.Sc. Thesis, Moss Landing Marine Laboratories, San Francisco State University.

[B289] Rathbun GB, Powell JA, Cruz G (1983) Status of the West Indian manatee in Honduras. Biological Conservation 26(4): 301–308. 10.1016/0006-3207(83)90094-0

[B290] Reid FA (2009) A field guide to the mammals of Southeast Mexico and Central America. Oxford University Press, New York.

[B291] Reséndiz-López MA (2025) Listado taxonómico de mamíferos con distribución en México. Version 1.3. No organization. Checklist dataset. https://www.snib.mx/iptconabio/resource?r=mamiferos

[B292] Reyes HOP, Matamoros WA, Glowinski SL (2010) Distribution and conservation status of the giant anteater (*Myrmecophaga tridactyla*) in Honduras. The Southwestern Naturalist, 118–120. 10.1894/CLG-25.1

[B293] Reyes HOP, Marineros L, Mejía D, Perla J, Manzanares T (2021) El perezosito *Cyclopes dorsalis* (Pilosa: Cyclopedidae) y su distribución potencial en Honduras. Scientia hondurensis 4(2): 1–7.

[B294] Rezsutek M, Cameron GN (1993) *Mormoops megalophylla*. Mammalian Species 448: 1–5. 10.2307/3504289

[B295] Rice DW (1973) Caribbean monk seal (*Monachus tropicalis*). In Proceedings of a working meeting of seal specialists on threatened and depleted seals of the world (98–112). International Union for Conservation of Nature and Natural Resources.

[B296] Rice EDW (1960) Population dynamics of the Hawaiian monk seal. Journal of Mammalogy 41: 376–385. 10.2307/1377496

[B297] Rodrigues MLF (2013) História evolutiva de *Conepatus* (Carnivora: Mephitidae): Padrões biogeográficos de diversificação, investigação filogenética e revisão taxonómica do gênero. Porto Alegre: Pontifícia Universidade Católica do Río Grande do Sul, 162 pp. [PhD Thesis]

[B298] Rogers DS, González MW (2010) Phylogenetic relationships among spiny pocket mice (*Heteromys*) inferred from mitochondrial and nuclear sequence data. Journal of Mammalogy 91: 914–930. 10.1644/09-mamm-a-287.1

[B299] Rudnick A (1960) A revision of the mites of the family Spinturnicidae (Acarina). University of California Publications in Entomology 17: 157–283.

[B300] Ruedas LA, López LI, Mora JM (2023) A propaedeutic to the taxonomy of the eastern cottontail rabbit (*Sylvilagus floridanus*) from Central America. Therya 14(1): 99–119. 10.12933/therya-23-2203

[B301] Ruiz-Garcia M, Cáceres AM, Luengas-Villamil K, Aliaga-Rossel E, Zeballos H, Singh MD, Shostell JM (2022) Mitogenomic phylogenetics and population genetics of several taxa of agoutis (*Dasyprocta* spp.). Mammal Research 67(3): 367–397. 10.1007/s13364-022-00626-6

[B302] Sagastume-Espinoza KO, Romero SJ (2017) First photographic evidence of the iconic big mammals of Honduras, Baird’s tapir (*Tapirus bairdii*) and jaguar (*Panthera onca*), in La Muralla Wildlife Refuge. Therya 8(1): 63–65. https://therya.mastozoologiamexicana.com/index.php/THERYA/article/view/448

[B303] Sanborn CC (1935) New mammals from Guatemala and Honduras. Field Museum of Natural History 20(1): 81–85.

[B304] Sánchez K, Aguilar F, Donaire D, Coll J, Arauz W, Andrade Y, Carías MA, Gómez-Corea W (2023) *Puma concolor*: first photographic record in El Jilguero Reserve Water Production Zone, Honduras. Therya Notes 4: 242–248. 10.12933/therya_notes-23-136

[B305] Schank C, Mendoza E, Vettorazzi MJG, Cove MV, Jordan CA, O’Farrill G, Meyer N, Lizcano DJ, Estrada N, Poot C, Leonardo R (2015) Integrating current range-wide occurrence data with species distribution models to map the potential distribution of Baird’s tapir. Tapir Conservation 24(33): 15–25.

[B306] Schorr GS, Falcone EA, Moretti DJ, Andrews RD (2014) First long-term behavioral records from Cuvier’s beaked whales (*Ziphius cavirostris*) reveal record-breaking dives. PLoS ONE 9(3): e92633. 10.1371/journal.pone.0092633 PMC3966784 24670984

[B307] Segura V, Prevosti F, Cassini G (2013) Cranial ontogeny in the puma lineage (*Puma concolor*, *Herpailurus yagouaroundi*, *Acinonyx jubatus*). Zoological Journal of the Linnean Society 169(1): 235–250. 10.1111/zoj.12047

[B308] Sikes RS, The Animal Care and Use Committee of the American Society of Mammalogists (2016) Guidelines for the use of wild mammals in research and education. Journal of Mammalogy 97: 663–688. 10.1093/jmammal/gyz019 PMC5909806 29692469

[B309] Siles L, Baker RJ (2020) Revision of the pale-bellied *Micronycteris*. Journal of Zoological Systematics and Evolutionary Research 58(4): 1411–1431. 10.1111/jzs.12388

[B310] Silva FP, Silva LGD, Semedo TB, Santos T, Lopes GP, Montes MA, Garbino GS (2023) Pleistocene distribution of MacConnell’s bat suggests connections between Amazonia and Atlantic Forest. Therya 14(1): 55–62. 10.12933/therya-23-2219

[B311] Silva LG (2017) Ecology and evolution of melanism in big cats: case study with black leopards and jaguars. In: Big Cats. Academic Press. InTech. 10.5772/intechopen.69558

[B312] Simmons NB, Cirranello AL (2026) Bat Species of the World: A taxonomic and geographic database. Version 1.10. https://batnames.org/ [Accessioned 2026]

[B313] Spradling TA, Demastes JW, Hafner DJ, Milbach PL, Cervantes FA, Hafner MS (2016) Systematic revision of the pocket gopher genus *Orthogeomys*. Journal of Mammalogy 97(2): 405–423. 10.1093/jmammal/gyv185

[B314] Strong WD (1935) Archaeological investigations in the Bay Islands, Spanish Honduras. Smithsonian Miscellaneous Collections 92(14): 1–172.

[B315] Tate GHH (1933) A systematic revision of the marsupial genus *Marmosa*. Bulletin of the American Museum of Natural History 66: 67–72.

[B316] Teta P, Reyes-Amaya N (2021) Morphology and species boundaries in *Dasyprocta*. Journal of Mammalogy 102(6): 1548–1563. 10.1093/jmammal/gyab101

[B317] Teta P, Abba AM, Cassini GH, Flores DA, Galliari CA, Lucero SO, Ramírez M (2018) Lista revisada de los mamíferos de Argentina. Mastozoología Neotropical 25(1): 163–198. 10.31687/saremMN.18.25.1.0.15

[B318] Thomas O (1892–1912) [series entries] Various taxonomic descriptions. Annals and Magazine of Natural History.

[B319] Thomas O (1898) On indigenous Muridæ in the West Indies; with the description of a new Mexican *Oryzomys*. Annals and Magazine of Natural History, Series 1(2): 176–180. 10.1080/00222939808677948

[B320] Thornton D, Reyna R, Perera-Romero L, Radachowsky J, Hidalgo-Mihart MG, Garcia R, McNab R, Mcloughlin L, Foster R, Harmsen B, Moreira-Ramírez JF, Diaz-Santos F, Jordan C, Salom-Pérez R, Meyer N, Castañeda F, Valle FAE, Santizo GP, Amit R, Arroyo-Arce S, Thomson I, Moreno R, Schank C, Arroyo-Gerala P, Bárcenas HV, Brenes-Mora E, Calderón AP, Cove MV, Gomez-Hoyos D, González-Maya J, Guy D, Jiménez GH, Hofman M, Kays R, King T, Menjivar MAM, de la Maza J, León-Pérez R, Ramos VH, Rivero M, Romo-Asunción S, Juárez-López R, Jesús-de A la Cruz, de la Torre JA, Towns V, Schipper J, Reyes HOP, Artavia A, Hernández-Perez E, Martínez W, Urquhart GR, Quigley H, Pardo LE, Sáenz JC, Sanchez K, Polisar J (2020) Precipitous decline of white-lipped peccary populations in Mesoamerica. Biological Conservation 242: 108410. 10.1016/j.biocon.2020.108410

[B321] Timm RM, Salazar RM, Peterson AT (1997) Historical distribution of the extinct tropical seal (*Monachus tropicalis*). Conservation Biology 11(2): 549–551. 10.1046/j.1523-1739.1997.96035.x

[B322] Tirira DG, Brito J, Burneo SF, Carrera-Estupiñán JP, Comisión de Diversidad de la AEM (2021) Mamíferos del Ecuador: lista oficial actualizada de especies / Mammals of Ecuador: official updated species checklist. 2021.1. Asociación Ecuatoriana de Mastozoología.

[B323] Trujillo LA, Escobar-Anleu BI (2023) First record of the genus *Metachirus* in Guatemala. Therya Notes 4: 97–100. 10.12933/therya_notes-23-113

[B324] Trujillo LA, Martínez-Fonseca JG, Hernández J, Cajas-Castillo JO, Girón-Galván L, Medina-Fitoria A, Rodríguez ME, Barahona-Fong R (2022) Notes on the life history of *Centurio senex* (Chiroptera: Phyllostomidae) from northern Central America. Mammalia 86(5): 468–473. 10.1515/mammalia-2021-0178

[B325] Turcios-Casco MA (2026) History of Mammalogy in Honduras. In: Melletti M, Gallina Tessaro S, Ortega J, Tirira DG (Eds) Mammals of Middle and South America: History, Biogeography, Conservation. Handbook of the Mammals of Middle and South America. Springer, Cham. 10.1007/978-3-031-43163-0_14-1

[B326] Turcios-Casco MA, López CM (2024) Safeguard the endemic species of Honduras. Science 385(6712): 941–942. 10.1126/science.adp8662 39208086

[B327] Turcios-Casco MA, López CM (2025) Especímenes, fechas y problemas científico-sociales: La situación crítica de las colecciones zoológicas en Honduras. Mastozoología Neotropical 32(2): e01094. 10.31687/saremMN.25.32.02.05.e1094

[B328] Turcios-Casco MA, López CM (2025) Especímenes, fechas y problemas científico-sociales: La situación crítica de las colecciones zoológicas en Honduras. Mastozoología Neotropical 32(2): e01094. 10.31687/saremMN.25.32.02.05.e1094

[B329] Turcios-Casco MA, Medina-Fitoria A (2019) Occurrence of *Hylonycteris underwoodi* (Chiroptera, Phyllostomidae) and *Thyroptera tricolor* (Chiroptera, Thyropteridae) in Honduras. Studies on Neotropical Fauna and Environment 54(1): 69–72. 10.1080/01650521.2018.1544205

[B330] Turcios-Casco MA, Mazier DIO, Orellana JAS, Ávila-Palma HD, Trejo EJO (2019a) Two caves in western Honduras are important for bat conservation: first checklist of bats in Santa Bárbara. Subterranean Biology 30: 41–55. 10.3897/subtbiol.30.35420

[B331] Turcios-Casco MA, Ávila-Palma HD, Ordoñez EJT, Orellana JAS, Ordoñez DIM (2019b) Comments on the diet of phyllostomid bats (Chiroptera) in a subtropical dry forest in central Honduras. Studies on Neotropical Fauna and Environment 54(3): 239–244. 10.1080/01650521.2019.1663114

[B332] Turcios-Casco MA, Ávila-Palma HD, LaVal RK, Stevens RD, Ordoñez-Trejo EJ, Soler-Orellana JA, Ordoñez-Mazier DI (2020a) A systematic revision of the bats (Chiroptera) of Honduras: an updated checklist with corroboration of historical specimens and new records. Zoosystematics and Evolution 96(2): 411–429. 10.3897/zse.96.51059

[B333] Turcios-Casco MA, Ávila-Palma HD, Martínez M, Trejo-Ordoñez EJ, Meza-Flores DE, Castañeda F, King T, Thornton D, Superina M (2020b) The xenarthrans of Honduras: new records, main threats, and comments on their conservation status. Edentata 21: 13–28. 10.2305/IUCN.CH.2020.Edentata-20-1.3.en

[B334] Turcios-Casco MA, Medina-Fitoria A, Manzanares T, Martínez M, Ávila-Palma HD (2020c) Sobre la distribución de tres especies de murciélagos filostómidos (Chiroptera), con registros adicionales para el Departamento de Gracias a Dios, Honduras. Graellsia 76(1): 1113–1118. 10.3989/graellsia.2020.v76.256

[B335] Turcios-Casco MA, Mejía-Suazo CJ, Ordoñez Bautista DJ, Ávila-Palma HD (2020d) Noteworthy records of the Geoffroy’s tailless bat and the Eastern Pipistrelle in Copán, western Honduras (MammaliaChiroptera). Biodiversity Journal 11(2): 527–534. 10.31396/Biodiv.Jour.2020.11.2.527.534

[B336] Turcios-Casco MA, Ávila-Palma HD, Ordoñez EJT, Soler JAS, Ordoñez DIM, Meza-Flores DE, Velásquez A (2020e) Rare or misidentified? On the external identification of the neglected *Artibeus inopinatus* Davis and Carter, 1964 (Chiroptera, Phyllostomidae) in Honduras. Evolutionary Systematics 4(1): 35–43. 10.3897/evolsyst.4.49377

[B337] Turcios-Casco MA, Ávila-Palma HD, Soler-Orellana JA, Ordoñez-Trejo EJ, Mazier-Ordoñez DI, Velásquez A, Estrada-Andino N (2020f) Diversidad de murciélagos (Chiroptera) de Sabanagrande, República de Honduras. Notas sobre Mamíferos Sudamericanos 2(1). 10.31687/saremNMS.20.0.18

[B338] Turcios-Casco MA, Soler-Orellana JA, Meza-Flores DE (2021a) ¿Cuántas especies de primates hay en Honduras? Neotropical Primates 27(1): 42–46. 10.62015/np.2021.v27.67

[B339] Turcios-Casco MA, LaVal RK, Wilson DE, Ávila-Palma HD (2021b) Bats in time: historical and geographic distribution in Honduras. Occasional Papers The Museum Texas Tech University 375: 1–24. https://www.depts.ttu.edu/nsrl/publications/downloads/OP375.pdf

[B340] Turcios-Casco MA, Ávila-Palma HD, Soler JAO, Ordoñez EJT, Ordoñez DIM (2021c) Patrones de actividad de murciélagos filostómidos en un bosque seco en Honduras. Therya Notes 2: 34–38. https://theryanotes.mastozoologiamexicana.com/index.php/theryanotes/article/view/82

[B341] Turcios-Casco MA, Meza D, Martínez M (2023a) Distribución actualizada de *Cabassous centralis* (Cingulata: Chlamyphoridae) en Honduras. Edentata 23: 19–22. 10.2305/IUCN.CH.2022.Edentata-23-1.5.en

[B342] Turcios-Casco MA, Hernández GS, Mancía FE, Molinero CF, Muñoz J, López CM, Ordóñez-Garza N (2023b) Unseen for 43 years! A new occurrence of *Glaucomys volans* (Linnaeus, 1758) (Rodentia, Sciuridae) in Honduras. Check List 19(1): 133–139. 10.15560/19.1.133

[B343] Turcios-Casco MA, Velásquez A, Almeida OJG, Ordóñez-Garza N, López CM (2023c) Notas de distribución de *Bassaricyon gabbii* (Carnivora: Procyonidae) para Honduras, con comentarios de su ocurrencia para la región. Notas sobre Mamíferos Sudamericanos 5: 1: 10.31687/SaremNMS23.1.2

[B344] Turcios-Casco MA, Cardoso VC, Lee Jr TE (2024) Back to the future: A preserved specimen validates the presence of *Molossus pretiosus* (Molossidae, Chiroptera) in Honduras. ZooKeys 1196: 139–148. 10.3897/zookeys.1196.116144 PMC10980877 38560092

[B345] Turcios-Casco MA, Martínez-Fonseca JG, Miller BW (2025a) Listening northward: first evidence of *Cormura brevirostris* (Wagner, 1843) (Emballonuridae, Chiroptera) in Honduras. Check List 21(6): 1262–1270. 10.15560/21.6.1262

[B346] Turcios-Casco MA, López CM, Bautista Alcantara LF, Velásquez A, Ordóñez-Mazier DI, Mungia N, García Almeida OJ (2025b) A mammal dilemma? Description of interspecific relationships within two threatened cacti in northern Honduras. Brazilian Journal of Botany 48: 39. 10.1007/s40415-025-01082-7

[B347] Turcios-Casco MA, Teta P, Reyes-Amaya N, López CM, Wilson DE, Schiavetti A (2026) Quantitative morphology supports the synonymy of *Dasyprocta ruatanica* O. Thomas, 1901 under *D. punctata* J. E. Gray, 1842 (Rodentia: Dasyproctidae). Journal of Mammalian Evolution 33: 5. 10.1007/s10914-026-09807-5

[B348] Tyne JA, Johnston DW, Rankin R, Loneragan NR, Bejder L (2015) The importance of spinner dolphin (*Stenella longirostris*) resting habitat: Implications for management. Journal of Applied Ecology 52(3): 621–630. 10.1111/1365-2664.12434

[B349] Valdez R, LaVal RK (1971) Records of bats from Honduras and Nicaragua. Journal of Mammalogy 52(1): 247–250. 10.2307/1378465

[B350] Vallejo-Ham A, Castelblanco-Martínez N, Pinel AL (2026) Fatal asphyxiation with plastic debris in a melon-headed whale (*Peponocephala electra*) off the coast of Honduras, and first record of the species for the country. Aquatic Mammals 52(1): 20–26. 10.1578/AM.52.1.2026.20

[B351] Van Dort J, Peña-Acuña M, Juárez R, Komar O (2022) Composition, sex ratio and reproductive phenology of a bat assemblage from a fragmented landscape in the Honduran highlands. Mastozoología Neotropical 29(1): 1–31. 10.31687/saremMN.22.29.1.03.e0590

[B352] von Lehmann, Schaefer HE (1979) Cytologisch‐taxonomische Studien an einer Kleinsaugeraufsammlung aus Honduras (Spermienmorphologie und vergleichende Cytochemie) 1. Journal of Zoological Systematics and Evolutionary Research 17(3): 226–236. 10.1111/j.1439-0469.1979.tb00703.x

[B353] Voss RS (2024) A new genus for the “alfaroi group” of *Oryzomys* sensu lato (Rodentia: Cricetidae: Sigmodontinae). American Museum Novitates 2024(4030): 1–12. 10.1206/4030.1

[B354] Voss RS, Jansa SA (2021) Opossums: an adaptive radiation of New World marsupials. Johns Hopkins University Press, Baltimore.

[B355] Voss RS, Giarla TC, Jansa SA (2021) A revision of the didelphid marsupial genus *Marmosa*. Part 4. Species of the Alstoni group (subgenus *Micoureus*). American Museum Novitates 3983: 1–31. 10.1206/3983.1

[B356] WCS (2021) Lista Roja de especies amenazadas de Honduras [Informe técnico]. Tegucigalpa, Honduras: WCS, MiAmbiente, UNAH-VS, ICF, IUCN.

[B357] Weksler M (2006) Phylogenetic relationships of oryzomyine rodents (Muroidea: Sigmodontinae): separate and combined analyses of morphological and molecular data. Bulletin of the American Museum of Natural History 296: 1–149. 10.1206/0003-0090(2006)296[0001:PROORM]2.0.CO;2

[B358] Wells RS, Scott MD (2018) Bottlenose dolphin, *Tursiops truncatus*, common bottlenose dolphin. In Encyclopedia of marine mammals (118–125). Academic Press. 10.1016/B978-0-12-804327-1.00072-8

[B359] Wells RS, Natoli A, Braulik GT (2019) *Tursiops truncatus* (errata version published in 2019). The IUCN Red List of Threatened Species 2019: e.T22563A156932432. 10.2305/IUCN.UK.2019-1.RLTS.T22563A156932432.en

[B360] Wells W (1857) Explorations and Adventures in Honduras. Harper and Brothers, New York, 588 pp.

[B361] West KL, Walker WA, Baird RW, Mead JG, Collins PW (2017) Diet of Cuvier’s beaked whales (*Ziphius cavirostris*) from the North Pacific and a comparison with their diet worldwide. Marine Ecology Progress Series 574: 227–242. 10.3354/meps12214

[B362] Whitehead H, Barlow J, Dawson SM, Eguiguren A, Miller B, Shabangu F (2025) *Physeter macrocephalus*. The IUCN Red List of Threatened Species 2025: e.T41755A50382216. 10.2305/IUCN.UK.2025-2.RLTS.T41755A50382216.en

[B363] Woodman N (2000) *Cryptotis merriami* Choate in Costa Rica: syntopy with *Cryptotis* nigrescens and possible character displacement. Caribbean Journal of Science 36(3–4): 289–299.

[B364] Woodman N (2011) Patterns of morphological variation amongst semifossorial shrews in the highlands of Guatemala. Zoological Journal of the Linnean Society 163(4): 1267–1288. 10.1111/j.1096-3642.2011.00754.x

[B365] Woodman N (2015) Morphological variation among broad-clawed shrews from Honduras, with descriptions of three new cryptic species. Annals of Carnegie Museum 83(2): 95–119. 10.2992/007.083.0203

[B366] Woodman N (2019) Three new species of small-eared shrews from El Salvador, Guatemala, and Honduras. Special Publications of the Museum of Texas Tech University 72: 1–46.

[B367] Woodman N, Croft DA (2005) Fossil shrews from Honduras and their significance. Fieldiana Geology 51: 1–30. 10.3158/0096-2651(2005)51[1:FSFHAT]2.0.CO;2

[B368] Woodman N, Croft DA (2026) Revisiting subfossil small-eared shrews of the genus *Cryptotis* Pomel, 1848 from near Copán, Honduras, with the description of a new species (Mammalia: Eulipotyphla: Soricidae). Occasional Papers, Museum of Texas Tech University 394: 1–28. https://ttumatt-ojs-ttu.tdl.org/ttumatt/article/view/23

[B369] Woodman N, Timm RM (1992) A new species of small-eared shrew from Honduras. Proceedings of the Biological Society of Washington 105(1): 1–12.

[B370] Woodman N, Timm RM (1999) Geographic variation in *Cryptotis goldmani*-group shrews. Fieldiana Zoology 91: 1–35. 10.5962/bhl.title.2669

[B371] Woodman N, Matson JO, McCarthy TJ, Eckerlin RP, Bulmer W, Ordóñez-Garza N (2012) Distributional records of shrews (Mammalia, Soricomorpha, Soricidae) from Northern Central America with the first record of *Sorex* from Honduras. Annals of Carnegie Museum 80(3): 207–237.

[B372] Young CJ, Jones JK (1984) *Reithrodontomys gracilis*. Mammalian Species 218: 1–3. 10.2307/3503973

